# Design, Synthesis, and Biological Activity of (*E*)‑α-Fluorovinylphosphonate-Based Reversible Cathepsin C Inhibitors

**DOI:** 10.1021/acs.joc.5c02923

**Published:** 2026-02-13

**Authors:** Marcin Kaźmierczak, Monika Bilska-Markowska, Katarzyna Wiśniewska, Małgorzata Pawełczak, Damian Nowak, Marcin Hoffmann

**Affiliations:** † Faculty of Chemistry, 467899 Adam Mickiewicz University in Poznań, Uniwersytetu Poznańskiego 8, Poznań 61-614, Poland; ‡ Center for Advanced Technologies, Adam Mickiewicz University in Poznań, Uniwersytetu Poznańskiego 10, Poznań 61-614, Poland; § Institute of Chemistry, Opole University, Opole 45-052, Poland

## Abstract

In this study, we describe the synthesis of novel dipeptide analogs of (*E*)-α-fluorovinylphosphonates, with a key step involving the Horner–Wadsworth–Emmons (HWE) reaction. The synthesized compounds were evaluated as potential reversible inhibitors of the cathepsin C enzyme. Comprehensive characterization of the target molecules was performed, and their inhibitory activity was assessed. Additionally, molecular docking studies were conducted to elucidate the binding interactions of the synthesized derivatives within the cathepsin C active site. The results highlight promising structural features for the design of effective enzyme inhibitors and provide a foundation for further optimization of fluorovinylphosphonate-based dipeptides.

## Graphical abstract

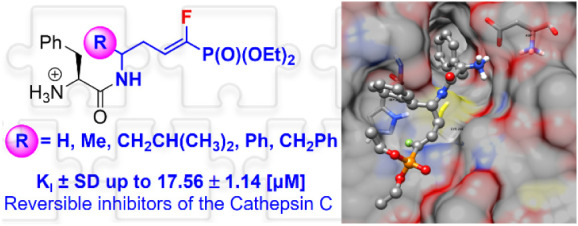

## Introduction

In recent decades, aminophosphonates and aminophosphonic acids have garnered significant attention due to their structural resemblance to naturally occurring phosphates and their role as bioisosteric mimics of phosphorus-containing amino acids.^1,2^ A notable characteristic of these compounds is the enhanced chemical stability of the phosphonate moiety, which exhibits marked resistance to enzymatic hydrolysis under physiological conditions, particularly by phosphatases that typically cleave phosphate ester bonds. Consequently, fluorinated aminophosphonates and related derivatives have emerged as a rapidly expanding focus in organic, bioorganic, and medicinal chemistry, as evidenced by the growing body of research in this area.^3,4^ Our study focuses on the structural modification of well-known inhibitors of cathepsin C,^5^ which belong to the class of dipeptide analogs of β- or γ-amino-α-hydroxyphosphonates **1**,**2** (Figure 1).^6^ These compounds are recognized for their strong affinity toward cysteine proteases. Cathepsin C is a cysteine protease that plays a pivotal role in the regulation of immune system function.^7^ Hyperactivation of cathepsin C has been linked to the progression of inflammatory and autoimmune disorders, underscoring the need for effective inhibitors of this enzyme.^8^ By introducing targeted structural modifications, we aim to explore structure–activity relationships that could enhance the inhibitory potency, selectivity, and pharmacological properties of these molecules. In our earlier work, we demonstrated that dipeptide fluorophosphonate analogs **3,4** display only moderate inhibitory activity against cathepsin C (Figure 1).^9^ Building on these findings, we have now introduced an additional methylene bridge together with a fluorovinyl substituent into the studied compounds **5**, in comparison to derivative **2** (Figure 1), which is expected to enhance conformational stability through both electronic and steric effects.^10^ This approach is particularly relevant, as fluorovinyl derivatives are well documented in the literature as components of protease inhibitors, where increased molecular rigidity improves complementarity with the enzyme active site.^11,12^

**Figure 1:**
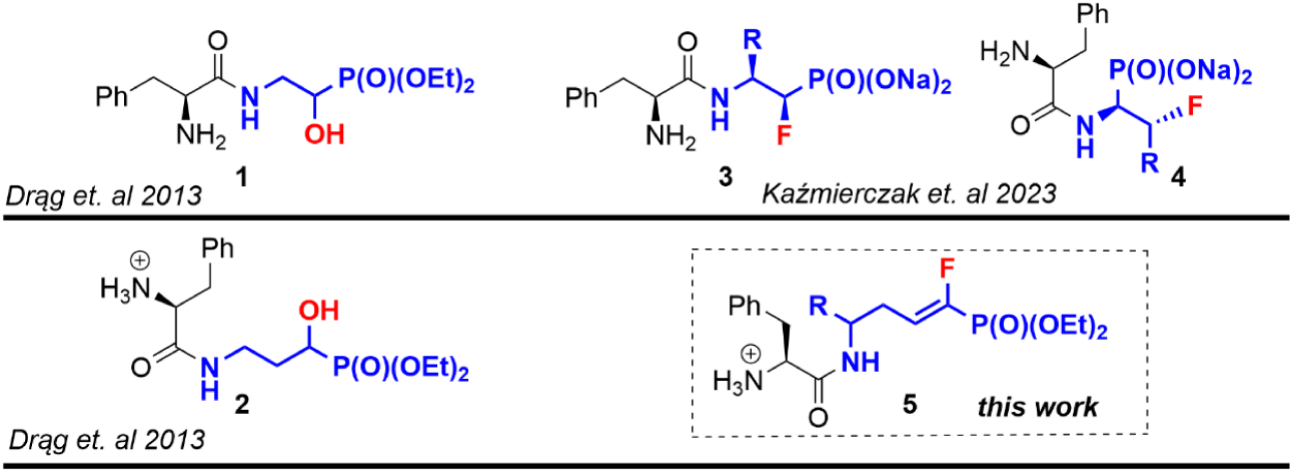
Representative examples of known and potential cathepsin C inhibitors.

## Results and Discussion

### Chemistry

The synthesis of dipeptide analogs of (*E*)-α-fluorovinylphosphonates **5** began with the conversion of β-amino acids **6** (β-alanine, R = H; *rac-*3-aminobutanoic acid, R = Me; *rac-*β-homoleucine, R = CH_2_CH­(CH_3_)_2_; *rac-*β-phenylalanine, R = Ph; *rac-*β-homophenylalanine, R = CH_2_Ph) into the corresponding amino alcohols **7** (Scheme 1). The amino group was then protected by reaction with Boc_2_O.^13^ The resulting *N*-Boc amino alcohols **8** were subsequently oxidized to the corresponding aldehydes **9** using the Dess–Martin periodinane method.^13^ The key step involved the introduction of the fluorovinyl motif through a Horner–Wadsworth–Emmons (HWE) olefination reaction, which we have previously employed successfully in related synthetic studies.^14–16^

**Scheme 1:**
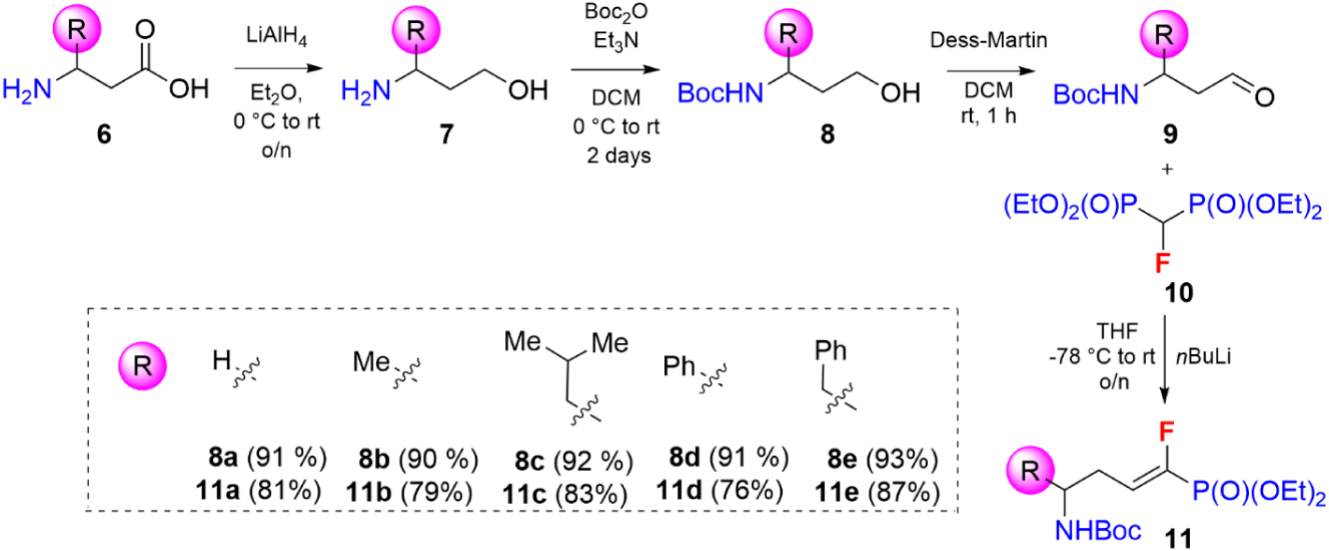
Synthetic Strategy for the Preparation of (*E*)-α-Fluorovinylphosphonates **11**

NMR analysis of the crude reaction mixtures indicated that, in all cases, the HWE reaction proceeded selectively, affording exclusively the (*E*)-α-fluorovinylphosphonates **11**. In each case, the ^3^ *J*_FH_ coupling constant was approximately 39 Hz, while ^3^ *J*_PH_ was around 8 Hz, clearly confirming the formation of the (*E*)-isomers (Figure 2).^17^ An additional advantage of the HWE reaction was the consistently high yields of the desired (*E*)-α-fluorovinylphosphonates **11**, reaching up to 87%. Importantly, the method developed in this work represents a significant advancement in the synthesis of such derivatives, as one of the previously reported approaches toward related systems afforded the corresponding (*E*)-α-fluorovinylphosphonates in only 8% yield.^18^ The use of *n*-BuLi, a very strong and essentially nonnucleophilic base, in the present case ensures efficient deprotonation at the α-carbon of the bisphosphonate **10**. At the same time, its minimal nucleophilicity toward the carbonyl group, combined with carefully controlled low-temperature conditions, results in significantly improved reaction efficiency and enhanced selectivity.

**Figure 2:**
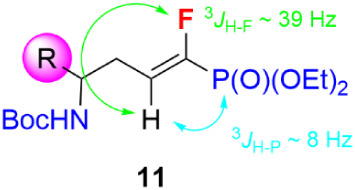
NMR analysis of the (*E*)-α-fluorovinylphosphonates **11**.

In the subsequent stage of the synthesis, after deprotection of the amino group with trifluoroacetic acid (TFA), the (*E*)-α-fluorovinylphosphonates **12** were subjected to peptide bond formation with *N*-Boc-l-phenylalanine **13** (Scheme 2). This reaction was carried out in the presence of EDCI and OxymaPure under basic conditions.^19,20^ In all cases, the reactions proceeded smoothly, affording the desired products in high yields of up to 89%. Except for derivative **14a**, (R = H), the remaining compounds **14b**–**e** were obtained as 1:1 mixtures of diastereomers, since the (*E*)-α-fluorovinylphosphonates **11** used in the synthesis were racemic mixtures. The next step involved the deprotection of the nitrogen atom. This reaction proceeded quantitatively in each case upon treatment with a 4 M solution of HCl in anhydrous CH_3_OH, which was freshly prepared immediately before use from SOCl_2_ and anhydrous methanol.^6^ The resulting dipeptide analogs of (*E*)-α-fluorovinylphosphonates **5** were fully characterized, confirming their structures. The synthesized compounds were evaluated for their biological activity, and the corresponding molecular structures were employed in docking studies at the enzyme active sites.

**Scheme 2:**
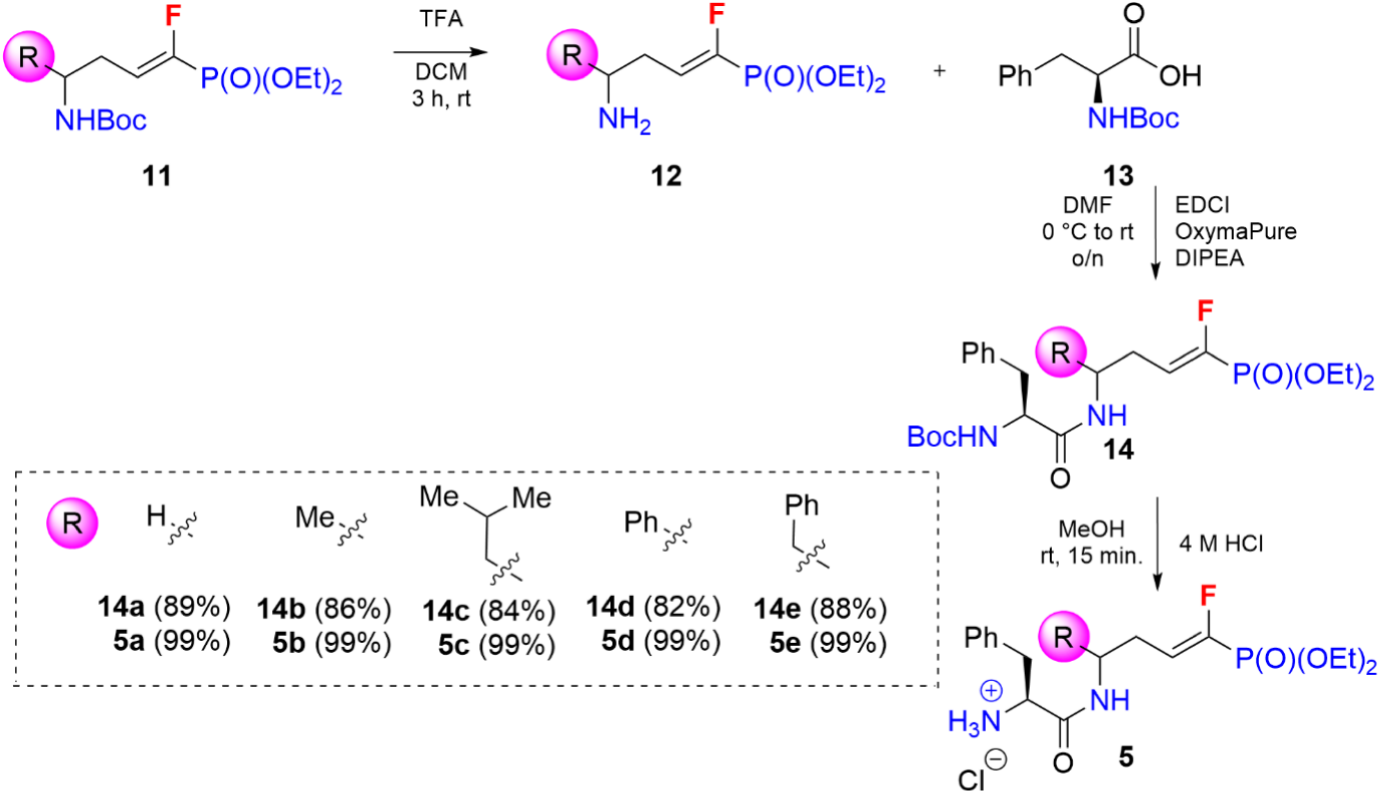
Synthetic Strategy for the Preparation of Dipeptide Analogs of (*E*)-α-Fluorovinylphosphonates

### Enzymatic Studies

The strategy for designing effective cathepsin C inhibitors took into account the structural and stereochemical requirements previously described for competitive cathepsin C inhibitors, including the presence of l-amino acids, a free amino group at the N-terminus of the peptide, and a blocked C-terminal group.^6,21,22^

The synthesized analogs with the general formula **14**, **5** shown in Table 1 were tested for cathepsin C. Compounds **5** were found to be moderate competitive inhibitors, binding slowly to the enzyme (Table 1, Figure 3). Consistent with previous reports, derivatives bearing a blocked amino group, such as Boc-protected amines, generally exhibited reduced inhibitory activity against cathepsin C.^23^ Across the tested concentration range (0.2–0.3 mM), compounds **14** showed negligible effects on enzyme activity, with none of the derivatives demonstrating measurable inhibition (Table 1). These results corroborate earlier findings and indicate that Boc protection of the amino group limits effective interaction with the enzyme’s active site.

**Table 1:**
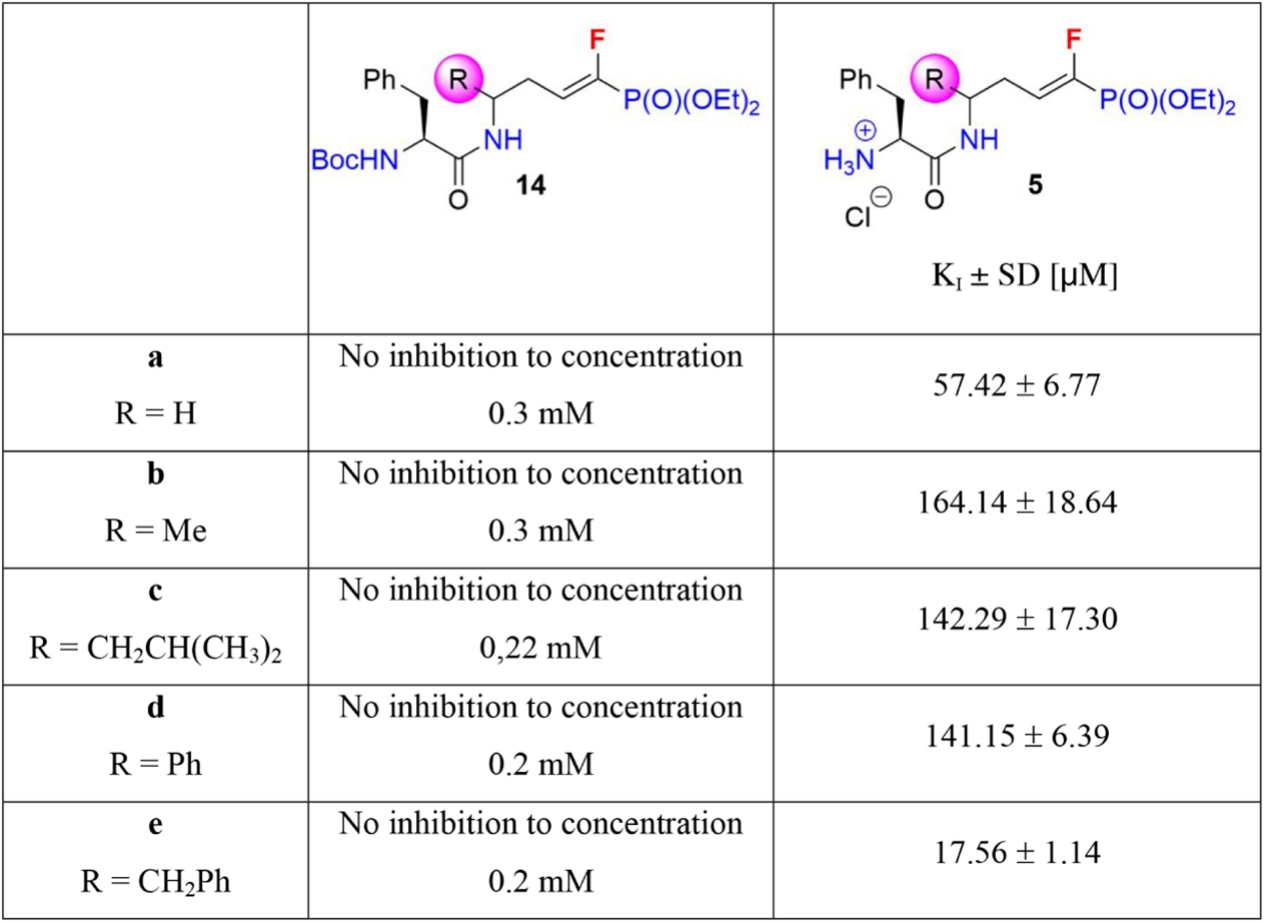
Inhibitory Constants of the Studied Dipeptide Analogs of (*E*)-α-Fluorovinylphosphonates towards Cathepsin C

**Figure 3:**
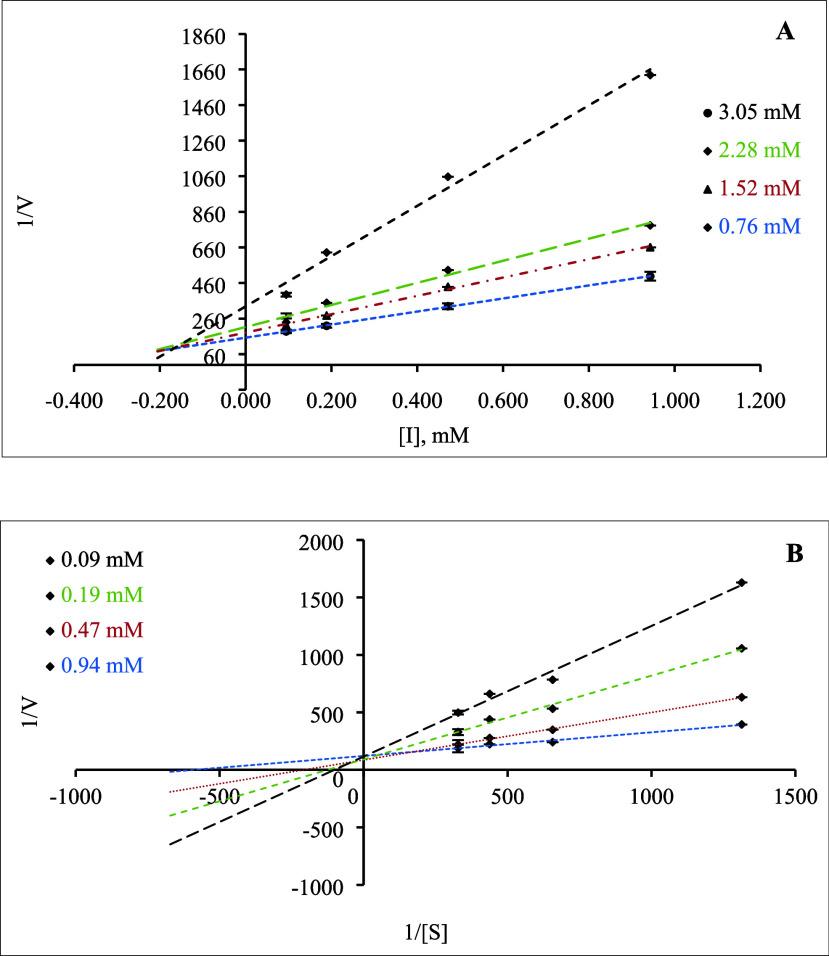
Effect of compound **5a** on cathepsin C activity (Dixon plot—A, Lineweaver–Burk plot—B). Inhibitor solution at appropriate final concentrations (0.09, 0.19, 0.47, and 0.94 mM) and enzyme solution (0.021 mg/mL) were preincubated for 30 min at 37 °C in 100 mM acetate buffer at pH 5 containing 1 mM EDTA-Na_2_, 1 mM DTT, 30 mM NaCl. The substrate Gly-l-Phe-p-nitroanilide was used at final concentrations of 0.76, 1.52, 2.28, and 3.05 mM. The enzymatic reaction was initiated by adding the substrate solution to the enzyme and inhibitor solutions. The absorbance of the hydrolysis reaction product was measured using a Jasco 730 UV–Vis spectrophotometer for 10 min at 405 nm (the wavelength of maximum absorbance of the colored reaction product, p-nitroaniline) compared to a control sample containing no enzyme. All experiments were performed in triplicate. The graph presents the mean values of three measurements along with the standard deviations (SD).

The binding of the enzyme to the slow-binding inhibitor reached equilibrium at a slower rate. The progression curves obtained in the presence of this inhibitor were exponential (Figure 4).

**Figure 4:**
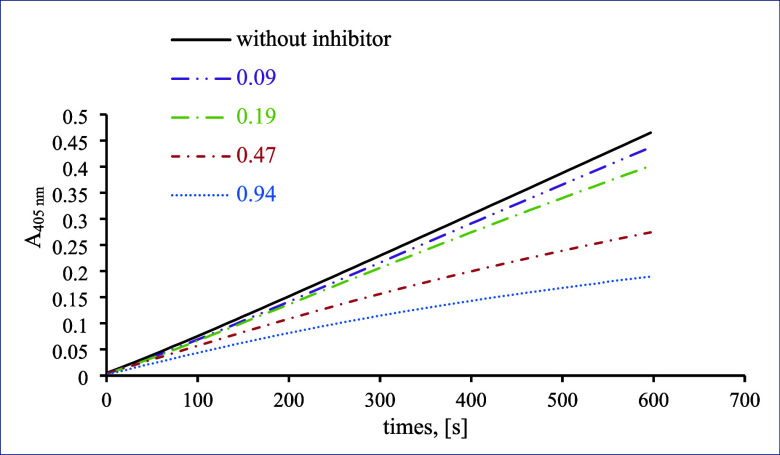
Course of the Gly-l-Phe-pNA hydrolysis reaction catalyzed by cathepsin C in the presence of increasing concentrations of compound **5a**.

A linear increase in the concentration of the enzymatic reaction product in the presence of the slow-binding inhibitor could be achieved by preincubating the enzyme with the inhibitor for 30 min before adding the substrate (Figure 5).

**Figure 5:**
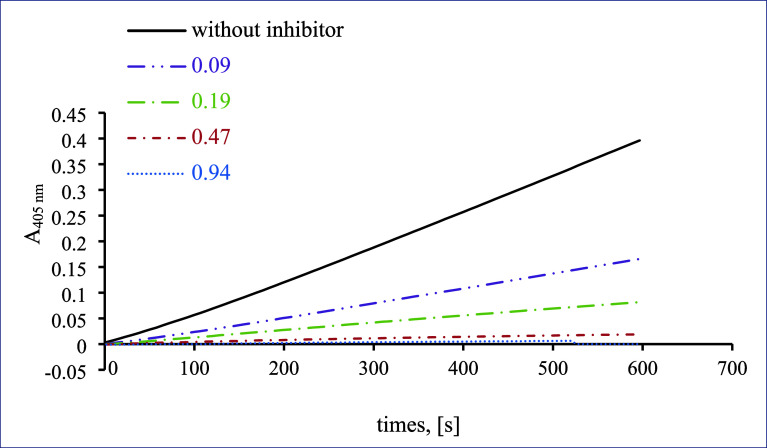
Course of the Gly-l-Phe-pNA hydrolysis reaction catalyzed by cathepsin C in the presence of increasing concentrations of compound **5a** after preincubation of the enzyme with a potential inhibitor.

From the test group of potential inhibitors, compound **5a** was a good one, which is substituted with a β-alanine, residue at the R position (R = H). The inhibition constant determined for this compound was 57.42 μM. This observation aligns with earlier reports, in which the most active inhibitors were dipeptide derivatives of β- or γ-amino-α-hydroxyphosphonates **1**, **2** bearing a first amino acid residue with the substituent R = H.^6^ The best inhibitor was compound **5e** with an inhibition constant of 17.56 μM. Compound **5e** had a *rac-*β-homophenylalanine residue substituted at the R position (R = CH_2_Ph). In a study on dipeptidyl nitriles, promising results were also reported for the derivative bearing a first amino acid residue with the substituent R = CH_2_Ph.^24^ This finding suggests that the presence of a bulky benzyl-like (CH_2_Ph) substituent at this position may contribute favorably to the enzyme–inhibitor interactions and enhance overall binding affinity.

### Molecular Docking

The results of molecular docking for the investigated compounds with the 1K3B protein domain (PDB ID)^25,26^ were summarized in Table 2. Lower (more negative) binding energies correspond to more stable ligand–receptor complexes. As shown in Table 2, the binding energies ranged from −7.2 to −6.4 kcal/mol, with most compounds clustering around −6.8 kcal/mol, suggesting comparable binding affinities. Among the tested derivatives, (*S*,*S*)-**5d** (R = Ph) and (*S*,*S*)-**5e** (R = CH_2_Ph) exhibited the lowest binding energy (−7.2 kcal/mol), indicating the highest affinity, whereas (*S*,*R*)-**5c** (R = CH_2_CH­(CH_3_)_2_) had the highest binding energy (−6.4 kcal/mol), suggesting lower affinity.

**Table 2:** Binding Energies of Compounds Docked to the 1K3B Protein Domain

Compound	Binding energy [kcal/mol]	Ligand efficiency [kcal/mol]
(*S*)-**5a** R = H	–6.7	–0.268
(*S,S*)-**5b** R = Me	–6.8	–0.262
(*S,R*)-**5b** R = Me	–6.5	–0.250
(*S,S*)-**5c** R = CH_2_CH(CH_3_)_2_	–6.8	–0.234
(*S,R*)-**5c** R = CH_2_CH(CH_3_)_2_	–6.4	–0.221
(*S,S*)-**5d** R = Ph	–7.2	–0.232
(*S,R*)-**5d** R = Ph	–7.0	–0.226
(*S,S*)-**5e** R = CH_2_Ph	–7.2	–0.225
(*S,R*)-**5e** R = CH_2_Ph	–6.9	–0.216

Ligand efficiencies were calculated by dividing the binding energy by the number of heavy (nonhydrogen) atoms, thereby normalizing for ligand size and enabling direct comparison across molecules with different structures. The atom counts for each structure are as follows: **5a**, R = H (25); **5b**, R = Me (26 each); **5c**, R = CH_2_CH­(CH_3_)_2_ (29 each); **5d**, R = Ph (31 each); **5e**, R = CH_2_Ph (32 each).

The calculated ligand efficiencies, summarized in Table 2, showed that the novel derivatives typically exhibited values around −0.237 kcal/mol per atom. Among these, (*S*,*R*)-**5e** (R = CH_2_Ph), which contains 32 atoms and has a binding energy of −6.9 kcal/mol, displayed the lowest (worst) ligand efficiency (−0.216 kcal/mol per atom). In contrast, (*S*)-**5a** (R = H), with 25 atoms and a binding energy of −6.7 kcal/mol, showed the highest (best) efficiency at −0.268 kcal/mol per atom. These results highlighted the favorable binding energy relative to ligand size for the new compounds, which is a key consideration in rational drug design.

Analysis of protein–ligand interactions revealed critical distances between the ligands and key catalytic residues in the 1K3B active site (Table 3). The catalytic triad of cathepsin C comprises ASP1, CYS234, and HIS381, with CYS234 serving as the nucleophilic residue. The distances to these residues provide insight into the binding mode and potential catalytic interactions of each compound.

**Table 3:** Distances between the Ligand and the ASP1, CYS234, and HIS381 Residues

Compound	ASP1 distance [Å]	CYS234 distance [Å]	HIS381 distance [Å]
(*S*)-**5a** R = H	2.16	3.05	3.17
(*S,S*)-**5b** R = Me	2.12	3.03	3.18
(*S,R*)-**5b** R = Me	2.06	3.19	2.74
(*S,S*)-**5c** R = CH_2_CH(CH_3_)_2_	1.92	3.06	3.53
(*S,R*)-**5c** R = CH_2_CH(CH_3_)_2_	2.01	3.04	3.33
(*S,S*)-**5d** R = Ph	2.65	3.07	3.25
(*S,R*)-**5d** R = Ph	2.27	3.07	3.25
(*S,S*)-**5e** R = CH_2_Ph	2.12	2.69	3.28
(*S,R*)-**5e** R = CH_2_Ph	2.52	3.12	3.40

Among all tested compounds, (*S*,*S*)-**5c** (R = CH_2_CH­(CH_3_)_2_) exhibited the closest interaction with ASP1 (1.92 Å), while (*S*,*S*)-**5e** (R = CH_2_Ph) showed the shortest distance to CYS234 (2.69 Å), the catalytic nucleophile. The closest interaction with HIS381 was observed for (*S*,*R*)-**5b** (R = Me) at 2.74 Å. Notably, (*S*,*R*)-**5b** demonstrated the best overall positioning with an average distance of 2.66 Å across all three catalytic residues, suggesting optimal engagement with the active site. The (*S*,*S*)-**5e** derivative, despite having lower ligand efficiency, achieved the closest proximity to CYS234, which may be critical for inhibitory activity given the nucleophilic role of this cysteine in the catalytic mechanism.

Figure 6 illustrates the interactions between (*S,S*)-**5e** (R = CH_2_Ph) and the 1K3B protein domain. Panel (a), generated using Proteins Plus,^27^ presented a 2D depiction of hydrogen bonds (black dashed lines). Panel (b), created with Chimera 1.16,^28^ depicted hydrogen bond distances (green dashed lines). Figure 7 shows all investigated ligands within the binding site of the 1K3B protein domain, illustrating their relative orientations and overlap within the enzyme’s active site.

**Figure 6:**
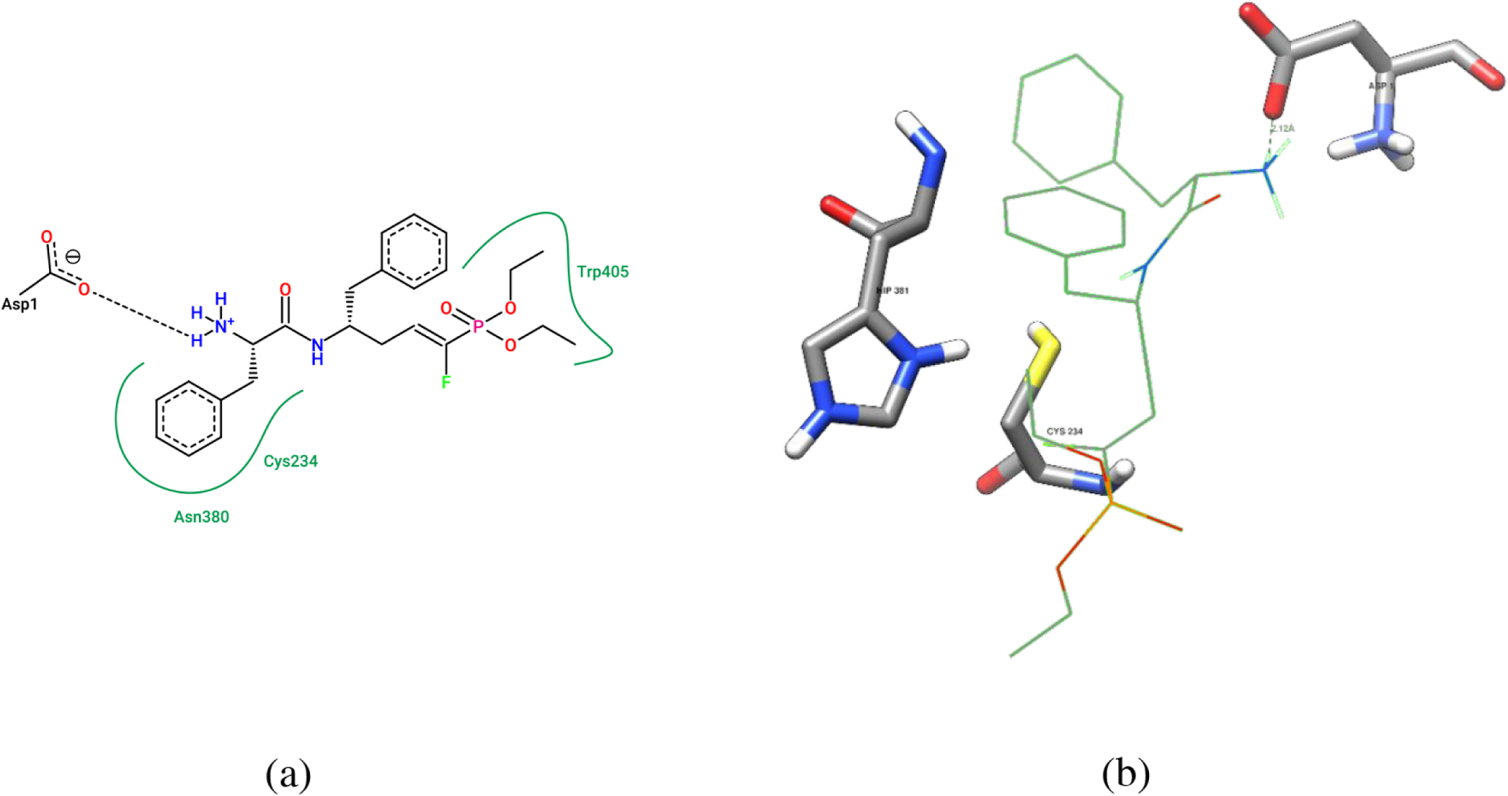
Potential interactions between ligand (*S*,*S*)-**5e** R = CH_2_Ph and 1K3B protein domain. The black dashed lines indicate hydrogen bonds (a). The green dashed lines (b) with green distances indicate the hydrogen bond distances.

**Figure 7:**
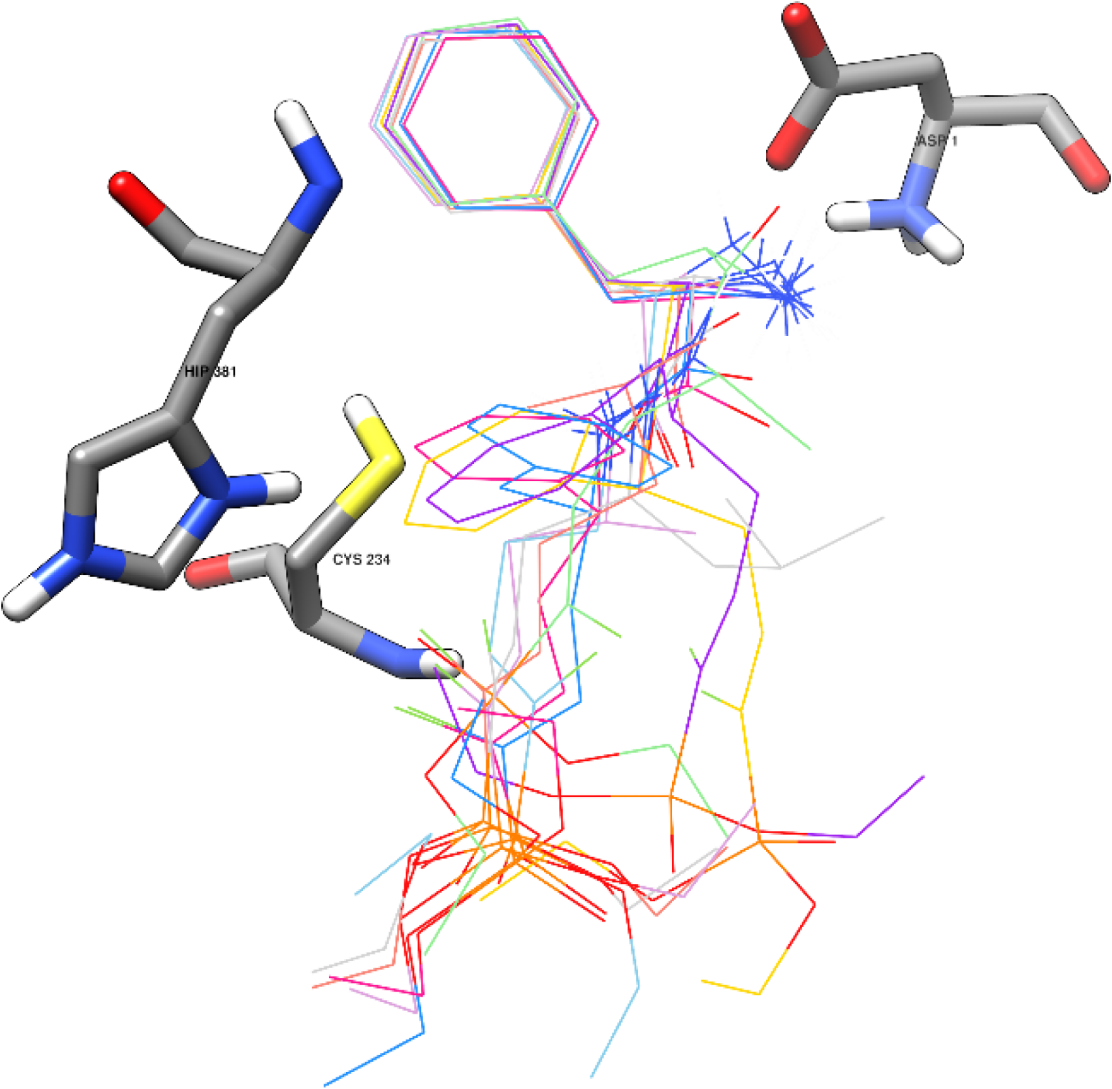
All investigated ligands at once in the binding site of 1K3B protein domain.

### Structure–Activity Relationship and Experimental Validation

The experimental inhibitory constants (Table 1) revealed striking potency differences across the series, with kinetic parameters that both validate and significantly refine the molecular docking predictions. Compound **5e** (R = CH_2_Ph) emerged as the most potent inhibitor with a K_I_ of 17.56 ± 1.14 μM, displaying remarkable selectivity within this series. This superior potency correlates directly with the computational prediction of the strongest binding energy (−7.2 kcal/mol) and, critically, the shortest distance to the catalytic nucleophile CYS234 (2.69 Å in the (*S*,*S*)-**5e** conformation). The benzyl substituent appears to facilitate optimal positioning within the active site, enabling both stabilizing interactions with aromatic residues and proximal engagement with the sulfhydryl group of CYS234, the residue essential for proteolytic catalysis.

In striking contrast to the docking predictions, compound **5a** (R = H), which demonstrated the highest ligand efficiency (−0.268 kcal/mol per atom), showed moderate inhibitory potency with a K_I_ of 57.42 ± 6.77 μM- approximately 3.3-fold weaker than **5e**. While the unsubstituted hydrogen at the R position minimizes steric bulk and maximizes ligand efficiency by docking metrics, the experimental data indicate that this simple derivative fails to achieve the precise spatial orientation and hydrophobic stabilization required for potent enzyme inhibition. The lack of hydrophobic interactions that larger substituents provide appears to outweigh the favorable entropy considerations associated with ligand efficiency.

The three bulky derivatives, **5b** (R = Me), **5c** (R = CH_2_CH­(CH_3_)_2_), and **5d** (R = Ph), displayed dramatically reduced potency despite their favorable binding energies predicted by docking. These compounds showed K_I_ values exceeding 140 μM (**5d**: 141.15 ± 6.39 μM; **5c**: 142.29 ± 17.30 μM; **5b**: 164.14 ± 18.64 μM), representing a 9.3-fold difference in potency compared to **5e**. This pronounced loss of activity, despite docking scores comparable to or better than those of **5e**, reveals a critical discrepancy between computational binding predictions and actual enzyme inhibition. The steric incompatibility introduced by methyl, isopropylmethyl, and phenyl substituents appears to distort the optimal binding geometry, preventing the inhibitor from achieving the precise alignment required for transition state stabilization or covalent modification of the catalytic cysteine. The poor tolerance for bulk at the R position suggests that the active site of cathepsin C provides a highly constrained binding pocket that cannot accommodate even modestly sized groups without geometric penalty.

The potency ranking revealed by kinetic analysis (**5e** > **5a** > **5d** ≈ **5c** > **5b**) diverges substantially from predictions based on simple ligand efficiency metrics but exhibits impressive alignment with the distance-based analysis of catalytic residue interactions. Notably, the (*S*,*S*)-**5e** conformation achieved the closest proximity to CYS234 (2.69 Å), and this compound proved to be the most potent inhibitor. This observation suggests that for protease inhibitors targeting catalytic nucleophiles, proximity to the catalytic sulfhydryl group may be a more predictive parameter than bulk binding energy or ligand efficiency alone.

### Fluorine Interaction Analysis

The fluorine atoms in compounds (*S*,*S*)-**5b**, (*S*,*S*)-**5c**, (*S*,*S*)-**5d**, and (*S*,*S*)-**5e** occupy unique positions within the binding pocket and establish favorable interactions with key residues. Analysis of ligand–receptor distances indicates several interaction modes, which are described in detail in the Supporting Information. The improvement in binding affinity associated with fluorine substitution reflects the cumulative stabilization arising from multiple interaction modes rather than a single dominant interaction type. The fluorine atom, through its high electronegativity and favorable geometric positioning, optimizes the binding pocket microenvironment and enhances overall ligand–protein complementarity.

## Conclusion

The synthesis of novel dipeptide analogs of (*E*)-α-fluorovinylphosphonates via the Horner–Wadsworth–Emmons reaction yielded compounds with promising inhibitory properties against cathepsin C. Detailed chemical characterization and biological activity assessments confirmed their potential as reversible enzyme inhibitors. The comprehensive analysis of computational docking, molecular efficiency metrics, and experimental enzyme kinetics elucidates key design principles for cathepsin C inhibitors. While absolute binding energy provides some predictive value, the correlation with experimental K_I_ is substantially improved by considering: (1) ligand efficiency normalized to molecular size, (2) precise distances to individual catalytic residues, and (3) the steric constraints of the active site. The exceptional potency of compound **5e** (K_I_ = 17.56 μM), coupled with its optimal positioning relative to CYS234 and favorable overall binding energy, demonstrates that strategic placement of appropriately sized hydrophobic substituents can substantially enhance inhibitor efficacy. Conversely, the weak performance of bulky derivatives despite favorable docking scores emphasizes that computational models must be validated against experimental data, as the three-dimensional atomic-level details of enzyme–inhibitor interactions can impose stringent geometric requirements not fully captured by simple scoring functions. The integration of kinetic measurements with structure-based design, as demonstrated here, provides a robust framework for rational optimization of protease inhibitors and highlights the importance of experimental validation in guiding structure–activity relationships.

## Experimental Section

### General Procedures

^1^H NMR, ^13^C NMR, ^19^F NMR and ^31^P NMR spectra were performed on Bruker ASCEND 400 (400 MHz) or Bruker ASCEND 600 (600 MHz) spectrometers. Chemical shifts of ^1^H NMR were expressed in parts per million downfield from tetramethylsilane (TMS) as an internal standard (δ = 0) in CDCl_3_ or MeOD. Chemical shifts of ^19^F, ^31^P NMR were expressed in parts per million. Structural assignments were made with additional information from gHSQC experiments. High-resolution mass spectra were recorded by electron spray (MS-ESI) techniques using QToF Impact HD Bruker spectrometer. Solvents were dried with CaH_2_·(CH_2_Cl_2_), NaH (Et_2_O), P_2_O_5_ (MeOH) and distilled under argon atmosphere. All moisture-sensitive reactions were carried out under an argon atmosphere using oven-dried glassware. TLC was performed on Merck Kieselgel 60-F254 with EtOAc/*n*-hexane and MeOH/CHCl_3_ as developing systems, and products were detected by inspection under UV light (254 nm) and with a solution of potassium permanganate. The β-amino acids **6** used as substrates for the syntheses were purchased from Merck.

### Synthesis of **7**

β-Amino acids *rac*-**6** (5 mmol, 1 equiv) were added to a stirred suspension of lithium aluminum hydride (15 mmol, 3 equiv) in diethyl ether (15 mL) under an argon atmosphere at 0 °C. After 15 min, the cooling bath was removed, and the reaction mixture was stirred overnight at room temperature. The mixture was then cooled again to 0 °C, and the reaction was quenched by the cautious, dropwise addition of 0.5 mL of water, followed by 1 mL of 15% aqueous KOH and an additional 1.5 mL of water. The resulting aluminum salts were filtered off, and the filtrate was concentrated under reduced pressure. The crude residues were taken without any further purification for the next reactions.

### Synthesis of **8**

A solution of Boc_2_O (4 mmol, 1.0 equiv., 873 mg) in CH_2_Cl_2_ (5 mL) was added to a stirred mixture of *rac-* **7** (4 mmol, 1.0 equiv) and triethylamine (4.8 mmol, 1.2 equiv., 669 μL) in CH_2_Cl_2_ (15 mL) at 0 °C. The reaction mixture was then allowed to warm to room temperature and stirred for 2 days. Subsequently, approximately half of the solvent was evaporated, and 1% aqueous HCl (15 mL) was added. The mixture was stirred for 10 min, and the layers were separated. The aqueous phase was extracted with CH_2_Cl_2_ (3 × 20 mL), and the combined organic extracts were washed with water, dried over Na_2_SO_4_, filtered, and concentrated under reduced pressure. The crude product was purified by flash column chromatography.

#### *Tert*-butyl (3-Hydroxypropyl)­carbamate **(** *rac-* **8a**)

According to the general procedure, *rac-* **8a** was obtained using *rac-* **7a** (4 mmol, 1.0 equiv., 300 mg). Flash column chromatography on silicagel *n*-hexane/ethyl acetate gradient (from 95:10 to 60:40) gave a colorless oil (638 mg, 91%): ^**1**^ **H NMR** (400 MHz, Chloroform-*d*) δ = 4.76 (br.s, 1H, NH), 3.66 (q, *J* = 5.8 Hz, 2H, CH_2_), 3.29 (q, *J* = 6.3 Hz, 2H, CH_2_), 2.92 (br.s, 1H, OH), 1.70–1.61 (m, 2H, CH_2_), 1.45 (s, 9H, (CH_3_)_3_).

The NMR spectroscopic data were in good agreement with previous research.^29^

#### *Tert*-butyl (4-Hydroxybutan-2-yl)­carbamate (*rac-* **8b**)

According to the general procedure, *rac-* **8b** was obtained using *rac-* **7b** (4 mmol, 1.0 equiv., 357 mg). Flash column chromatography on silicagel *n*-hexane/ethyl acetate gradient (from 95:10 to 60:40) gave a colorless oil (681 mg, 90%): ^1^H NMR (600 MHz, Chloroform-*d*) δ = 4.42 (br.s, 1H, NH), 3.97–3.81 (m, 1H, CH), 3.68–3.57 (m, 2H, CH_2_), 3.41 (br.s, 1H, OH), 1.91–1.74 (m, 1H, CH_a_H_b_), 1.72–1.54 (m, 1H, CH_a_ H_b_), 1.45 (s, 9H, (CH_3_)_3_), 1.19 (d, *J* = 6.7 Hz, 3H, CH_3_).

The NMR spectroscopic data were in good agreement with previous research.^30^

#### *Tert*-butyl (1-Hydroxy-5-methylhexan-3-yl)­carbamate (*rac-* **8c**)

According to the general procedure, *rac-* **8c** was obtained using *rac-* **7c** (4 mmol, 1.0 equiv., 525 mg). Flash column chromatography on silicagel *n*-hexane/ethyl acetate gradient (from 95:10 to 60:40) gave a colorless oil (851 mg, 92%): ^1^H NMR (400 MHz, Chloroform-*d*) δ *=* 4.32 (br.d, *J* = 9.2 Hz, 1H, NH), 3.96–3.76 (m, 1H, CHNH), 3.69–3.52 (m, 3H, CH_2_OH, OH), 1.88–1.75 (m, 1H, CH_a_H_b_CH_2_OH), 1.75–1.64 (m, 1H, CH­(CH_3_)_2_), 1.45 (s, 9H, (CH_3_)_3_), 1.39–1.26 (m, 2H, CH_2_CHNH), 1.27–1.15 (m, 1H, CH_a_ H_b_CH_2_OH), 0.92 (d, *J* = 2.9 Hz, 3H, CH_3_), 0.91 (d, *J* = 2.8 Hz, 3H, CH_3_). ^13^C­{/^1^H} NMR (101 MHz, Chloroform-*d*) δ *=* 157.2 (s, CO), 79.7 (s, C­(CH_3_)_3_), 58.7 (s, CH_2_OH), 45.2 (s, CHNH), 44.9 (s, CH_2_CHNH), 39.6 (s, CH_a_H_b_CH_2_OH), 28.3 (s, (CH_3_)_3_), 24.9 (s, CH­(CH_3_)_2_, 23.0 (s, CH_3_), 22.0 (s, CH_3_). HRMS (ESI) calcd. for C_12_H_25_NO_3_Na ([M + Na]^+^): 254.1732, found: 254.1731.

#### *Tert*-butyl (3-Hydroxy-1-phenylpropyl)­carbamate (*rac-* **8d**)

According to the general procedure, *rac-* **8d** was obtained using *rac-* **7d** (4 mmol, 1.0 equiv., 605 mg). Flash column chromatography on silicagel *n*-hexane/ethyl acetate gradient (from 95:10 to 60:40) gave a white solid (915 mg, 91%): ^1^H NMR (400 MHz, Chloroform-*d*) δ = 7.39–7.24 (m, 5H. Ar-H), 5.00 (br.s, *J* = 8.5 Hz, 1H, NH), 4.90 (br.s, 1H, OH), 3.70 (td, *J* = 7.3, 6.9, 3.6 Hz, 2H, CH_2_), 2.14–1.97 (m, 1H, CH_a_H_b_), 1.88–1.78 (m, 1H, CH_a_ H_b_), 1.44 (s, 9H, (CH_3_)_3_).

The NMR spectroscopic data were in good agreement with previous research.^31^

#### *Tert*-butyl (4-Hydroxy-1-phenylbutan-2-yl)­carbamate (*rac-* **8e**)

According to the general procedure, *rac-* **8e** was obtained using *rac-* **7e** (4 mmol, 1.0 equiv., 661 mg). Flash column chromatography on silicagel *n*-hexane/ethyl acetate gradient (from 95:10 to 60:40) gave a colorless oil (987 mg, 93%): ^1^H NMR (400 MHz, Chloroform-*d*) δ *=* 7.81–6.82 (m, 5H, Ar-H), 4.53 (br.d, *J* = 9.1 Hz, 1H, NH), 4.17–3.98 (m, 1H, CHNH), 3.69–3.54 (m, 2H, CH_2_OH), 2.81 (d, *J* = 6.7 Hz, 2H, CH_2_Ph), 1.96–1.74 (m, 1H, CH_a_H_b_CH_2_OH), 1.54–1.29 (m, 11H, (CH_3_)_3_, CH_a_ H_b_CH_2_OH). ^13^C­{/^1^H} NMR (101 MHz, Chloroform-*d*) δ *=* 156.8 (s, CO), 137.7, 129.2, 128.4, 126.5 (4 × s, Ar-C), 79.8 (s, C­(CH_3_)_3_), 58.9 (s, CH_2_OH), 48.0 (s, CHNH), 41.4 (s, CH_2_Ph), 37.8 (s, CH_a_H_b_CH_2_OH, 28.3 (s, (CH_3_)_3_). HRMS (ESI) calcd. for C_15_H_23_NO_3_Na ([M + Na]^+^): 288.1575, found: 288.1599.

### Synthesis of **10**

#### Tetraethyl Fluoromethylenebisphosphonate (**10**)

Colorless oil (836 mg, 51%): ^1^H NMR (400 MHz, Chloroform-*d*) δ = 5.01 (dt, *J* = 45.9, 13.6 Hz, 1H, CHF), 4.44–4.10 (m, 8H, 4 × OCH_2_CH_3_), 1.38 (td, *J* = 7.1, 1.3 Hz, 12H, 4 × OCH_2_CH_3_).

Tetraethyl fluoromethylenebisphosphonate **10** was prepared according to a previously published procedure.^32^ The spectroscopic data of the obtained compound were consistent with those previously reported by us.^14^

### Synthesis of **11**

Dess–Martin periodinane (2.4 mmol, 1.2 equiv., 1,02 g) was added to a stirred solution of *rac-* **8** alcohols (2 mmol, 1 equiv) in dichloromethane (15 mL) at room temperature. After 60 min, the reaction mixture was diluted with CH_2_Cl_2_ and a 1:1 mixture of saturated aqueous NaHCO_3_ and Na_2_S_2_O_3_ (30 mL total). The mixture was stirred for 10 min, and the layers were separated. The organic phase was dried over Na_2_SO_4_ and concentrated under reduced pressure to afford crude oils **9**, which were used directly without further purification.

Lithium tetraethyl fluoromethylenebisphosphonate was prepared by the addition of *n*-butyllithium (3.45 mmol, 1.3 equiv., 2 M in pentane, 1.7 mL) to a stirred solution of tetraethyl fluoromethylenebisphosphonate **10** (2.64 mmol, 1.3 equiv., 808 mg) in THF (1 mL) under an argon atmosphere at–78 °C. After 10 min, a solution of aldehydes **9** (2 mmol, 1.0 equiv) in THF (1 mL) was added dropwise. The reaction mixture was then allowed to warm to room temperature and stirred overnight. The reaction was quenched with saturated aqueous NH_3_Cl (10 mL), and the crude product was extracted with ethyl acetate (3 × 10 mL). The combined organic layers were dried over MgSO_4_, filtered, and concentrated under reduced pressure. The crude residue was purified by flash column chromatography.

#### *Tert*-butyl (*E*)-(4-(Diethoxyphosphoryl)-4-fluorobut-3-en-1-yl)­carbamate (**11a**)

According to the general procedure, **11a** was obtained using *rac-* **8a** (2 mmol, 1.0 equiv., 350 mg). Flash column chromatography on silicagel *n*-hexane/ethyl acetate gradient (from 95:5 to 50:50) gave a white solid (527 mg, 81%): ^1^H NMR (400 MHz, Chloroform-*d*) δ *=* 5.95 (dq, *J* = 39.2, 7.6 Hz, 1H, CHCFP), 4.84 (br. s, 1H, NH), 4.37–3.89 (m, 4H, 2 × OCH_2_CH_3_), 3.39–3.02 (m, 2H, CH_2_NH), 2.71–2.15 (m, 2H, CH_2_), 1.44 (s, 9H, (CH_3_)_3_), 1.37 (t, *J* = 7.1 Hz, 6H, 2 × OCH_2_CH_3_). ^13^C­{/^1^H} NMR (101 MHz, Chloroform-*d*) δ *=* 155.7 (s, CO), 151.4 (dd, *J* = 275.7, 235.4 Hz, CFP), 122.7 (dd, *J* = 28.7, 5.7 Hz, CHCFP), 79.2 (s, C­(CH_3_)_3_), 63.0 (d, *J* = 5.5 Hz, 2 × OCH_2_CH_3_), 39.0 (s, CH_2_NH), 28.2 (s, (CH_3_)_3_), 24.9 (d, *J* = 10.0 Hz, CH_2_), 16.1 (d, *J* = 6.2 Hz, 2 × OCH_2_ CH_3_). ^19^F NMR (377 MHz, Chloroform-*d*) δ *=* −129.37 (dd, *J* = 102.1, 39.1 Hz, 1F). ^31^P­{/^1^H} NMR (162 MHz, Chloroform-*d*) δ *=* 4.63 (d, *J* = 102.3 Hz, 1P). HRMS (ESI) calcd. for C_13_H_25_FNO_5_PNa ([M + Na]^+^): 348.1352, found: 348.1342.

#### *Tert*-butyl (*E*)-(5-(Diethoxyphosphoryl)-5-fluoropent-4-en-2-yl)­carbamate (*rac-* **11b**)

According to the general procedure, *rac-* **11b** was obtained using *rac-* **8b** (2 mmol, 1.0 equiv., 379 mg). Flash column chromatography on silicagel *n*-hexane/ethyl acetate gradient (from 95:5 to 50:50) gave a white solid (536 mg, 79%): ^1^H NMR (400 MHz, Chloroform-*d*) δ *=* 5.96 (dddd, *J* = 39.1, 7.7 Hz, 1H, CHCFP), 4.53 (br.s, 1H, NH), 4.23–4.08 (m, 4H, 2 × OCH_2_CH_3_), 3.85–3.75 (m, 1H, CHNH), 2.55–2.32 (m, 2H, CH_2_), 1.44 (s, 9H, (CH_3_)_3_), 1.36 (t, *J* = 7.1 Hz, 6H, 2 × OCH_2_CH_3_), 1.16 (d, *J* = 6.7 Hz, 3H, CH_3_). ^13^C­{/^1^H} NMR (101 MHz, Chloroform-*d*) δ *=* 155.0 (s, CO), 151.5 (dd, *J* = 275.5, 235.2 Hz, CFP), 122.0 (dd, *J* = 28.8, 5.7 Hz, CHCFP), 79.2 (s, C­(CH_3_)_3_), 63.0 (d, *J* = 5.5 Hz, OCH_2_CH_3_), 62.9 (d, *J* = 5.4 Hz, OCH_2_CH_3_), 45.4 (s, CHNH), 31.4–30.7 (m, CH_2_), 28.2 (s, (CH_3_)_3_), 20.4 (s, CH_3_), 16.1 (d, *J* = 6.2 Hz, 2 × OCH_2_ CH_3_). ^19^F NMR (377 MHz, Chloroform-*d*) δ *=* −129.34 (dd, *J* = 102.7, 39.1 Hz, 1F). ^31^P­{/^1^H} NMR (162 MHz, Chloroform-*d*) δ *=* 4.67 (d, *J* = 102.5 Hz, 1P). HRMS (ESI) calcd. for C_14_H_27_FNO_5_PNa ([M + Na]^+^): 362.1508, found: 362.1496.

#### *Tert*-butyl (*E*)-(1-(Diethoxyphosphoryl)-1-fluoro-6-methylhept-1-en-4-yl)­carbamate (*rac-* **11c**)

According to the general procedure, *rac-* **11c** was obtained using *rac-* **8c** (2 mmol, 1.0 equiv., 463 mg). Flash column chromatography on silicagel *n*-hexane/ethyl acetate gradient (from 95:5 to 50:50) gave a white crystalline oil (633 mg, 83%): ^1^H NMR (400 MHz, Chloroform-*d*) δ *=* 5.97 (dq, *J* = 39.2, 7.7 Hz, 1H, CHCFP), 4.58–4.44 (m, 1H, NH), 4.25–4.03 (m, 4H, 2 × OCH_2_CH_3_), 3.91–3.69 (m, 1H, CHNH), 2.55–2.44 (m, 1H, CH_a_H_b_), 2.44–2.29 (m, 1H, CH_a_ H_b_), 1.74–1.59 (m, 1H, CH­(CH_3_)_2_), 1.43 (s, 9H, (CH_3_)_3_), 1.41–1.28 (m, 7H, 2 × OCH_2_CH_3_, CH_a_H_b_CH­(CH_3_)_2_), 1.24 (ddd, *J* = 14.7, 7.1, 3.5 Hz, 1H, CH_a_ H_b_CH­(CH_3_)_2_), 0.91 (d, *J* = 6.7, 6H, 2 × CH_3_). ^13^C­{/^1^H} NMR (101 MHz, Chloroform-*d*) δ *=* 155.2 (s, CO), 151.3 (dd, *J* = 275.0, 235.2 Hz, CFP), 122.1 (dd, *J* = 28.9, 5.4 Hz, CHCFP), 79.0 (s, C­(CH_3_)_3_), 62.9 (d, *J* = 5.2 Hz, OCH_2_CH_3_), 62.8 (d, *J* = 5.3 Hz, OCH_2_CH_3_), 47.6 (s, CHNH), 43.7 (s, CH_2_CH­(CH_3_)_2_), 29.9 (d, *J* = 10.2 Hz, CH_2_), 28.2 (s, (CH_3_)_3_), 24.6 (s, CH­(CH_3_)_2_, 22.8 (s, CH_3_), 21.9 (s, CH_3_), 16.0 (d, *J* = 6.2 Hz, 2 × OCH_2_ CH_3_). ^19^F NMR (377 MHz, Chloroform-*d*) δ *=* −129.44 (dd, *J* = 103.2, 39.4 Hz, 1F). ^31^P­{/^1^H} NMR (162 MHz, Chloroform-*d*) δ *=* 4.71 (d, *J* = 103.2 Hz, 1P). HRMS (ESI) calcd. for C_17_H_33_FNO_5_PNa ([M + Na]^+^): 404.1978, found: 404.1975.

#### *Tert*-butyl (*E*)-(4-(Diethoxyphosphoryl)-4-fluoro-1-phenylbut-3-en-1-yl)­carbamate (*rac-* **11d**)

According to the general procedure, *rac-* **11d** was obtained using *rac-* **8d** (2 mmol, 1.0 equiv., 503 mg). Flash column chromatography on silicagel *n*-hexane/ethyl acetate gradient (from 95:5 to 50:50) gave a white crystalline oil (631 mg, 76%): ^1^H NMR (600 MHz, Chloroform-*d*) δ *=* 7.47–7.11 (m, 5H, Ar-H), 5.89 (dddd, *J* = 38.8, 7.6 Hz, 1H, CHCFP), 5.00 (d, *J* = 8.1 Hz, 1H, NH), 4.79 (br.s, 1H, CHNH), 4.14–3.88 (m, 4H, 2 × OCH_2_CH_3_), 2.77 (d, *J* = 7.9 Hz, 2H, CH_2_), 1.42 (s, 9H, (CH_3_)_3_), 1.31 (t, *J* = 7.1 Hz, 3H, OCH_2_CH_3_), 1.28 (t, *J* = 7.1 Hz, 3H, OCH_2_CH_3_). ^13^C­{/^1^H} NMR (151 MHz, Chloroform-*d*) δ *=* 154.9 (s, CO), 151.5 (dd, *J* = 276.3, 233.6 Hz, CFP), 141.0, 128.6, 127.5, 126.2 (4x s, Ar-C), 121.7 (dd, *J* = 28.8, 5.3 Hz, CHCFP), 79.6 (s, C­(CH_3_)_3_), 63.0 (d, *J* = 4.9 Hz, OCH_2_CH_3_), 62.9 (d, *J* = 5.5 Hz, OCH_2_CH_3_), 53.6 (s, CHNH), 31.0 (s, CH_2_), 28.2 (s, (CH_3_)_3_), 16.1 (d, *J* = 5.9 Hz, OCH_2_ CH_3_), 16.0 (d, *J* = 5.6 Hz, OCH_2_ CH_3_). ^19^F NMR (565 MHz, Chloroform-*d*) δ *=* −128.39 (dd, *J* = 102.0, 38.5 Hz, 1F). ^31^P­{/^1^H} NMR (243 MHz, Chloroform-*d*) δ *=* 4.48 (d, *J* = 101.4 Hz, 1P). HRMS (ESI) calcd. for C_19_H_29_FNO_5_PNa ([M + Na]^+^): 424.1665, found: 424.1663.

#### *Tert*-butyl (*E*)-(5-(Diethoxyphosphoryl)-5-fluoro-1-phenylpent-4-en-2-yl)­carbamate (*rac-* **11e**)

According to the general procedure, *rac-* **11e** was obtained using *rac-* **8e** (2 mmol, 1.0 equiv., 531 mg). Flash column chromatography on silicagel *n*-hexane/ethyl acetate gradient (from 95:5 to 50:50) gave a white solid (723 mg, 87%): ^1^H NMR (400 MHz, Chloroform-*d*) δ *=* 7.44–7.00 (m, 5H, Ar-H), 5.98 (dq, *J* = 39.1, 7.6 Hz, 1H, CHCFP), 4.44 (br.s, 1H, NH), 4.28–4.05 (m, 4H, 2 × OCH_2_CH_3_), 4.06–3.87 (m, 1H, CHNH), 2.88–2.70 (m, 2H, CH_2_Ph), 2.56–2.41 (m, 1H, CH_a_H_b_), 2.34 (dtt, *J* = 15.2, 7.8, 2.2 Hz, 1H, CH_a_ H_b_), 1.40 (s, 9H, (CH_3_)_3_), 1.39–1.32 (m, 6H, 2 × OCH_2_CH_3_). ^13^C­{/^1^H} NMR (101 MHz, Chloroform-*d*) δ *=* 155.1 (s, CO), 151.7 (dd, *J* = 275.7, 235.1 Hz, CFP), 137.3, 129.3, 128.5, 126.6 (4*s*, Ar-C), 122.1 (dd, *J* = 28.8, 5.5 Hz, CHCFP), 79.4 (s, C­(CH_3_)_3_), 63.1 (d, *J* = 3.0 Hz, OCH_2_CH_3_), 63.0 (d, *J* = 3.2 Hz, OCH_2_CH_3_), 50.7 (s, CHNH), 40.7 (s, CH_2_Ph), 28.5 (s, CH_2_), 28.3 (s, (CH_3_)_3_), 16.2 (d, *J* = 6.3 Hz, 2 × OCH_2_ CH_3_). ^19^F NMR (377 MHz, Chloroform-*d*) δ *=* −128.72 (dd, *J* = 102.4, 38.9 Hz, 1F). ^31^P­{/^1^H} NMR (162 MHz, Chloroform-*d*) δ *=* 4.57 (d, *J* = 102.2 Hz, 1P). HRMS (ESI) calcd. for C_20_H_31_FNO_5_PNa ([M + Na]^+^): 438.1821, found: 438.1815.

### Synthesis of **12**

Derivatives **11** (1 equiv) were dissolved in an excess of a 1:1 CH_2_Cl_2_/TFA mixture (1 mL) and stirred at room temperature for 3 h. The solvents were then evaporated under reduced pressure, and the resulting oily residue was treated with a saturated aqueous NaHCO_3_ solution. The products were extracted with CH_2_Cl_2_ (3 × 10 mL); the combined organic layers were dried over anhydrous MgSO_4_, filtered, and concentrated under reduced pressure. The obtained compounds were used directly in subsequent syntheses without additional purification.

### Synthesis of **14**

Boc-Phe-OH **13** (1.0 equiv., 265 mg), OxymaPure (1.1 mmol, 1.1 equiv., 156 mg), and EDCI (1.1 mmol, 1.1 equiv., 211 mg) were dissolved in DMF at 0 °C under an argon atmosphere. The mixture was stirred for 5 min at 0 °C to preactivate the carboxylic acid and form the corresponding active ester. Subsequently, DIPEA (1.0 mmol, 1.0 equiv) and amines **12** (1.0 mmol, 1.0 equiv., 238 μL) were added sequentially. The reaction was maintained at 0 °C for 30 min and then stirred at room temperature for 18 h. Upon completion, the reaction mixture was diluted with ethyl acetate (20 mL) and washed successively with saturated aqueous NaHCO_3_, 1 M HCl, and saturated NaCl solutions. The organic layer was dried over MgSO_4_ (or Na_2_SO_4_), filtered, and concentrated under reduced pressure. The crude product was purified by flash column chromatography. Before the biological evaluation, the compounds were subjected to lyophilization.

#### *Tert*-butyl (*S*,*E*)-(1-((4-(Diethoxyphosphoryl)-4-fluorobut-3-en-1-yl)­amino)-1-oxo-3-phenylpropan-2-yl)­carbamate (**14a**)

According to the general procedure, **14a** was obtained using **11a** (1 mmol, 1.0 equiv., 325 mg). Flash column chromatography on silicagel *n*-hexane/ethyl acetate gradient (from 60:40 to 50:50) gave a white crystalline oil (421 mg, 89%): ^1^H NMR (400 MHz, Chloroform-*d*) δ *=* 7.35–7.15 (m, 5H, Ar-H), 6.27 (br.s, 1H, NH), 5.84 (dq, *J* = 39.1, 7.6 Hz, 1H, CHCFP), 5.16 (br.s, 1H, NH), 4.36–4.26 (m, 1H, CHNHBoc), 4.24–4.02 (m, 4H, 2 × OCH_2_CH_3_), 3.33–3.21 (m, 2H, CH_2_NH), 3.14–2.93 (m, 2H, CH_2_Ph), 2.40–2.27 (m, 2H, CH_2_), 1.39 (s, 9H, (CH_3_)_3_), 1.39–1.31 (m, 6H, 2 × OCH_2_CH_3_). ^13^C­{/^1^H} NMR (101 MHz, Chloroform-*d*) δ *=* 171.4 (s, CO), 155.5 (s, CO), 151.4 (dd, *J* = 276.0, 236.0 Hz, CFP), 136.7, 129.2, 128.6, 126.9 (4*s*, Ar-C), 122.2 (dd, *J* = 28.8, 5.6 Hz, CHCFP), 80.1 (s, C­(CH_3_)_3_), 63.13 (d, *J* = 5.4 Hz, OCH_2_CH_3_), 63.11 (d, *J* = 5.6 Hz, OCH_2_CH_3_), 55.8 (s, CHNHBoc), 38.7 (s, CH_2_Ph), 37.9 (s, CH_2_NH), 28.2 (s, (CH_3_)_3_), 24.4 (dd, *J* = 10.6, 4.6 Hz, CH_2_), 16.2 (d, *J* = 6.2 Hz, 2 × OCH_2_ CH_3_). ^19^F NMR (377 MHz, Chloroform-*d*) δ *=* −128.91 (dd, *J* = 102.3, 39.2 Hz, 1F). ^19^F­{/^1^H} NMR (377 MHz, Chloroform-*d*) δ *=* −128.91 (d, *J* = 102.5 Hz, 1F). ^31^P­{/^1^H} NMR (162 MHz, Chloroform-*d*) δ *=* 4.48 (d, *J* = 102.4 Hz, 1P). HRMS (ESI) calcd. for C_22_H_34_FN_2_O_6_PNa ([M + Na]^+^): 495.2036, found: 495.2044.

#### *Tert*-butyl ((2*S*)-1-(((*E*)-5-(Diethoxyphosphoryl)-5-fluoropent-4-en-2-yl)­amino)-1-oxo-3-phenylpropan-2-yl)­carbamate (*rac-* **14b**)

According to the general procedure, *rac-* **14b** was obtained using *rac-* **11b** (1 mmol, 1.0 equiv., 339 mg). Flash column chromatography on silicagel *n*-hexane/ethyl acetate gradient (from 60:40 to 50:50) gave a white crystalline oil (418 mg, 86%): ^1^H NMR (400 MHz, Chloroform-*d*) δ *=* 7.36–7.18 (m, 10H, 2 × Ar-H), 5.94–5.76 (m, 2H, 2 × CHCFP), 5.73 (d, *J* = 8.0 Hz, 1H, NH), 5.60 (d, *J* = 8.2 Hz, 1H, NH), 5.15 (br.s, 1H, NH), 5.04 (br.s, 1H, NH), 4.29–4.22 (m, 2H, CHNHBoc), 4.24–4.03 (m, 8H, 4 × OCH_2_CH_3_), 4.05–3.96 (m, 2H, 2 × CHNH), 3.13–2.94 (m, 4H, 2 × CH_2_Ph), 2.39–2.15 (m, 4H, 2 × CH_2_), 1.41 (s, 18H, 2­(CH_3_)_3_), 1.40–1.31 (m, 12H, 4 × OCH_2_CH_3_), 1.07 (d, *J* = 6.7 Hz, 3H, CH_3_), 0.97 (d, *J* = 6.7 Hz, 3H, CH_3_). ^13^C­{/^1^H} NMR (101 MHz, Chloroform-*d*) δ *=* 170.5 (s, 2 × CO), 155.4 (s, 2 × CO), 154.6–148.3 (m, 2 × CFP), 136.8, 136.7, 129.3, 129.3, 128.7, 128.7, 127.00, 126.97 (8*s*, Ar-C), 121.5 (dd, *J* = 28.9, 5.5 Hz, 2 × CHCFP), 80.2 (s, 2 × C­(CH_3_)_3_), 63.2–62.9 (m, 4 × OCH_2_CH_3_), 56.3–55.7 (m, 2 × CHNHBoc), 44.3 (s, CHN), 44.2 (s, CHN), 38.8 (s, CH_2_Ph), 38.5 (s, CH_2_Ph), 31.1–30.5 (m, 2 × CH_2_), 28.2 (s, 2­(CH_3_)_3_), 19.8 (s, 2 × CH_3_), 16.3 (s, 2 × OCH_2_ CH_3_), 16.2 (s, 2 × OCH_2_ CH_3_). ^19^F NMR (377 MHz, Chloroform-*d*) δ *=* −128.40 to −129.30 (m, 2F). ^19^F­{/^1^H} NMR (377 MHz, Chloroform-*d*) δ *=* −128.72 (d, *J* = 102.1 Hz, 1F), −128.92 (d, *J* = 103.0 Hz, 1F). ^31^P­{/^1^H} NMR (162 MHz, Chloroform-*d*) δ *=* 4.57 (d, *J* = 102.7 Hz, 1P), 4.52 (d, *J* = 102.5 Hz, 1P). HRMS (ESI) calcd. for C_23_H_36_FN_2_O_6_PNa ([M + Na]^+^): 509.2193, found: 509.2180.

#### *Tert*-butyl ((2*S*)-1-(((*E*)-1-(Diethoxyphosphoryl)-1-fluoro-6-methylhept-1-en-4-yl)­amino)-1-oxo-3-phenylpropan-2-yl)­carbamate (*rac-* **14c**)

According to the general procedure, *rac-* **14c** was obtained using *rac-* **11c** (1 mmol, 1.0 equiv., 381 mg). Flash column chromatography on silicagel *n*-hexane/ethyl acetate gradient (from 60:40 to 50:50) gave a white crystalline oil (444 mg, 84%): ^1^H NMR (400 MHz, Chloroform-*d*) δ *=* 7.32–7.01 (m, 10H, 2 × Ar-H), 6.16–5.97 (m, 2H, 2 × NH), 5.93–5.59 (m, 2H, 2 × CHCFP), 5.20 (br.s, 1H, NH), 5.14 (d, *J* = 8.3 Hz, 1H, NH), 4.27–4.16 (m, 2H, 2 × CHNHBoc), 4.13–4.04 (m, 8H, 4 × OCH_2_CH_3_), 4.03–3.92 (m, 2H, 2 × CHNH), 3.05–2.86 (m, 4H, 2 × CH_2_Ph), 2.40–2.23 (m, 2H, CH_2_), 2.24–2.13 (m, 2H, CH_2_), 1.55–1.37 (m, 2H, 2 × CH­(CH_3_)_2_), 1.32 (s, 18H, 2­(CH_3_)_3_), 1.32–1.24 (m, 12H, 4 × OCH_2_CH_3_), 1.25–1.04 (m, 4H, 2 × CH_2_CH­(CH_3_)_2_), 0.78 (d, *J* = 6.6 Hz, 6H, 2 × CH_3_), 0.76–0.71 (m, 6H, 2 × CH_3_). ^13^C­{/^1^H} NMR (101 MHz, Chloroform-*d*) δ *=* 170.9 (s, CO), 170.8 (s, CO), 155.3 (s, 2 × CO), 151.4 (dd, *J* = 275.2, 236.1 Hz, 2 × CFP), 136.7, 129.2, 128.4, 126.7 (4*s*, 2 × Ar-C), 121.8 (dd, *J* = 28.9, 5.4 Hz, 2 × CHCFP), 79.9 (s, 2 × C­(CH_3_)_3_), 63.2–62.9 (m, 4 × OCH_2_CH_3_), 55.9 (s, CHNHBoc), 55.7 (s, CHNHBoc), 46.24 (s, CHNH), 46.17 (s, CHNH), 42.9 (s, 2 × CH_2_CH­(CH_3_)_2_), 38.3 (s, CH_2_Ph), 38.1 (s, CH_2_Ph), 29.9–29.6 (m, 2 × CH_2_), 28.1 (s, 2­(CH_3_)_3_), 24.5 (s, CH­(CH_3_)_2_, 24.3 (s, CH­(CH_3_)_2_, 22.92 (s, CH_3_), 22.90 (s, CH_3_), 21.7 (s, 2 × CH_3_), 16.1 (s, 2 v OCH_2_ CH_3_), 16.0 (s, 2 × OCH_2_ CH_3_). ^19^F NMR (377 MHz, Chloroform-*d*) δ *=* −128.35 to −129.83 (m, 2F). ^19^F­{/^1^H} NMR (377 MHz, Chloroform-*d*) δ *=* −128.94 (d, *J* = 103.3 Hz, 1F), −129.17 (d, *J* = 104.1 Hz, 1F). ^31^P­{/^1^H} NMR (162 MHz, Chloroform-*d*) δ *=* 4.64 (d, *J* = 104.1 Hz, 1P), 4.55 (d, *J* = 103.2 Hz, 1P). HRMS (ESI) calcd. for C_26_H_42_FN_2_O_6_PNa ([M + Na]^+^): 551.2662, found: 551.2659.

#### *Tert*-butyl ((2*S*)-1-(((*E*)-4-(Diethoxyphosphoryl)-4-fluoro-1-phenylbut-3-en-1-yl)­amino)-1-oxo-3-phenylpropan-2-yl)­carbamate (*rac-* **14d**)

According to the general procedure, *rac-* **14d** was obtained using *rac-* **11d** (1 mmol, 1.0 equiv., 401 mg). Flash column chromatography on silicagel *n*-hexane/ethyl acetate gradient (from 60:40 to 50:50) gave a white crystalline oil (450 mg, 82%): ^1^H NMR (400 MHz, Chloroform-*d*) δ *=* 7.37–7.05 (m, 20H, 2 × Ar-H), 6.55–6.26 (m, 2H, 2 × NH), 5.90–5.58 (m, 2H, 2 × CHCFP), 5.18–4.88 (m, 4H, 2 × CHNH, 2 × NH), 4.44–4.20 (m, 2H, 2 × CHNHBoc), 4.16–3.79 (m, 8H, 4 × OCH_2_CH_3_), 3.17–2.92 (m, 4H, 2 × CH_2_Ph), 2.82–2.47 (m, 4H, 2 × CH_2_), 1.38 (s, 18H, (CH_3_)_3_), 1.33–1.20 (m, 12H, 4 × OCH_2_CH_3_). ^13^C­{/^1^H} NMR (101 MHz, Chloroform-*d*) δ *=* 170.6 (s, CO), 170.4 (s, CO), 155.4 (s, 2 × CO), 151.53 (dd, *J* = 276.4, 234.2 Hz, CFP), 151.46 (dd, *J* = 276.9, 233.9 Hz, CFP), 139.9, 139.8, 136.7, 136.5, 129.3, 129.1, 128.73, 128.72, 128.68, 128.6, 127.79, 127.76, 126.9, 126.8, 126.52, 126.51 (16*s*, 2 × Ar-C), 121.7–120.8 (m, 2 × CHCFP), 80.2 (s, 2 × C­(CH_3_)_3_), 63.1–62.8 (m, 4 × OCH_2_CH_3_), 55.9 (s, CHNHBoc), 55.7 (s, CHNHBoc), 52.4 (s, CHNH), 52.1 (s, CHNH), 38.3 (s, 2 × CH_2_Ph), 30.3 (s, CH_2_), 30.2 (s, CH_2_), 28.2 (s, 2­(CH_3_)_3_), 16.2–15.9 (m, 4 × OCH_2_ CH_3_). ^19^F NMR (377 MHz, Chloroform-*d*) δ *=* −127.60 to −128.39 (m, 2F). ^19^F­{/^1^H} NMR (377 MHz, Chloroform-*d*) δ *=* −127.89 (d, *J* = 102.0 Hz, 1F), −128.01 (d, *J* = 102.4 Hz, 1F). ^31^P­{/^1^H} NMR (162 MHz, Chloroform-*d*) δ *=* 4.39 (d, *J* = 102.4 Hz, 1P), 4.35 (d, *J* = 101.8 Hz, 1P). HRMS (ESI) calcd. for C_28_H_38_FN_2_O_6_PNa ([M + Na]^+^): 571.2349, found: 571.2336.

#### *Tert*-butyl ((2*S*)-1-(((*E*)-5-(Diethoxyphosphoryl)-5-fluoro-1-phenylpent-4-en-2-yl)­amino)-1-oxo-3-phenylpropan-2-yl)­carbamate (*rac-* **14e**)

According to the general procedure, *rac-* **14e** was obtained using *rac-* **11e** (1 mmol, 1.0 equiv., 415 mg). Flash column chromatography on silicagel *n*-hexane/ethyl acetate gradient (from 60:40 to 50:50) gave a white crystalline oil (495 mg, 88%): ^1^H NMR (400 MHz, Chloroform-*d*) δ *=* 7.39–6.93 (m, 20H, 2 × Ar-H), 5.96–5.80 (m, 2H, 2 × CHCFP), 5.80–5.70 (m, 2H, 2 × NH), 5.18–4.76 (m, 2H, 2 × NH), 4.29–4.05 (m, 12H, 4 × OCH_2_CH_3_, 2 × CHNHBoc, 2 × CHNH), 3.05–2.89 (m, 4H, 2 × CH_2_Ph), 2.74–2.57 (m, 4H, 2 × CH_2_Ph), 2.49–2.18 (m, 4H, 2 × CH_2_), 1.39 (s, 18H, (CH_3_)_3_), 1.36 (t, *J* = 7.1 Hz, 12H, 4 × OCH_2_CH_3_). ^13^C­{/^1^H} NMR (101 MHz, Chloroform-*d*) δ *=* 170.83 (s, CO), 170.78 (s, CO), 155.3 (s, 2 × CO), 151.73 (dd, *J* = 276.1, 236.0 Hz, CFP), 151.71 (dd, *J* = 275.5, 236.2 Hz, CFP), 136.85, 136.79, 136.7, 136.6, 129.20, 129.17, 129.16, 129.1, 128.6, 128.51, 128.48, 126.84, 126.79, 126.7, 126.6 (15*s*, 2 × Ar-C), 121.53 (dd, *J* = 29.0, 5.5 Hz, CHCFP), 121.50 (dd, *J* = 29.0, 5.5 Hz, CHCFP), 80.0 (s, 2 × C­(CH_3_)_3_), 63.2–63.0 (m, 4 × OCH_2_CH_3_), 55.8 (s, 2 × CHNHBoc), 49.2 (s, CHNH), 49.1 (s, CHNH), 39.82 (s, CH_2_Ph), 39.77 (s, CH_2_Ph), 38.4 (s, CH_2_Ph), 38.3 (s, CH_2_Ph), 28.5–28.2 (m, 2 × CH_2_), 28.1 (s, 2­(CH_3_)_3_), 16.2 (s, 2 × OCH_2_ CH_3_), 16.1 (s, 2 × OCH_2_ CH_3_). ^19^F NMR (377 MHz, Chloroform-*d*) δ *=* −128.10 (dd, *J* = 102.7, 39.2 Hz, 1F), −128.30 (dd, *J* = 103.5, 39.2 Hz, 1F). ^19^F­{/^1^H} NMR (377 MHz, Chloroform-*d*) δ *=* −128.10 (d, *J* = 102.7 Hz, 1F), −128.30 (d, *J* = 103.3 Hz, 1F). ^31^P­{/^1^H} NMR (162 MHz, Chloroform-*d*) δ *=* 4.41 (d, *J* = 114.7 Hz, 1P), 4.40 (d, *J* = 91.6 Hz, 1P). HRMS (ESI) calcd. for C_29_H_40_FN_2_O_6_PNa ([M + Na]^+^): 585.2506, found: 585.2502.

### Synthesis of **5**

Compounds **14** were treated with 4 M HCl in MeOH (1 mL per 0.2 mM of each compound) at room temperature for 15 min. After the reaction, the solvents were removed under reduced pressure, and the resulting residues were washed with anhydrous Et_2_O. The ether layers were decanted, and the solids were dried under vacuum. Before the biological evaluation, the compounds were subjected to lyophilization.

#### (*S*,*E*)-1-((4-(Diethoxyphosphoryl)-4-fluorobut-3-en-1-yl)­amino)-1-oxo-3-phenylpropan-2-aminium chloride (**5a**)

According to the general procedure, **5a** was obtained using *rac-* **14a** (0.2 mmol, 1.0 equiv., 94 mg). White solid (81 mg, 99%): ^1^H NMR (400 MHz, Methanol-*d*_4_) δ *=* 7.49–7.18 (m, 5H, Ar-H), 5.89 (dq, *J* = 39.3, 7.5 Hz, 1H, CHCFP), 4.23–4.10 (m, 4H, 2 × OCH_2_CH_3_), 4.04 (dd, *J* = 7.9, 6.9 Hz, 1H, CHNH_3_), 3.36 (dt, *J* = 13.5, 6.7 Hz, 1H, CH_a_H_b_NH), 3.28–3.14 (m, 2H, CH_a_ H_b_NH, CH_a_H_b_Ph), 3.07 (dd, *J* = 13.9, 7.9 Hz, 1H, CH_a_ H_b_Ph), 2.47–2.30 (m, 2H, CH_2_), 1.42–1.24 (m, 6H, 2 × OCH_2_CH_3_). ^13^C­{/^1^H} NMR (101 MHz, Methanol-*d*_4_) δ *=* 170.6 (s, CO), 153.1 (dd, *J* = 273.0, 240.0 Hz, CFP), 136.5, 131.4, 131.0, 129.8 (4*s*, Ar-C), 125.2 (dd, *J* = 29.0, 5.5 Hz, CHCFP), 65.8 (d, *J* = 2.0 Hz, OCH_2_CH_3_), 65.7 (d, *J* = 2.0 Hz, OCH_2_CH_3_), 56.7 (s, CHNH_3_), 39.8 (s, CH_2_NH), 39.7 (s, CH_2_Ph), 26.1 (dd, *J* = 10.5, 5.0 Hz, CH_2_), 17.45 (s, OCH_2_ CH_3_), 17.39 (s, OCH_2_ CH_3_). ^19^F NMR (377 MHz, Methanol-*d*_4_) δ *=* −130.90 (dd, *J* = 106.4, 39.3 Hz, 2F). ^19^F­{/^1^H} NMR (377 MHz, Methanol-*d*_4_) δ *=* −130.90 (d, *J* = 106.2 Hz, 2F). ^31^P­{/^1^H} NMR (162 MHz, Methanol-*d*_4_) δ *=* 4.64 (d, *J* = 106.4 Hz, 2P). HRMS (ESI) calcd. for C_17_H_27_FN_2_O_4_P ([M+]^+^): 373.1692, found: 373.1691.

#### (2*S*)-1-(((*E*)-5-(Diethoxyphosphoryl)-5-fluoropent-4-en-2-yl)­amino)-1-oxo-3-phenylpropan-2-aminium chloride (*rac-* **5b**)

According to the general procedure, *rac-* **5b** was obtained using *rac-* **14a** (0.2 mmol, 1.0 equiv., 97 mg). White solid (84 mg, 99%): ^1^H NMR (400 MHz, Methanol-*d*_4_) δ *=* 7.43–7.25 (m, 10H, 2 × Ar-H), 6.04–5.70 (m, 2H, 2 × CHCFP), 4.23–4.08 (m, 8H, 4 × OCH_2_CH_3_), 4.09–3.87 (m, 4H, 2 × CHNH, 2 × CHNH_3_), 3.19 (dd, *J* = 13.9, 6.6 Hz, 1H, CH_a_H_b_Ph), 3.12 (dd, *J* = 7.5, 2.9 Hz, 2H, CH_2_Ph), 3.04 (dd, *J* = 13.9, 8.4 Hz, 1H, CH_a_ H_b_Ph), 2.52–2.25 (m, 4H, 2 × CH_2_), 1.40–1.26 (m, 12H, 4 × OCH_2_CH_3_), 1.18 (d, *J* = 6.8 Hz, 3H, CH_3_), 0.97 (d, *J* = 6.8 Hz, 3H, CH_3_). ^13^C­{/^1^H} NMR (101 MHz, Methanol-*d*_4_) δ *=* 169.8 (s, CO), 169.7 (s, CO), 156.3–150.1 (m, 2 × CFP), 136.6, 136.5, 131.44, 131.39, 131.0, 130.9, 129.8, 129.7 (8*s*, Ar-C), 125.4–123.8 (m, 2 × CHCFP), 66.1–65.4 (m, 4 × OCH_2_CH_3_), 56.7 (s, 2 × CHNH_3_), 46.8 (s, CHNH), 46.6 (s, CHNH), 39.9 (s, CH_2_Ph), 39.7 (s, CH_2_Ph), 32.6–32.4 (m, 2 × CH_2_), 20.87 (s, CH_3_), 20.83 (s, CH_3_), 17.44 (s, 2 × OCH_2_ CH_3_), 17.39 (s, 2 × OCH_2_ CH_3_). ^19^F NMR (377 MHz, Methanol-*d*_4_) δ *=* −130.72 (dd, *J* = 106.5, 39.0 Hz, 1F), −130.76 (dd, *J* = 106.2, 39.2 Hz, 1F). ^19^F­{/^1^H} NMR (377 MHz, Methanol-*d*_4_) δ *=* −130.72 (d, *J* = 106.3 Hz, 1F), −130.76 (d, *J* = 106.3 Hz, 1F). ^31^P­{/^1^H} NMR (162 MHz, Methanol-*d*_4_) δ *=* 4.87 (d, *J* = 106.0 Hz, 1F), 4.56 (d, *J* = 106.4 Hz, 1F). HRMS (ESI) calcd. for C_18_H_29_FN_2_O_4_P ([M+]^+^): 387.1849, found: 387.1852.

#### (2*S*)-1-(((*E*)-1-(Diethoxyphosphoryl)-1-fluoro-6-methylhept-1-en-4-yl)­amino)-1-oxo-3-phenylpropan-2-aminium chloride (*rac-* **5c**)

According to the general procedure, *rac-* **5c** was obtained using *rac-* **14c** (0.2 mmol, 1.0 equiv., 106 mg). White solid (92 mg, 99%): ^1^H NMR (400 MHz, Methanol-*d*_4_) δ *=* 7.43–7.24 (m, 10H, 2 × Ar-H), 6.04–5.70 (m, 2H, 2 × CHCFP), 4.24–4.02 (m, 11H, 4 × OCH_2_CH_3_, 2 × CHNH_3_, CHNH), 3.99–3.85 (m, 1H, CHNH), 3.27–2.95 (m, 4H, 2 × CH_2_Ph), 2.51–2.34 (m, 2H, CH_2_), 2.34–2.27 (m, 2H, CH_2_), 1.76–1.58 (m, 1H, CH­(CH_3_)_2_), 1.53–1.33 (m, 2H,CH_2_ CH­(CH_3_)_2_), 1.39–1.19 (m, 13H, 4 × OCH_2_CH_3_, CH_a_H_b_CH­(CH_3_)_2_), 1.22–1.10 (m, 2H, CH­(CH_3_)_2_, CH_a_ H_b_CH­(CH_3_)_2_), 0.97–0.87 (m, 6H, 2 × CH_3_), 0.83–0.74 (m, 6H, 2 × CH_3_). ^13^C­{/^1^H} NMR (101 MHz, Methanol-*d*_4_) δ *=* 170.1 (s, CO), 170.0 (s, CO), 156.3–150.0 (m, 2 × CFP), 136.5, 136.42, 131.40, 131.37, 131.05, 130.96, 129.8, 129.6 (8*s*, 2 × Ar-C), 125.2–124.1 (m, 2 × CHCFP), 66.0–65.6 (m, 4 × OCH_2_CH_3_), 56.8 (s, CHNH_3_), 56.6 (s, CHNH_3_), 49.0 (s, CHNH), 48.7 (s, CHNH), 45.0 (s, CH_2_CH­(CH_3_)_2_), 44.8 (s, CH_2_CH­(CH_3_)_2_), 40.0 (s, CH_2_Ph), 39.6 (s, CH_2_Ph), 32.3–31.1 (m, 2 × CH_2_), 26.6 (s, CH­(CH_3_)_2_, 26.3 (s, CH­(CH_3_)_2_, 24.4 (s, CH_3_), 24.3 (s, CH_3_), 23.1 (s, CH_3_), 22.9 (s, CH_3_), 17.5 (s, 2 × OCH_2_ CH_3_), 17.4 (s, 2 × OCH_2_ CH_3_). ^19^F NMR (377 MHz, Methanol-*d*_4_) δ *=* −130.83 (dd, *J* = 106.8, 38.8 Hz, 1F), −130.87 (dd, *J* = 106.6, 39.1 Hz, 1F). ^19^F­{/^1^H} NMR (377 MHz, Methanol-*d*_4_) δ *=* −130.82 (d, *J* = 106.8 Hz, 1F), −130.86 (d, *J* = 106.8 Hz, 1F). ^31^P­{/^1^H} NMR (162 MHz, Methanol-*d*_4_) δ *=* 4.98 (d, *J* = 107.3 Hz, 1P), 4.56 (d, *J* = 106.3 Hz, 1P). HRMS (ESI) calcd. for C_21_H_35_FN_2_O_4_P ([M+]^+^): 429.2318, found: 429.2315.

#### (2*S*)-1-(((*E*)-4-(Diethoxyphosphoryl)-4-fluoro-1-phenylbut-3-en-1-yl)­amino)-1-oxo-3-phenylpropan-2-aminium chloride (*rac-* **5d**)

According to the general procedure, *rac-* **5d** was obtained using *rac-* **14d** (0.2 mmol, 1.0 equiv., 110 mg). White solid (96 mg, 99%): ^1^H NMR (400 MHz, Methanol-*d*_4_) δ *=* 7.46–7.06 (m, 20H, 2 × Ar-H), 5.97–5.68 (m, 2H, 2 × CHCFP), 5.06–4.96 (m, 2H, 2 × CHNH), 4.17–4.03 (m, 2H, 2 × CHNH_3_), 4.07–3.87 (m, 8H, 4 × OCH_2_CH_3_), 3.24 (dd, *J* = 13.9, 6.8 Hz, 1H, CH_a_H_b_Ph), 3.17–3.06 (m, 2H, CH_2_Ph), 3.03 (dd, *J* = 13.8, 6.9 Hz, 1H, CH_a_ H_b_Ph), 2.88–2.71 (m, 2H, CH_2_), 2.68–2.58 (m, 2H, CH_2_), 1.33–1.19 (m, 12H, 4 × OCH_2_CH_3_). ^13^C­{/^1^H} NMR (101 MHz, Methanol-*d*_4_) δ *=* 169.8 (s, CO), 169.6 (s, CO), 155.9–150.0 (m, 2 × CFP) 142.5, 142.3, 136.5, 136.0, 131.5, 131.3, 131.0, 130.8, 130.7, 130.6, 129.81, 129.79, 129.7, 129.5, 128.9, 128.8 (16*s*, 2 × Ar-C), 125.0–123.9 (m, 2 × CHCFP), 65.8–65.5 (m, 4 × OCH_2_CH_3_), 56.6 (s, CHNH_3_), 56.5 (s, CHNH_3_), 55.1–54.4 (m, 2 × CHNH), 39.9 (s, CH_2_Ph), 39.4 (s, CH_2_Ph), 32.1 (dd, *J* = 10.5, 4.4 Hz, 2 × CH_2_), 17.5–17.2 (m, 4 × OCH_2_ CH_3_). ^19^F NMR (377 MHz, Methanol-*d*_4_) δ *=* −129.56 to −130.21 (m, 2F). ^19^F­{/^1^H} NMR (377 MHz, Methanol-*d*_4_) δ *=* −129.83 (d, *J* = 105.4 Hz, 1F), −129.91 (d, *J* = 105.3 Hz, 1F). ^31^P­{/^1^H} NMR (162 MHz, Methanol-*d*_4_) δ *=* 4.68 (d, *J* = 105.1 Hz, 1P), 4.46 (d, *J* = 105.3 Hz, 1P). HRMS (ESI) calcd. for C_23_H_31_FN_2_O_4_P ([M+]^+^): 449.2005, found: 449.2001.

#### (2*S*)-1-(((*E*)-5-(Diethoxyphosphoryl)-5-fluoro-1-phenylpent-4-en-2-yl)­amino)-1-oxo-3-phenylpropan-2-aminium chloride (*rac-* **5e**)

According to the general procedure, *rac-* **5e** was obtained using *rac-* **14e** (0.2 mmol, 1.0 equiv., 113 mg). White solid (99 mg, 99%): ^1^H NMR (400 MHz, Methanol-*d*_4_) δ *=* 7.58–6.94 (m, 20H, 2 × Ar-H), 6.17–5.62 (m, 2H, 2 × CHCFP), 4.38–4.06 (m, 10H, 4 × OCH_2_CH_3_, 2 × CHNH), 4.06–3.93 (m, 2H, 2 × CHNH_3_), 3.21 (dd, *J* = 14.1, 5.4 Hz, 1H, CH_a_H_b_Ph), 3.05–2.61 (m, 7H, CH_a_ H_b_Ph, 3 x CH_2_Ph), 2.64–2.33 (m, 4H, 2 × CH_2_), 1.38–1.23 (m, 12H, 4 × OCH_2_CH_3_). ^13^C­{/^1^H} NMR (101 MHz, Methanol-*d*_4_) δ *=* 170.2 (s, CO), 170.1 (s, CO), 156.2–149.2 (m, 2 × CFP), 140.1, 139.9, 136.5, 136.1, 131.4, 131.2, 131.11, 131.10, 130.9, 130.5, 129.8, 129.7, 128.7, 128.6, (14*s*, 2 × Ar-C) 125.1–124.1 (m, 2 × CHCFP), 66.0–65.7 (m, 4 × OCH_2_CH_3_), 56.6 (s, CHNH_3_), 56.4 (s, CHNH_3_), 52.6 (s, CHNH), 52.4 (s, CHNH), 42.2 (s, CH_2_Ph), 40.0 (s, 2 × CH_2_Ph), 39.5 (s, CH_2_Ph), 31.3 (dd, *J* = 10.6, 4.2 Hz, CH_2_), 30.7 (dd, *J* = 10.3, 4.6 Hz. CH_2_), 17.45 (d, *J* = 1.8 Hz, 2 × OCH_2_ CH_3_), 17.39 (d, *J* = 1.8 Hz, 2 × OCH_2_ CH_3_). ^19^F NMR (377 MHz, Methanol-*d*_4_) δ *=* −130.00 to −130.82 (m, 2F). ^19^F­{/^1^H} NMR (377 MHz, Methanol-*d*_4_) δ *=* −130.30 (d, *J* = 106.2 Hz, 1F), −130.44 (d, *J* = 106.5 Hz, 1F). ^31^P­{/^1^H} NMR (162 MHz, Methanol-*d*_4_) δ *=* 4.91 (d, *J* = 106.3 Hz, 1P), 4.45 (d, *J* = 106.4 Hz, 1P). HRMS (ESI) calcd. for C_24_H_33_FN_2_O_4_P ([M+]^+^): 463.2162, found: 463.2170.

### Enzymatic Studies

Cathepsin C was purchased from Sigma-Aldrich. Gly-l-Phe-pNA was purchased from Sigma-Aldrich. The remaining reagents were purchased pure for analytical testing.

Cathepsin C was activated for 0.5 h in a water bath at 37 °C in 1% NaCl solution containing 1 mM EDTA-Na_2_ and 5 mM 2-mercaptoethanol. Enzymatic reactions were carried out at 37 °C for 10 min in 0.1 M acetate buffer, pH 5, containing 30 mM NaCl, 1 mM EDTA-Na_2_, and 1 mM DTT (all final concentrations). The progress of the reaction was monitored spectrophotometrically (Jasco V-730) at 405 nm using glycine-l-phenylalanine p-nitroanilide (Gly-l-Phe-pNA) as the substrate. The enzyme solution (cathepsin C, 0.021 mg/mL) was preincubated with a potential inhibitor solution (the concentration of the compound depends on the inhibitory potency) for 0.5 h at 37 °C. Next, a substrate solution in 100 mM acetate buffer, pH 5, containing 1 mM EDTA-Na_2_, 1 mM DTT, and 30 mM NaCl was added to the reaction mixture (substrate concentration: 3.0 to 0.5 mM—final concentration). Kinetic constants were calculated using a computer program kindly provided by Dr. Eng. J. Hurek (University of Opole). Weighted regression was used for linearization calculations. The program created by Dr. Eng. J. Hurek is based on a generalized version of the Michaelis equation for inhibition type (1) described in the paper.^33^

1 $$V_{0}=\frac{V_{\max}\times \left[S\right]}{K_{M}\times \left(1+\frac{\left[I\right]}{K_{\mathrm{ic}}}\right)+\left[S\right]\times \left(1+\frac{\left[I\right]}{K_{\mathrm{iu}}}\right)}$$

where: V_max_—limiting rate, K_M_—Michaelis constant, K_ic_—competitive inhibition constant, K_iu_—uncompetitive inhibition constant, K_i_—inhibition constant, [I]—inhibitor concentration, [S]—substrate concentration.

The inhibition type is determined based on the relationship between the inhibition constants K_ic_ and K_iu_. In eq 1, in the case of competitive inhibition the term $\frac{\left[I\right]}{K_{iu}}$ is omitted from the calculations. If $K_{\mathrm{iu}}\gg K_{\mathrm{ic}}$ we are dealing with competitive inhibition we are dealing with competitive inhibition. The inhibition constants were determined by linearizing eq[Disp-formula eq1] to the form $\frac{1}{V_{0}}=f\left(\frac{1}{\left[S\right]}\right)$ (Lineweaver–Burk plot) or form $\frac{1}{V_{0}}=f\left(\left[I\right]\right)$ (Dixon plot).

### Molecular Docking

The molecular docking process began with converting the SMILES^34^ representation of a dipeptide ligand into a 3D structure using RDKit^35^ and OpenBabel (Pybel, version 3.1.0).^36,37^ The 1K3B protein domain (PDB ID)^25,26^ was protonated at pH 5.0 using the H++ web application^38–41^ and converted to the PDBQT format required by AutoDock Vina.^42^ The docking search space, defined in Ångstroms, was centered at coordinates (x, y, z) = (30.2, 25.3, 20.7) with a box size of (x, y, z) = (46.0, 40.0, 36.0). AutoDock Vina was run with an exhaustiveness of 32. Visualizations were prepared using Chimera 1.16 ^28^ and the Proteins Plus web application.^27,43^

## Supplementary Material





## Data Availability

The data underlying this study are available in the published article and its Supporting Information.

## Notes

Computational data (ZIP)
Copies of the NMR spectra and fluorine interaction analysis (PDF)
