# Supplementary material for: Design, Synthesis, and Biological Activity of (E)‑α-Fluorovinylphosphonate-Based Reversible Cathepsin C Inhibitors
Source: J Org Chem. 2026 Feb 13;91(8):3218–31. doi: 10.1021/acs.joc.5c02923 (PMC13298919; doi:10.1021/acs.joc.5c02923)
Supplement: Supplementary file 2 [file jo5c02923_si_002.pdf]

Design, Synthesis, and Biological Activity of (E)-α-Fluorovinylphosphonate-Based Reversible Cathepsin C Inhibitors

Marcin Kaźmierczak[a,b]*, Monika Bilska-Markowska[a], Katarzyna Wiśniewska[a], Małgorzata Pawełczak[c], Damian Nowak[a], Marcin Hoffmann[a]

[a]*Faculty of Chemistry, Adam Mickiewicz University in Poznań, Uniwersytetu Poznańskiego 8, 61-614 Poznań, Poland*

[b]*Center for Advanced Technologies, Adam Mickiewicz University in Poznań, Uniwersytetu Poznańskiego 10, 61-614 Poznań, Poland*

[c]*Institute of Chemistry, Opole University, 45-052 Opole, Poland*

* Email: marcin.kazmierczak@amu.edu.pl

Table of Contents

1. NMR Spectroscopy DATA……………………………………………………………S2
2. Fluorine Interaction Analysis…………………………………………………………...S95

1.NMR Spectroscopy DATA.

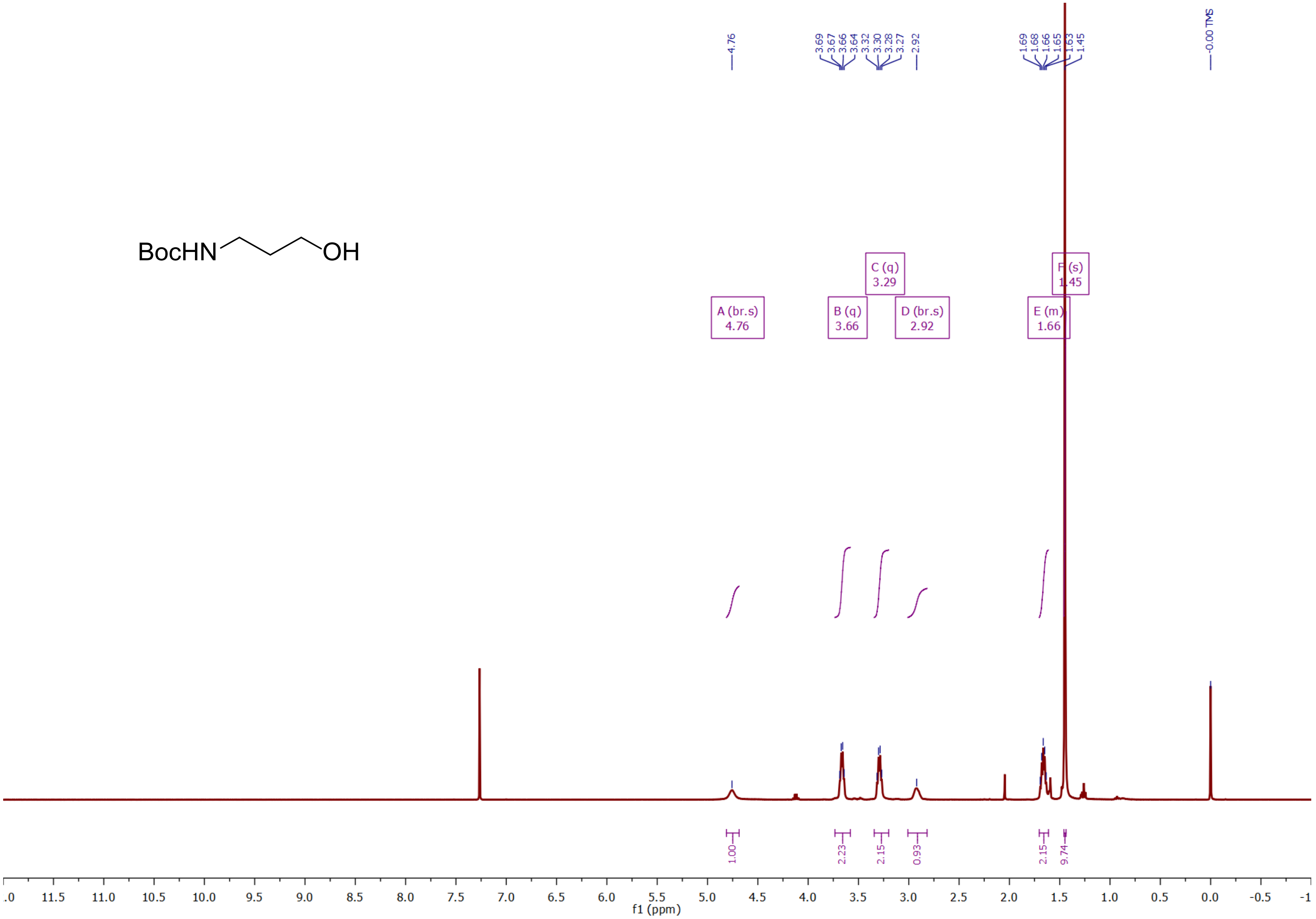

^{1}H NMR (400 MHz, Chloroform-*d*) of ***rac*-8a**.

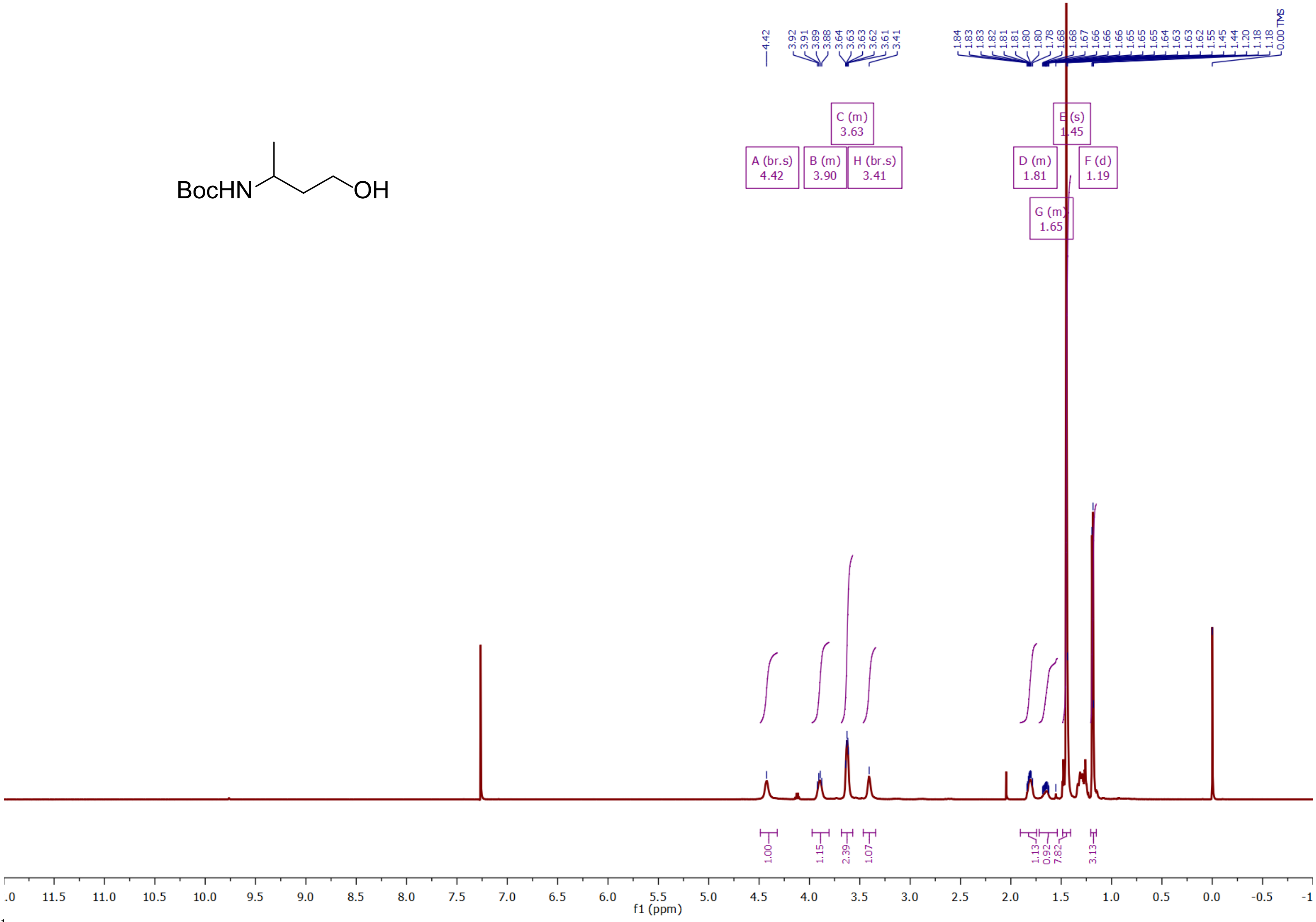

^{1}H NMR (600 MHz, Chloroform-d) of ***rac*-8b**.

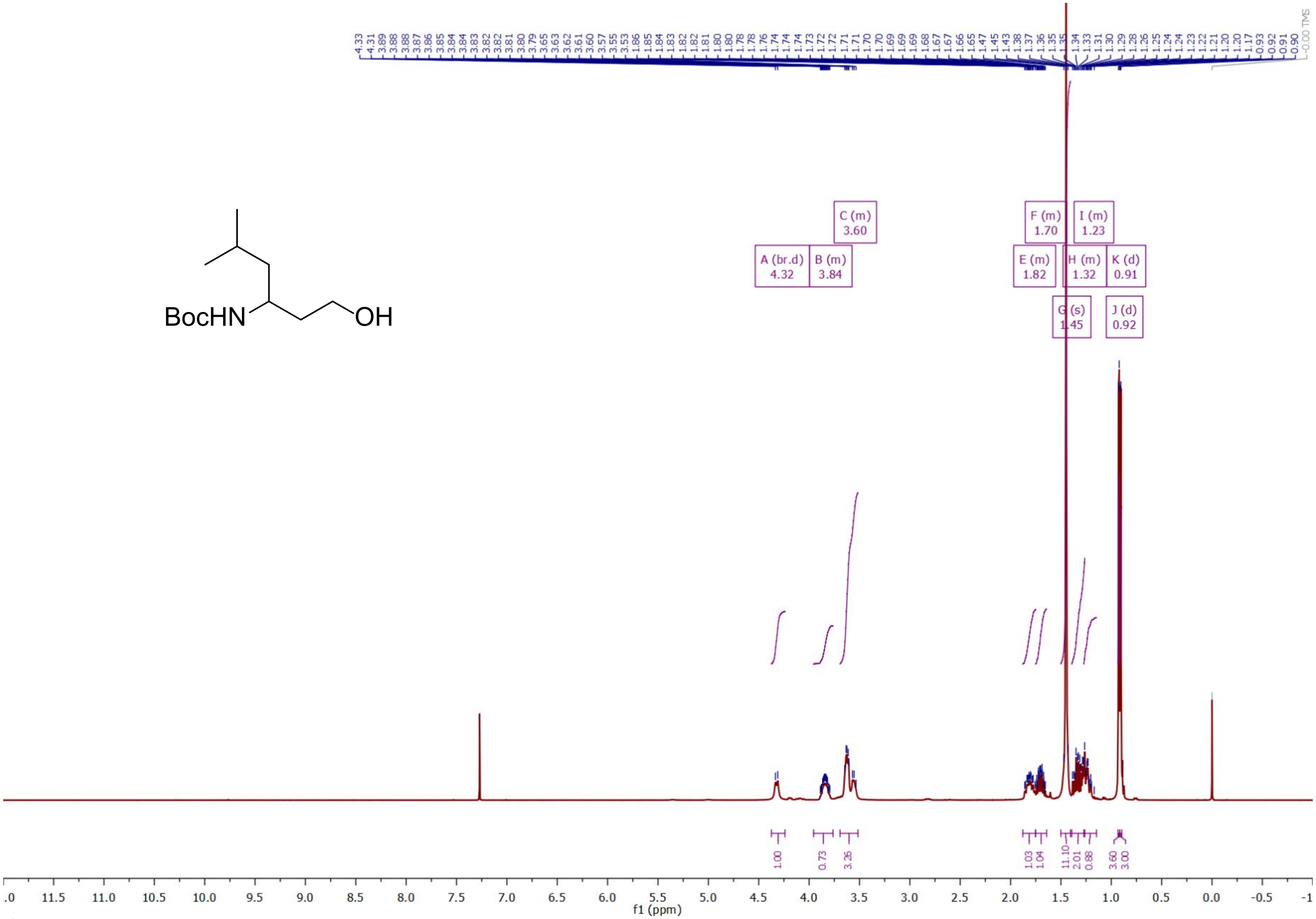

^{1}H NMR (400 MHz, Chloroform-*d*) of ***rac*-8**c.

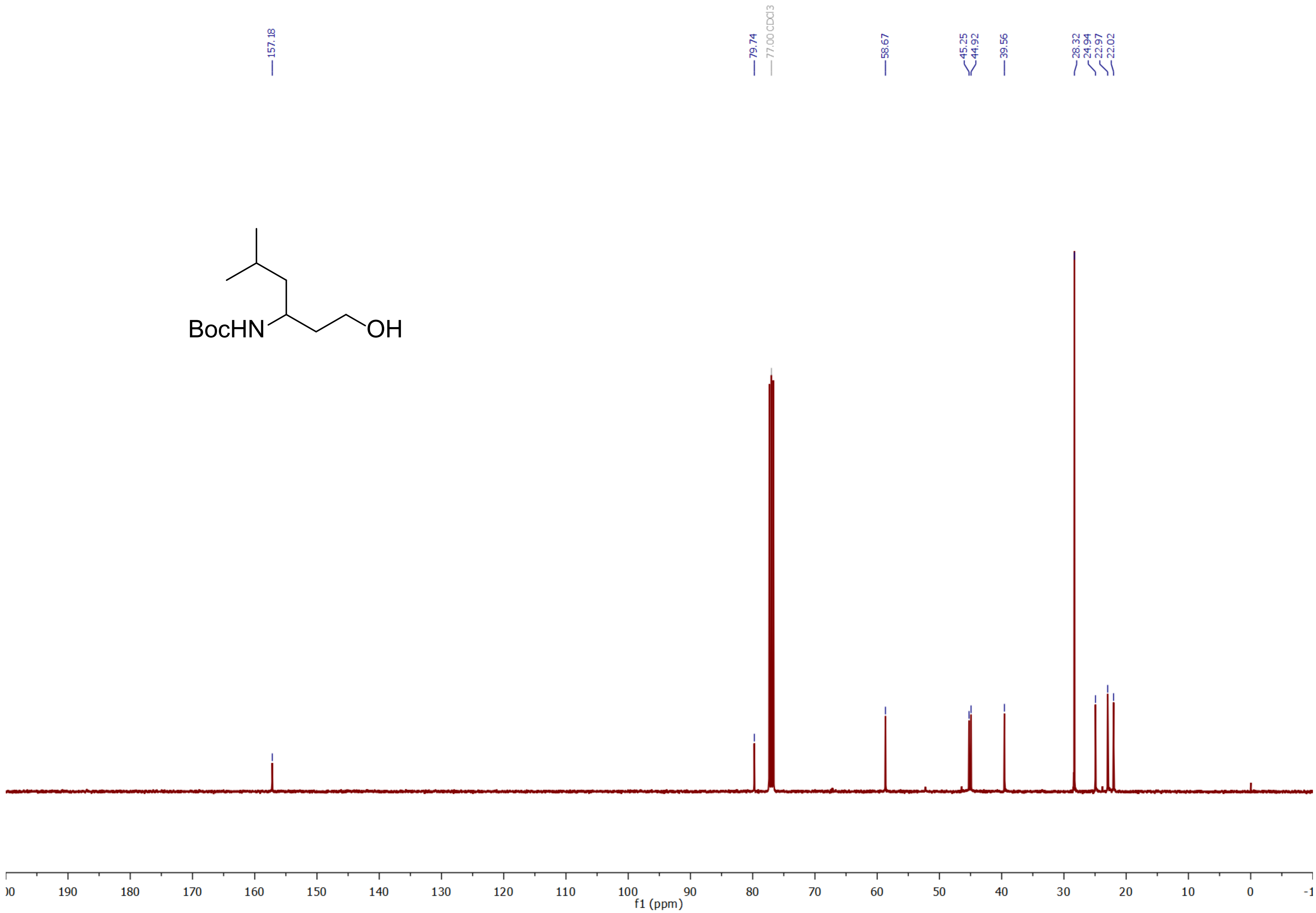

$^{13}C\{/^{1}H\}$ NMR (101 MHz, Chloroform-*d*) of ***rac*-8c**.

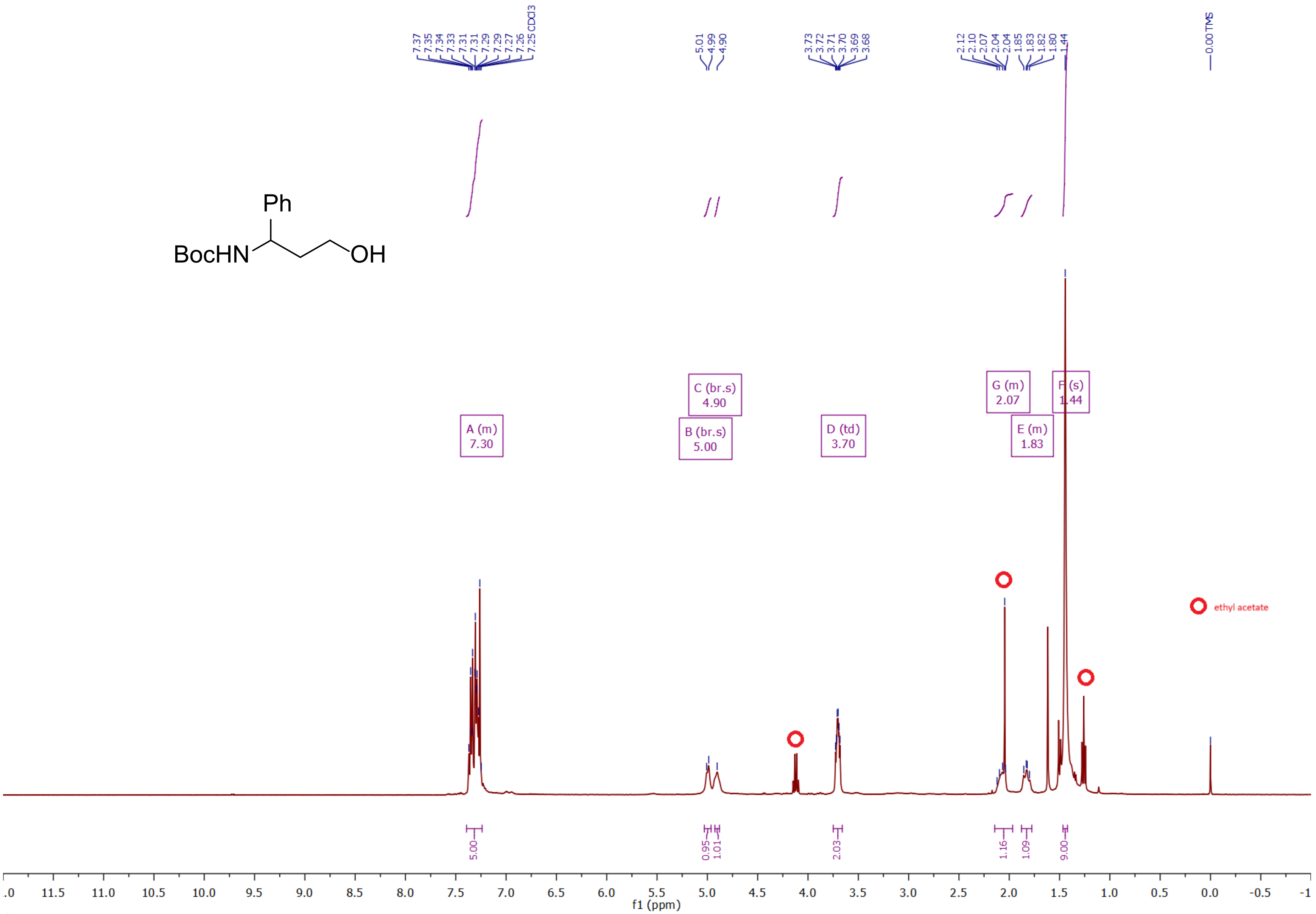

^{1}H NMR (400 MHz, Chloroform-*d*) of ***rac*-8d**.

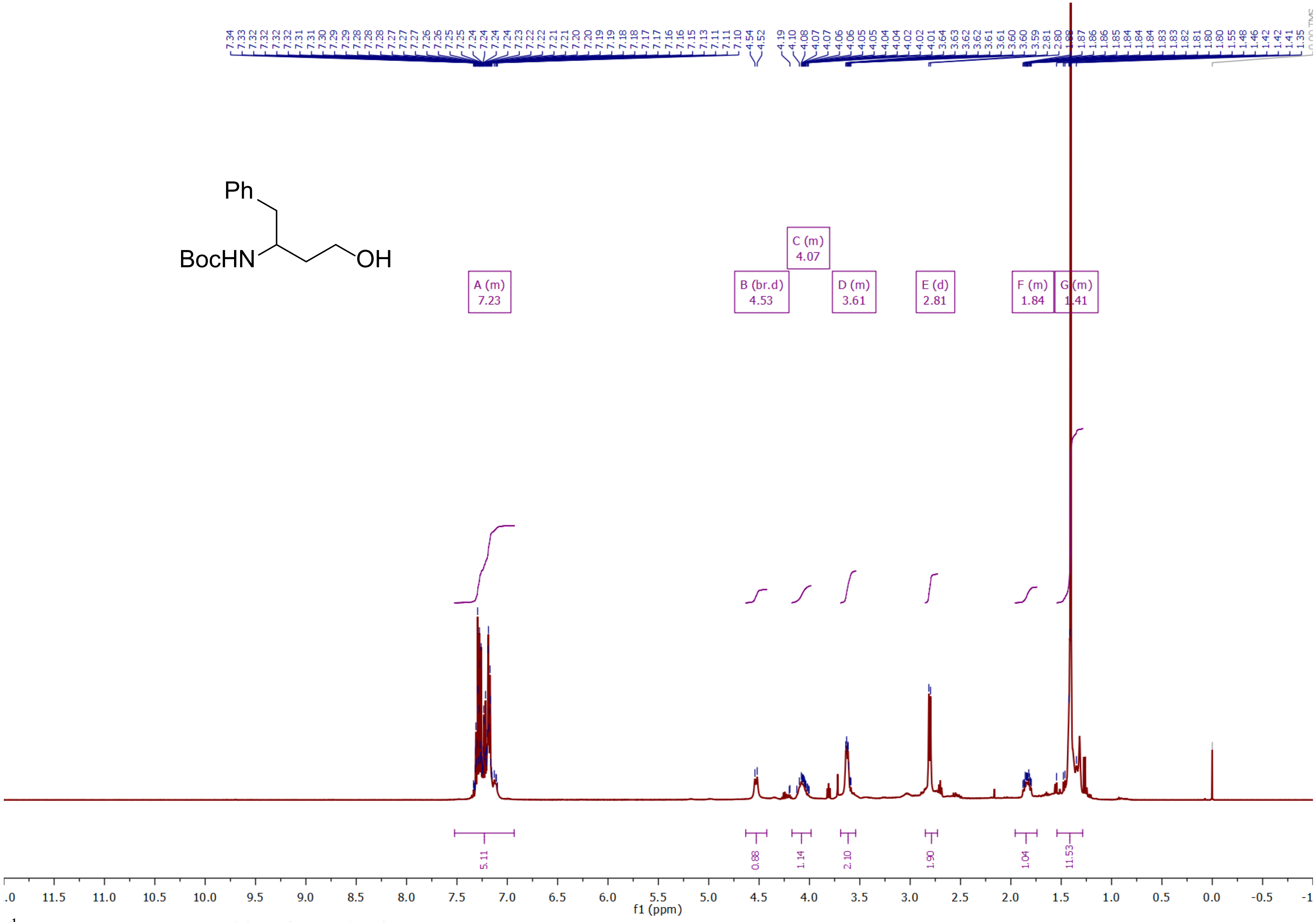

^{1}H NMR (400 MHz, Chloroform-*d*) of ***rac*-8e**.

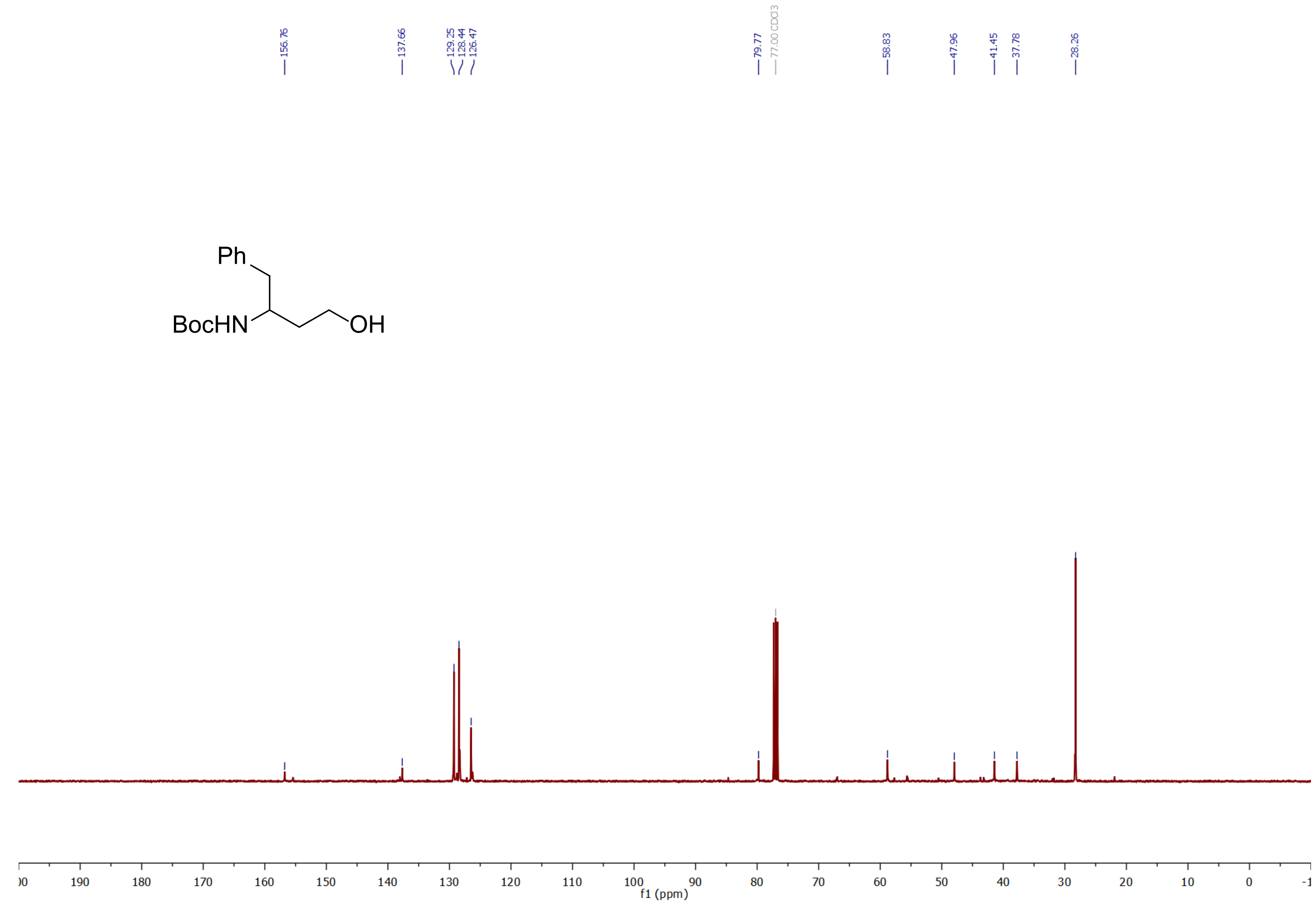

$^{13}C\{/^{1}H\}$ NMR (101 MHz, Chloroform-*d*) of ***rac*-8e**.

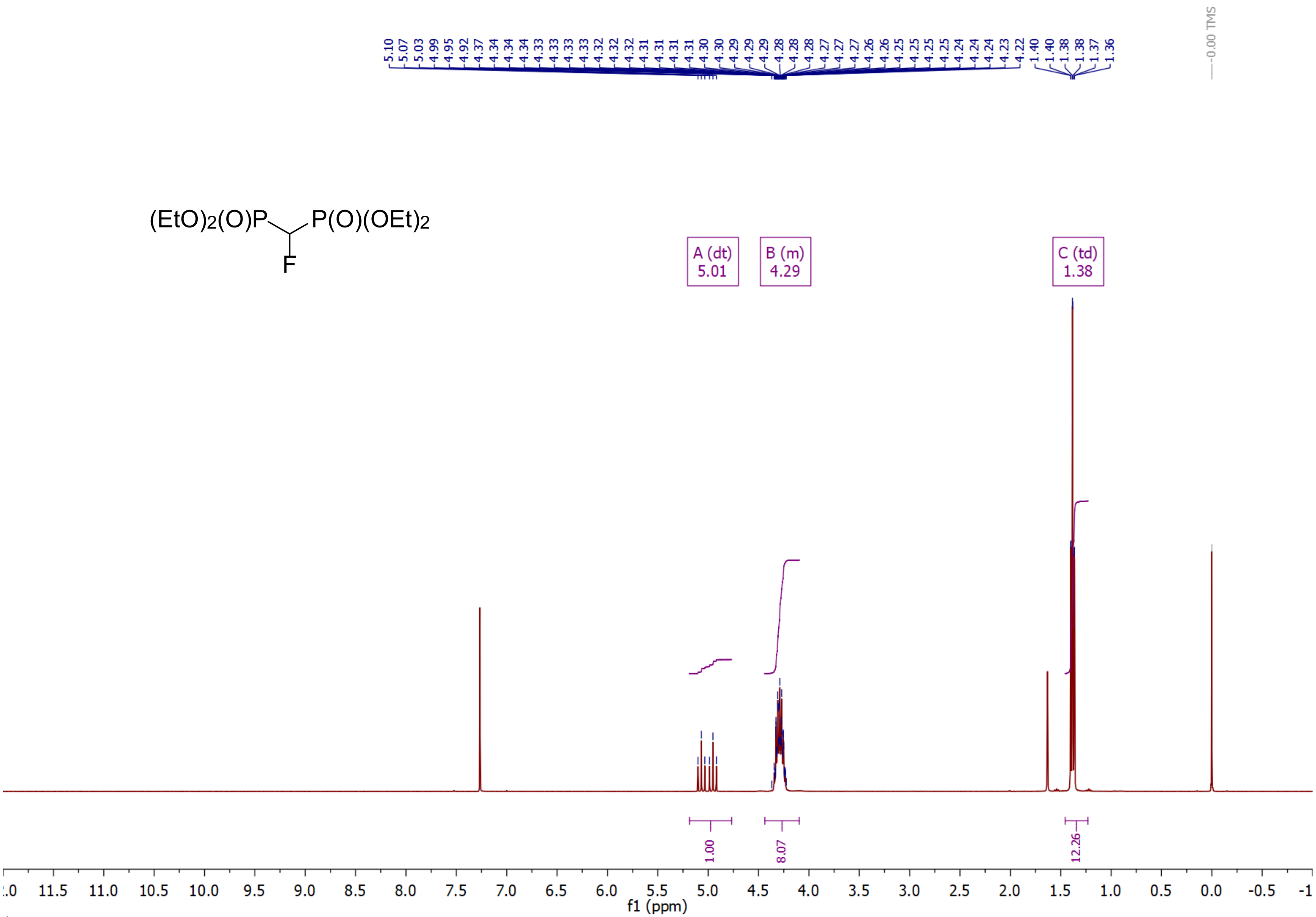

^{1}H NMR (400 MHz, Chloroform-*d*) of **10**.

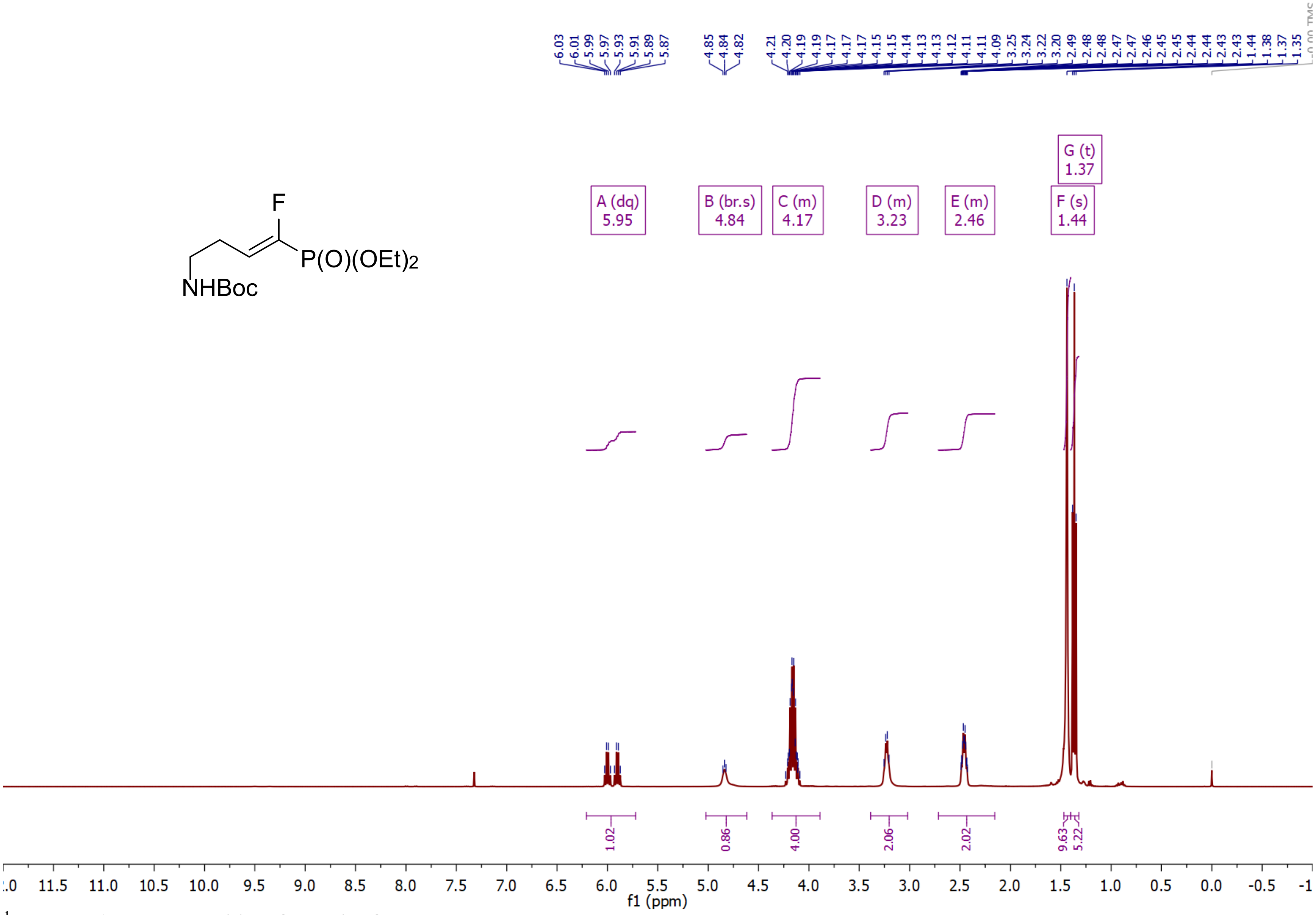

^{1}H NMR (400 MHz, Chloroform-*d*) of **11a**.

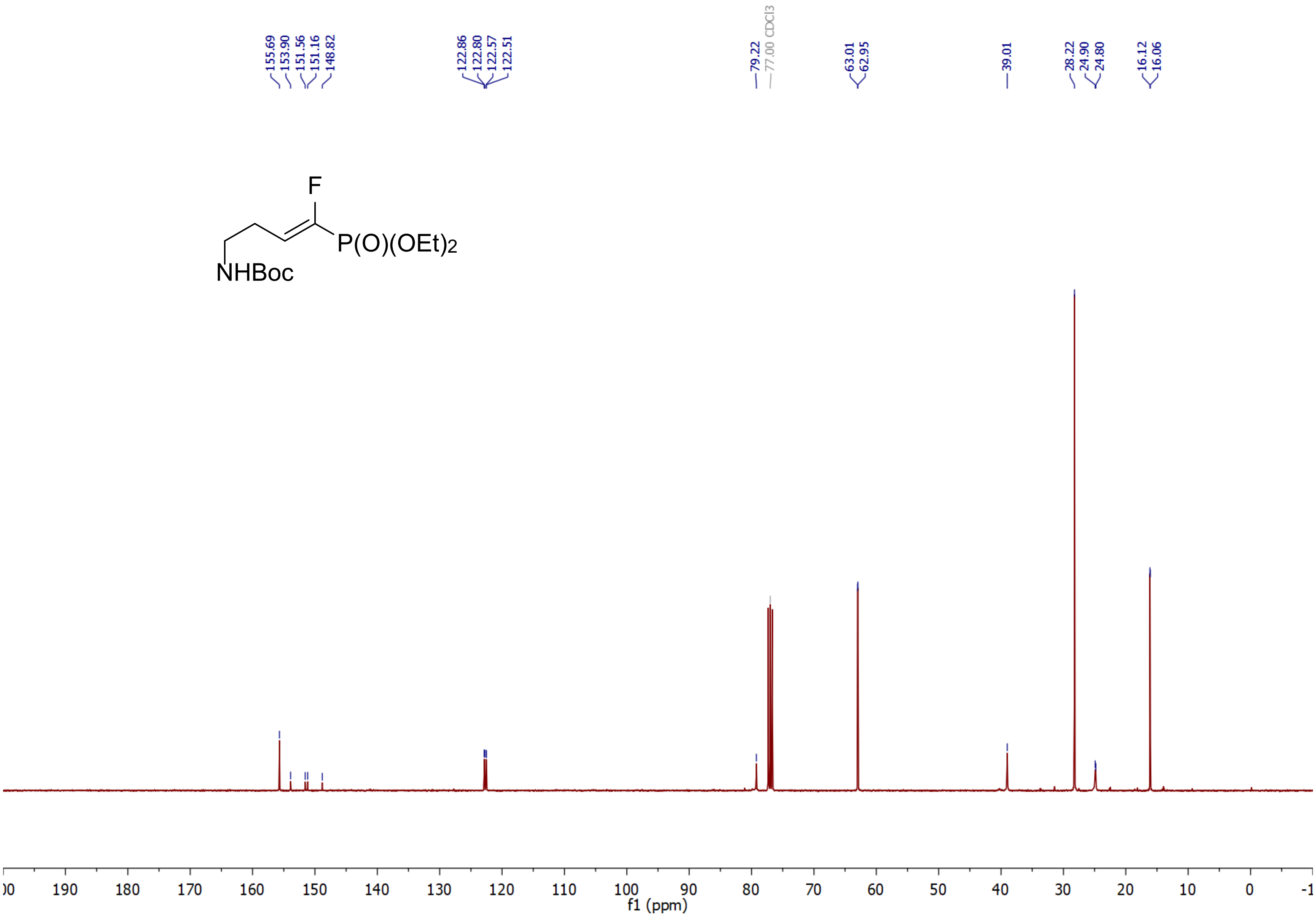

$^{13}C\{/^{1}H\}$ NMR (101 MHz, Chloroform-*d*) of **11a**.

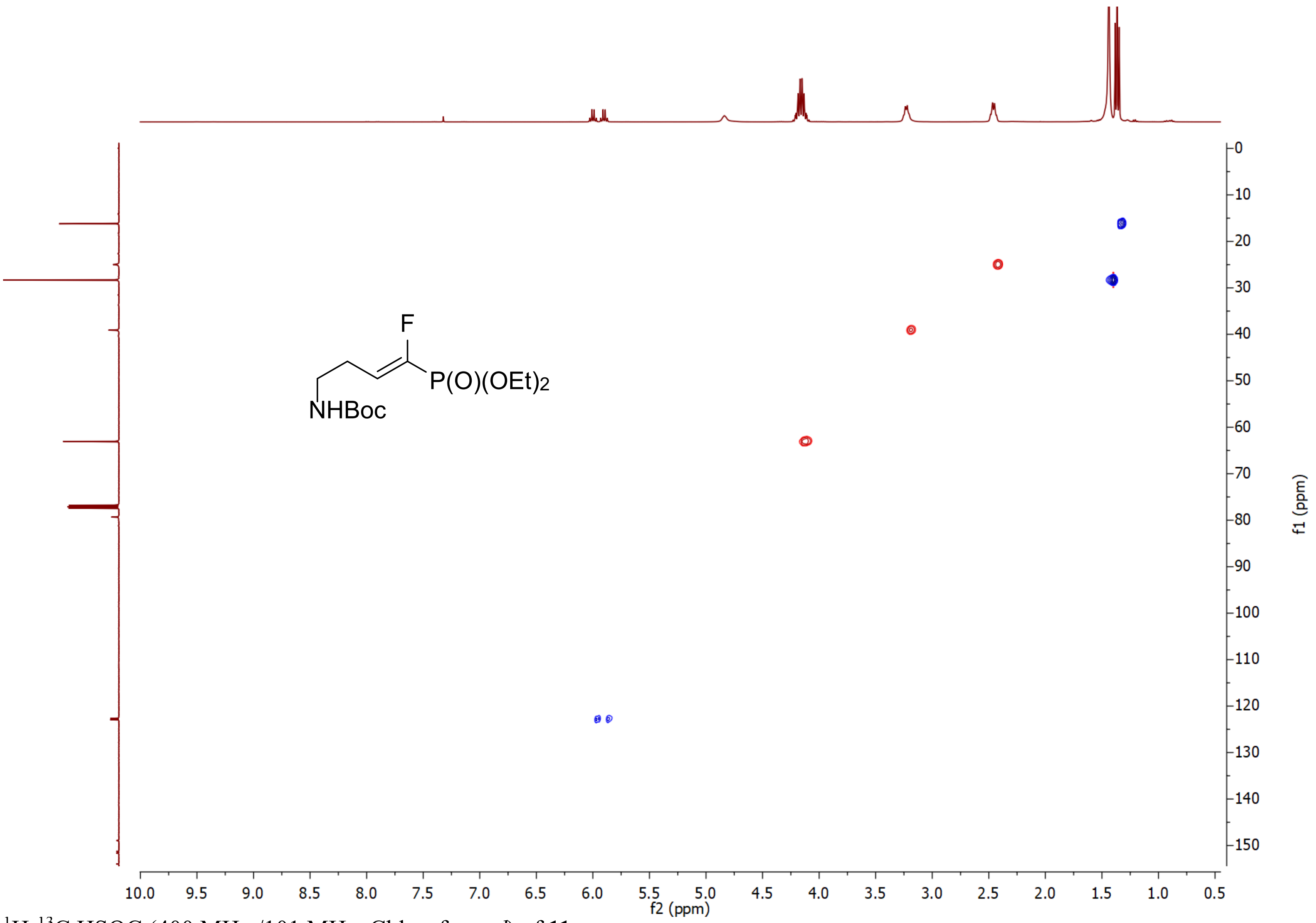

^{1}H-^{13}C HSQC (400 MHz /101 MHz, Chloroform-*d*) of **11a**.

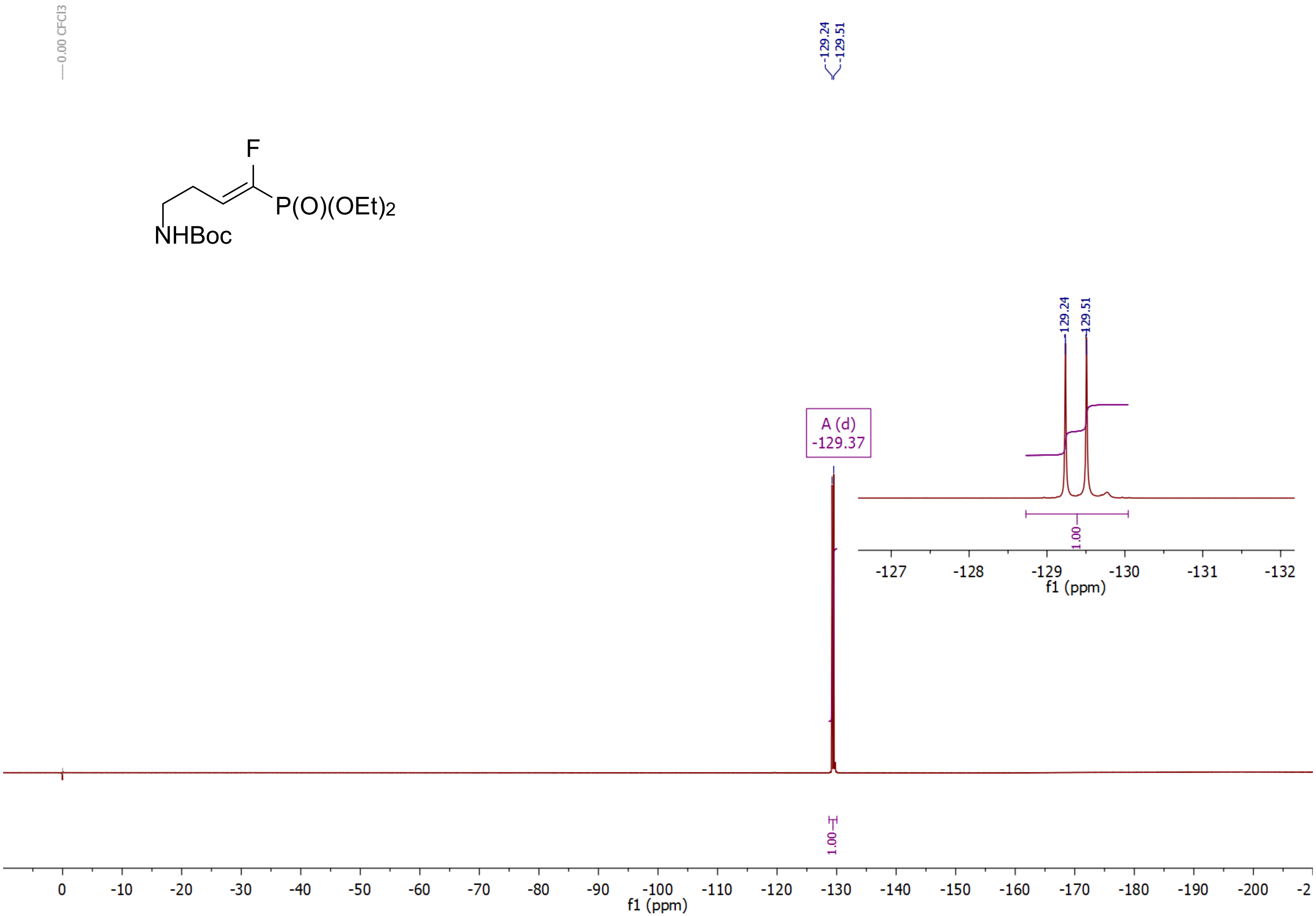

^{19}F NMR (377 MHz, Chloroform-*d*) of **11a**.

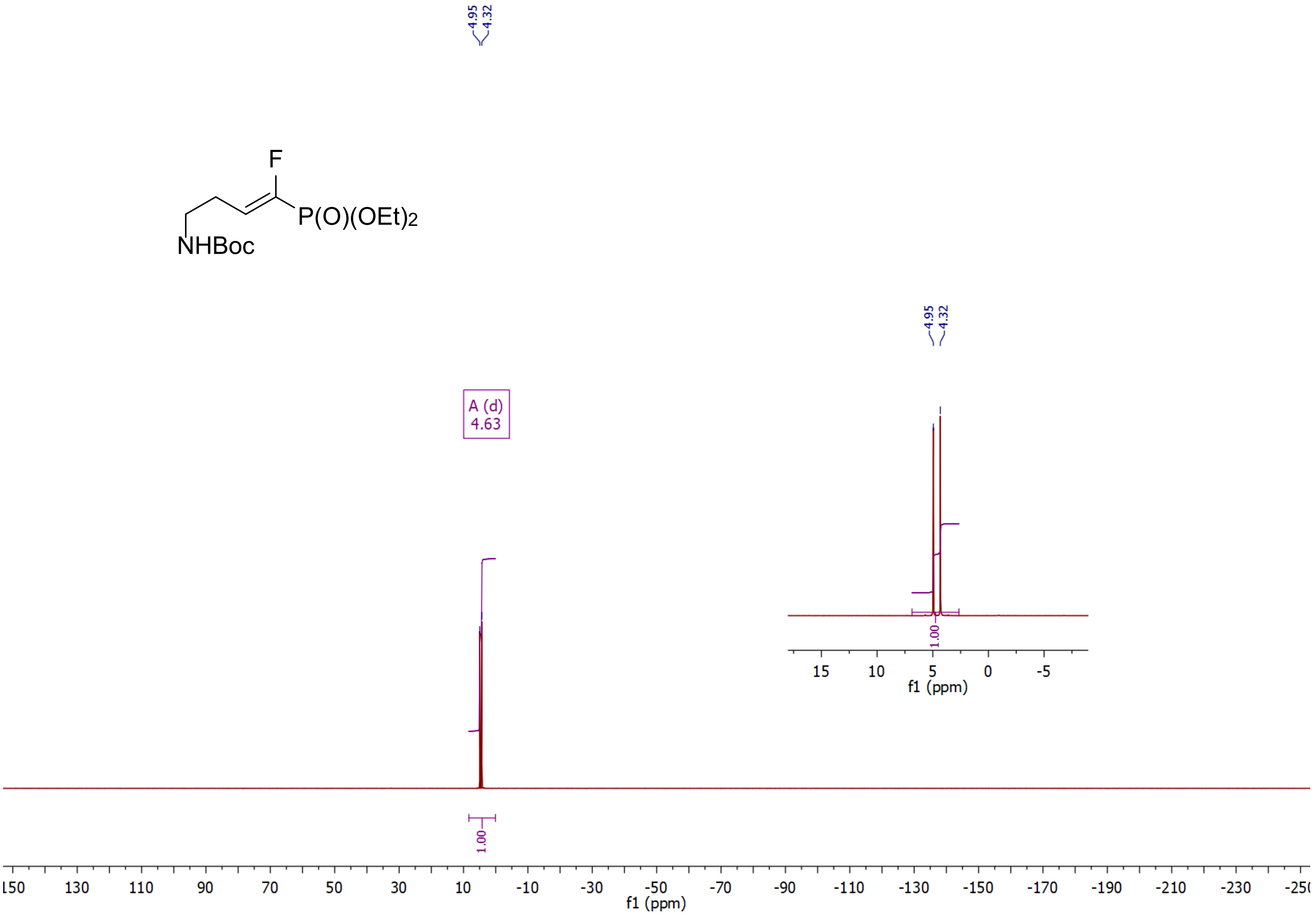

$^{31}P\{/^{1}H\}$ NMR (162 MHz, Chloroform-*d*) of **11a**.

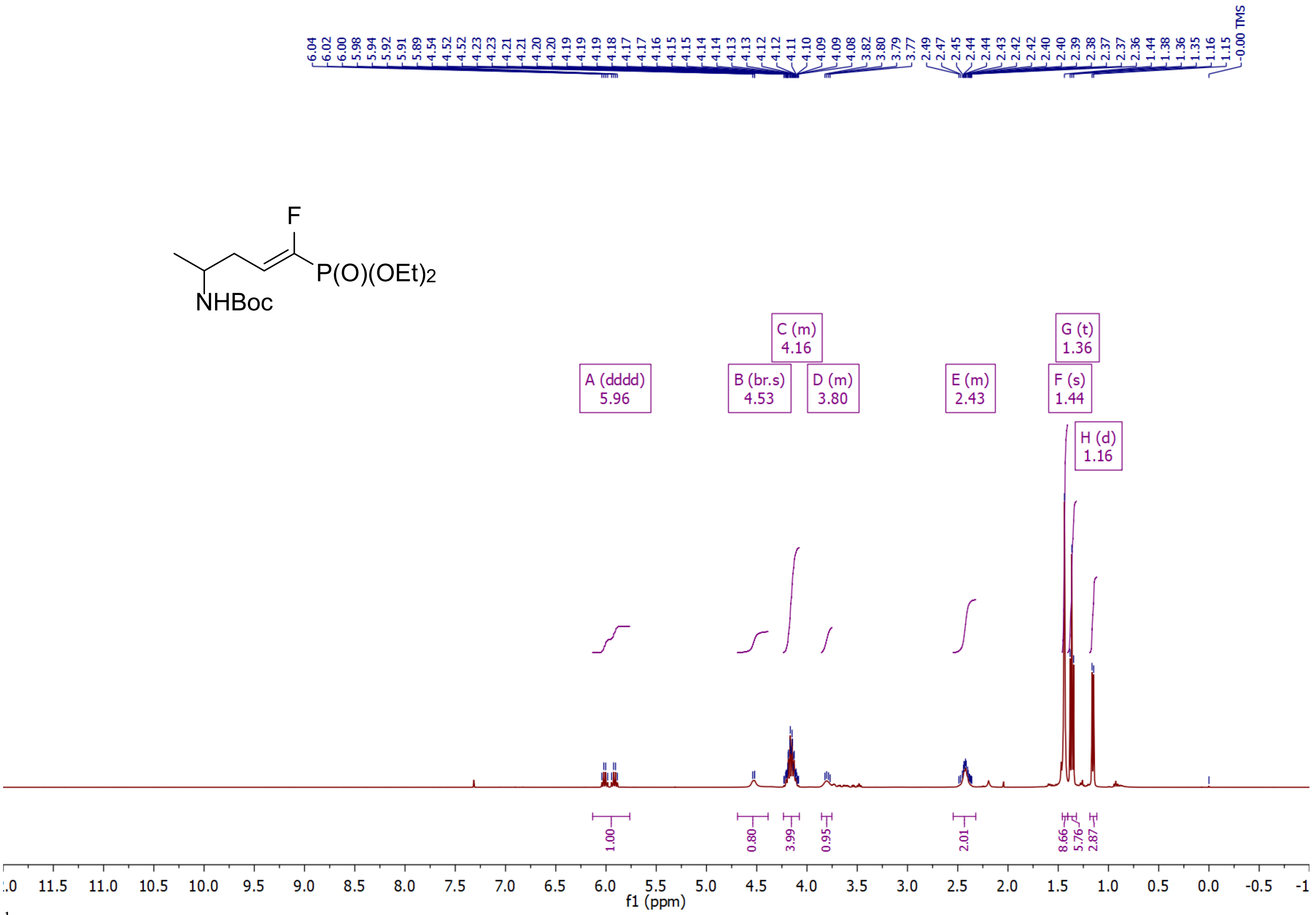

^{1}H NMR (400 MHz, Chloroform-*d*) of ***rac*-11b**.

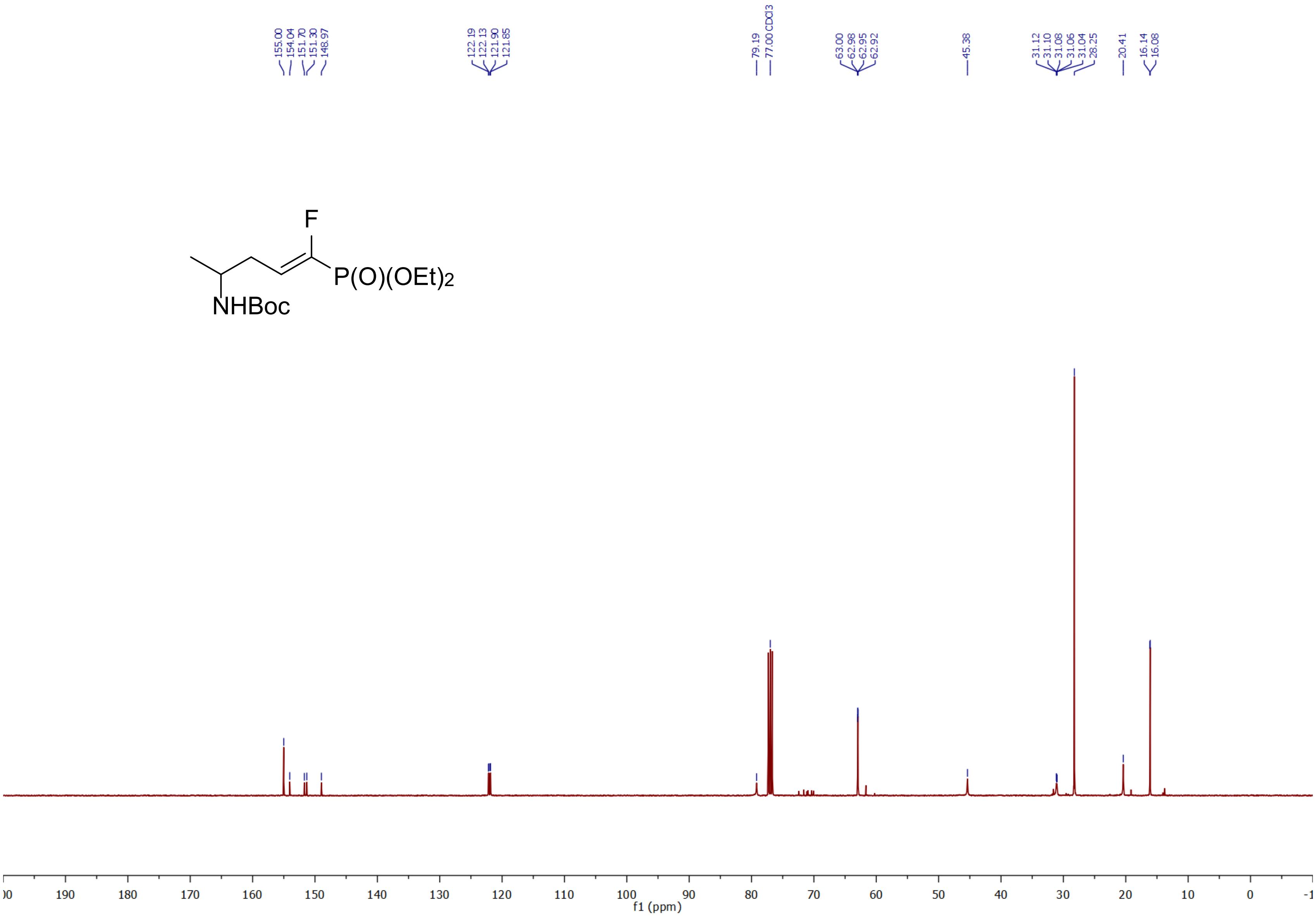

$^{13}C\{/^{1}H\}$ NMR (101 MHz, Chloroform-*d*) of ***rac*-11b**.

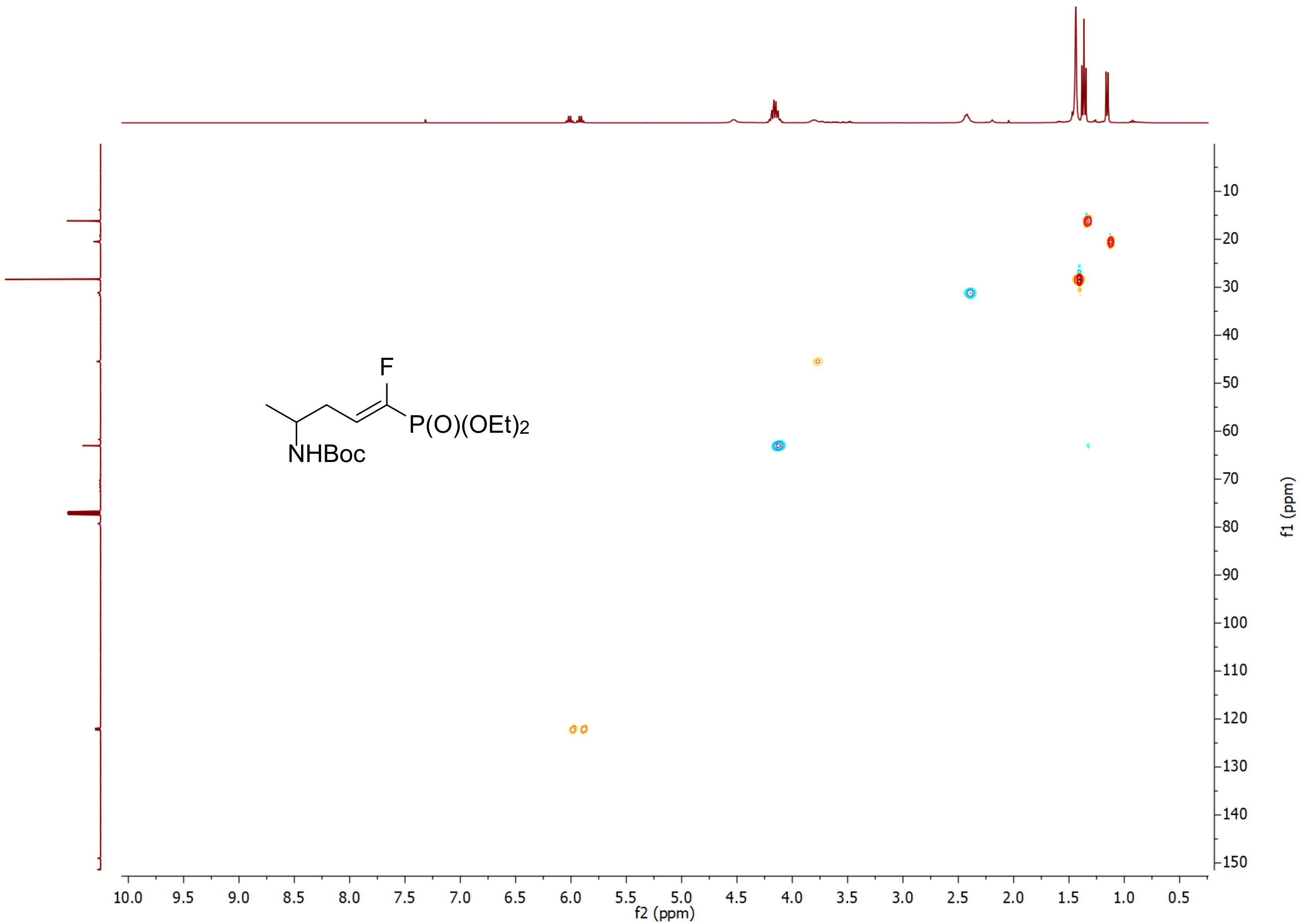

^{1}H-^{13}C HSQC (400 MHz /101 MHz, Chloroform-*d*) of ***rac*-11b**.

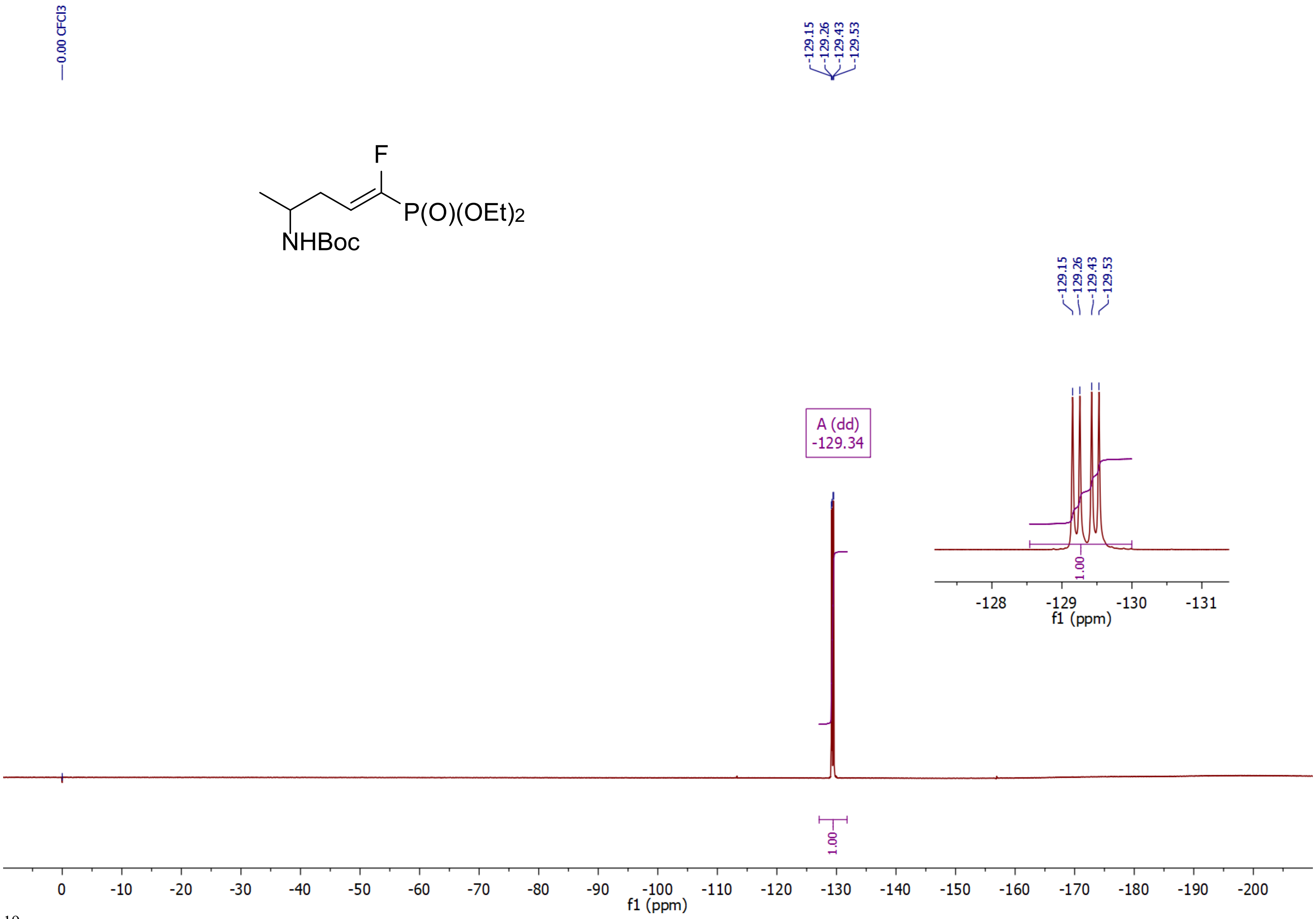

^{19}F NMR (377 MHz, Chloroform-*d*) of ***rac*-11b**.

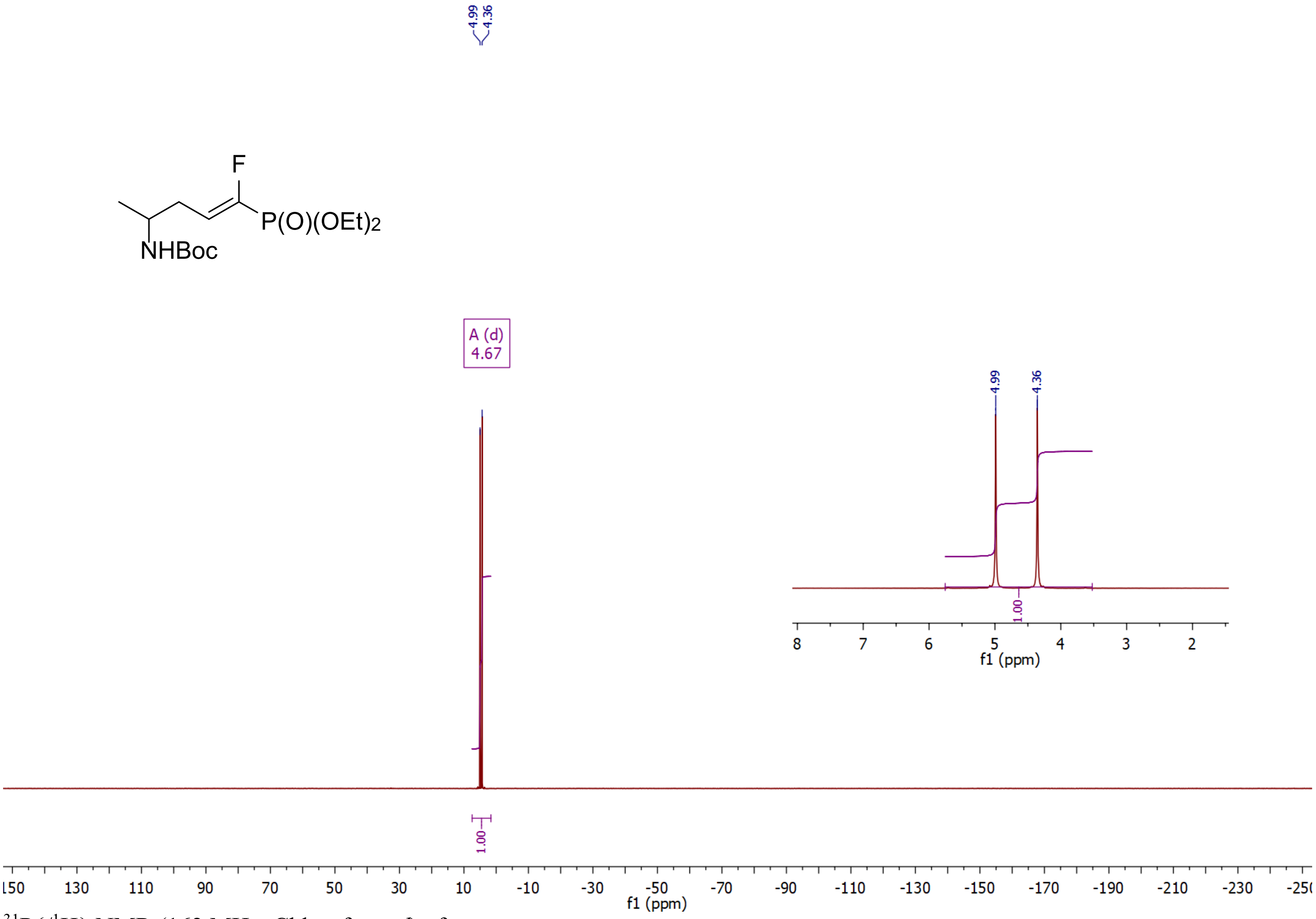

$^{31}P\{/^{1}H\}$ NMR (162 MHz, Chloroform-*d*) of ***rac*-11b**.

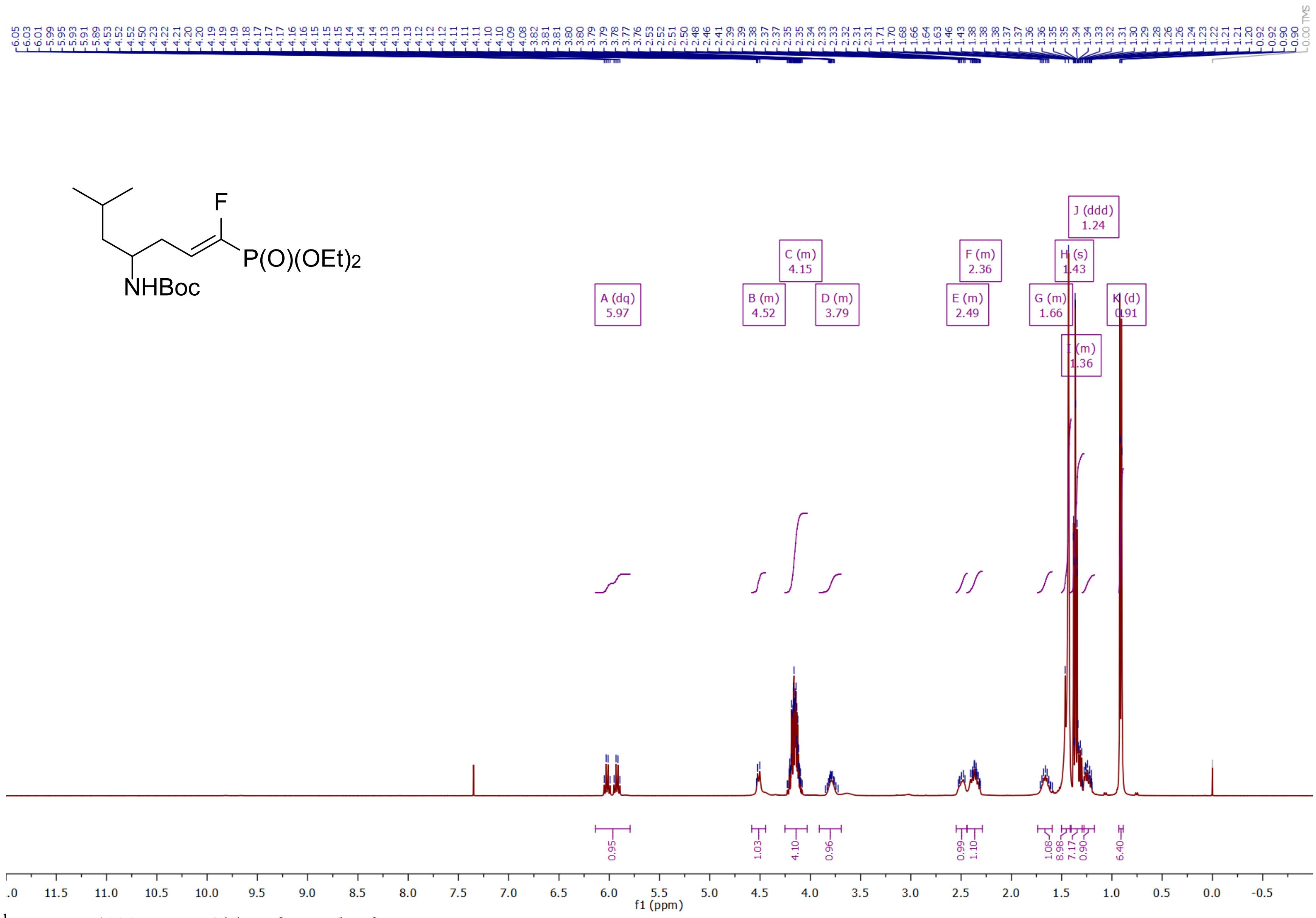

^{1}H NMR (400 MHz, Chloroform-*d*) of ***rac*-11c**.

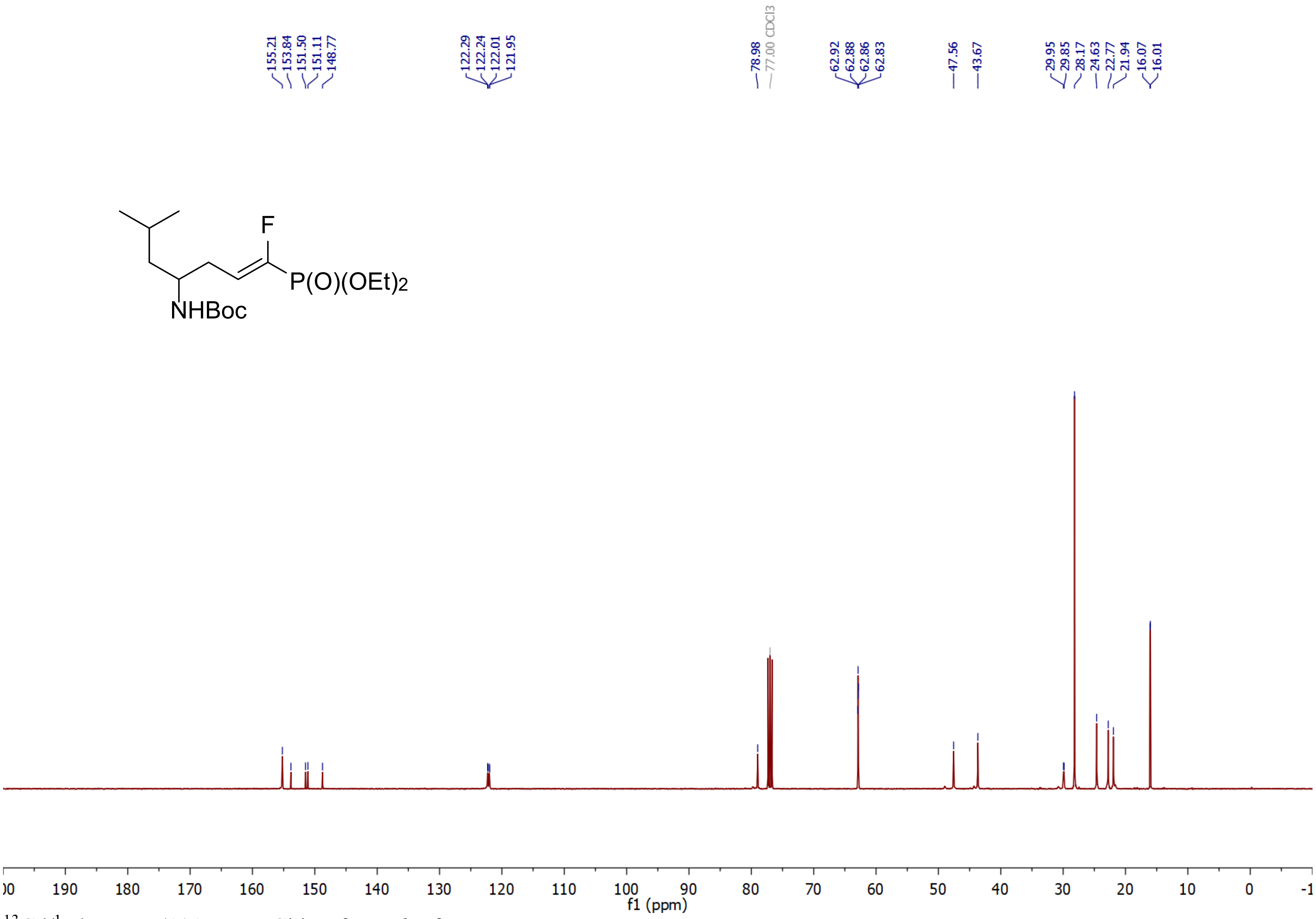

$^{13}C\{/^{1}H\}$ NMR (101 MHz, Chloroform-*d*) of ***rac*-11c**.

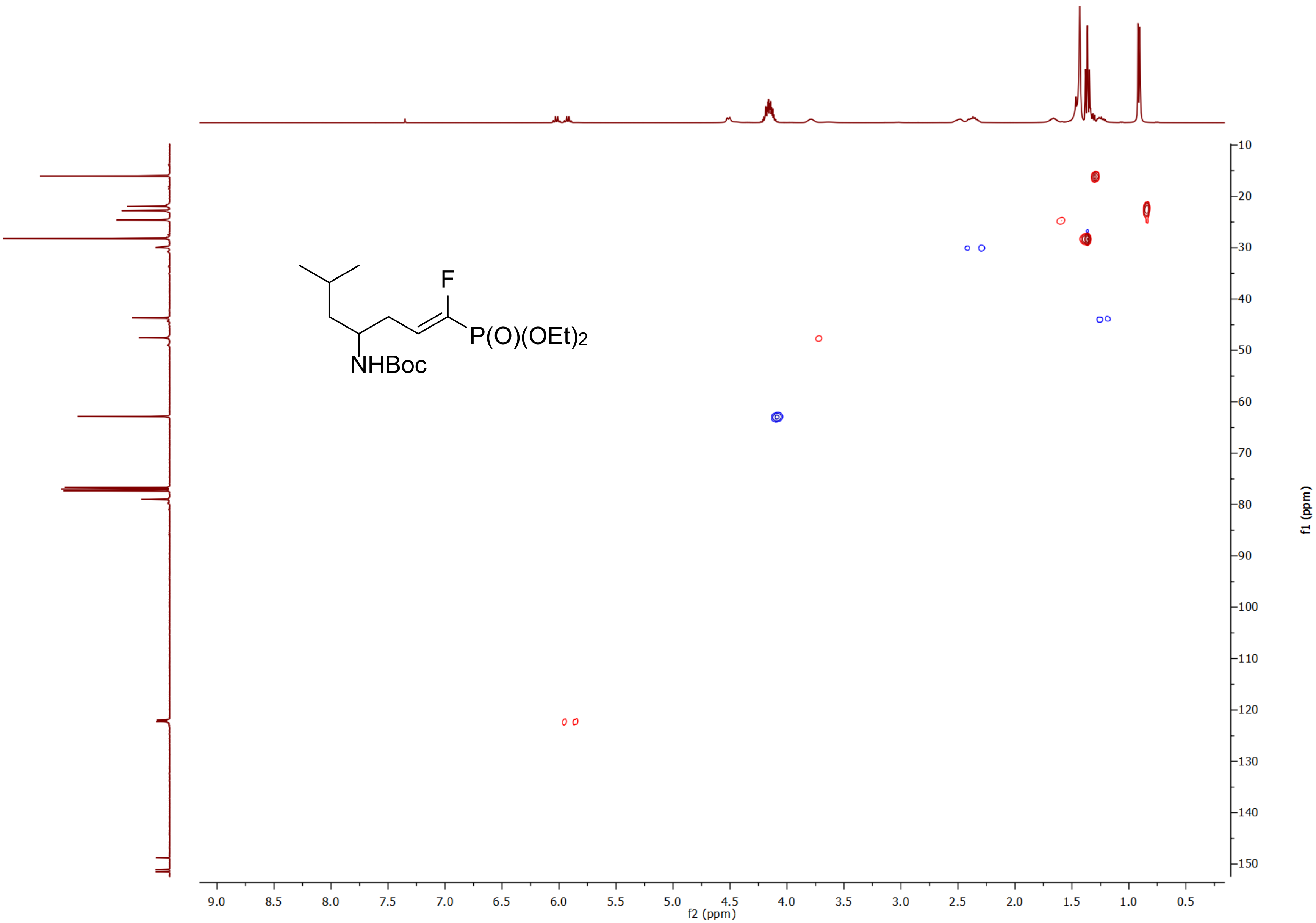

^{1}H-^{13}C HSQC (400 MHz /101 MHz, Chloroform-*d*) of ***rac*-11c**.

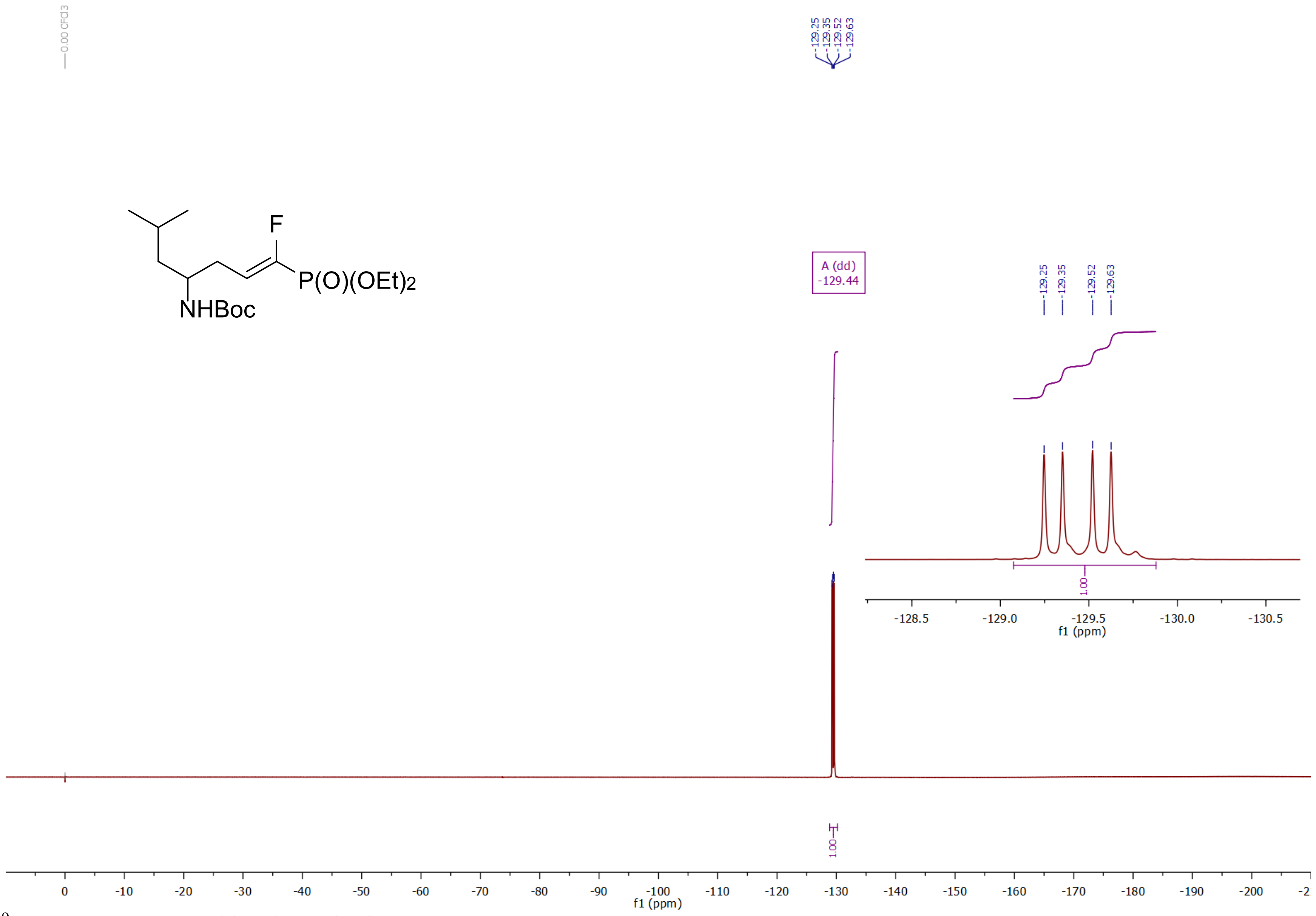

^{19}F NMR (377 MHz, Chloroform-*d*) of ***rac*-11c**.

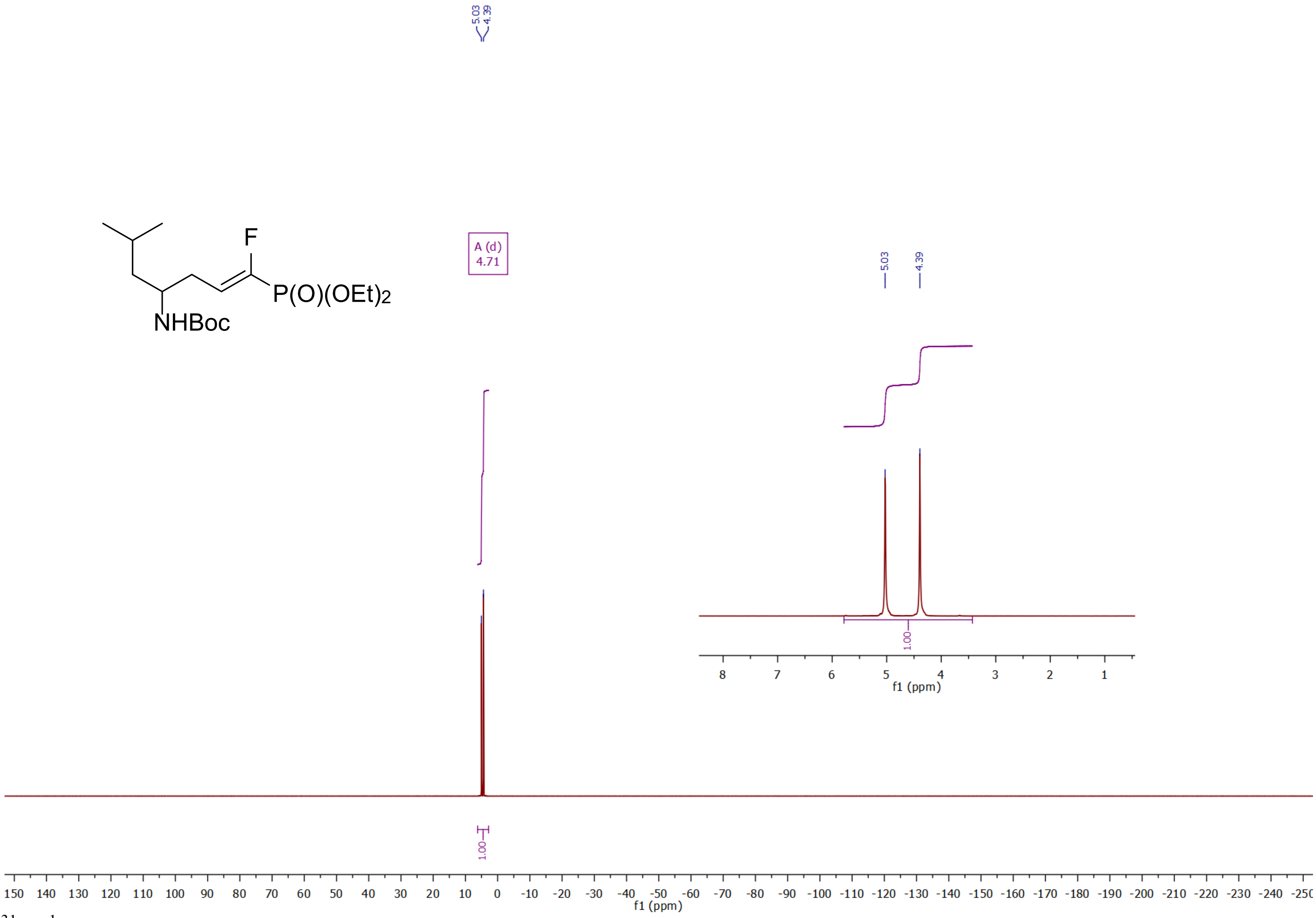

$^{31}P\{/^{1}H\}$ NMR (162 MHz, Chloroform-*d*) of ***rac*-11c**.

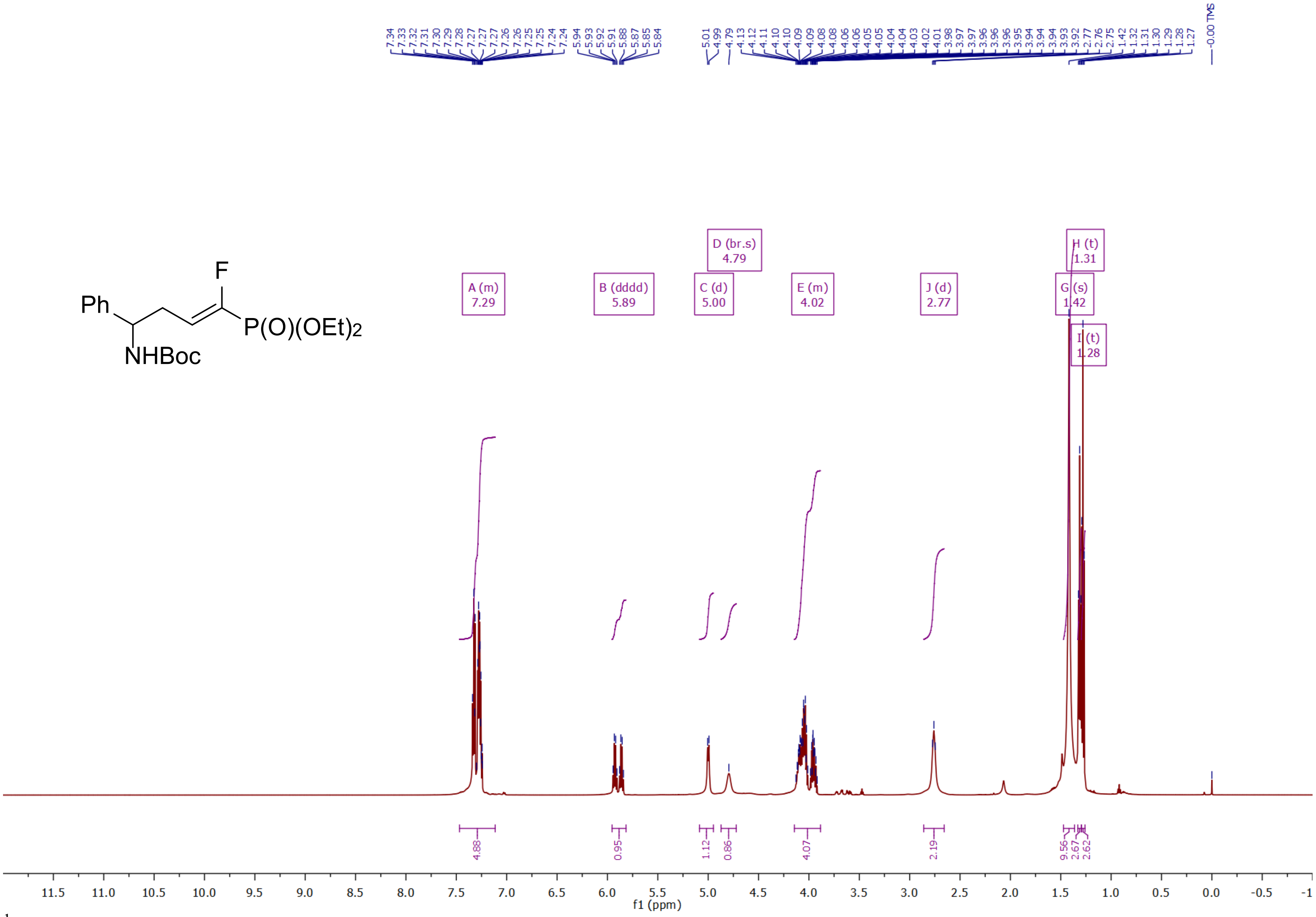

^{1}H NMR (600 MHz, Chloroform-*d*) of ***rac*-11d**.

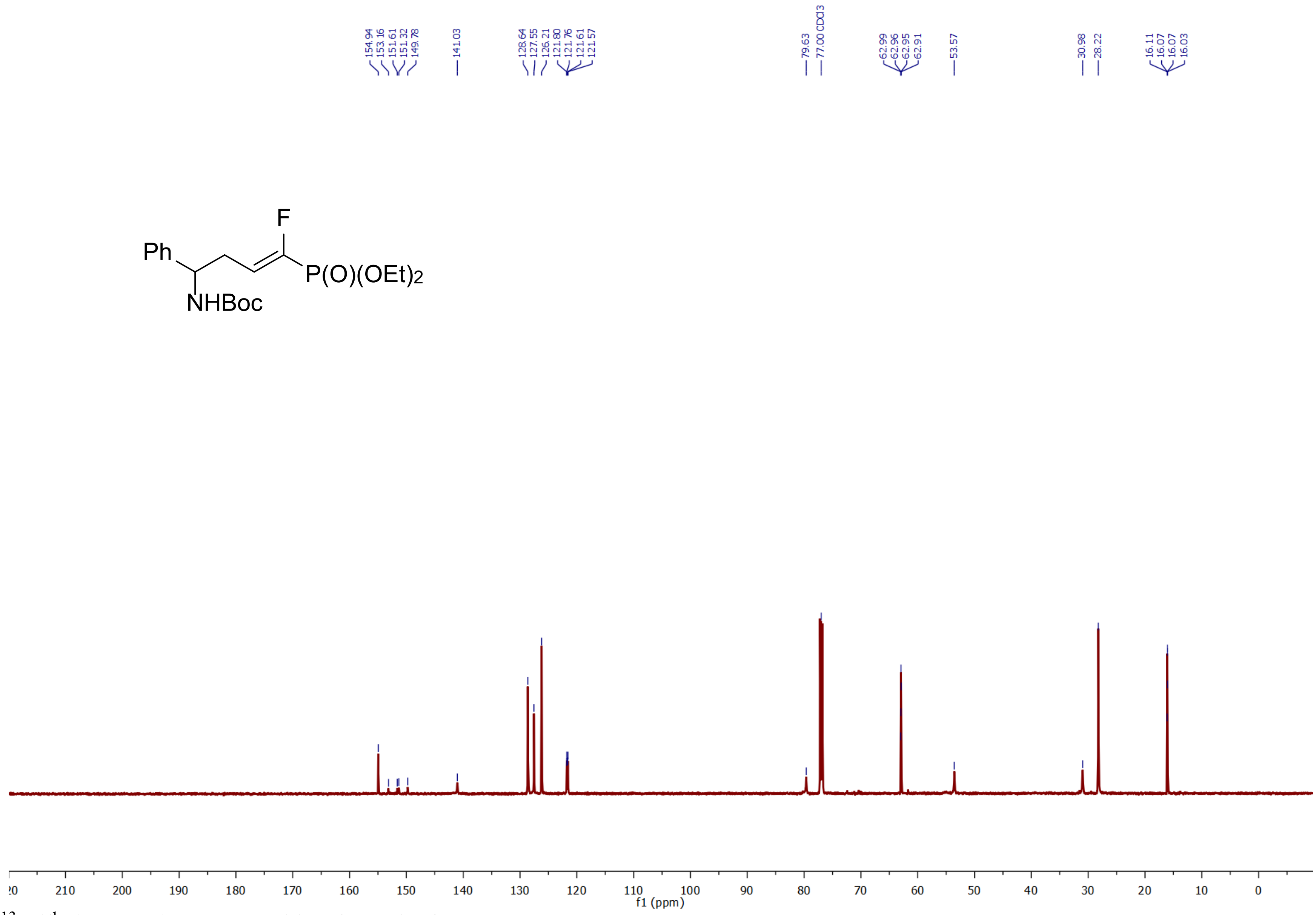

$^{13}C\{/^{1}H\}$ NMR (151 MHz, Chloroform-*d*) of ***rac*-11d**.

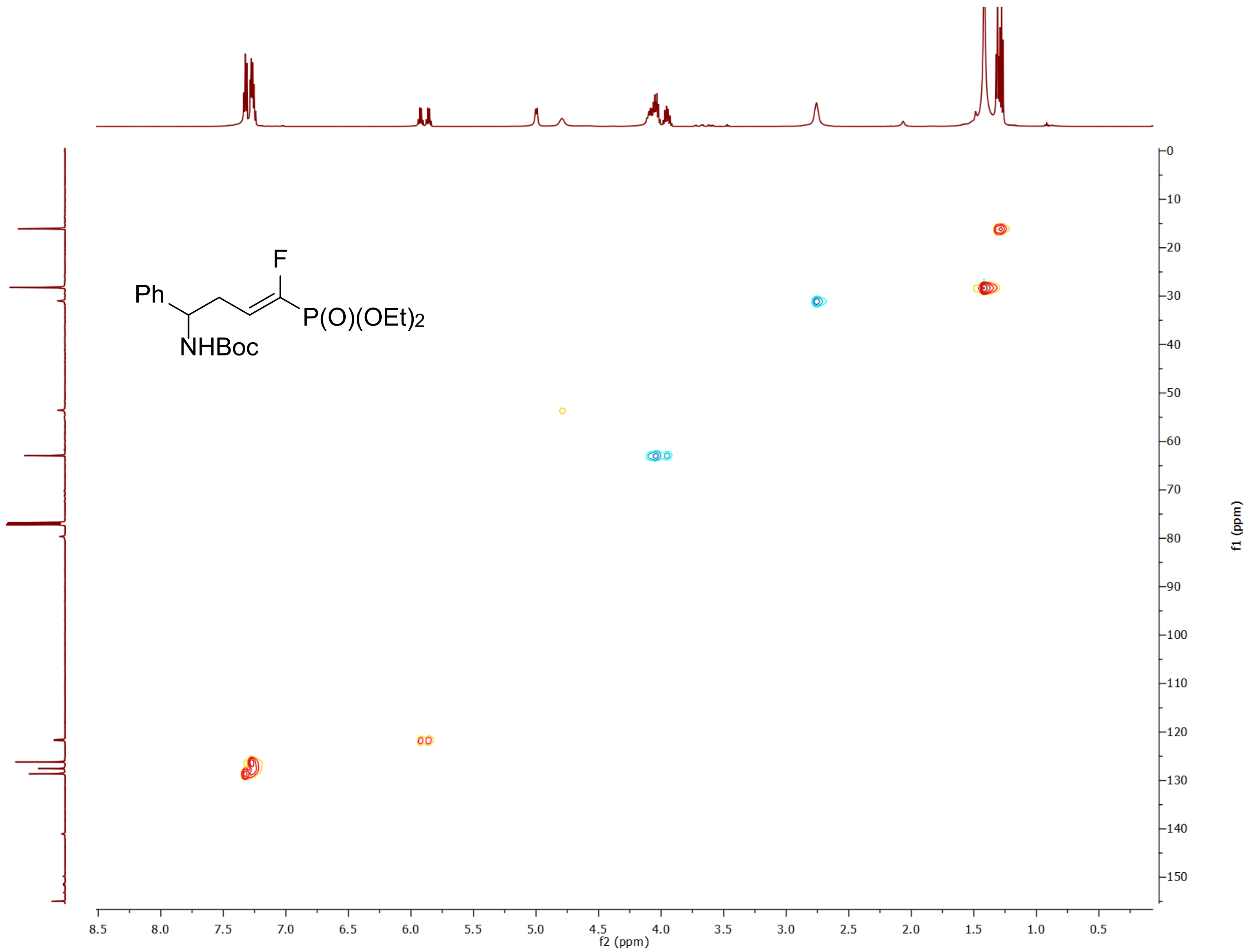

^{1}H-^{13}C HSQC (600 MHz /151 MHz,Chloroform-*d*) of ***rac*-11d**.

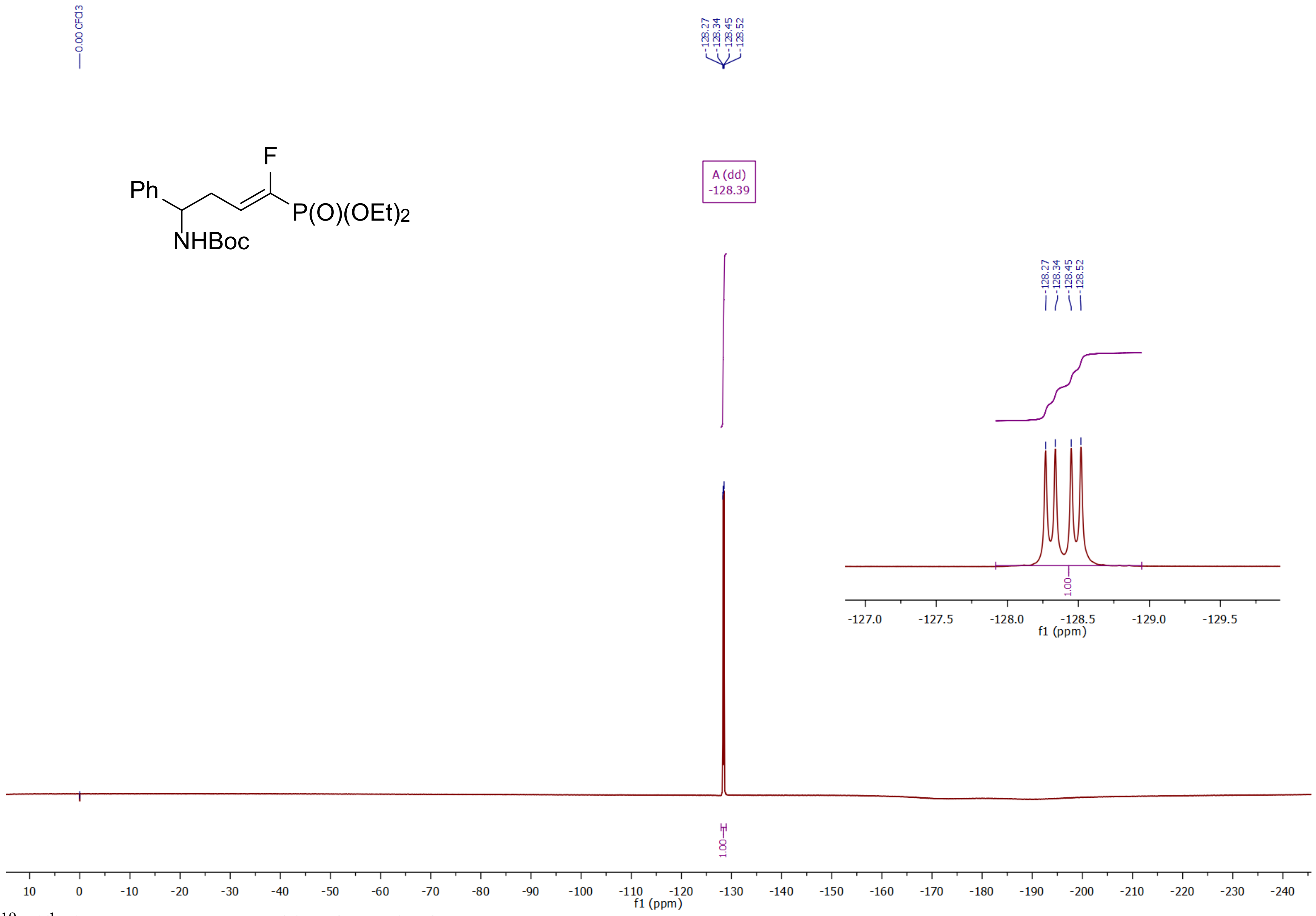

$^{19}F\{/^{1}H\}$ NMR (565 MHz, Chloroform-*d*) of ***rac*-11d**.

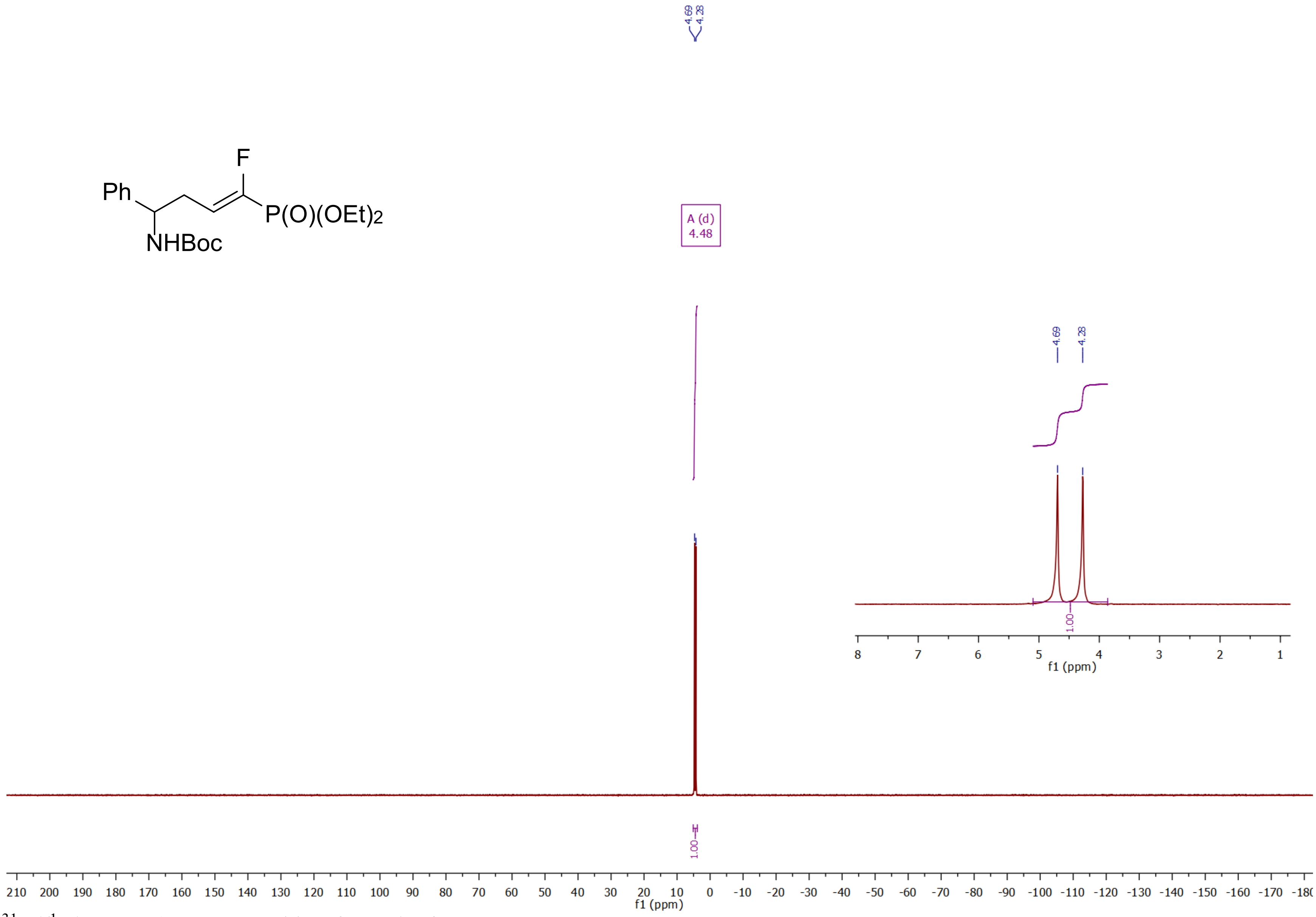

$^{31}P\{/^{1}H\}$ NMR (243 MHz, Chloroform-*d*) of ***rac*-11d**.

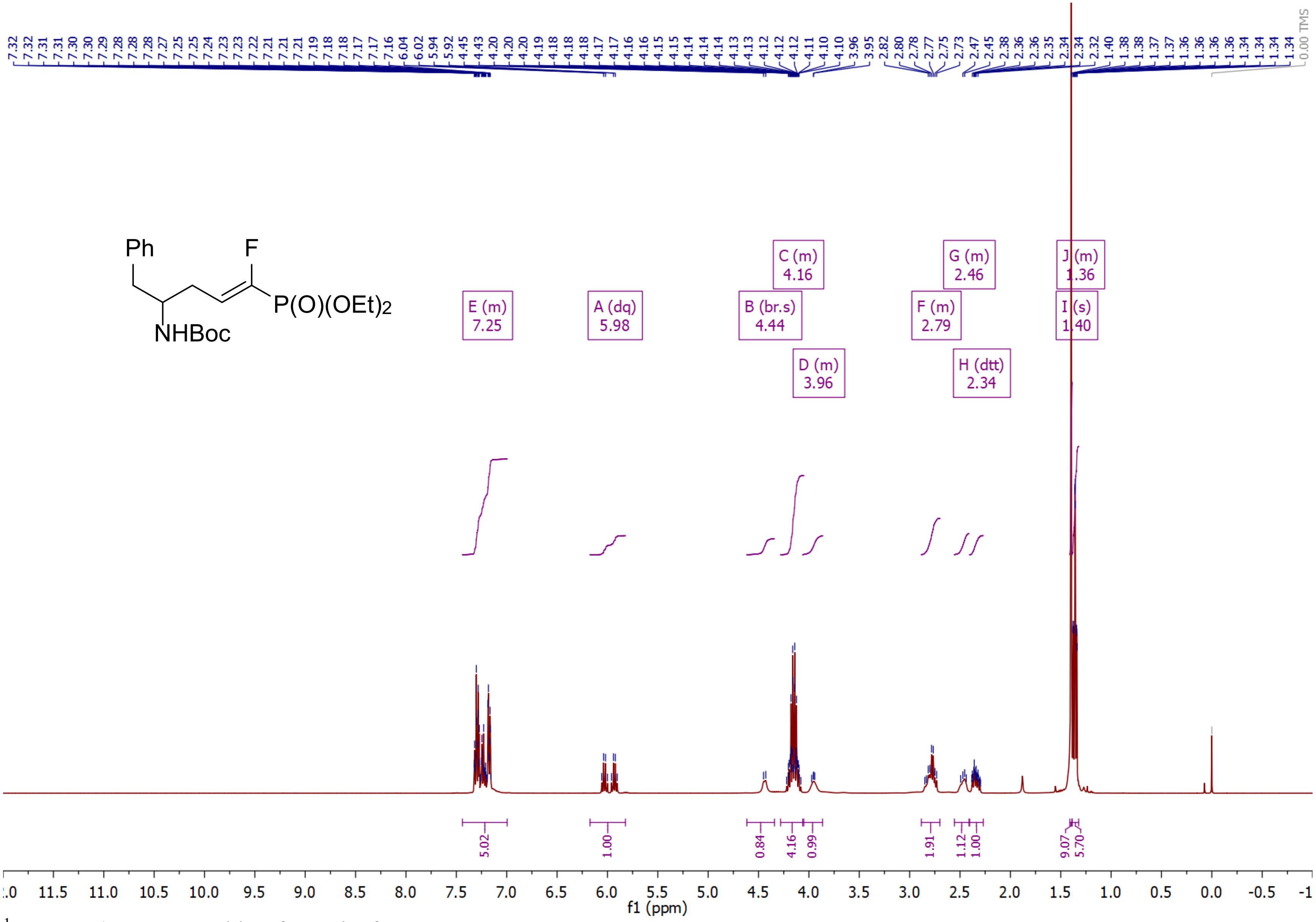

^{1}H NMR (400 MHz, Chloroform-*d*) of ***rac*-11e**.

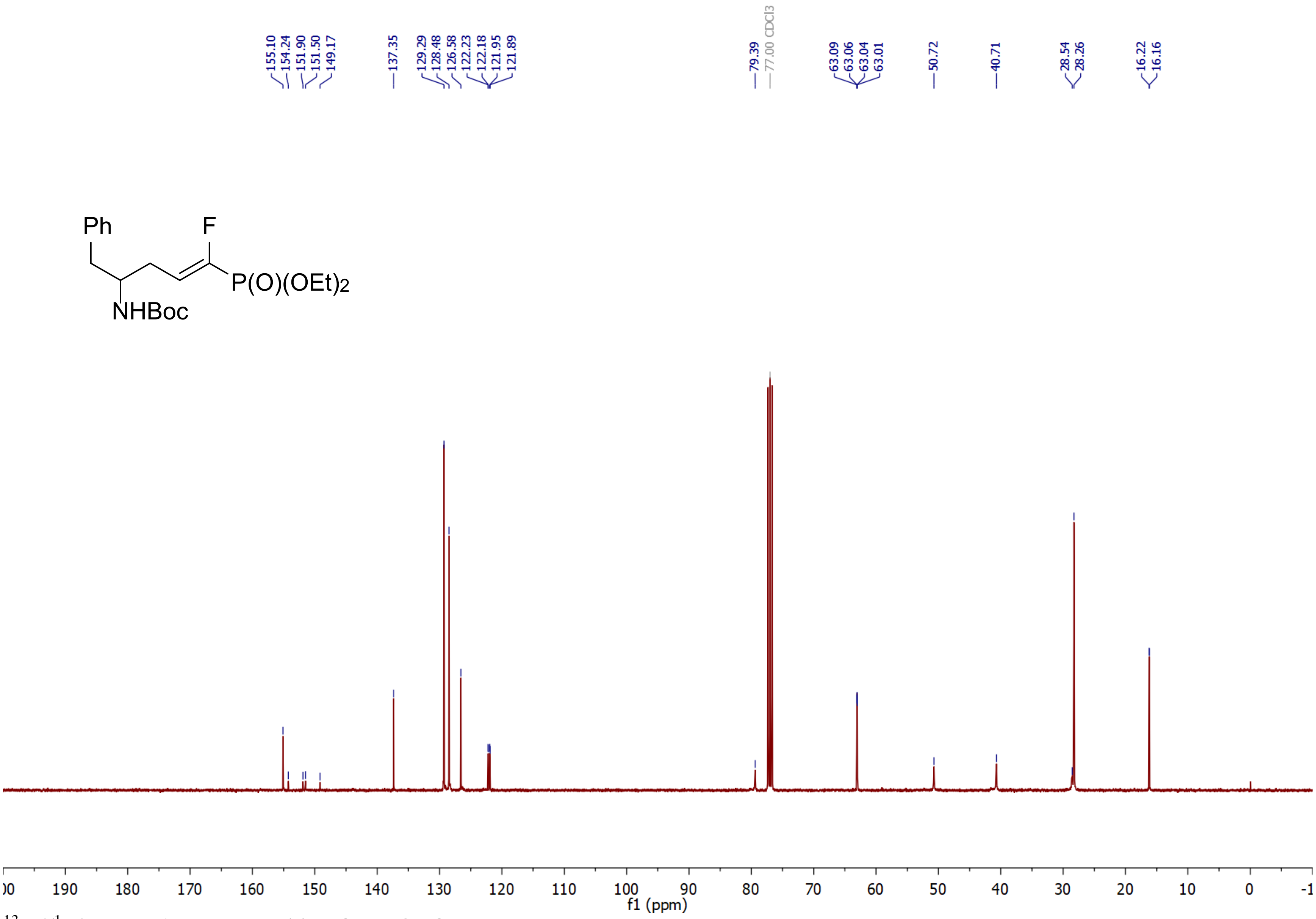

$^{13}C\{/^{1}H\}$ NMR (101 MHz, Chloroform-*d*) of ***rac*-11e**.

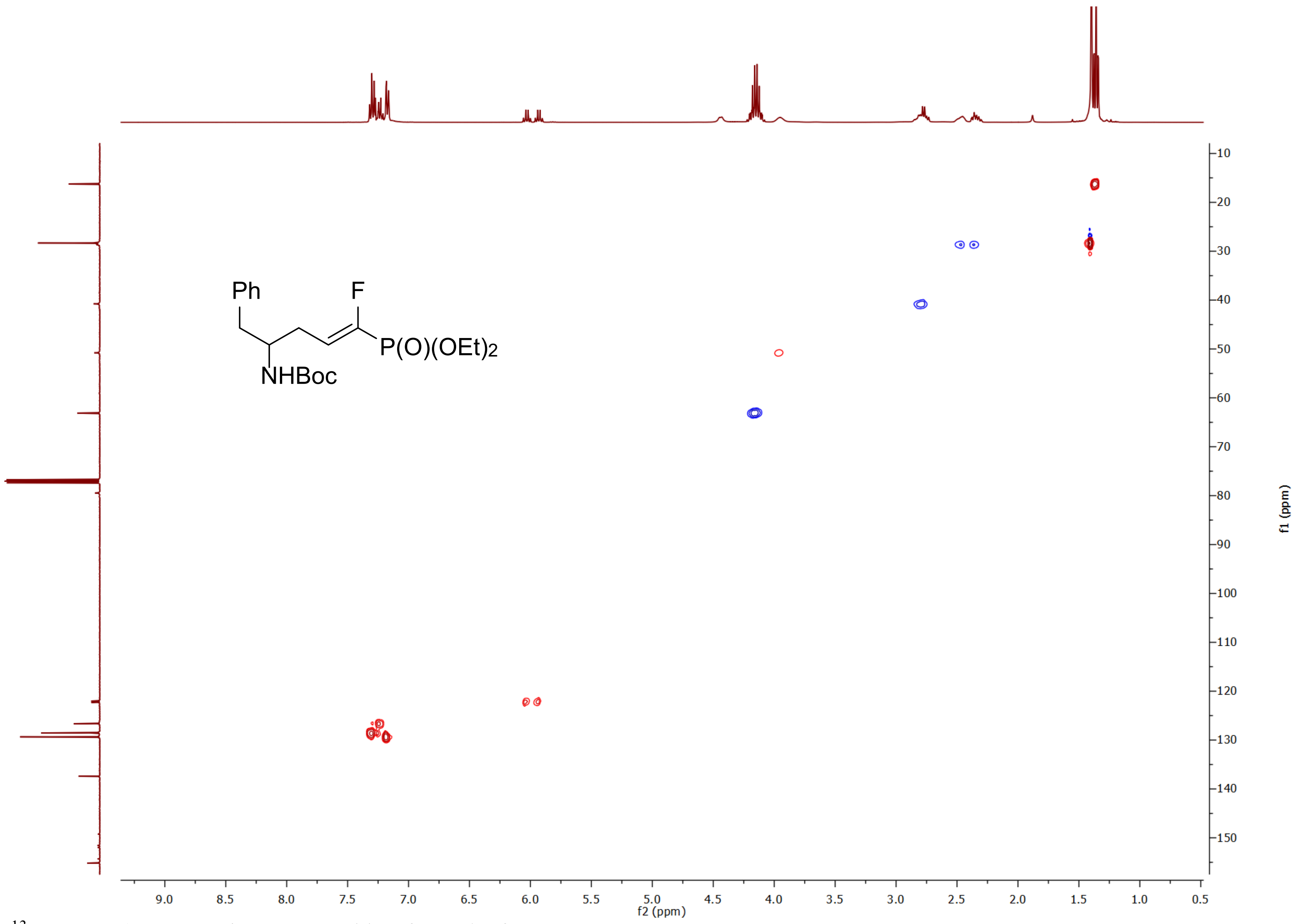

^{1}H-^{13}C HSQC (400 MHz /101 MHz, Chloroform-*d*) of ***rac*-11e**.

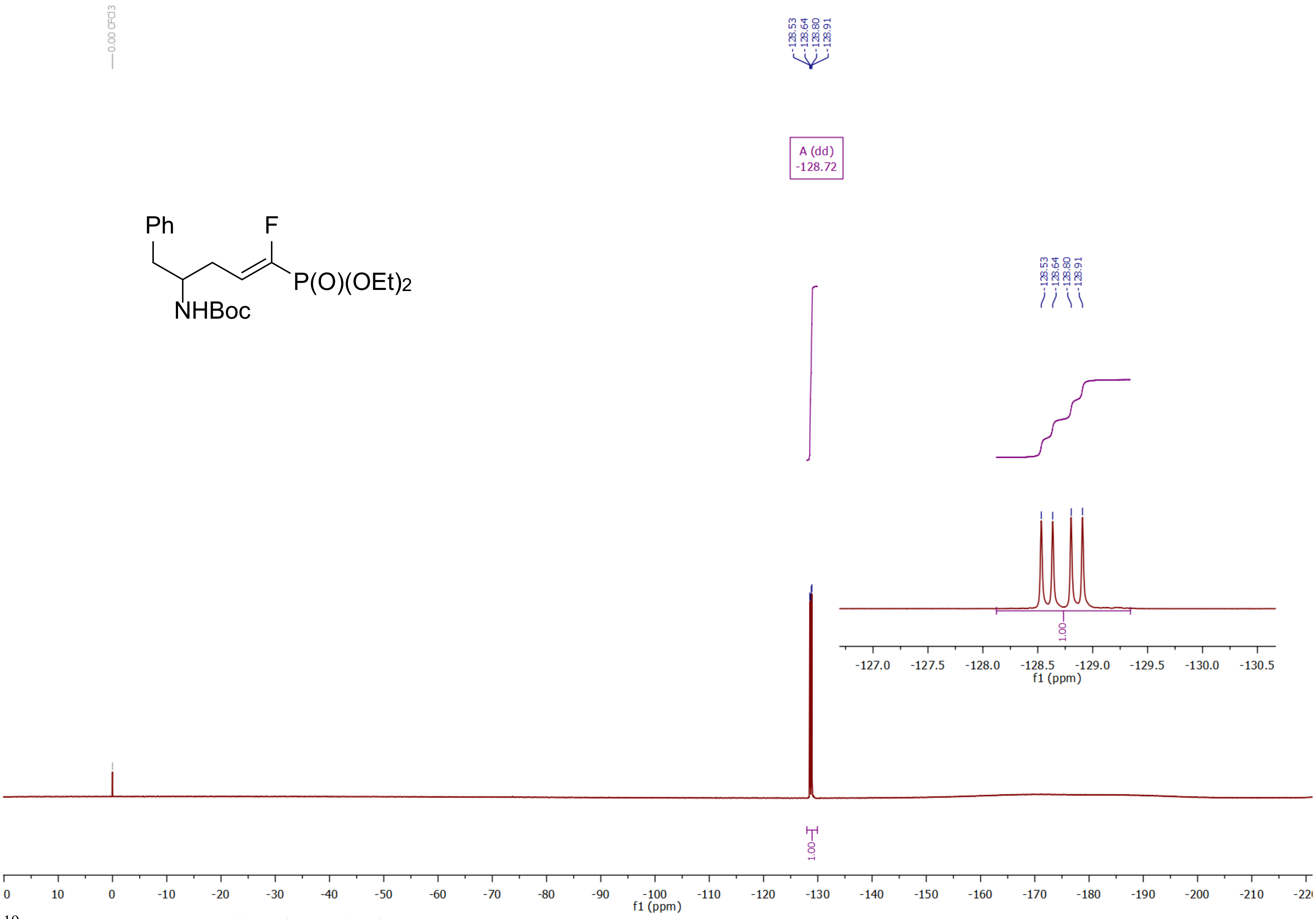

^{19}F NMR (377 MHz, Chloroform-*d*) of ***rac*-11e**.

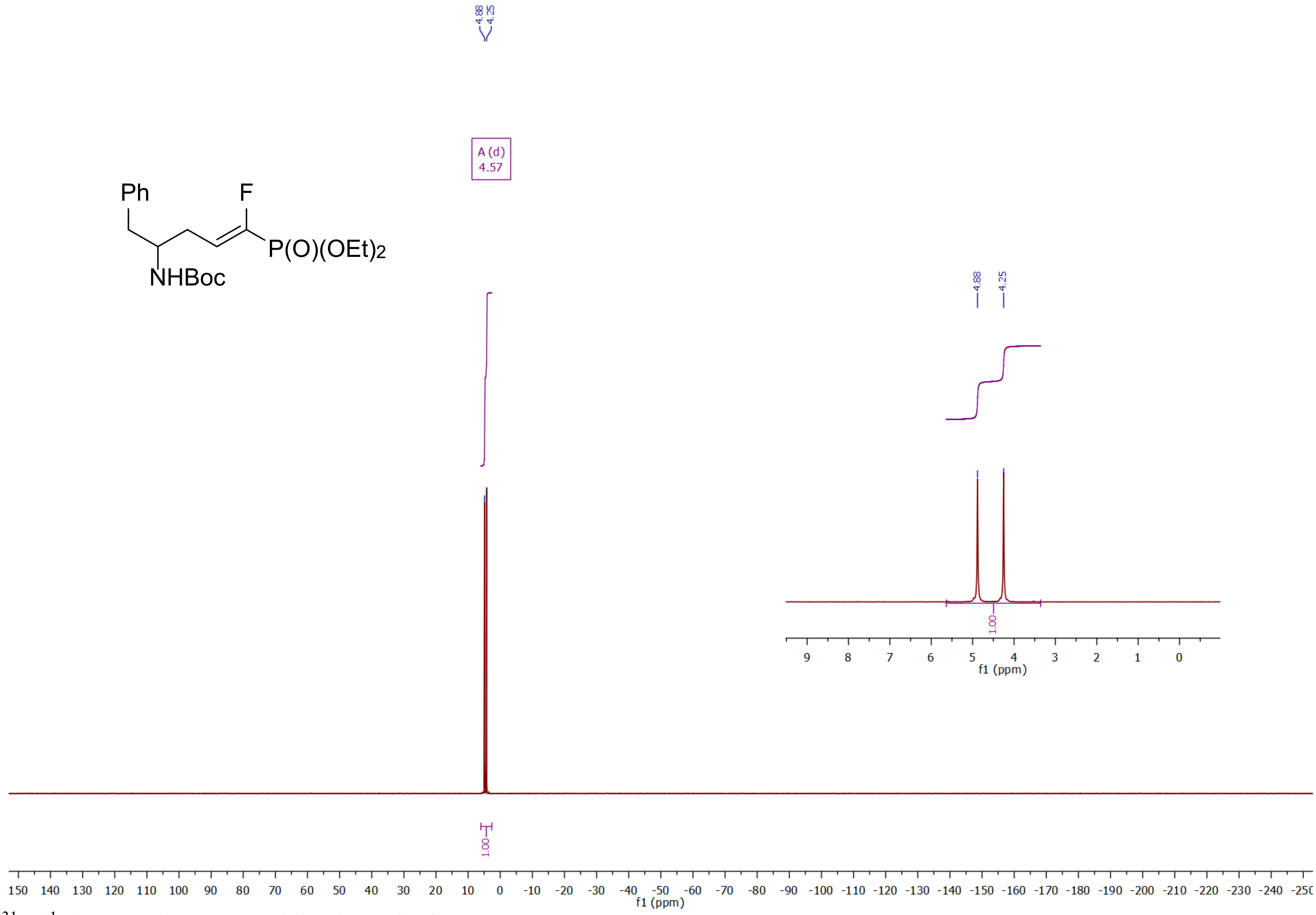

$^{31}P\{/^{1}H\}$ NMR (162 MHz, Chloroform-*d*) of ***rac*-11e**.

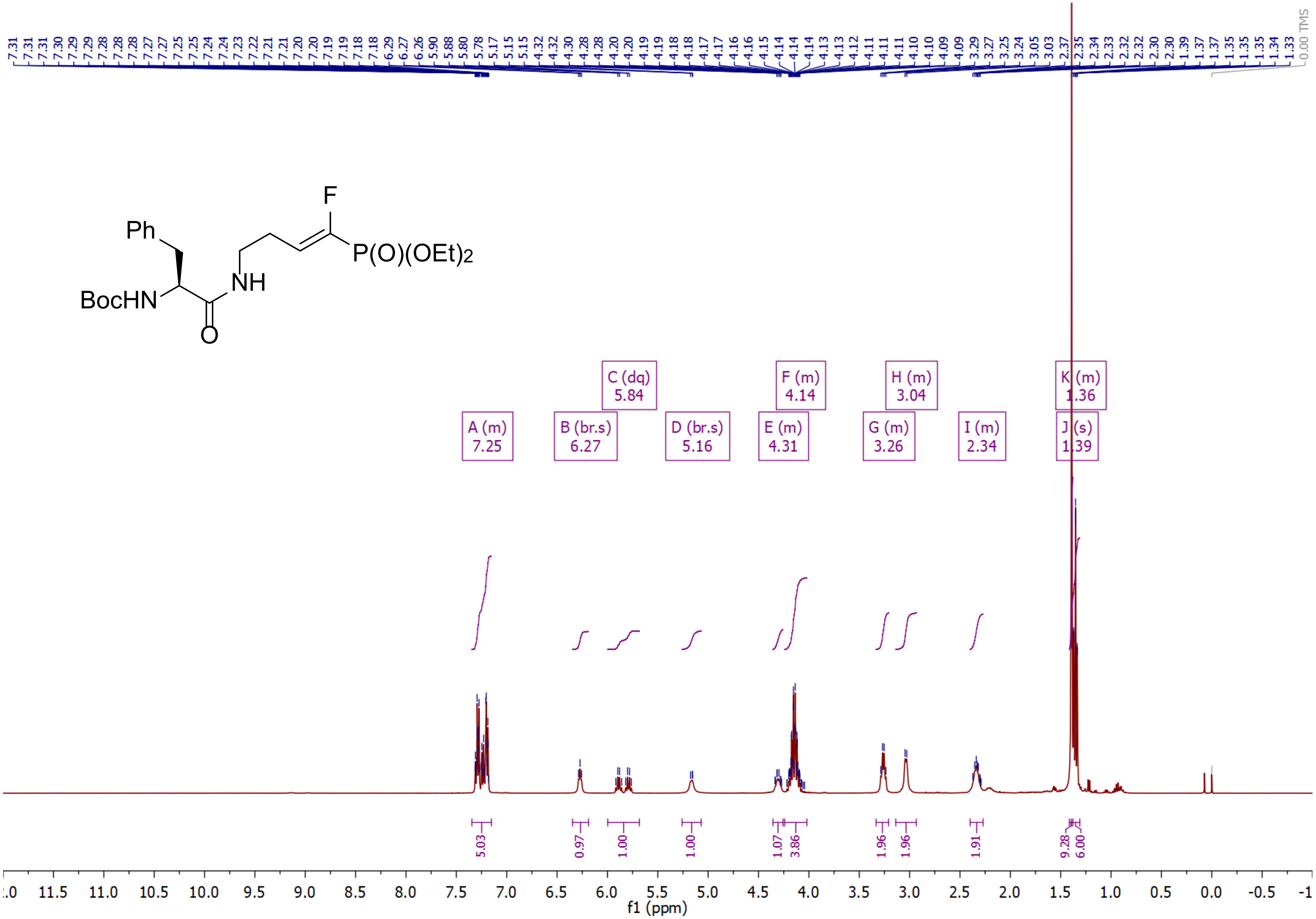

^{1}H NMR (400 MHz, Chloroform-*d*) of **14a**.

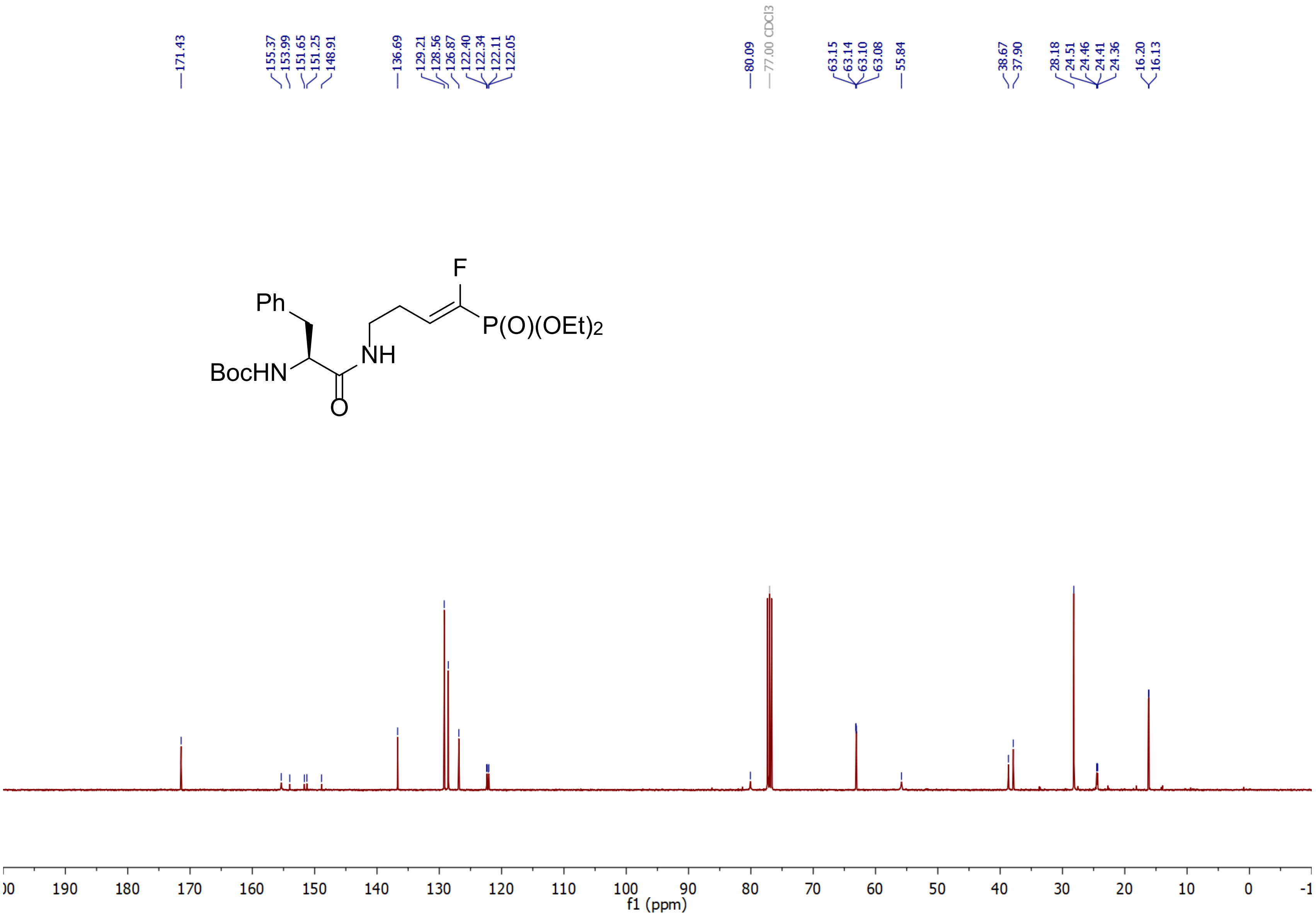

$^{13}C\{/^{1}H\}$ NMR (101 MHz, Chloroform-*d*) of **14a**.

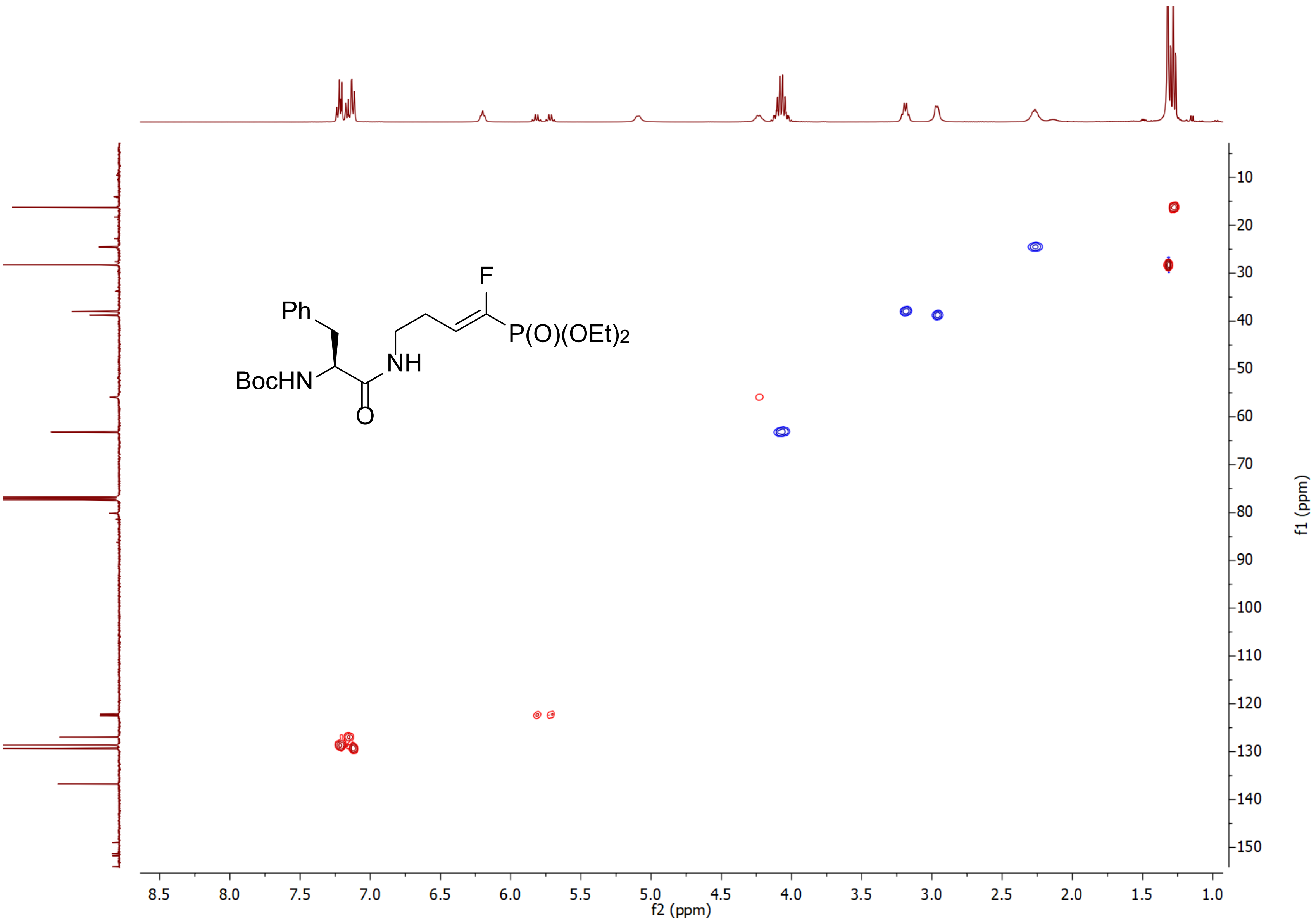

^{1}H-^{13}C HSQC (400 MHz /101 MHz, Chloroform-*d*) of **14a**.

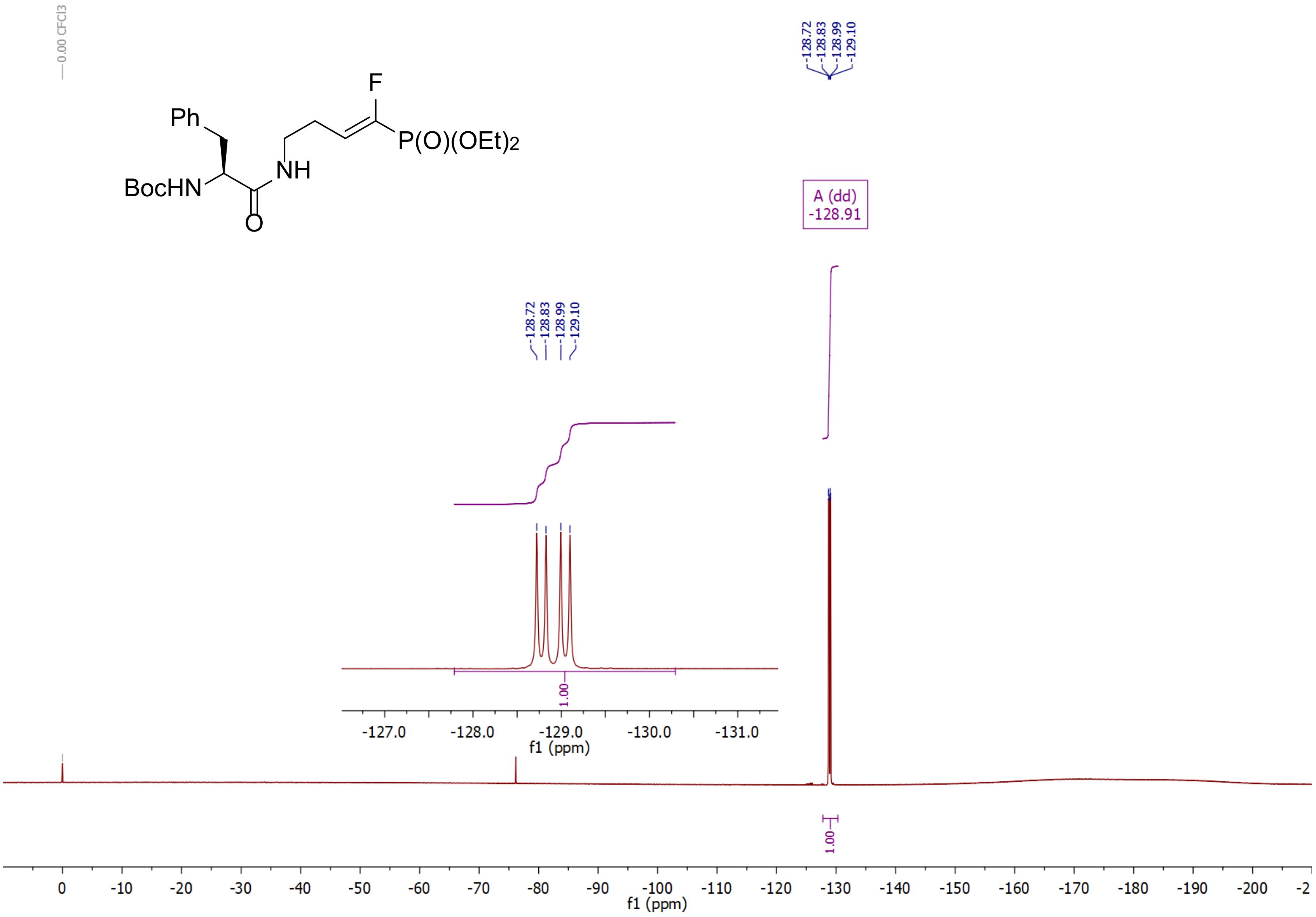

^{19}F NMR (377 MHz, Chloroform-*d*) of **14a**.

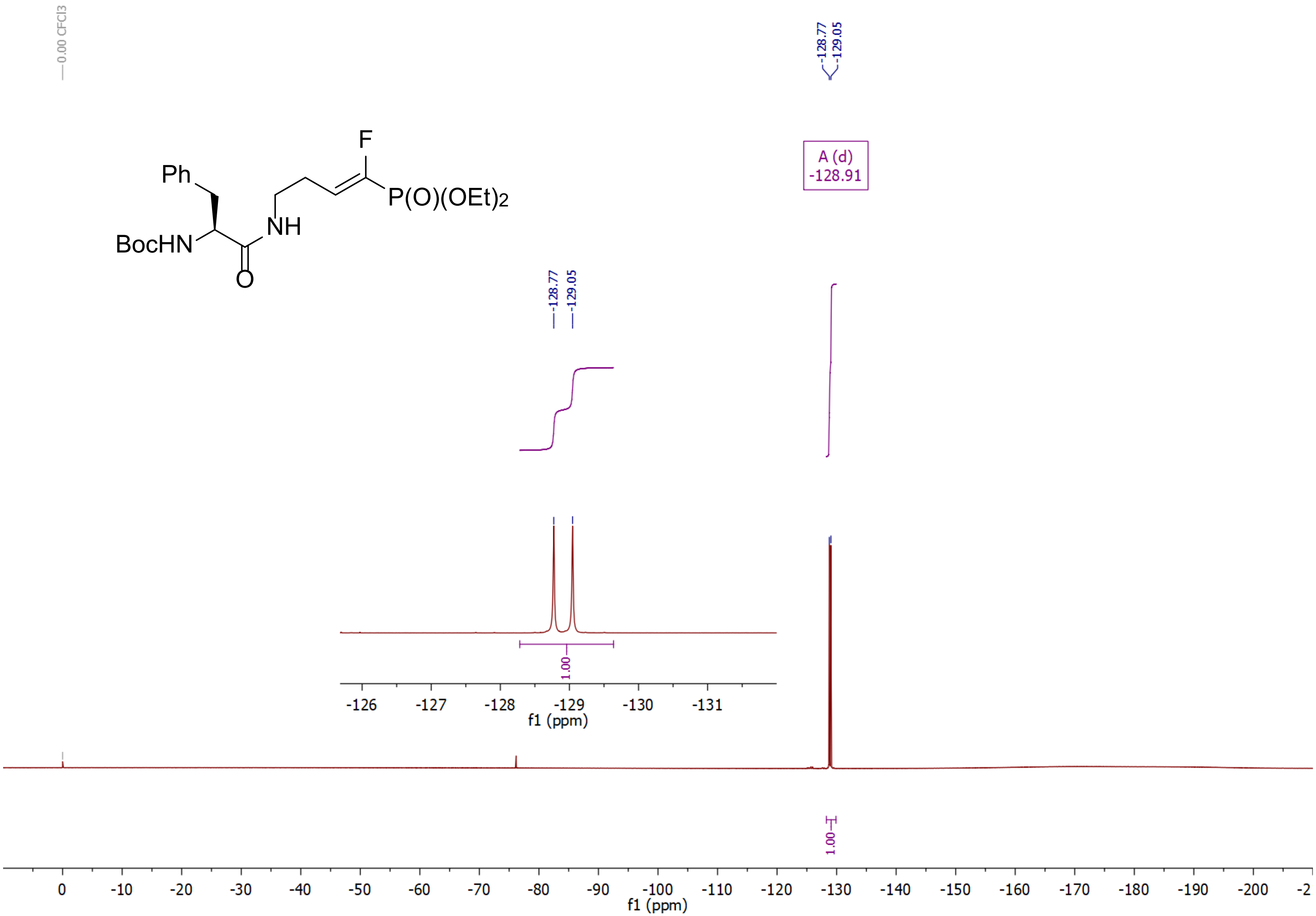

^{19}F{/^{1}H} NMR (377 MHz, Chloroform-*d*) of **14a**.

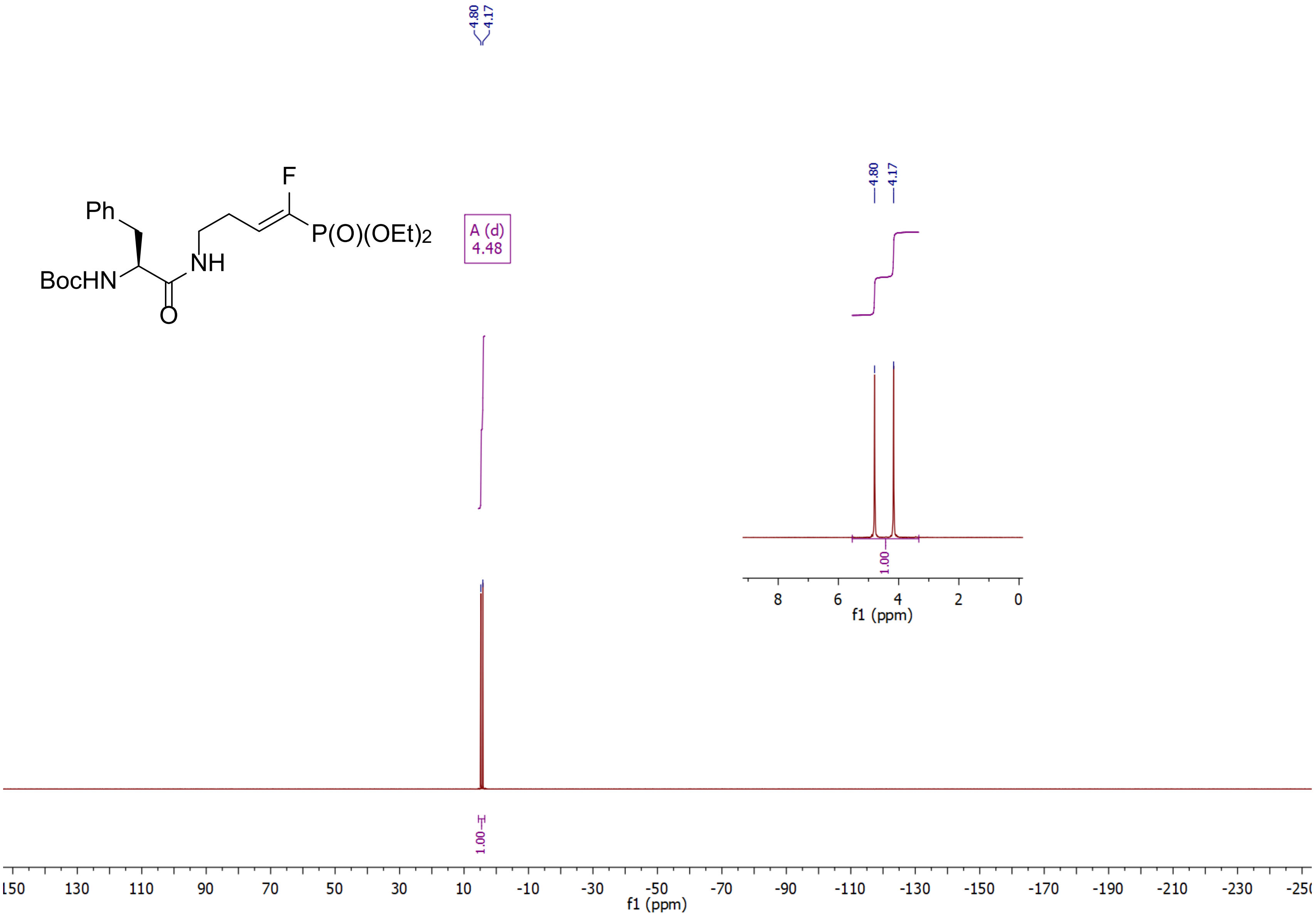

$^{31}P\{/^{1}H\}$ NMR (162 MHz, Chloroform-*d*) of **14a**.

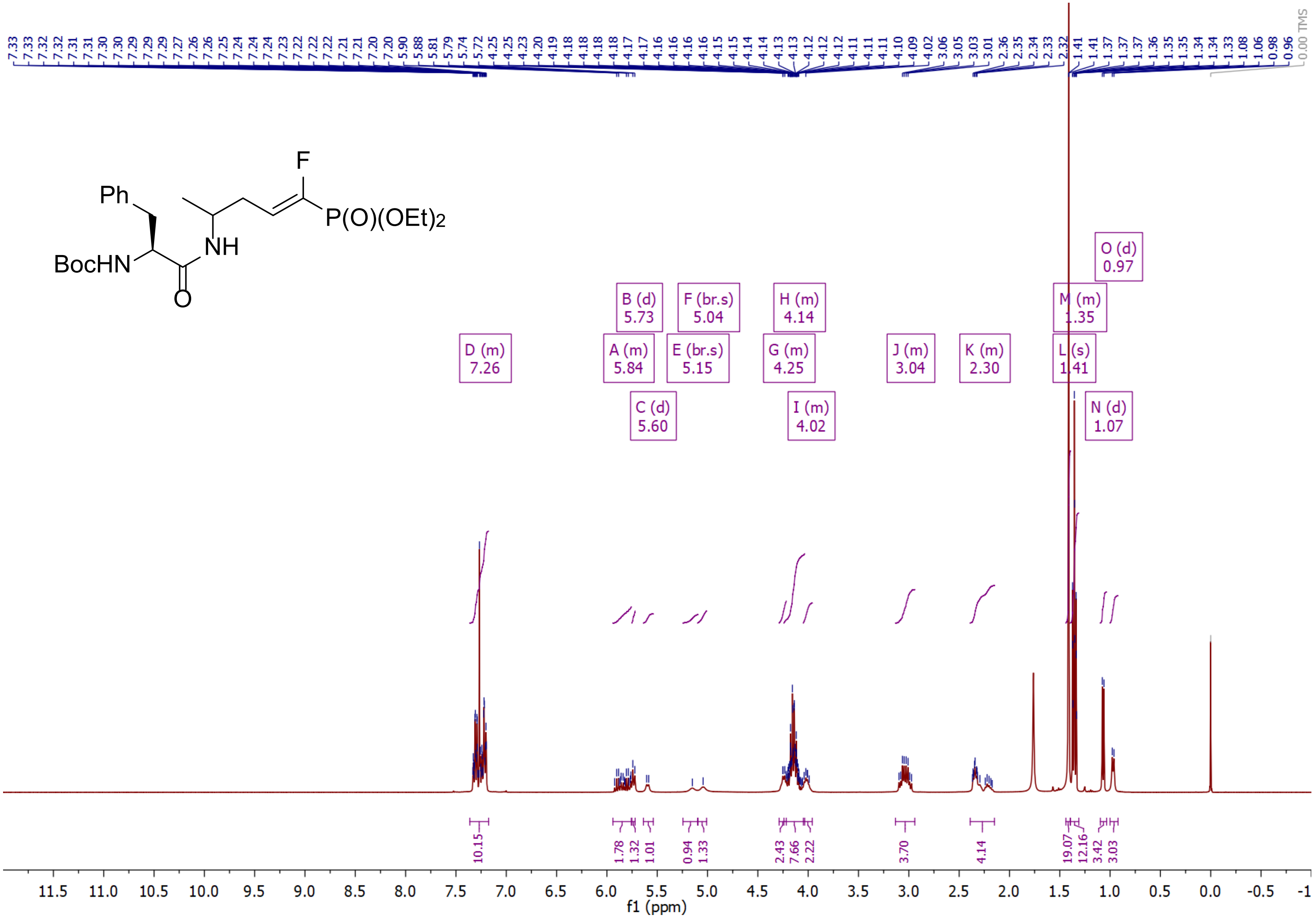

^{1}H NMR (400 MHz, Chloroform-*d*) of **14b**.

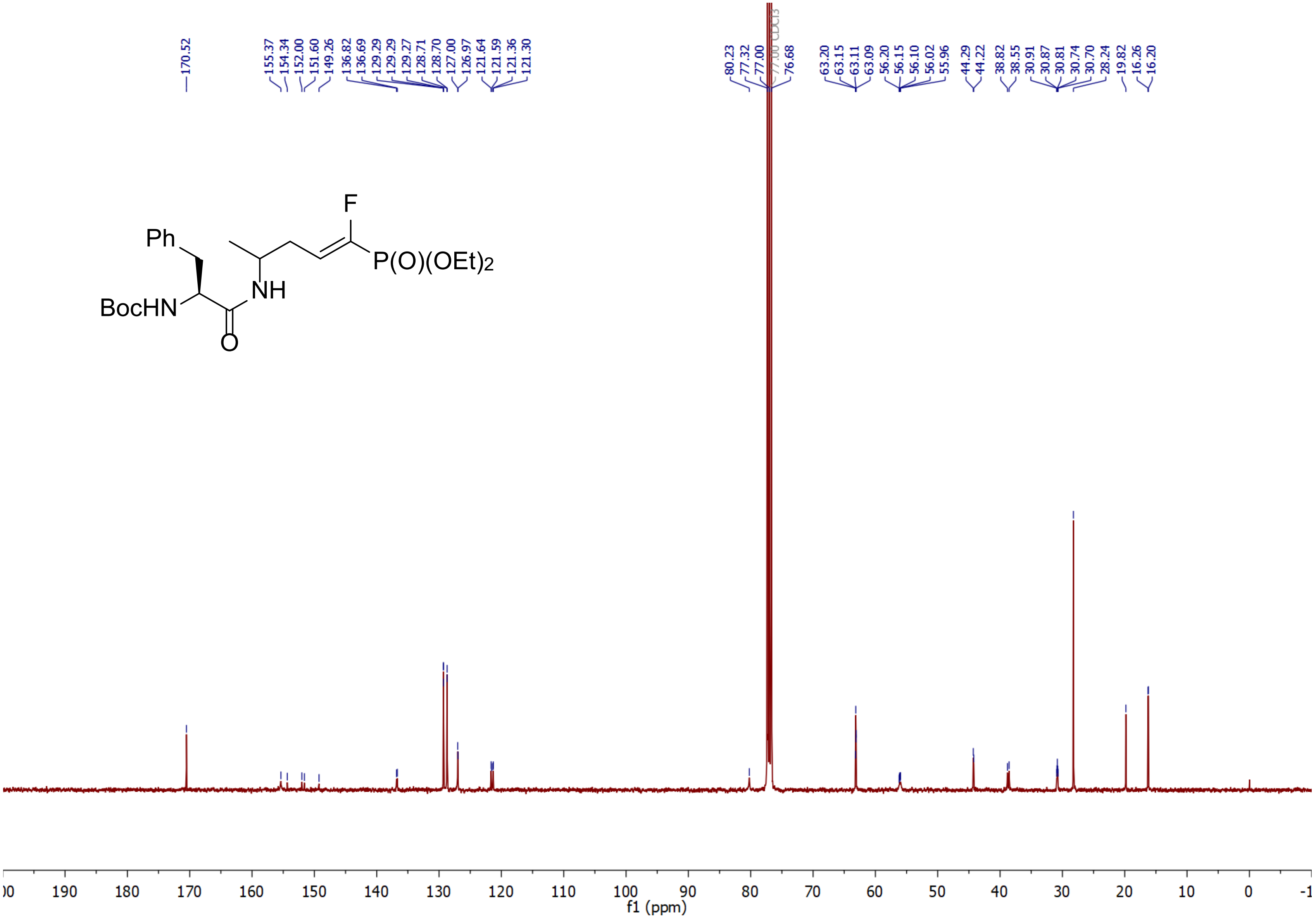

$^{13}C\{/^{1}H\}$ NMR (101 MHz, Chloroform-*d*) of ***rac*-14b**.

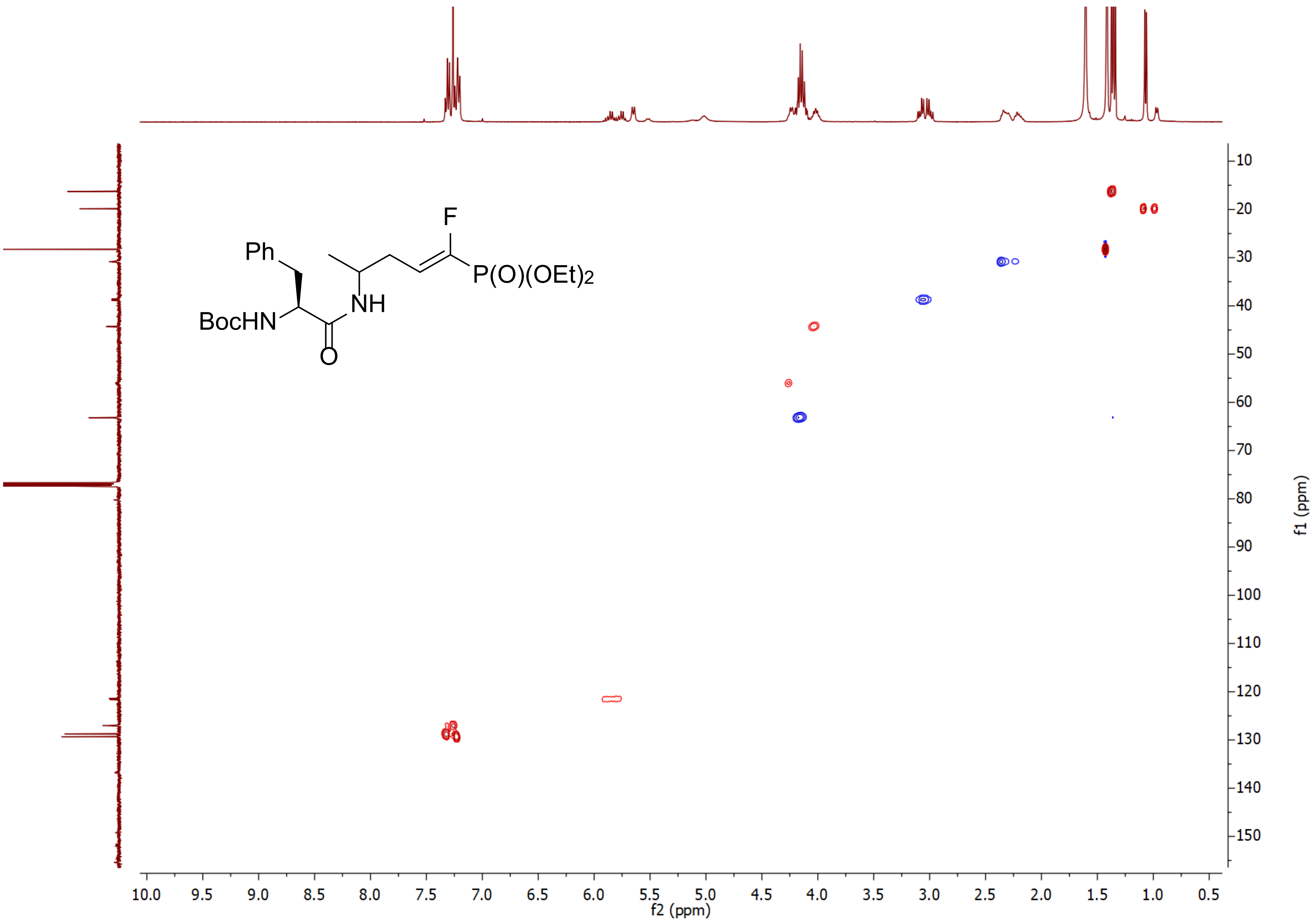

^{13}C{/^{1}H} NMR (400 MHz /101 MHz, 101 MHz, Chloroform-*d*) of ***rac*-14b**.

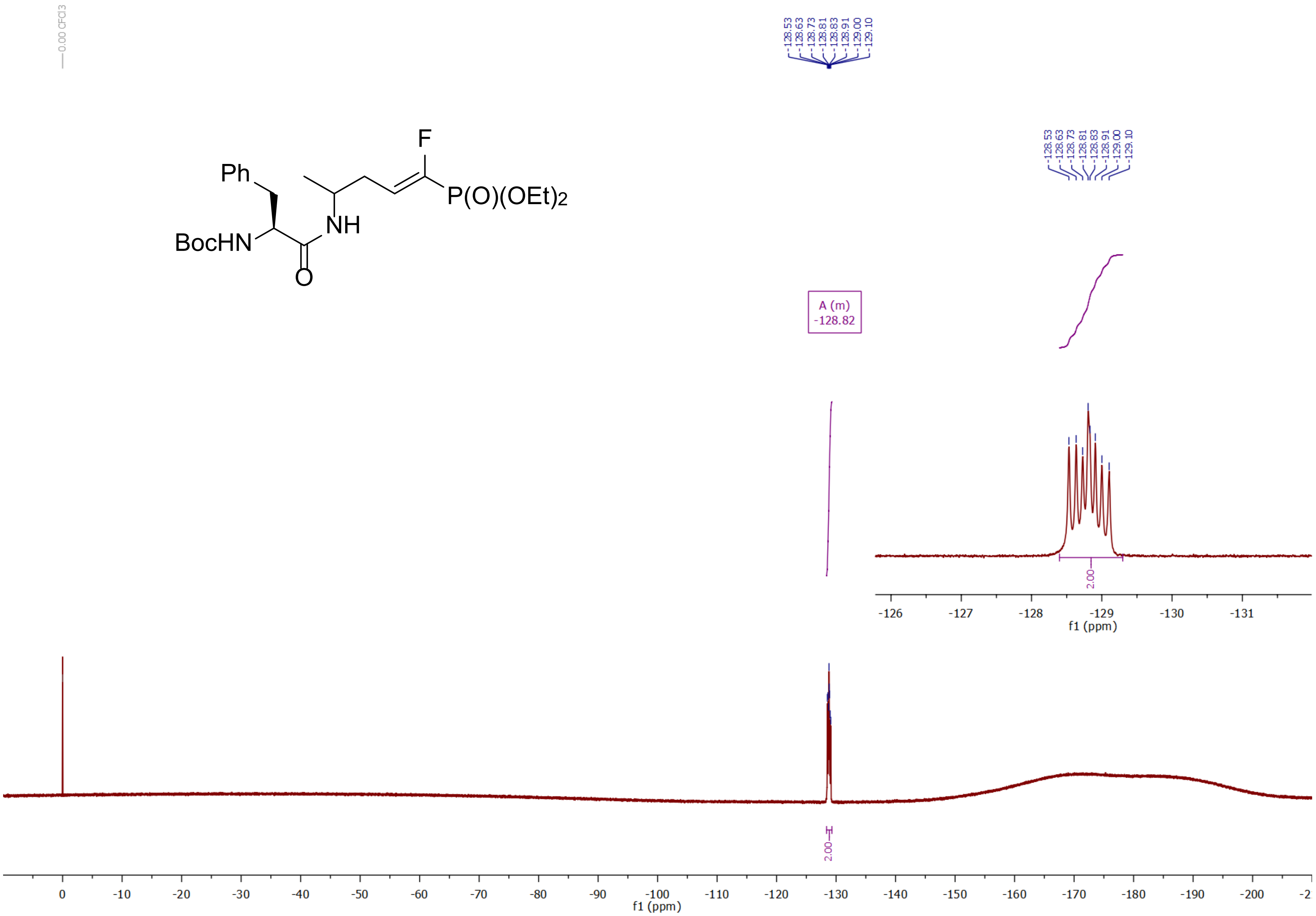

^{19}F NMR (377 MHz, Chloroform-*d*) of ***rac*-14b**.

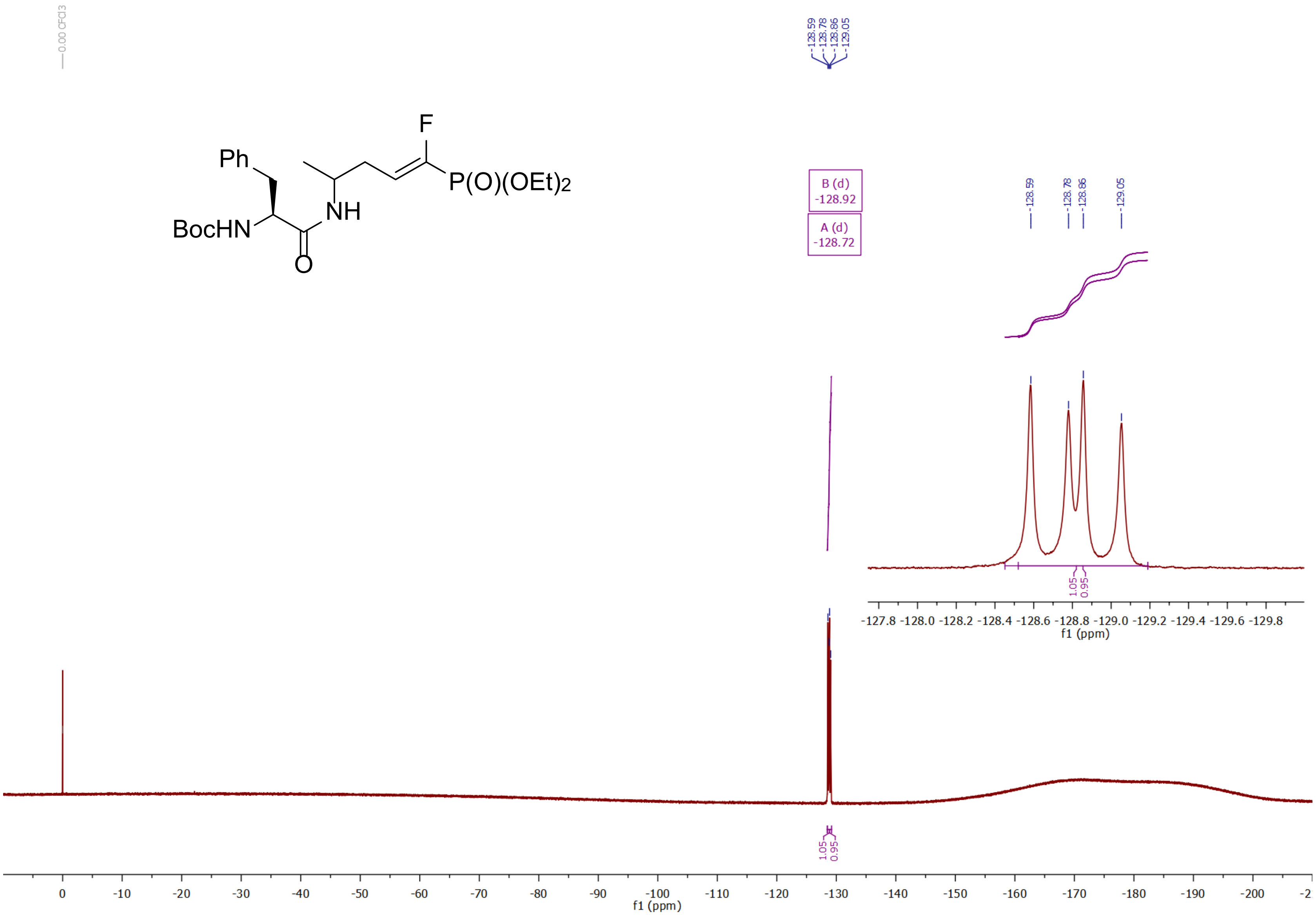

^{19}F{/^{1}H} NMR (377 MHz, Chloroform-*d*) of ***rac*-14b**.

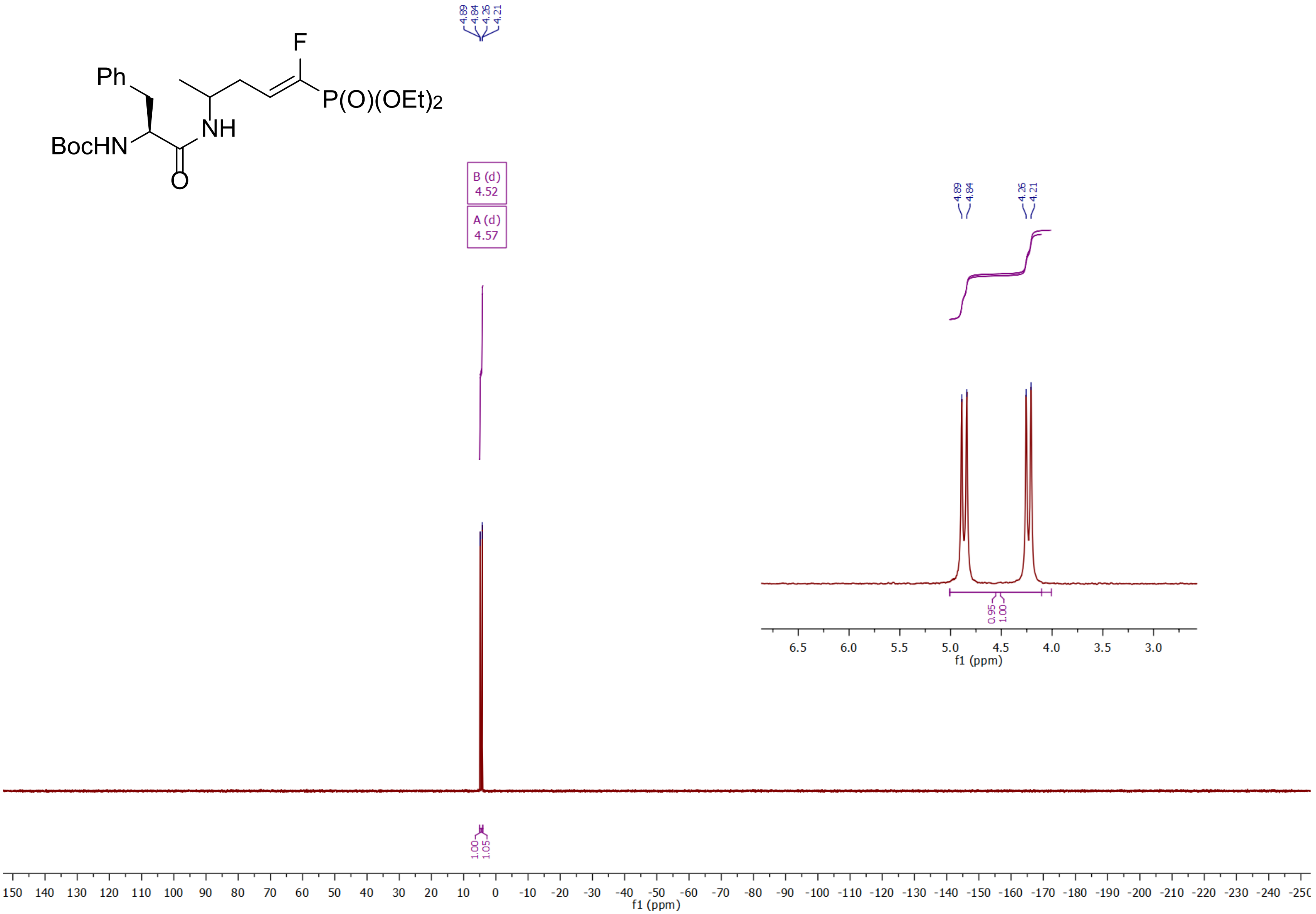

$^{31}P\{/^{1}H\}$ NMR (162 MHz, Chloroform-*d*) of ***rac*-14b**.

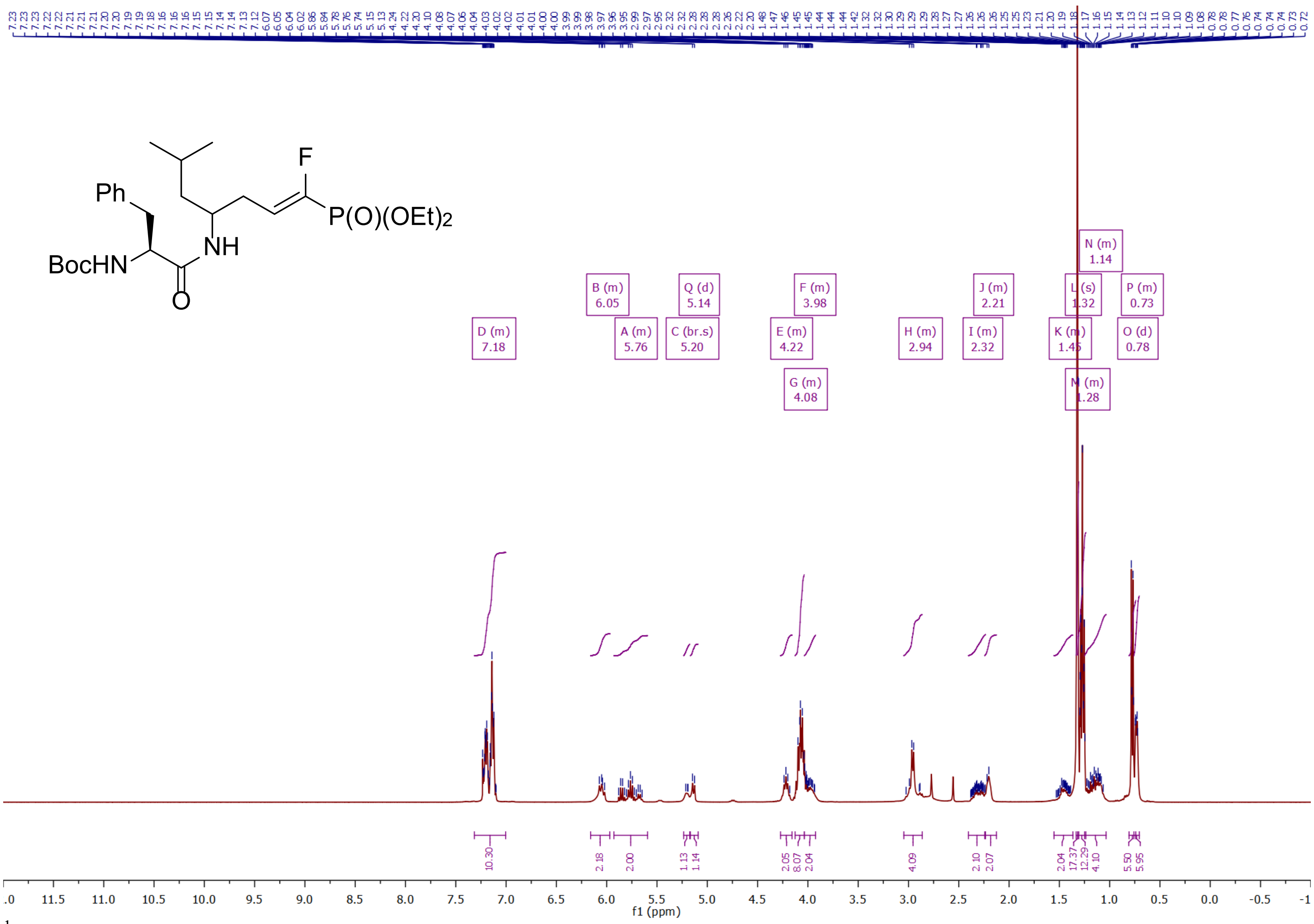

^{1}H NMR (400 MHz, Chloroform-*d*) of ***rac*-14c**.

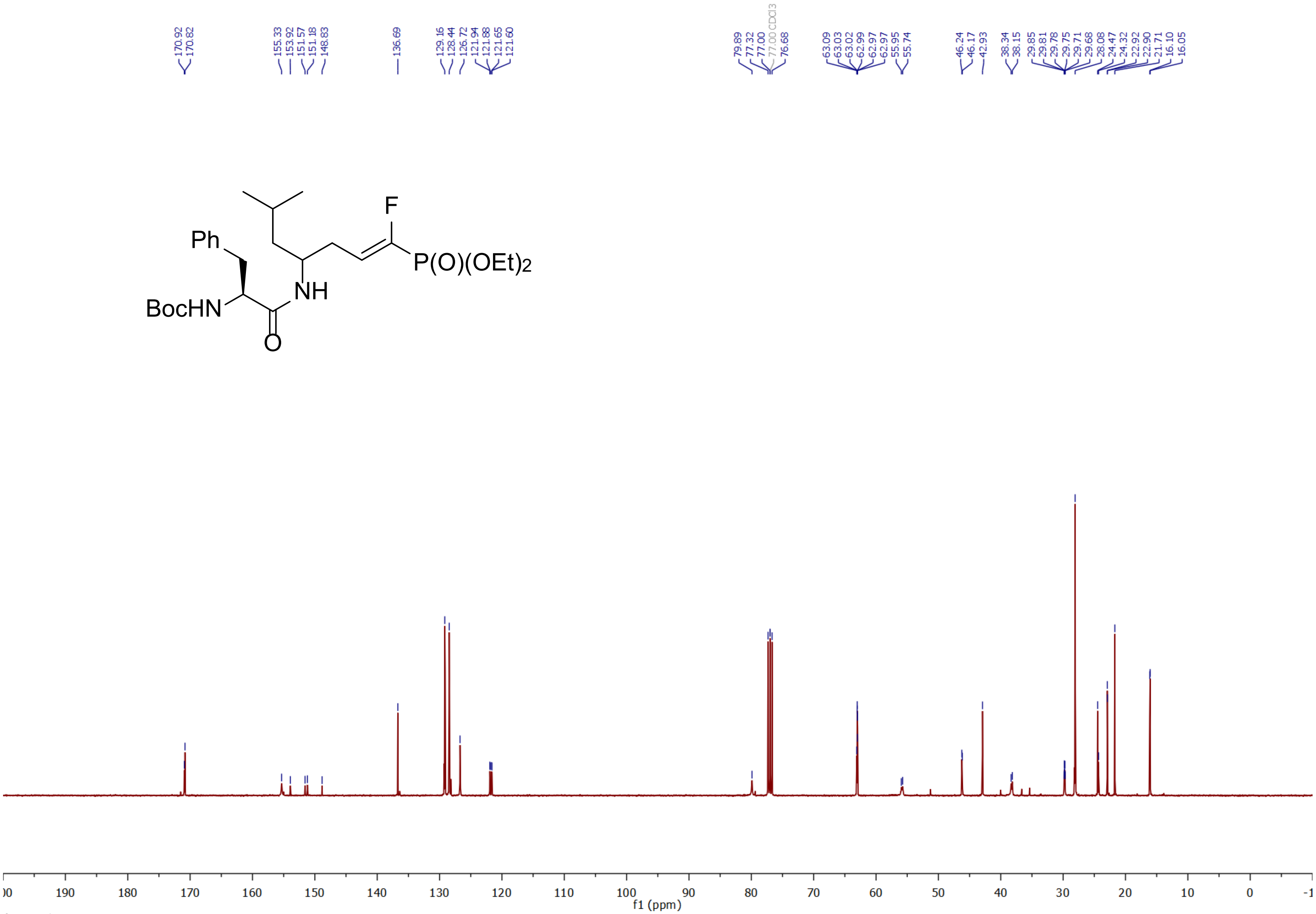

^{3}C{/^{1}H} NMR (101 MHz, Chloroform-*d*) of ***rac*-14c**.

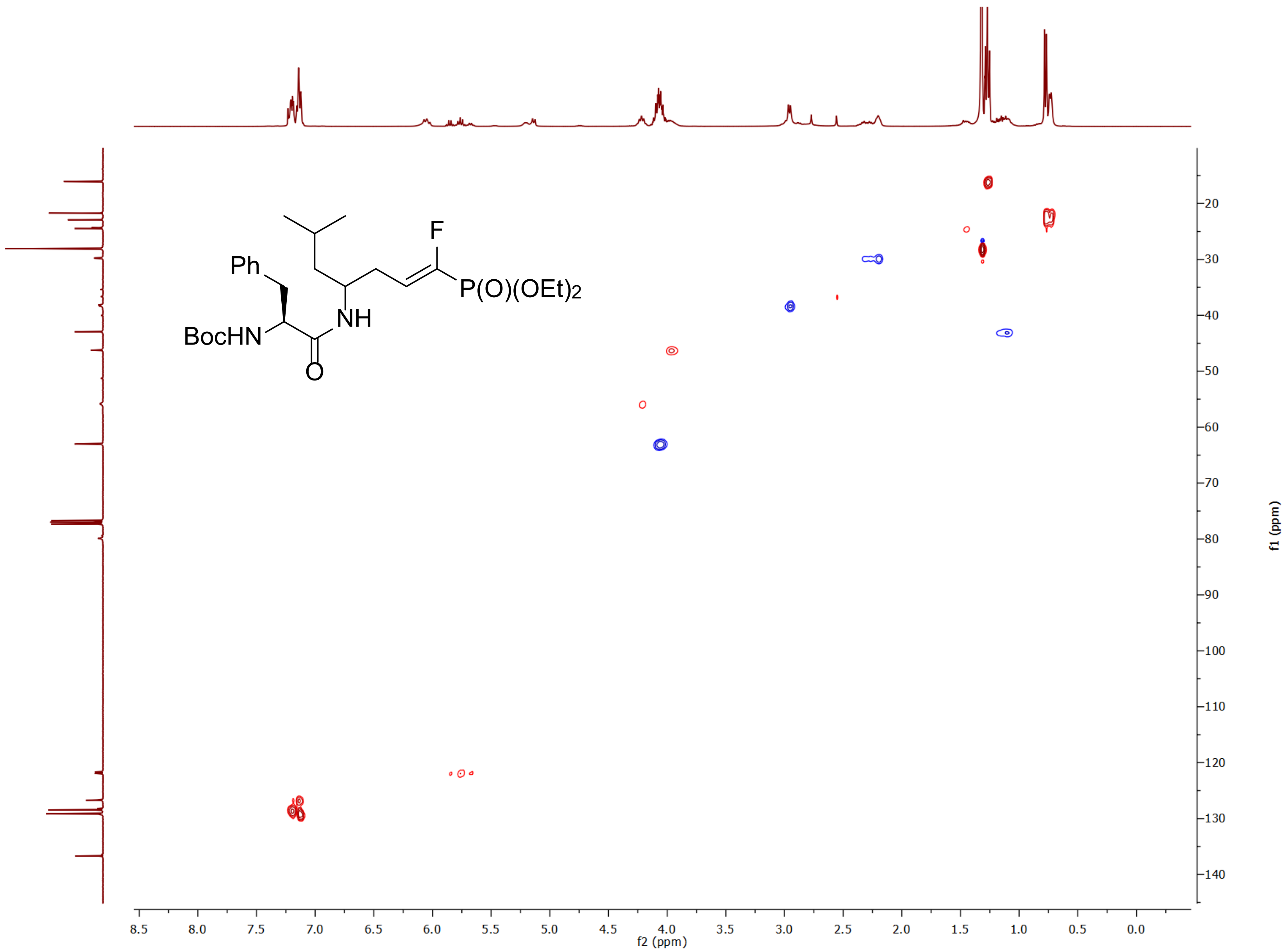

^{1}H-^{13}C HSQC (400 MHz /101 MHz, Chloroform-*d*) of ***rac*-14c**.

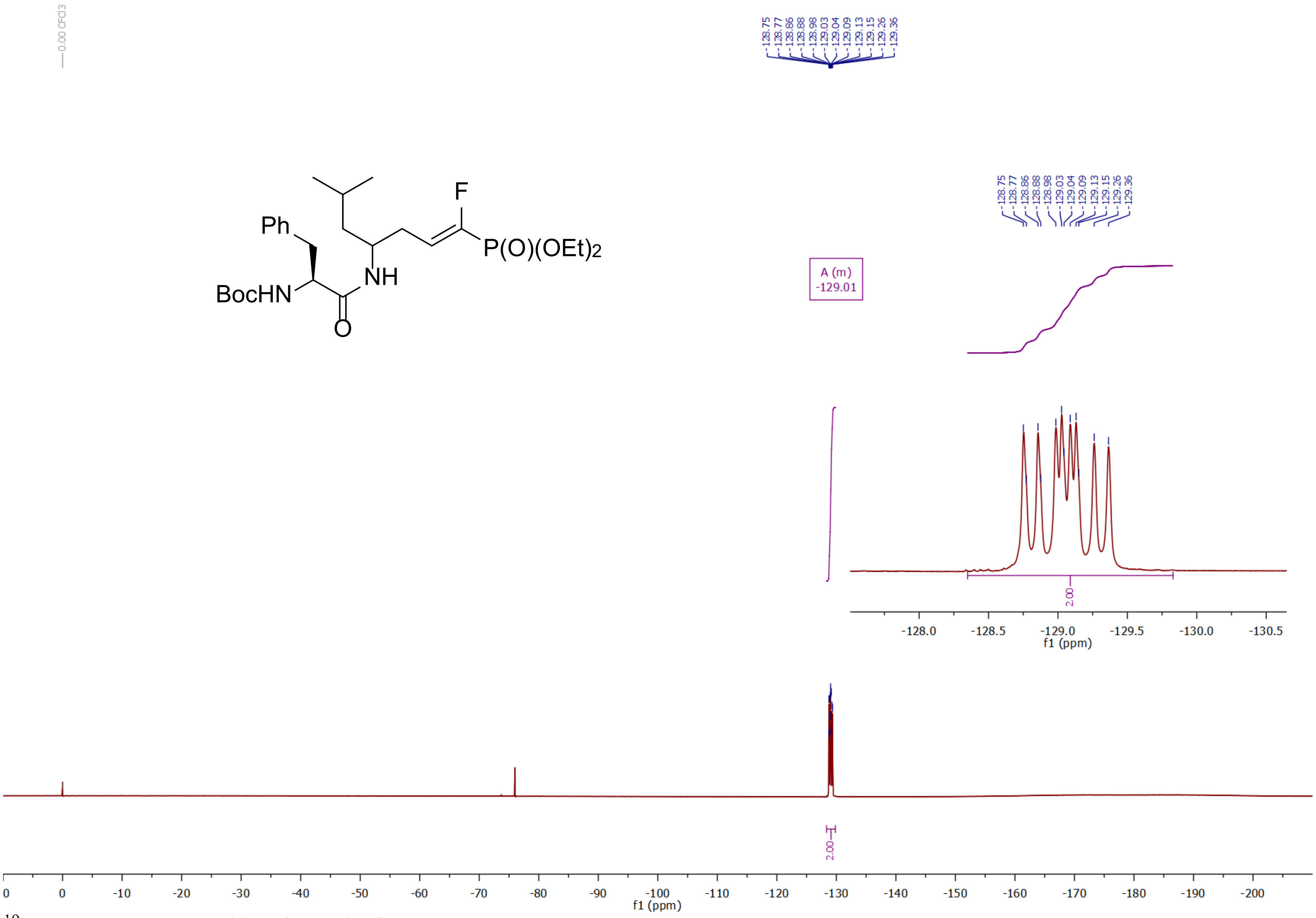

^{19}F NMR (377 MHz, Chloroform-*d*) of ***rac*-14c**.

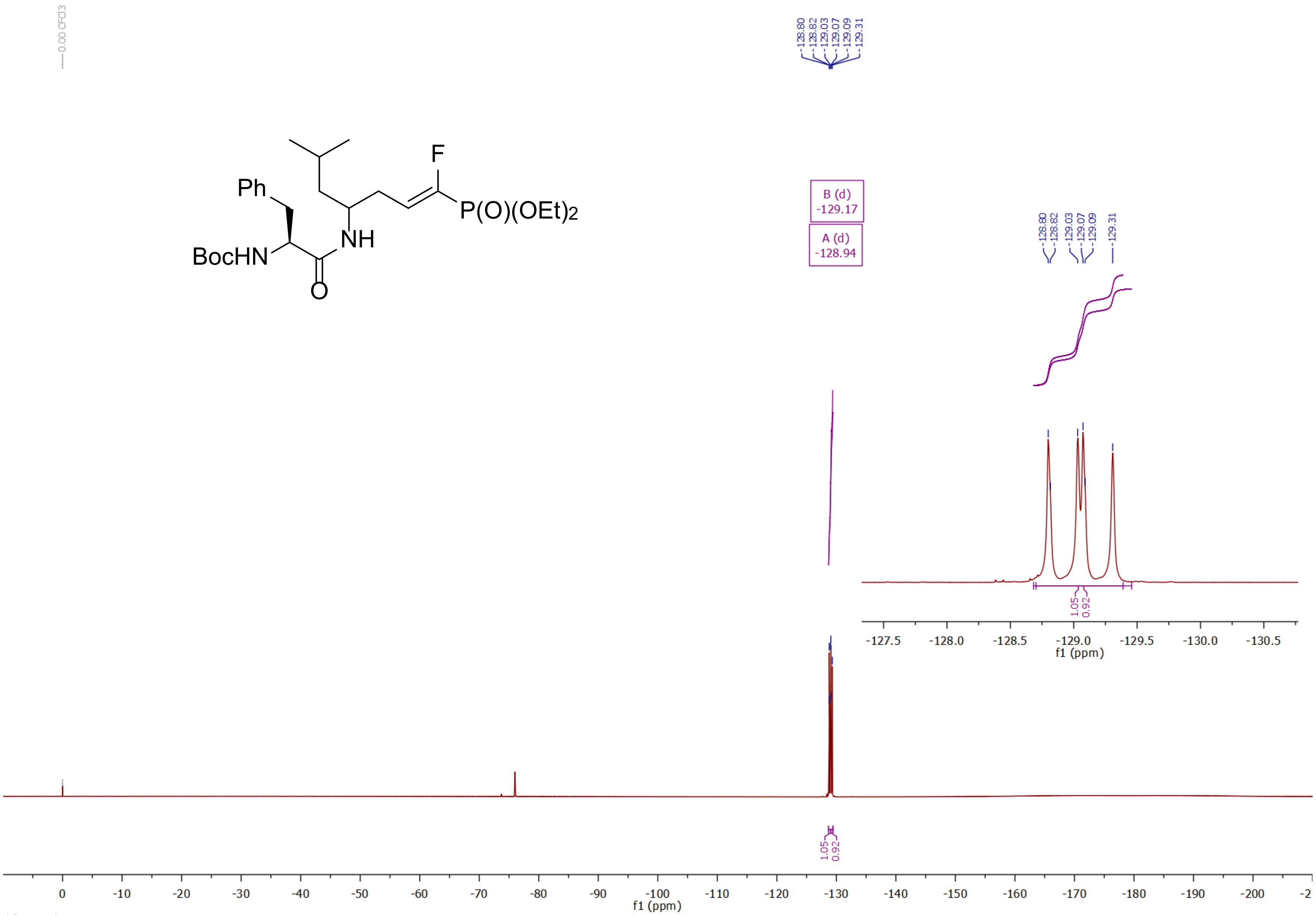

$^{19}F\{/^{1}H\}$ NMR (377 MHz, Chloroform-*d*) of ***rac*-14c**.

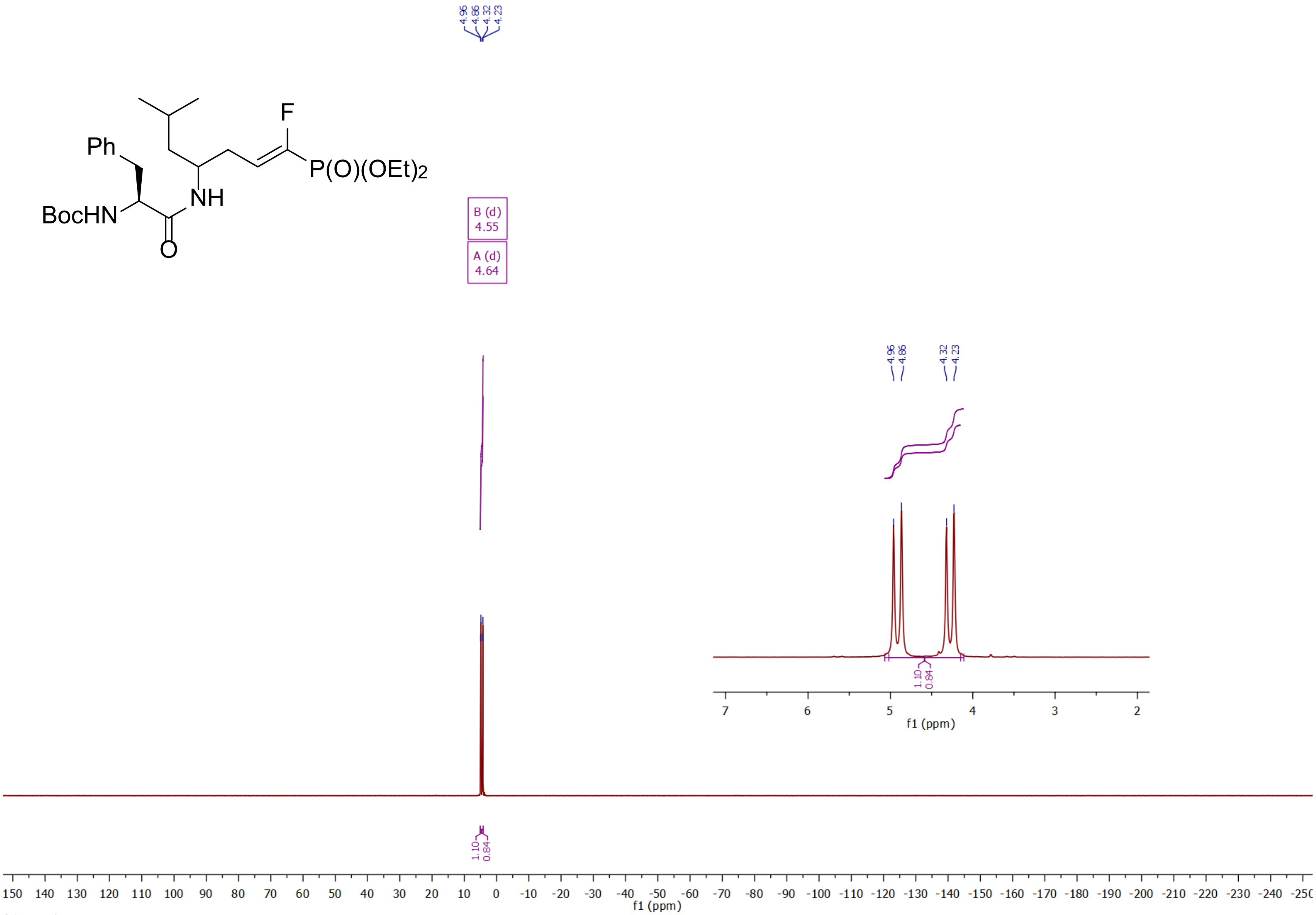

$^{31}P\{/^{1}H\}$ NMR (162 MHz, Chloroform-*d*) of ***rac*-14c**.

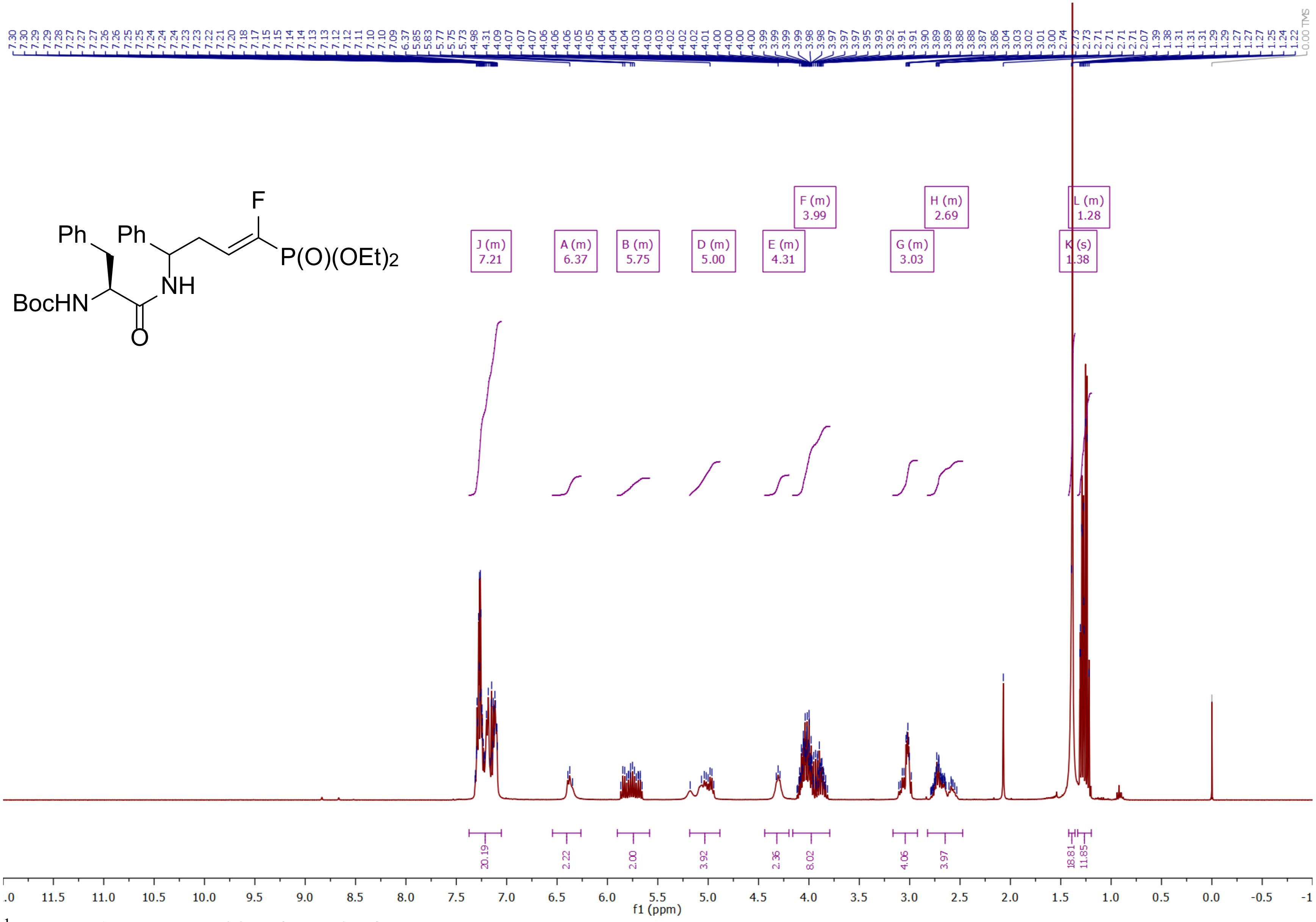

^{1}H NMR (400 MHz, Chloroform-*d*) of ***rac*-14d**.

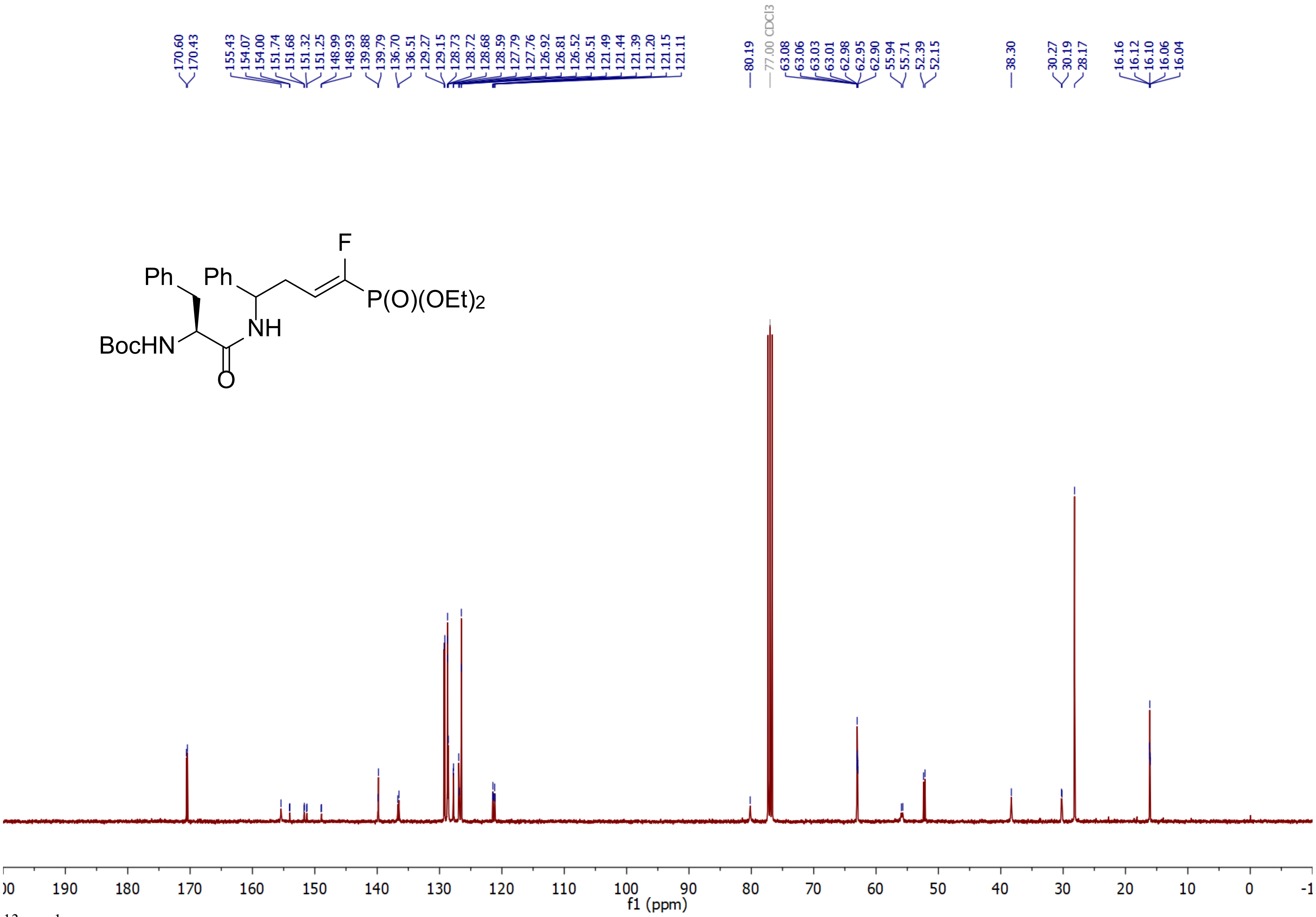

$^{13}C\{/^{1}H\}$ NMR (101 MHz, Chloroform-*d*) of ***rac*-14d**.

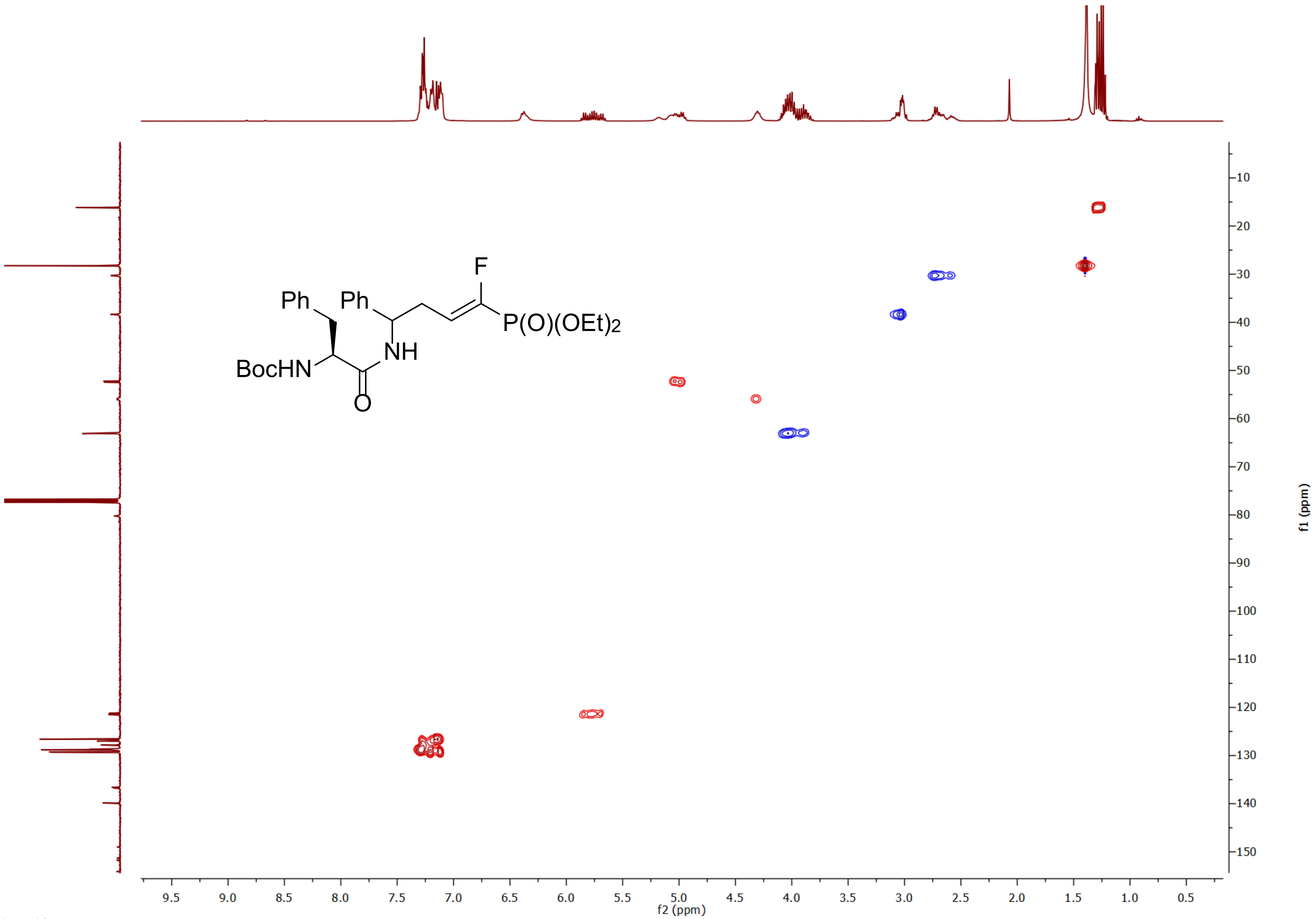

^{1}H-^{13}C HSQC (400 MHz /101 MHz, Chloroform-*d*) of ***rac*-14d**.

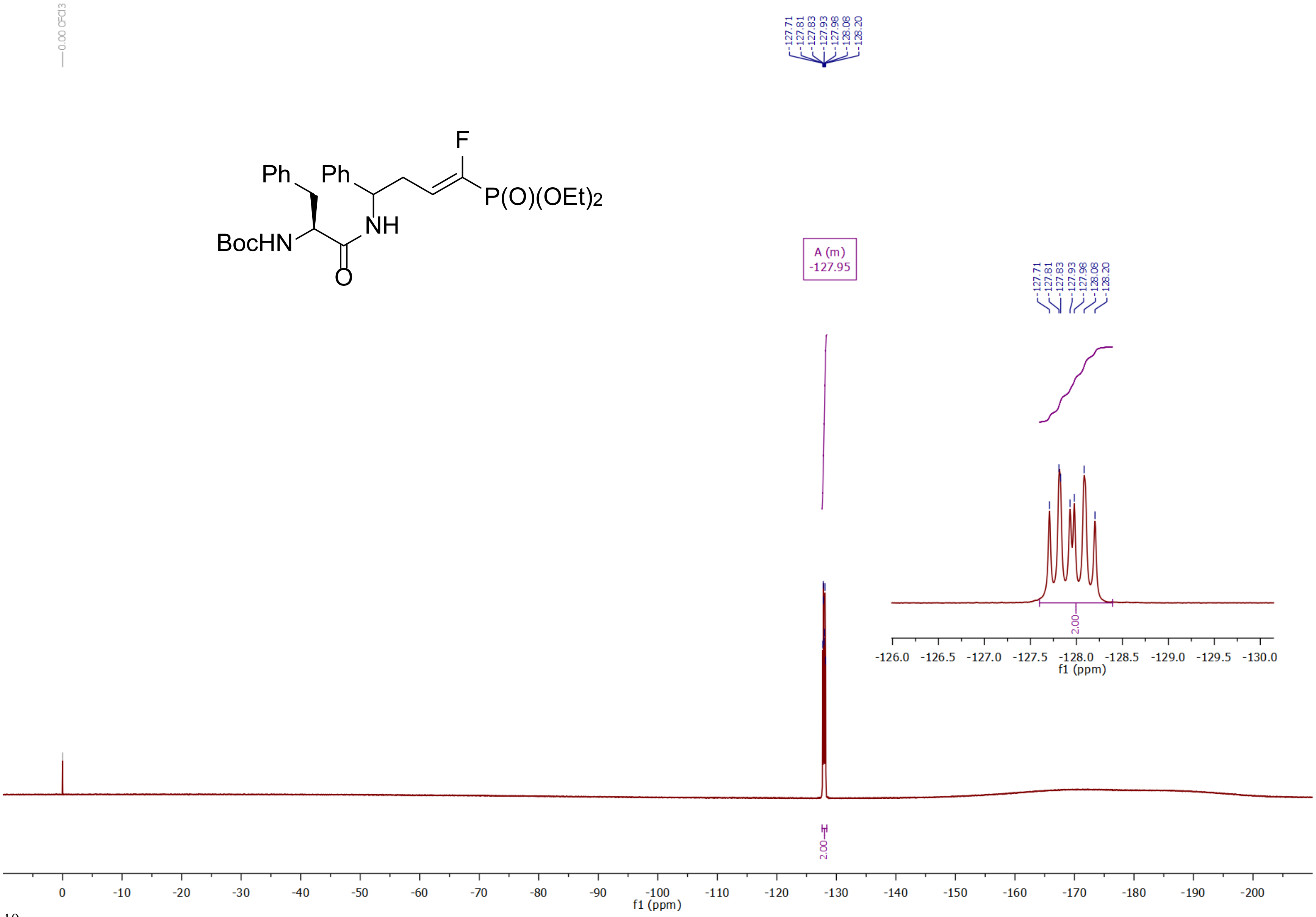

^{19}F NMR (377 MHz, Chloroform-*d*) of ***rac*-14d**.

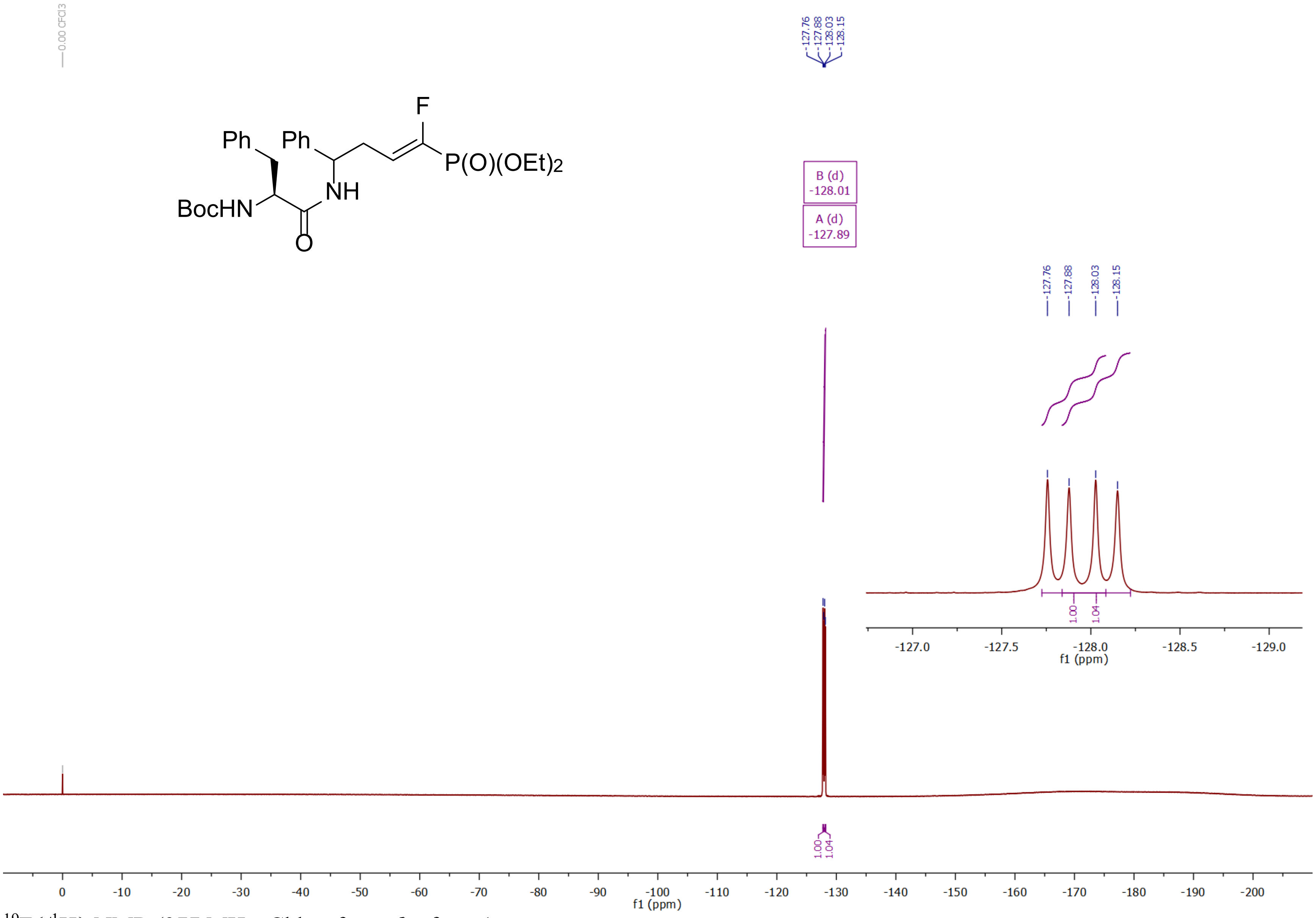

$^{19}F\{/^{1}H\}$ NMR (377 MHz, Chloroform-*d*) of ***rac*-14d**.

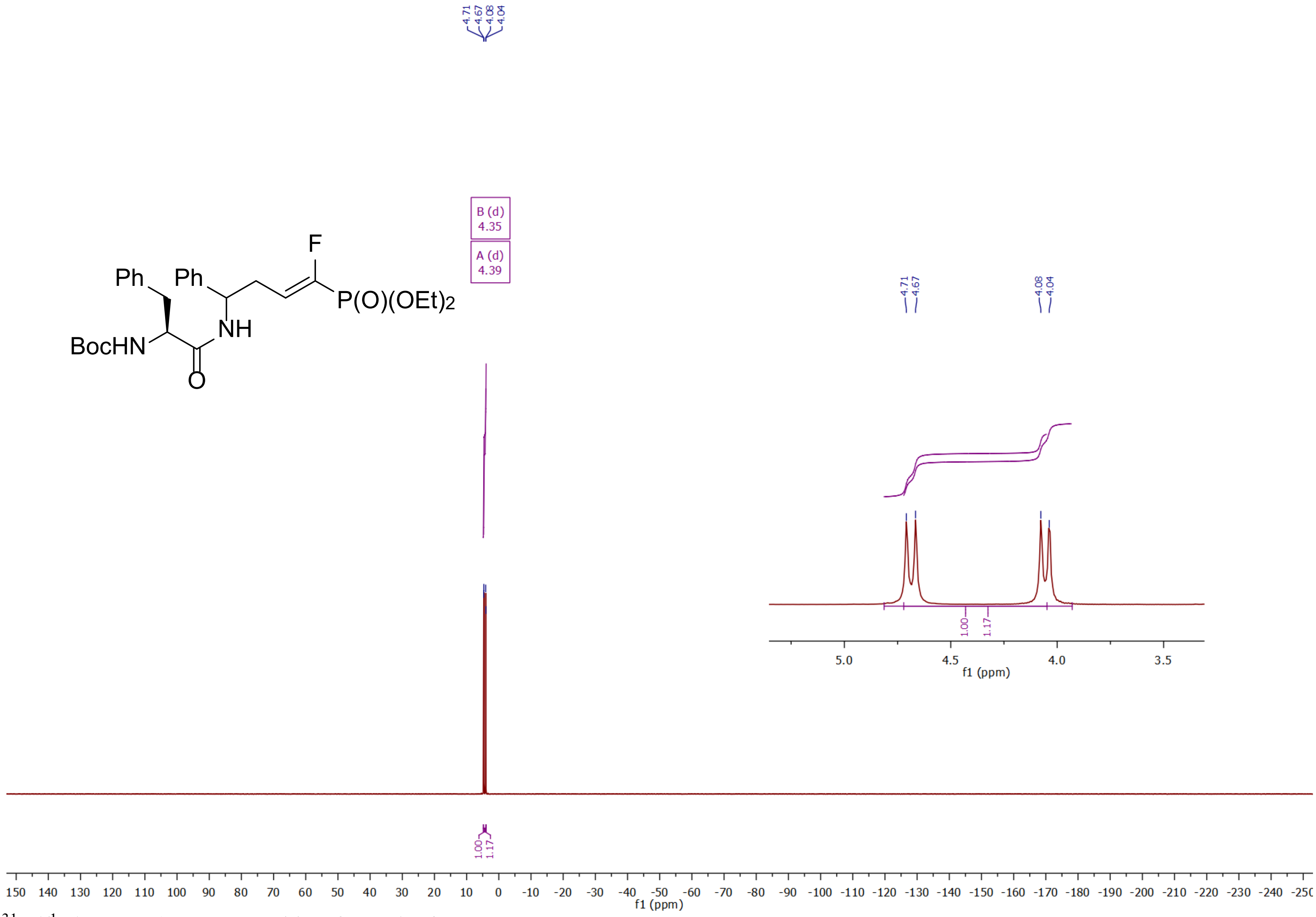

^{31}P{/^{1}H} NMR (162 MHz, Chloroform-*d*) of ***rac*-14d**.

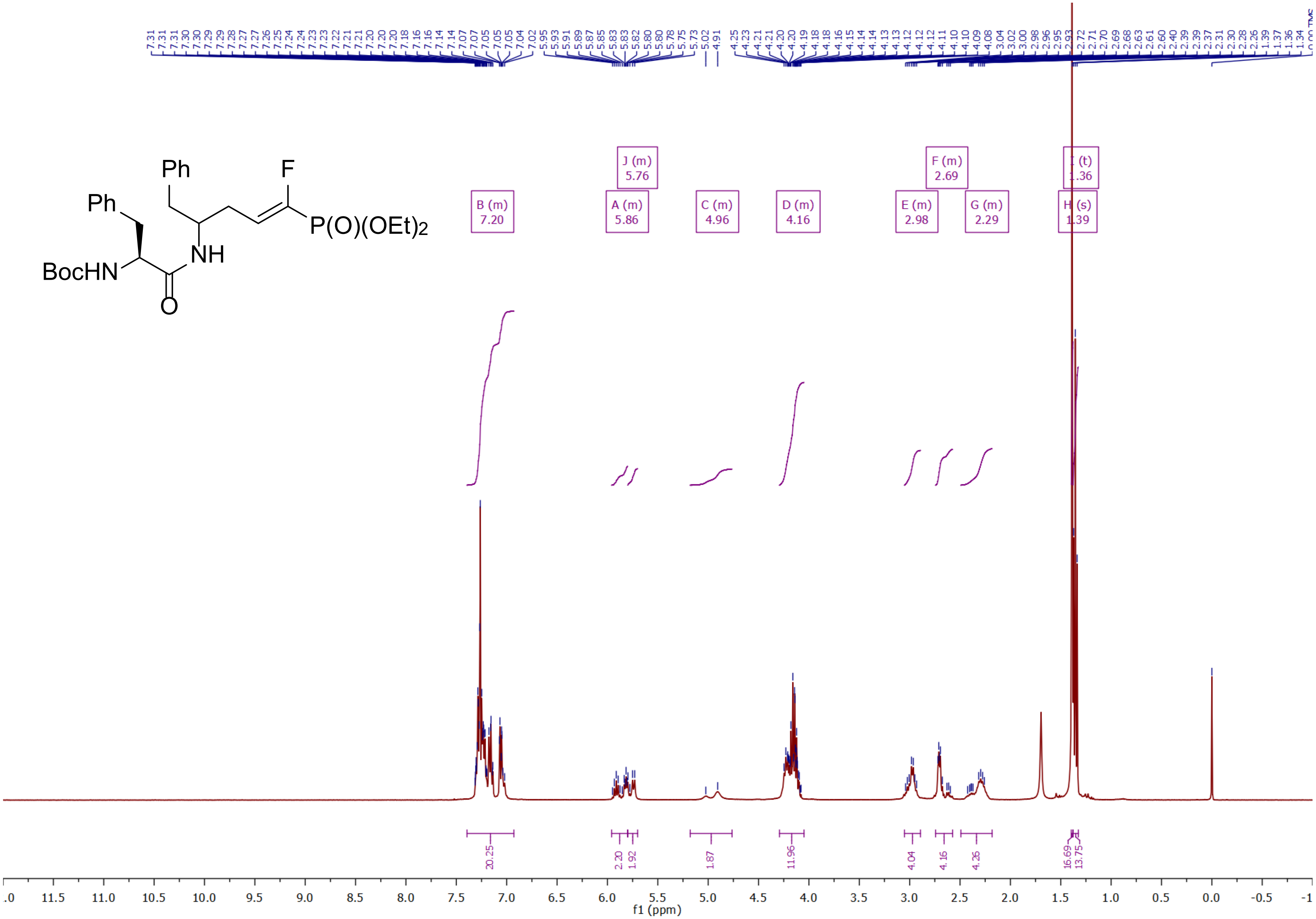

^{1}H NMR (400 MHz, Chloroform-*d*) of ***rac*-14e**.

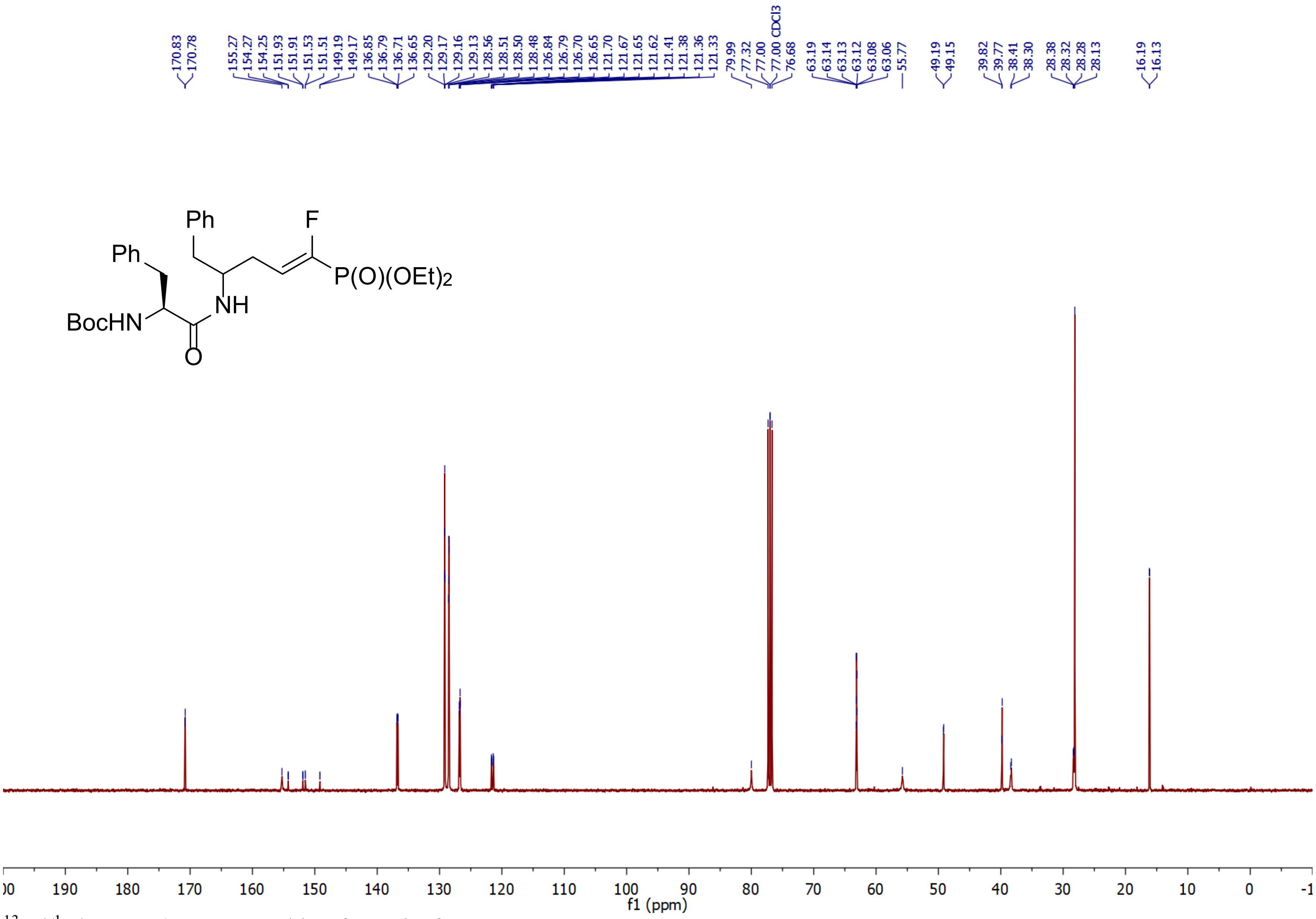

$^{13}C\{/^{1}H\}$ NMR (101 MHz, Chloroform-*d*) of ***rac*-14e**.

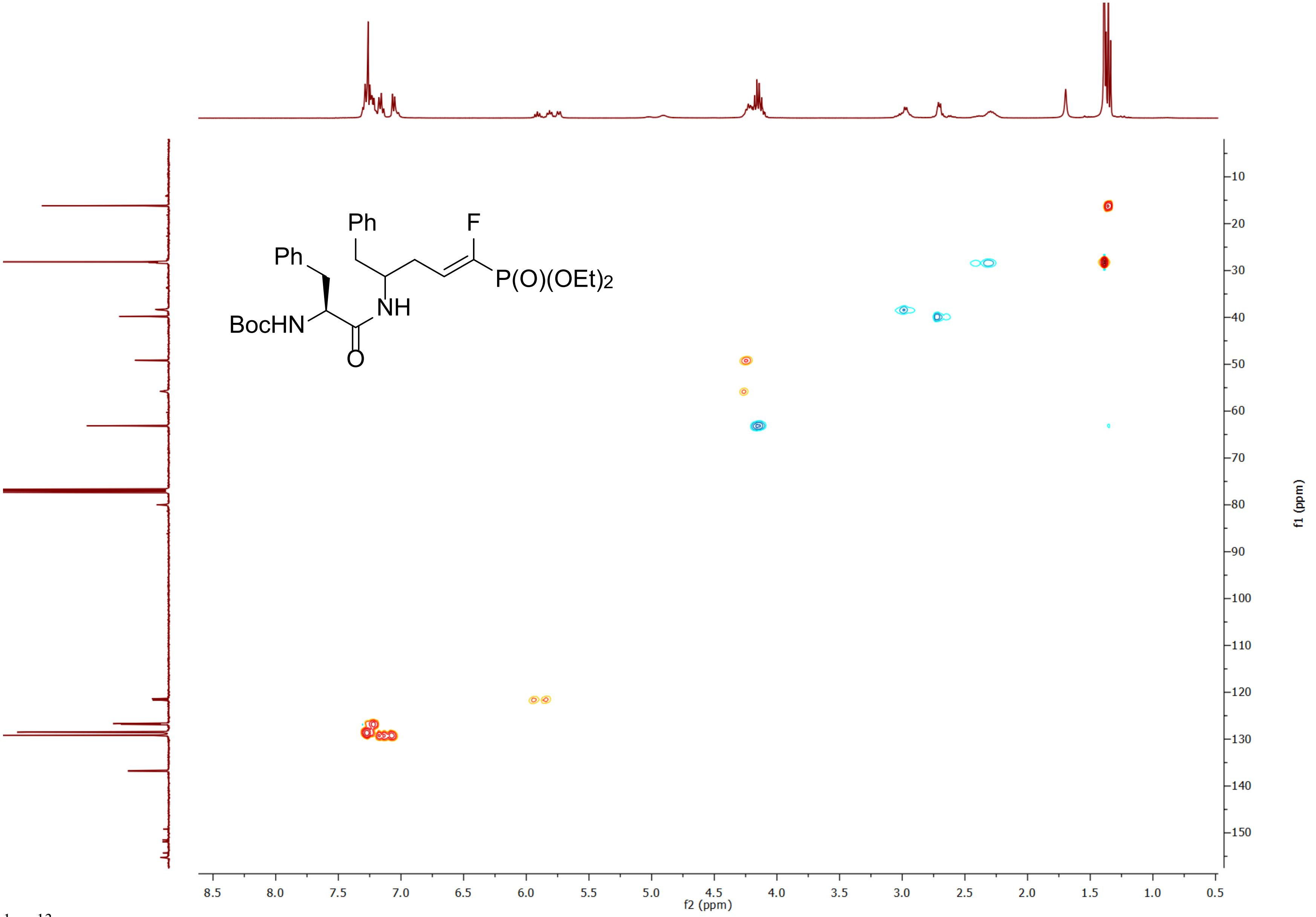

^1H-^{13}C HSQC (400 MHz /101 MHz, Chloroform-*d*) of ***rac*-14e**.

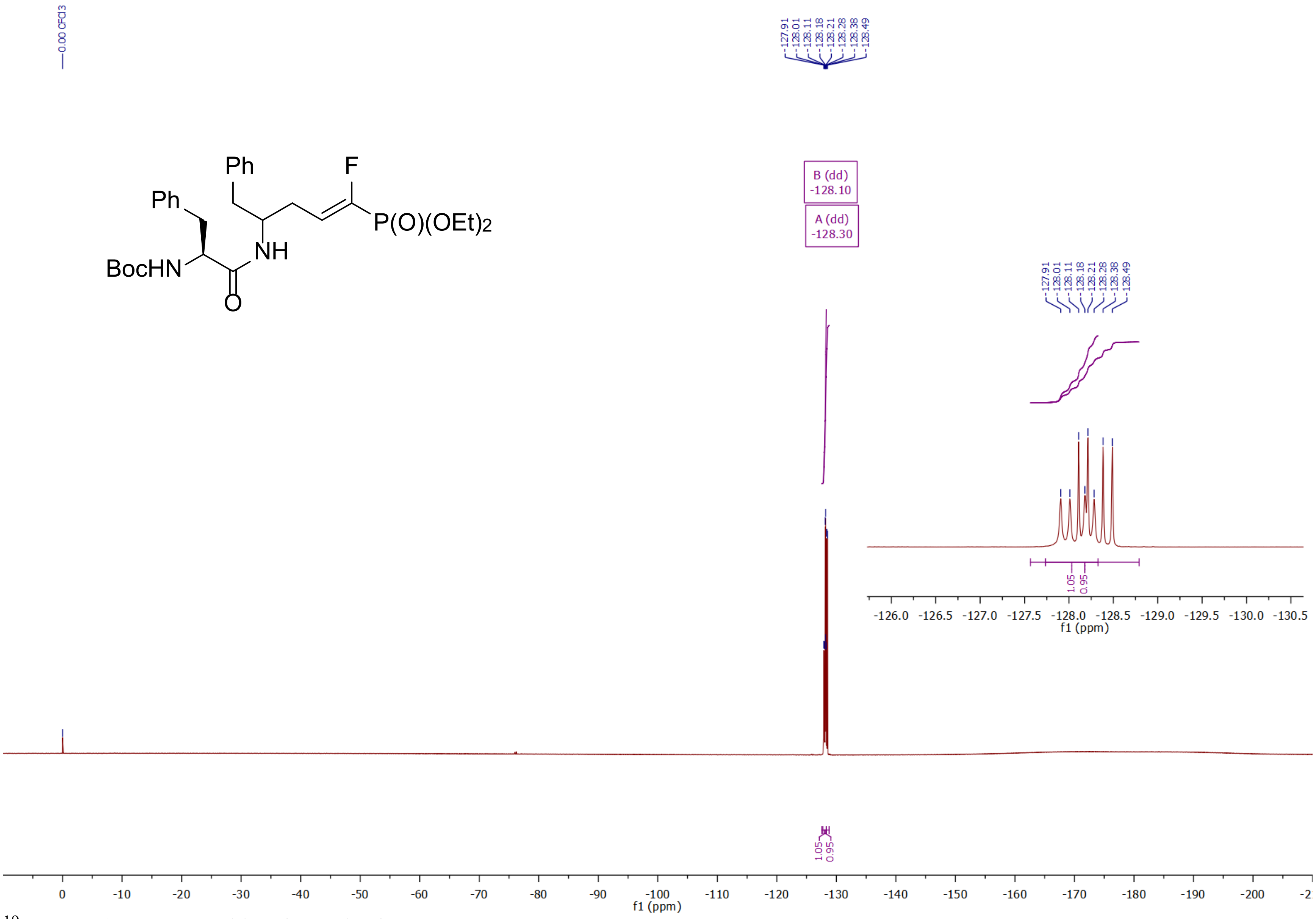

^{19}F NMR (377 MHz, Chloroform-*d*) of ***rac*-14e**.

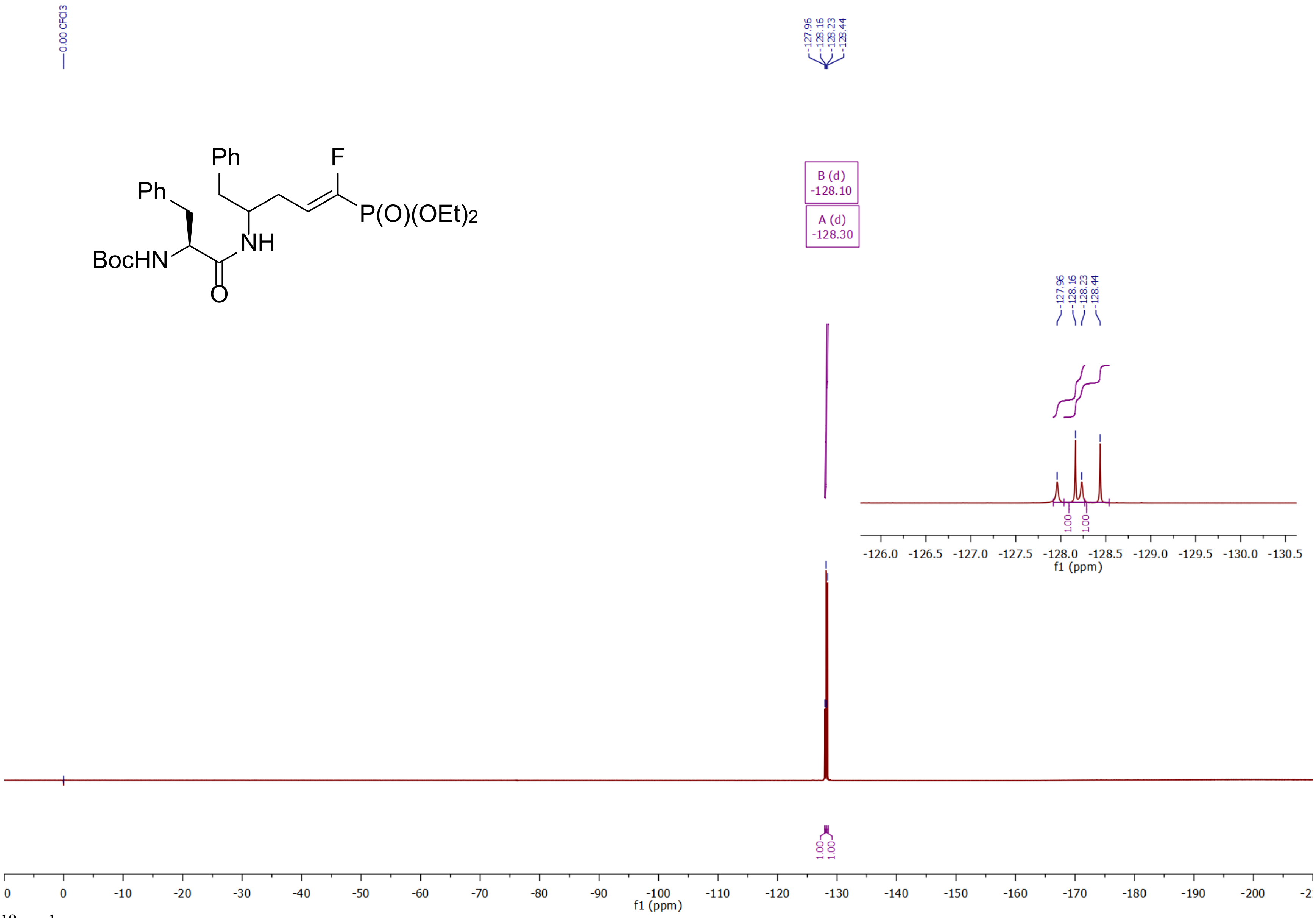

$^{19}F\{/^{1}H\}$ NMR (377 MHz, Chloroform-*d*) of ***rac*-14e**.

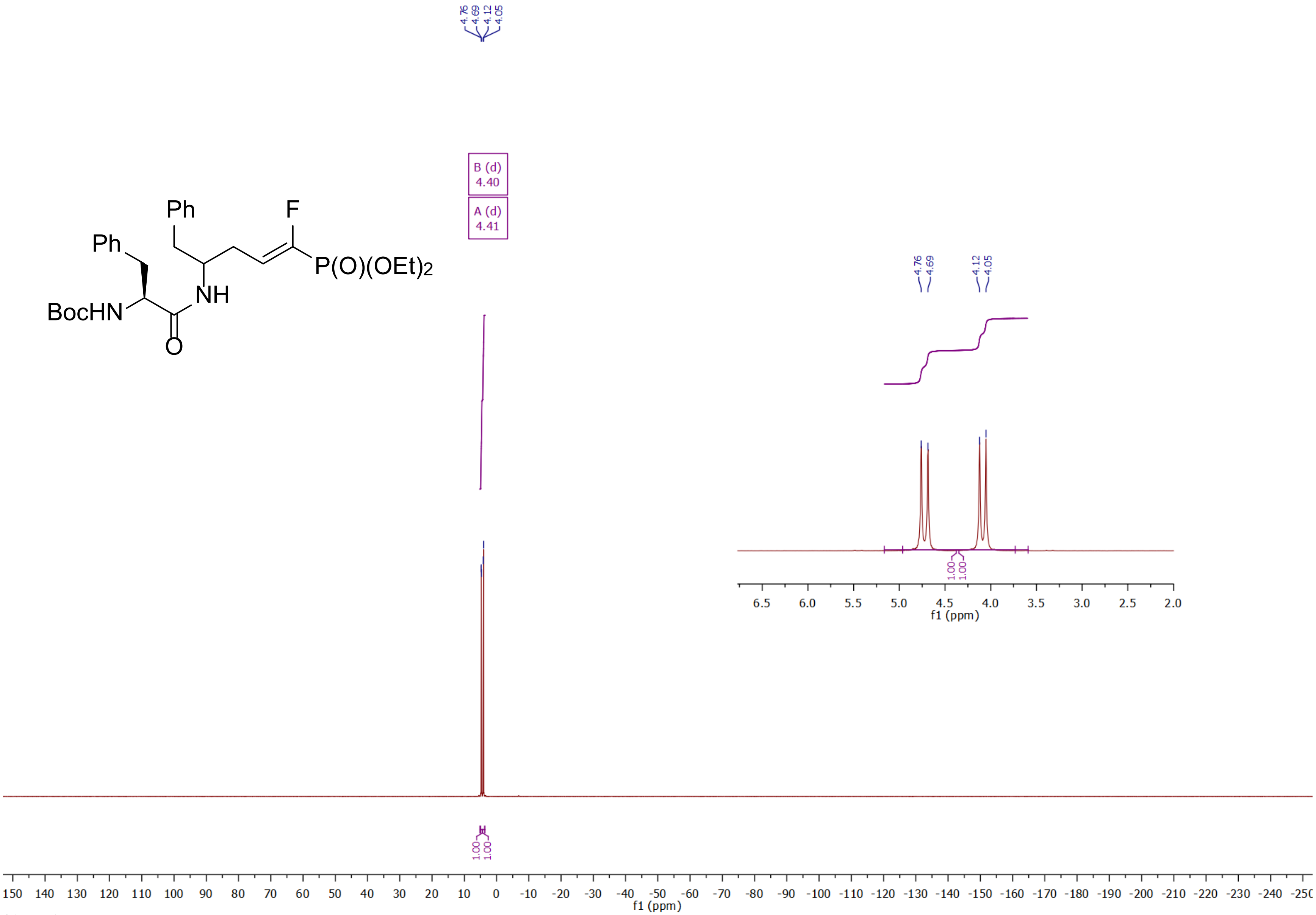

$^{31}P\{/^{1}H\}$ NMR (162 MHz, Chloroform-*d*) of ***rac*-14e**.

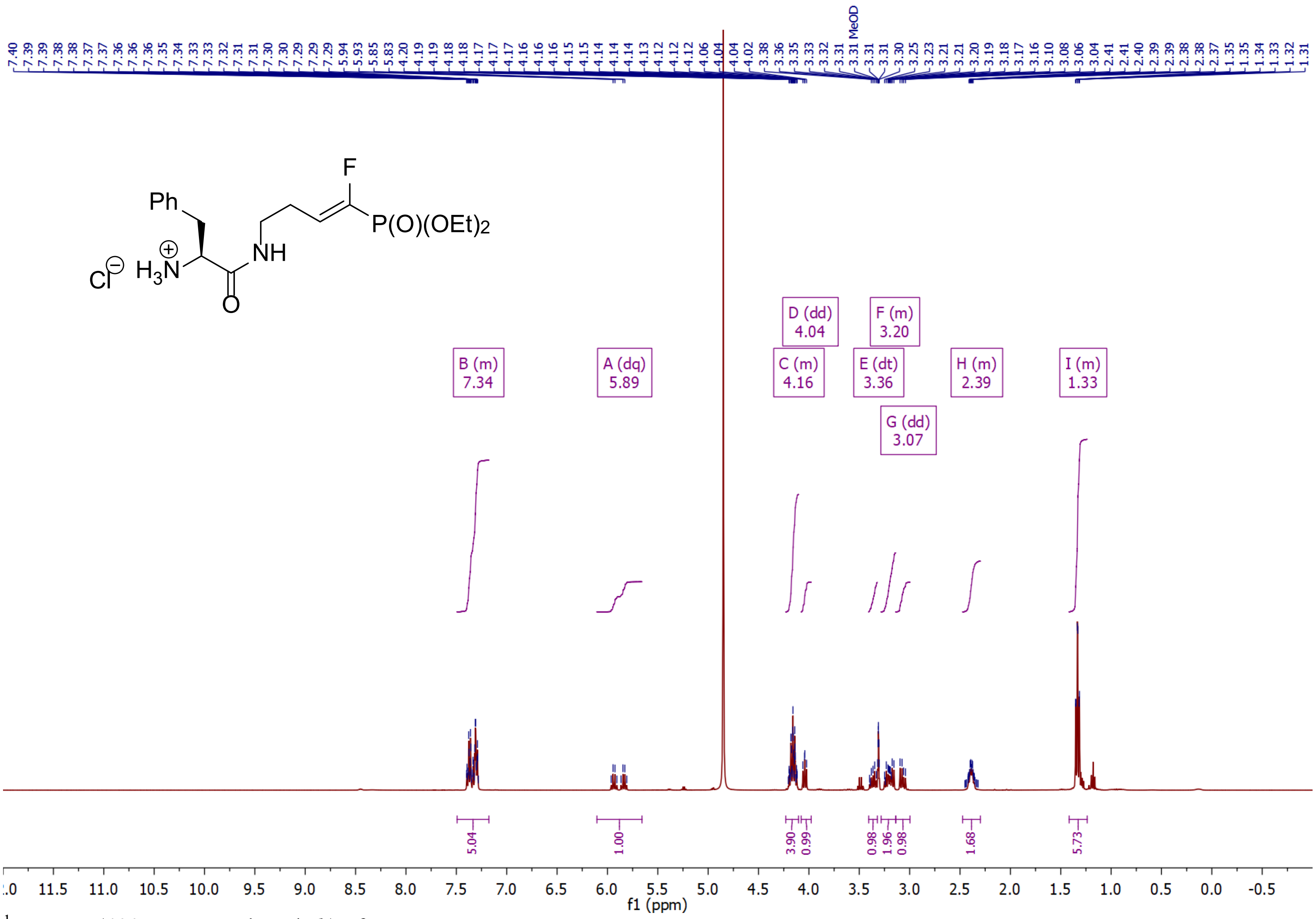

^{1}H NMR (400 MHz, Methanol-d_4) of **5a**.

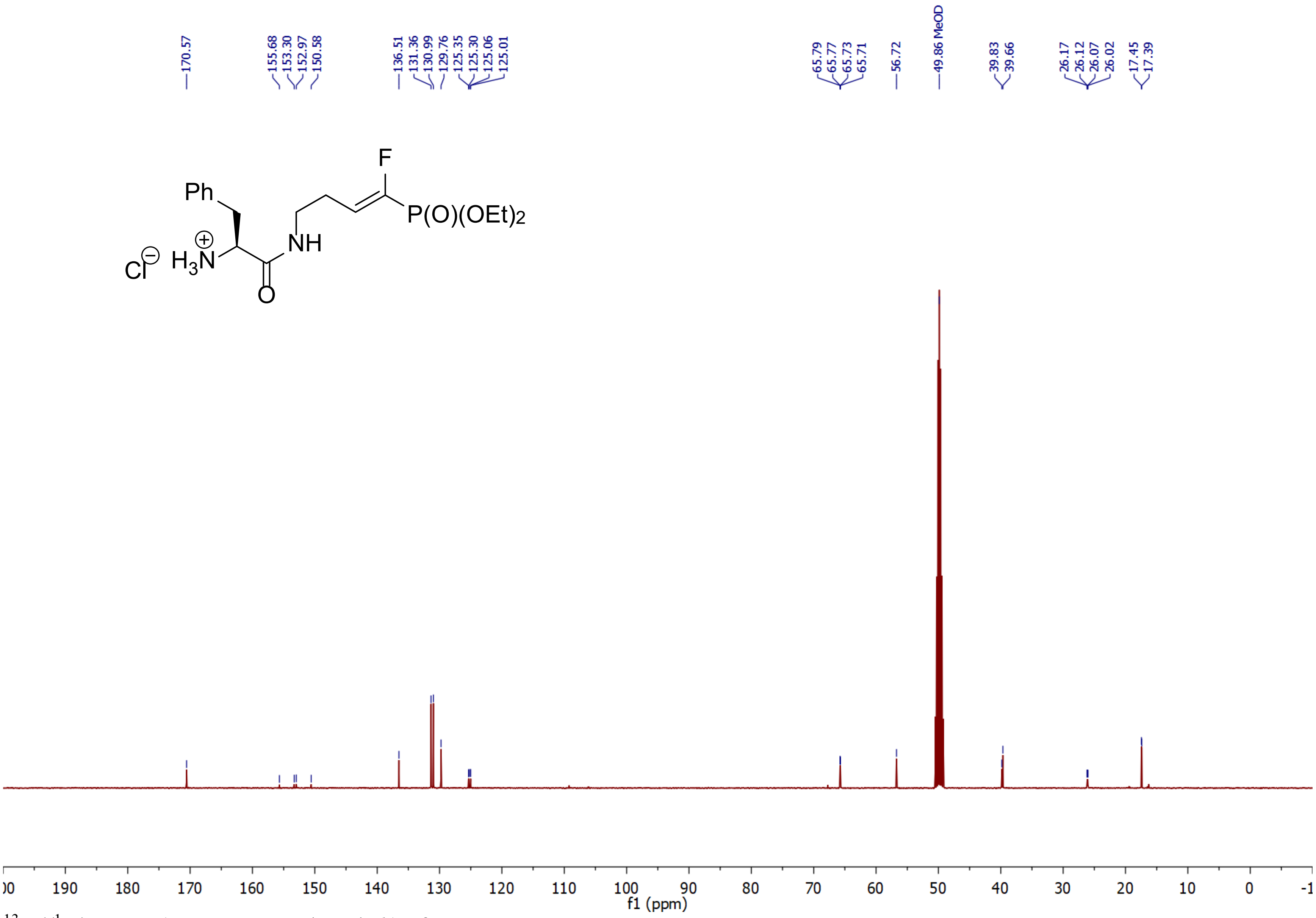

$^{13}C\{/^{1}H\}$ NMR (101 MHz, Methanol-d_4) of **5a**.

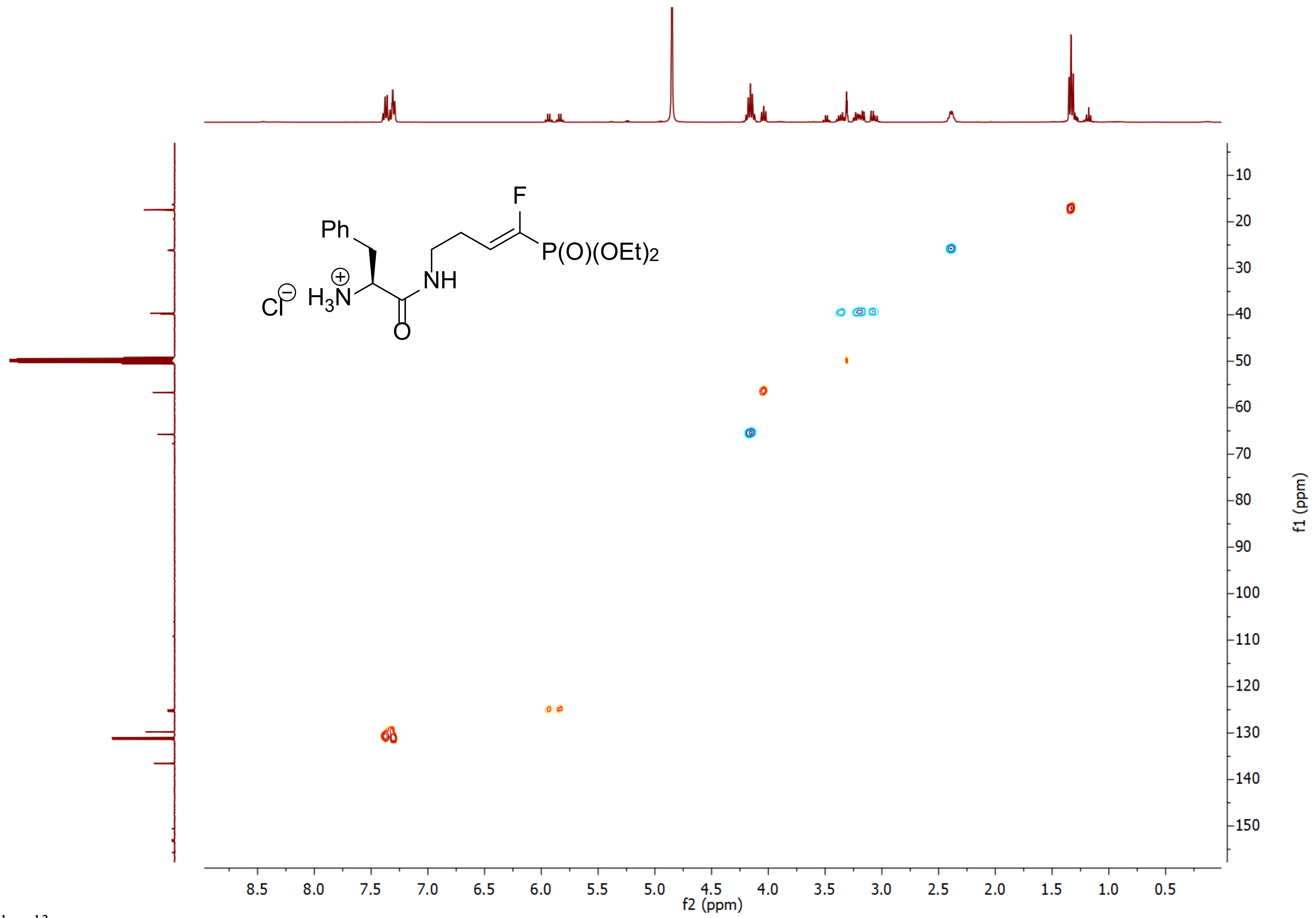

^{1}H-^{13}C HSQC (400 MHz /101 MHz, Methanol-d_4) of **5a**.

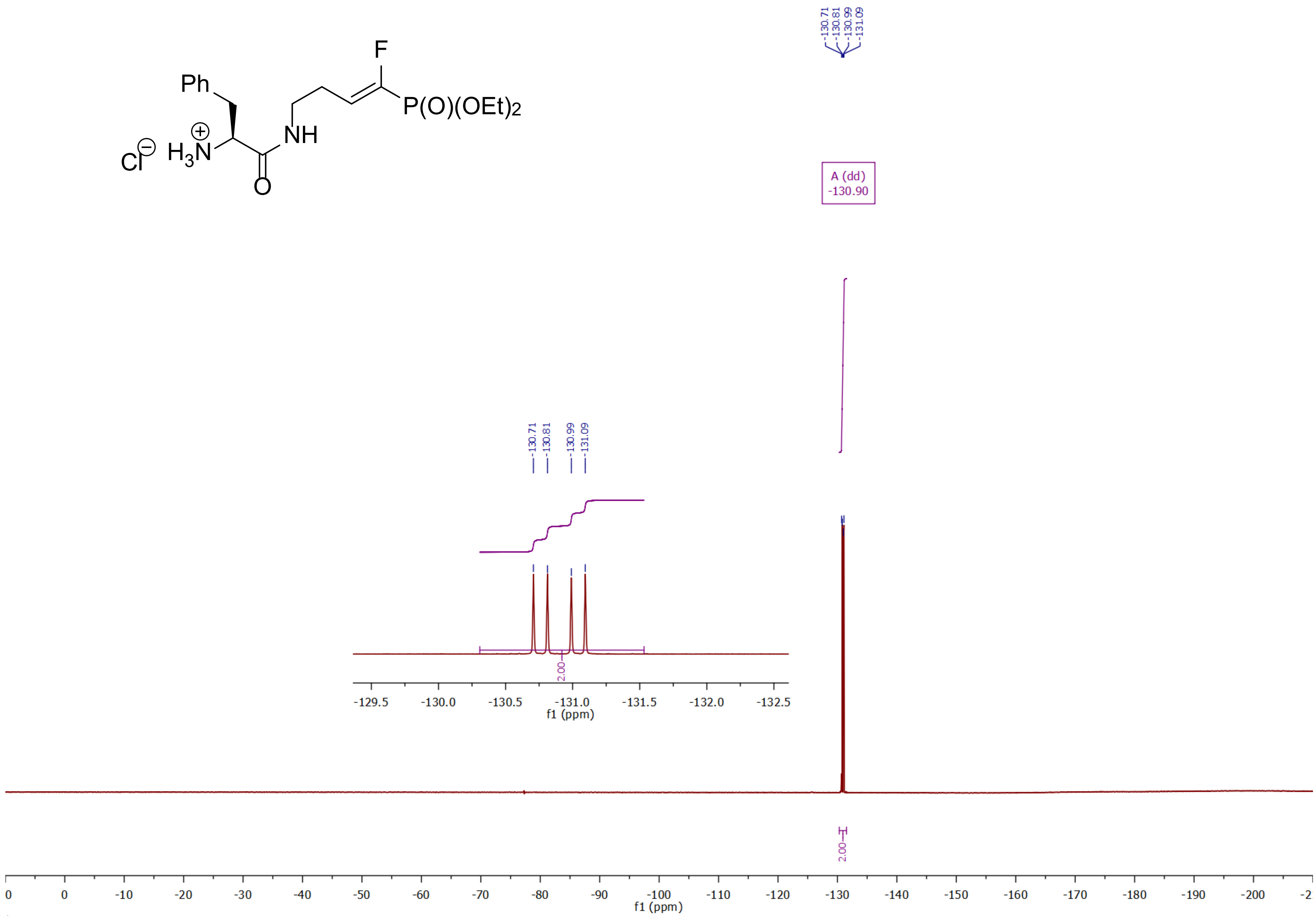

^{19}F NMR (377 MHz, Methanol-d_4) of **5a**.

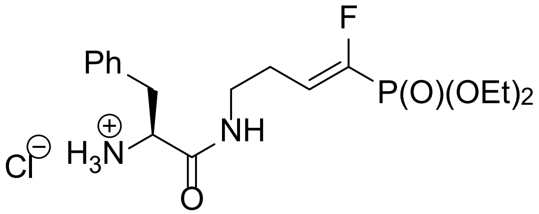

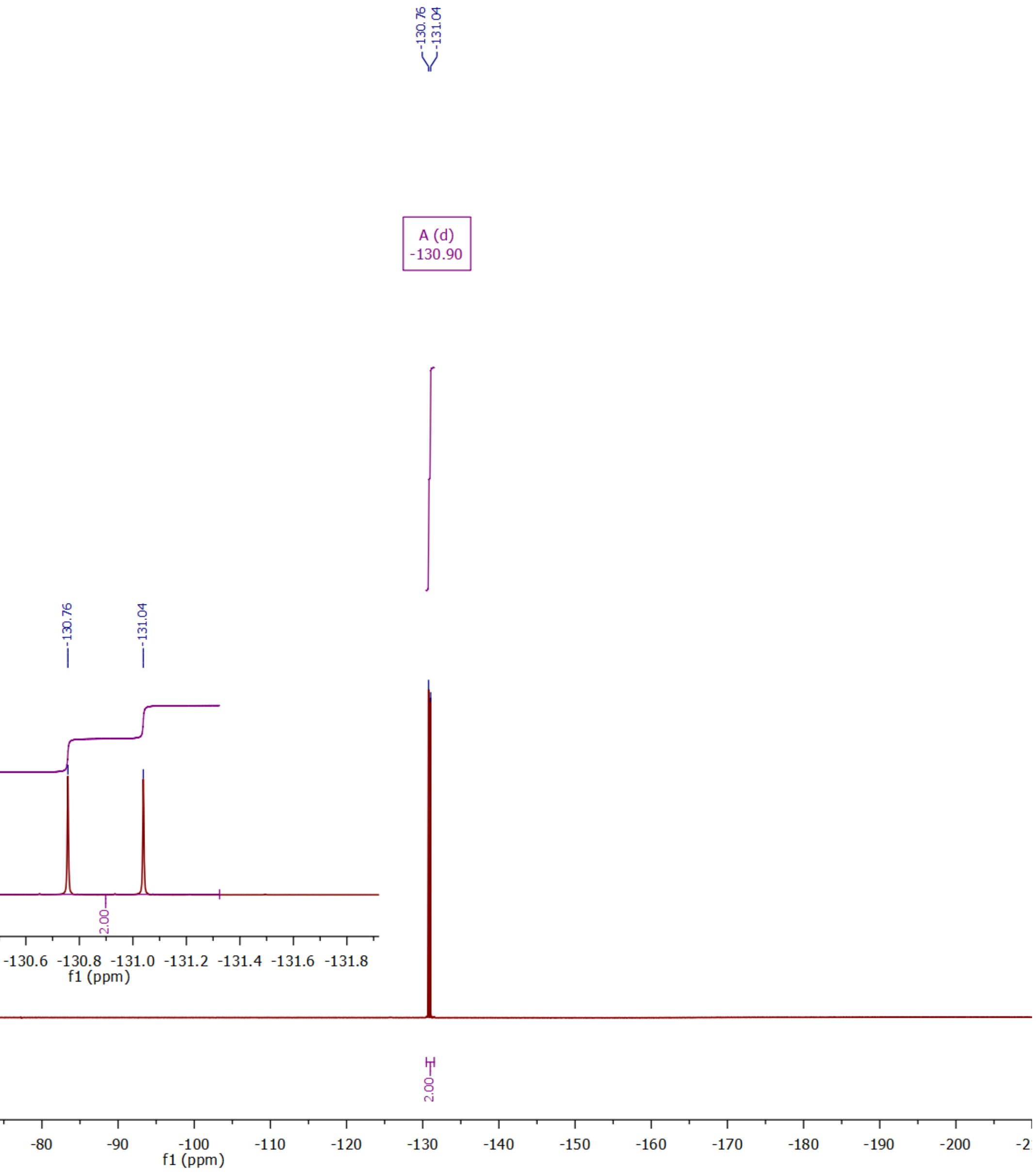

$^{19}F\{/^{1}H\}$ NMR (377 MHz, Methanol-d_4) of **5a**.

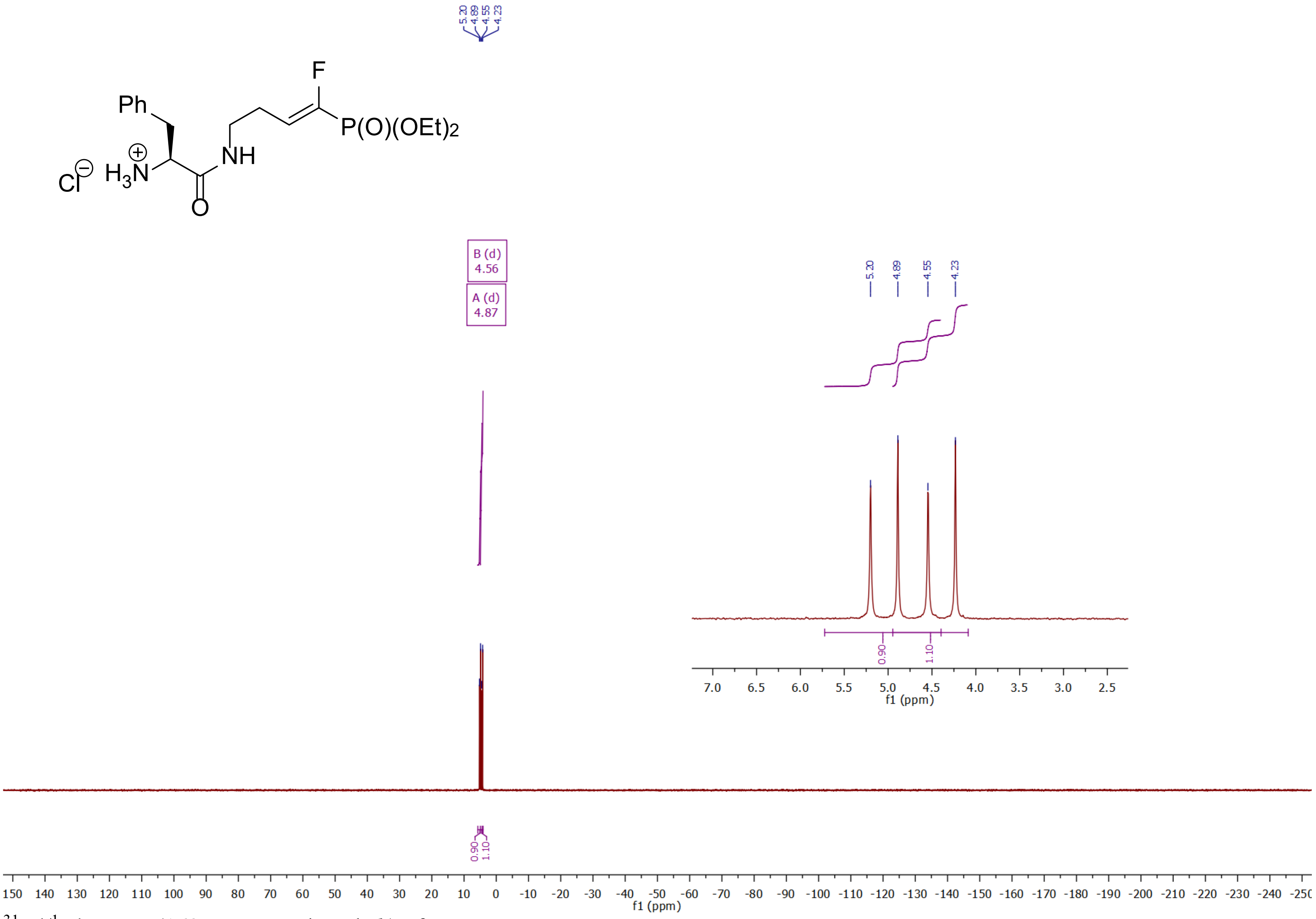

^{31}P{/^{1}H} NMR (162 MHz, Methanol-d_4) of **5a**.

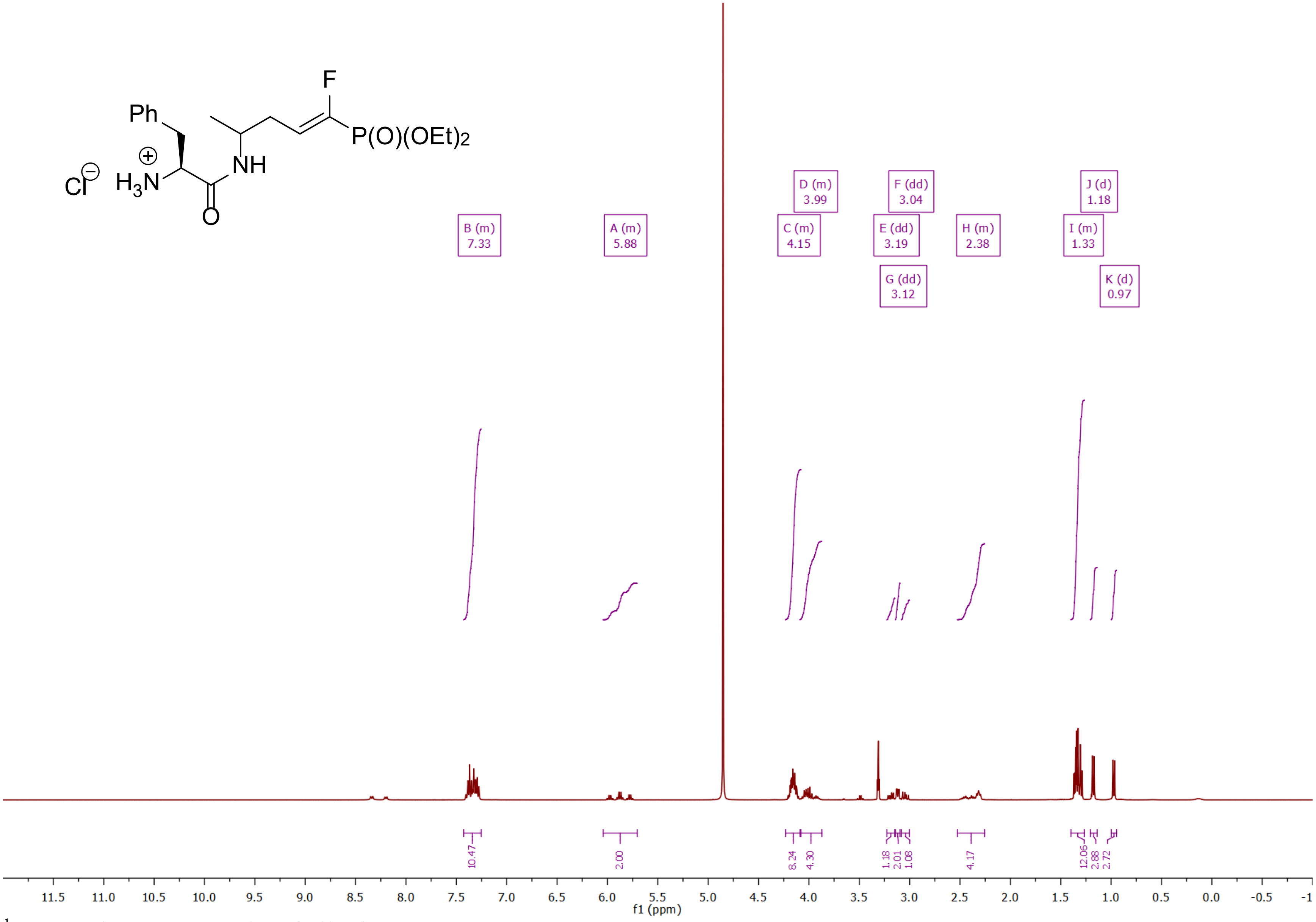

^{1}H NMR (400 MHz, Methanol-d_4) of ***rac*-5b**.

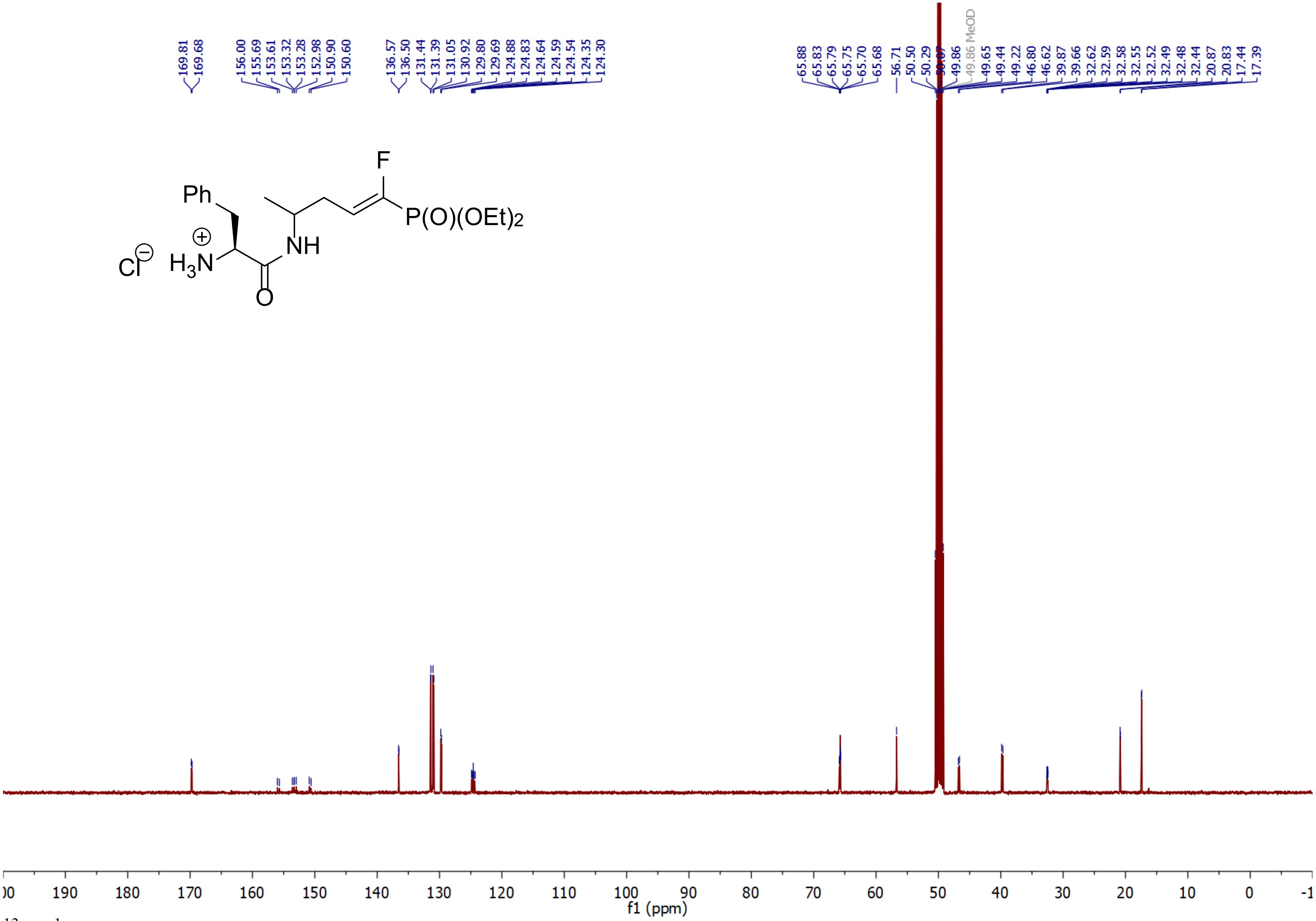

$^{13}C\{/^{1}H\}$ NMR (101 MHz, Methanol-d_4) of ***rac*-5b**.

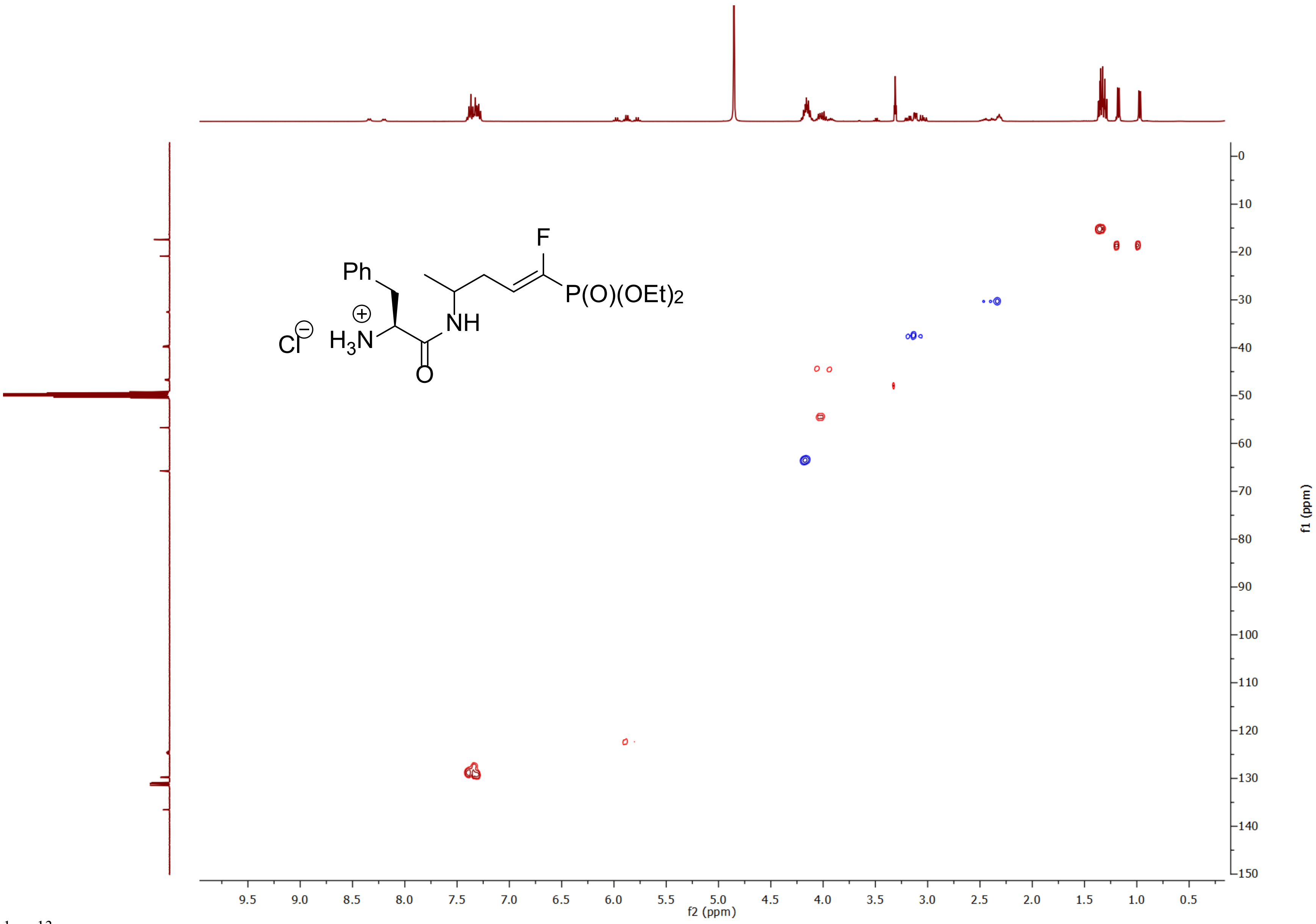

^{1}H-^{13}C HSQC (400 MHz /101 MHz, Methanol-d_4) of ***rac*-5b**.

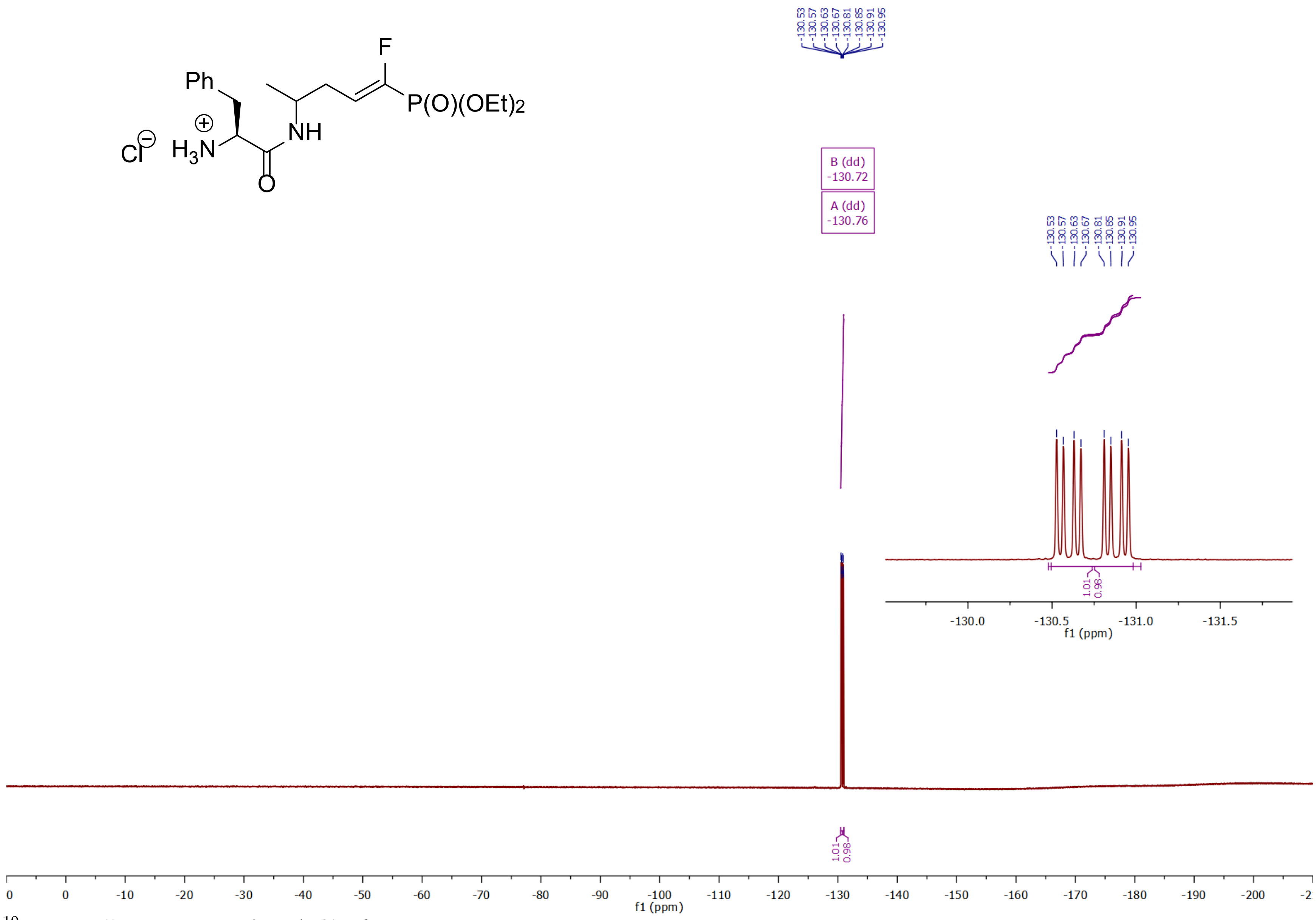

^{19}F NMR (377 MHz, Methanol-d_4) of ***rac*-5b**.

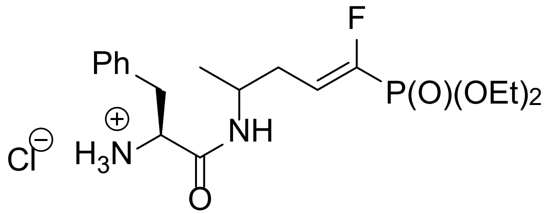

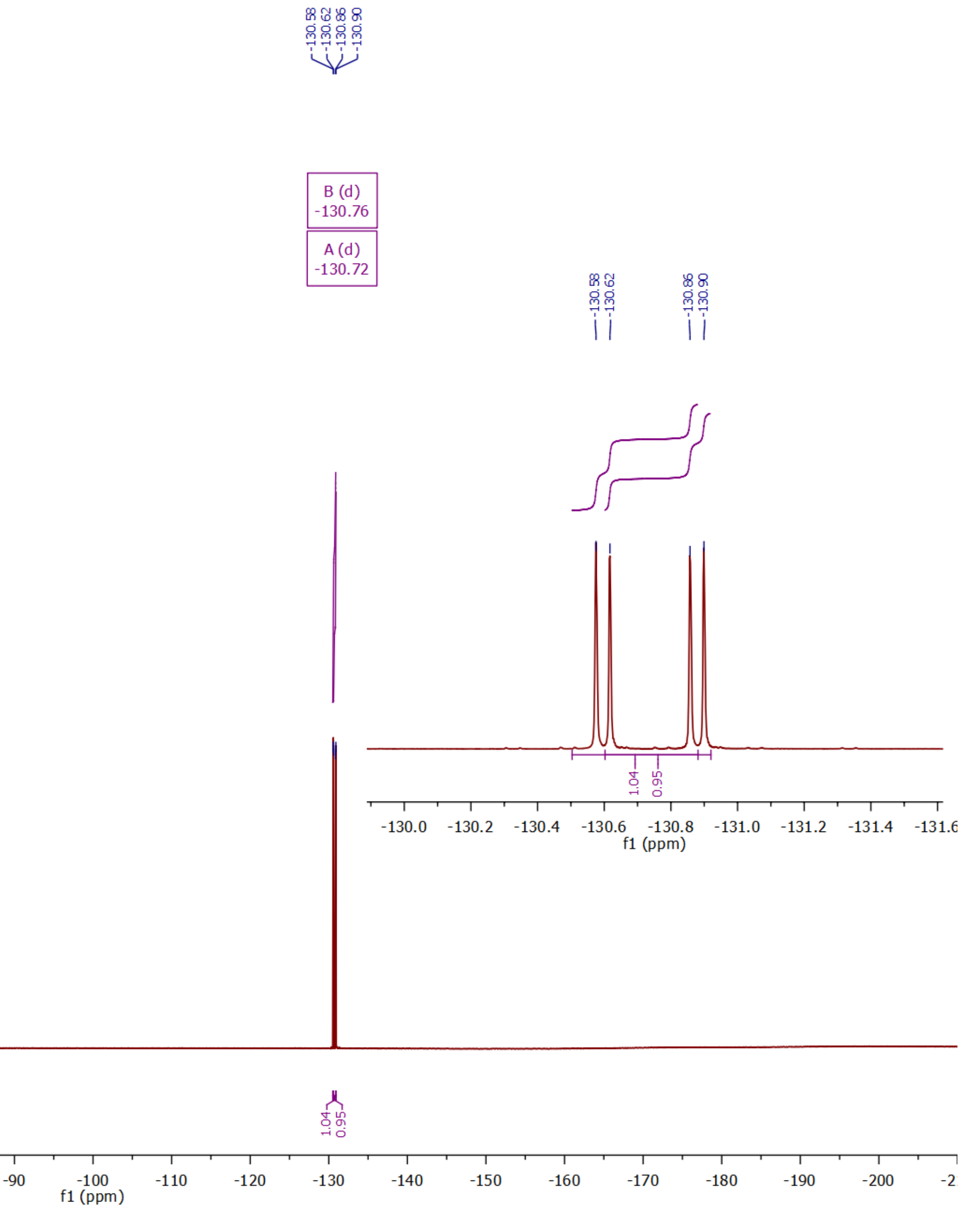

$^{19}F\{/^{1}H\}$ NMR (377 MHz, Methanol-d_4) of ***rac*-5b**.

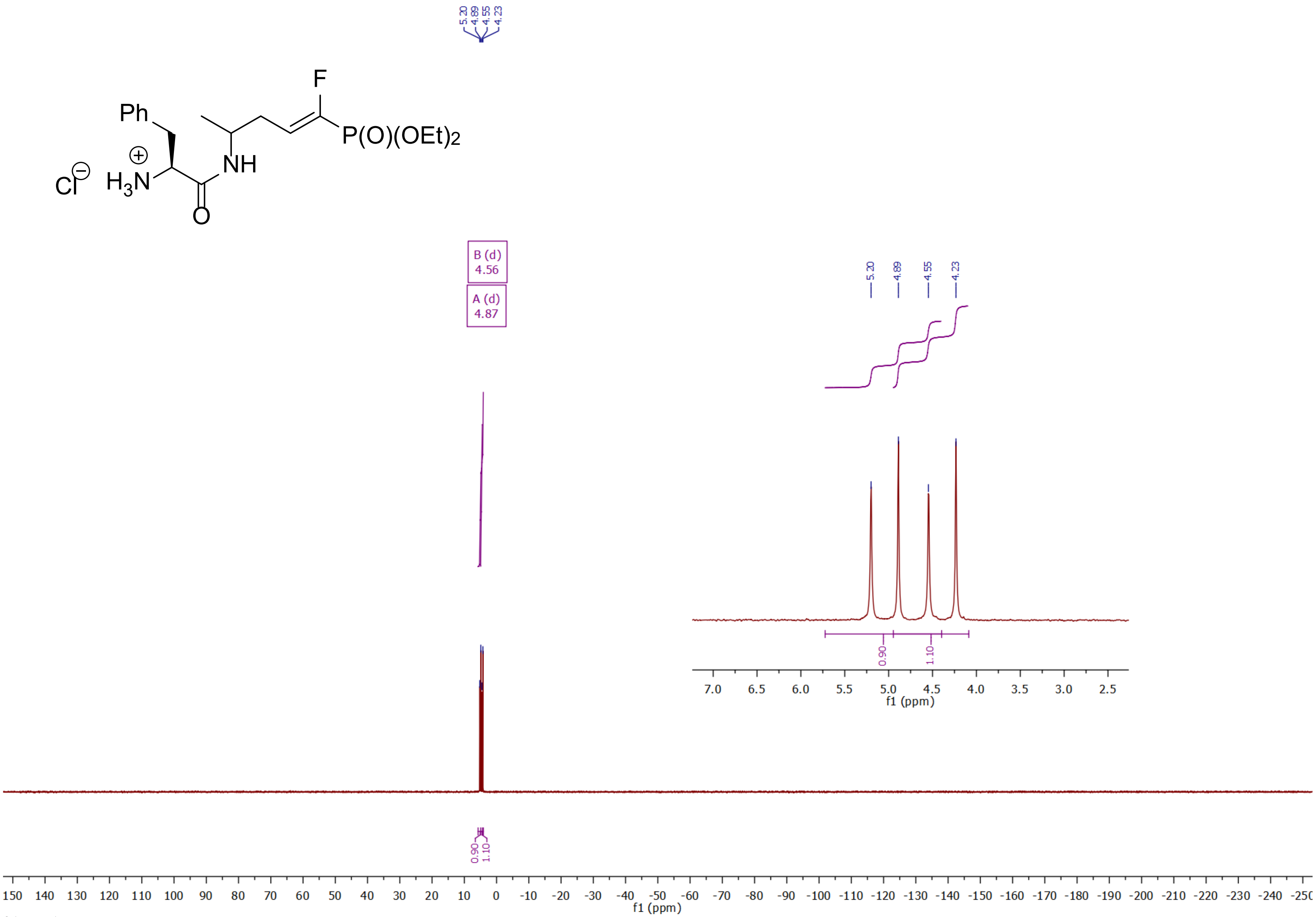

$^{31}P\{/^{1}H\}$ NMR (162 MHz, Methanol-d_4) of ***rac*-5b**.

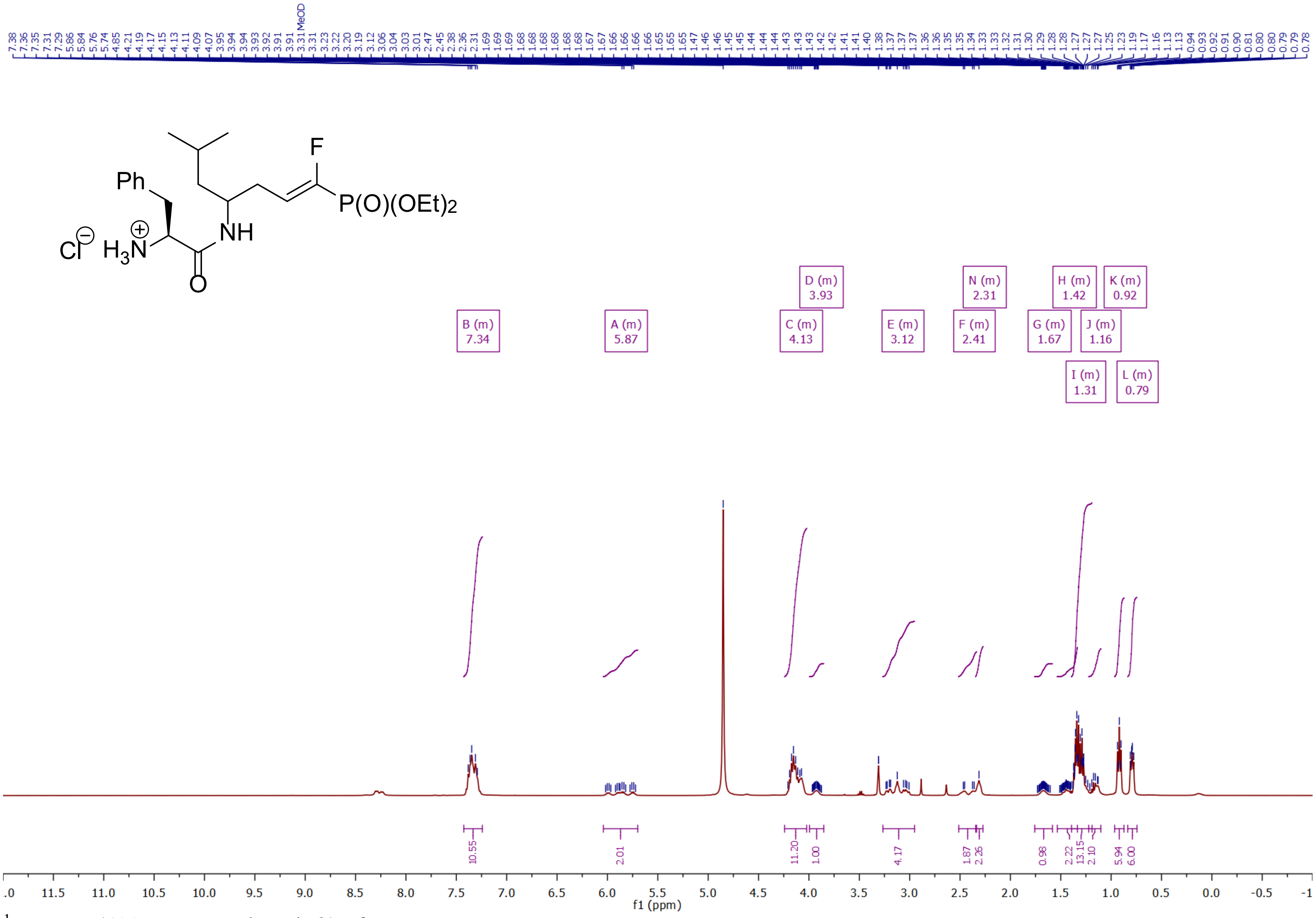

^{1}H NMR (400 MHz, Methanol-d_4) of ***rac*-5c**.

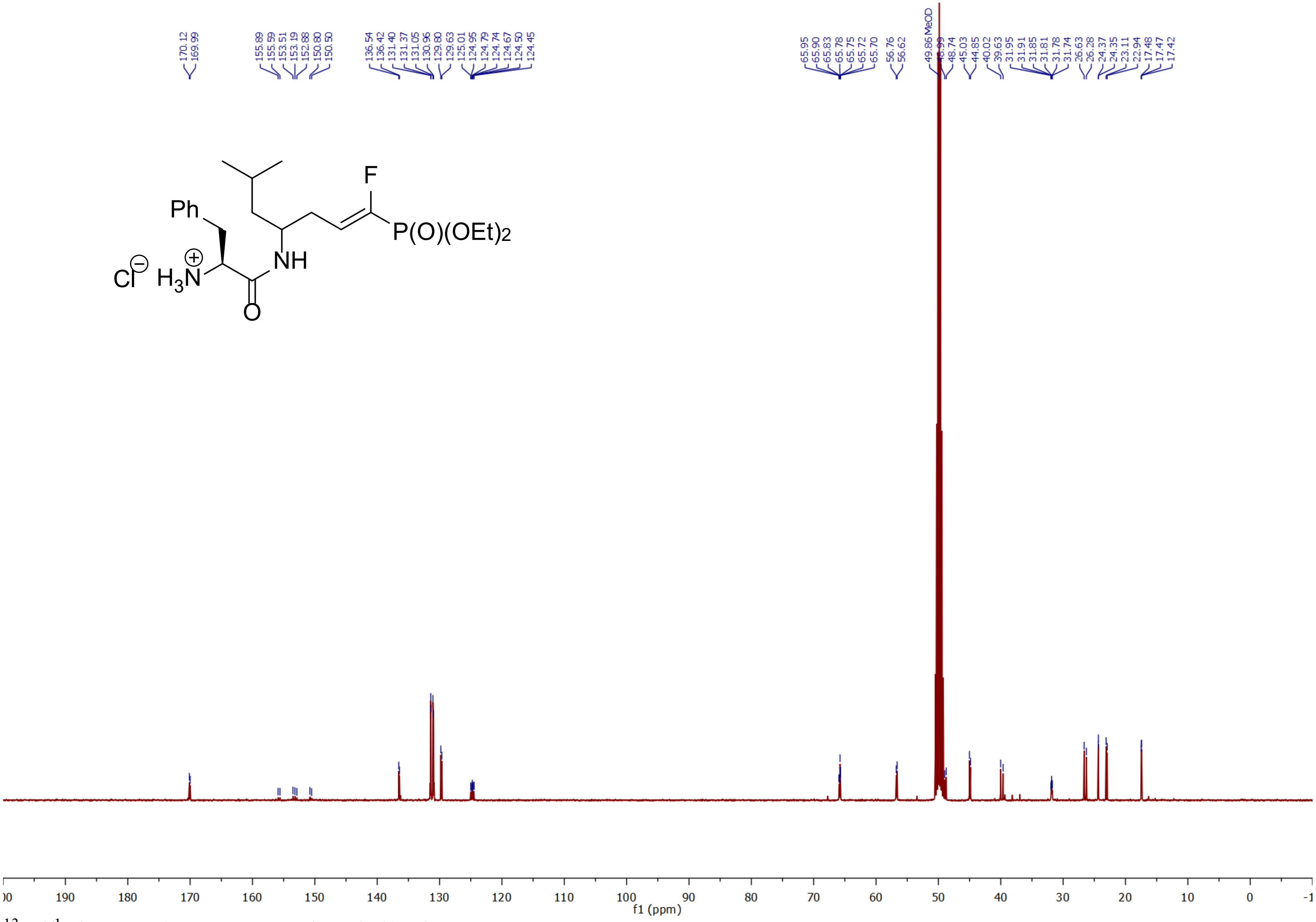

$^{13}C\{/^{1}H\}$ NMR (101 MHz, Methanol-d_4) of ***rac*-5c**.

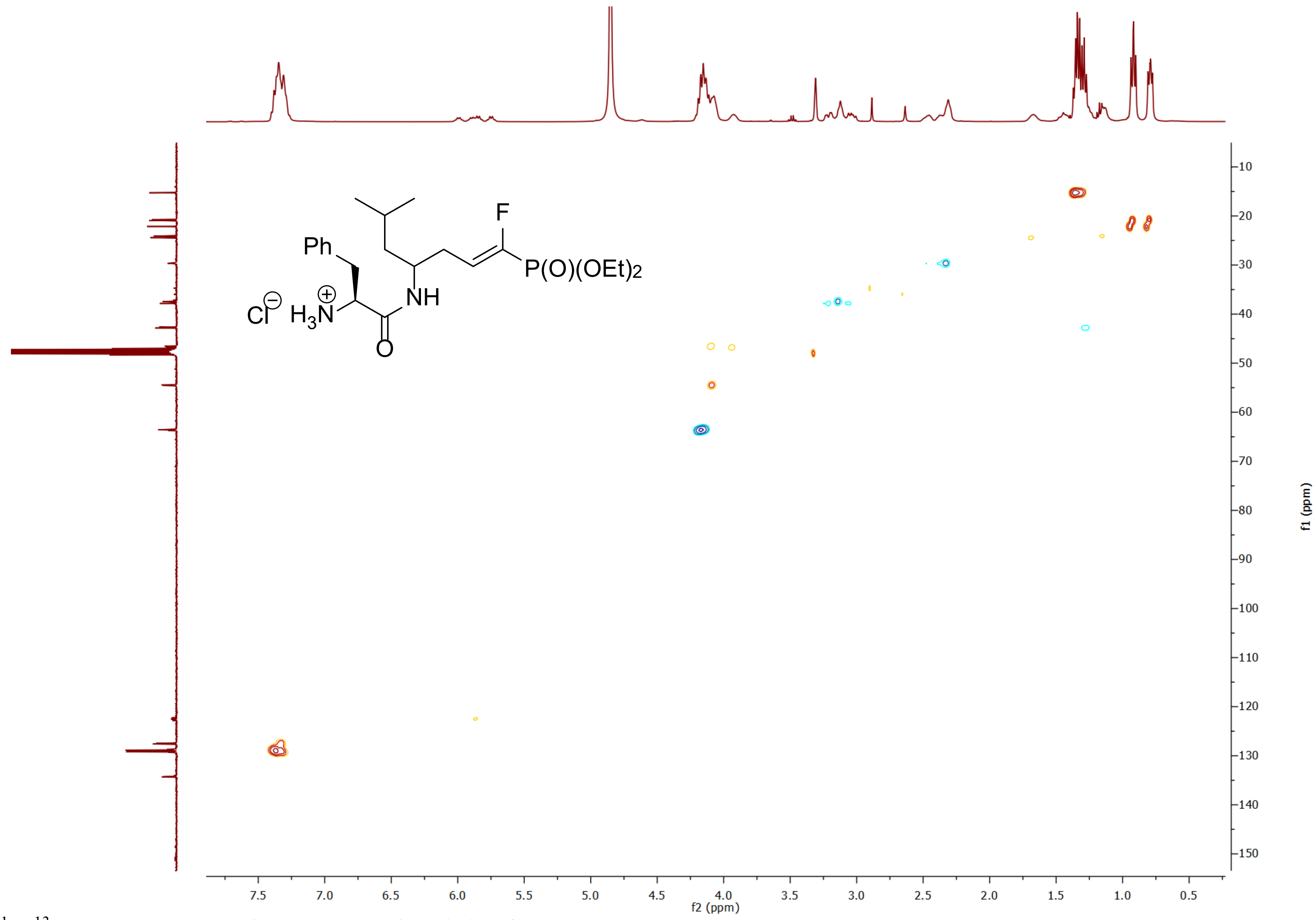

^{1}H-^{13}C HSQC (400 MHz /101 MHz, Methanol-d_4) of ***rac*-5c**.

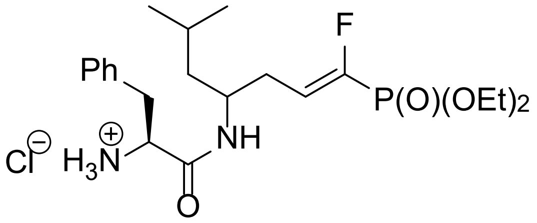

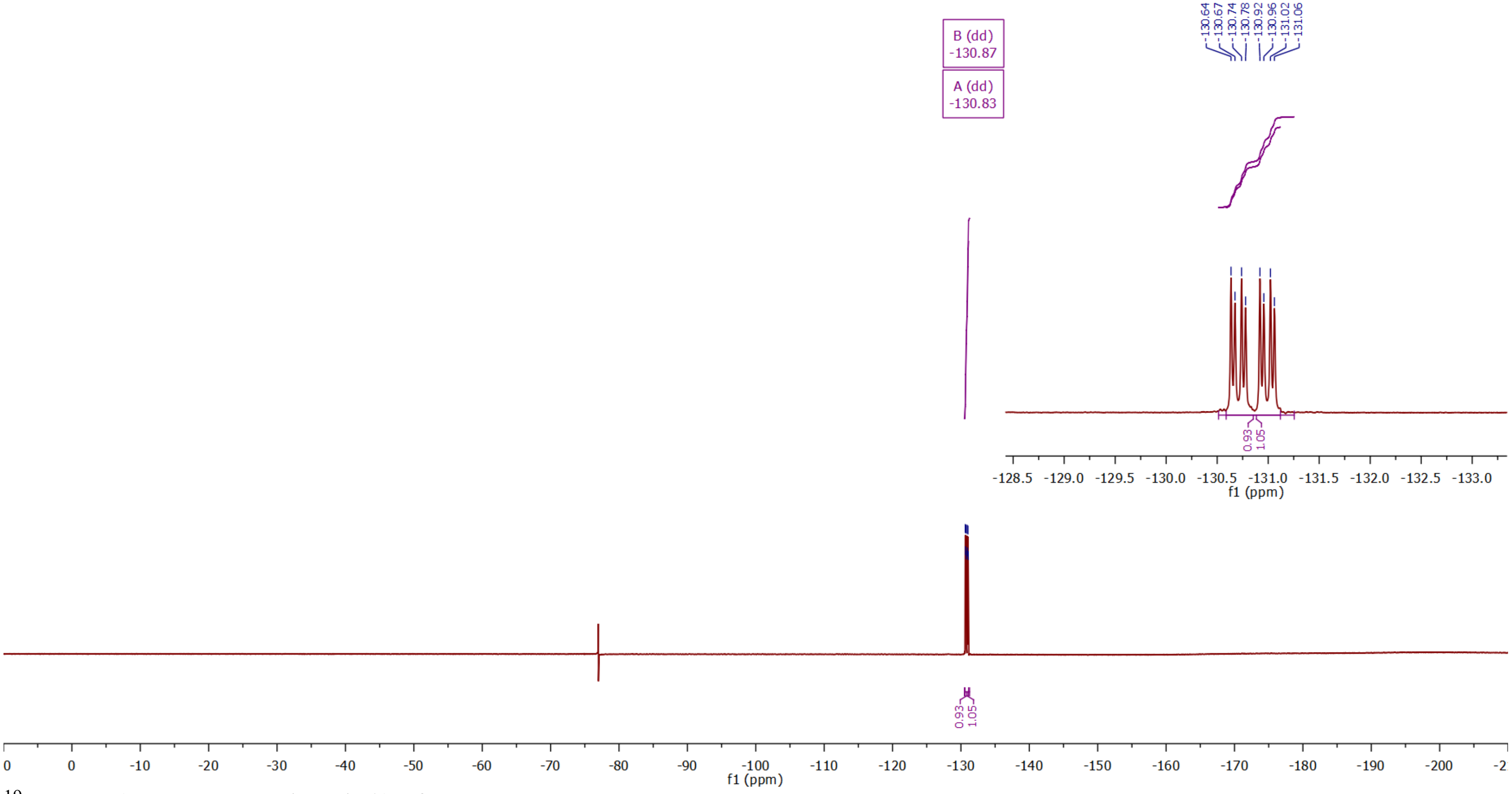

^{19}F NMR (377 MHz, Methanol-d_4) of ***rac*-5c**.

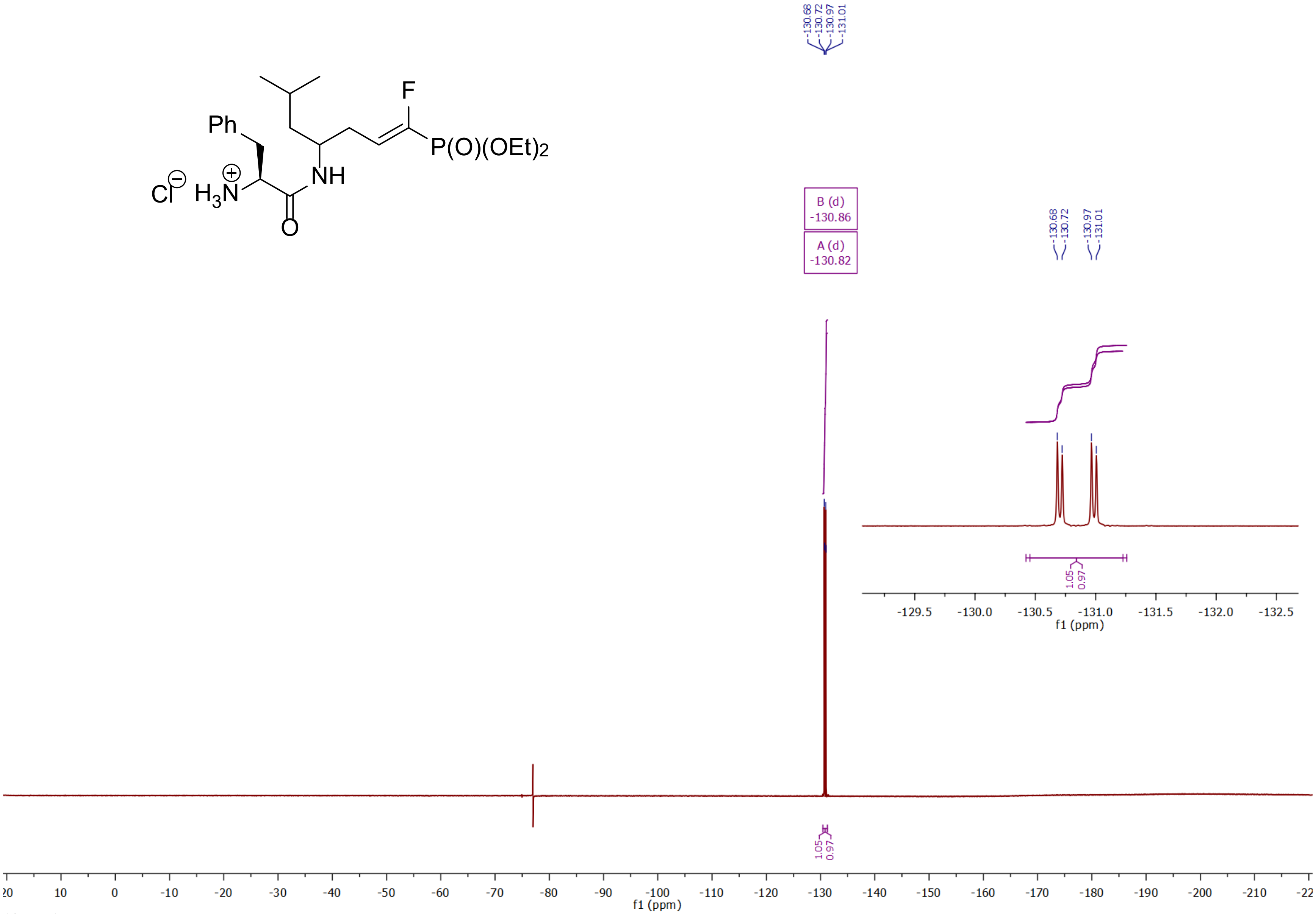

^{19}F{/^{1}H} NMR (377 MHz, Methanol-d_4) of ***rac*-5c**.

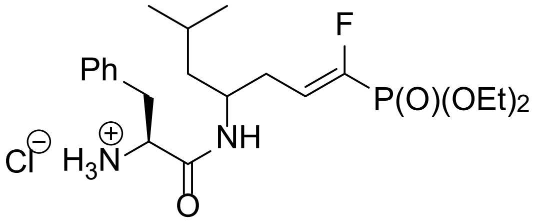

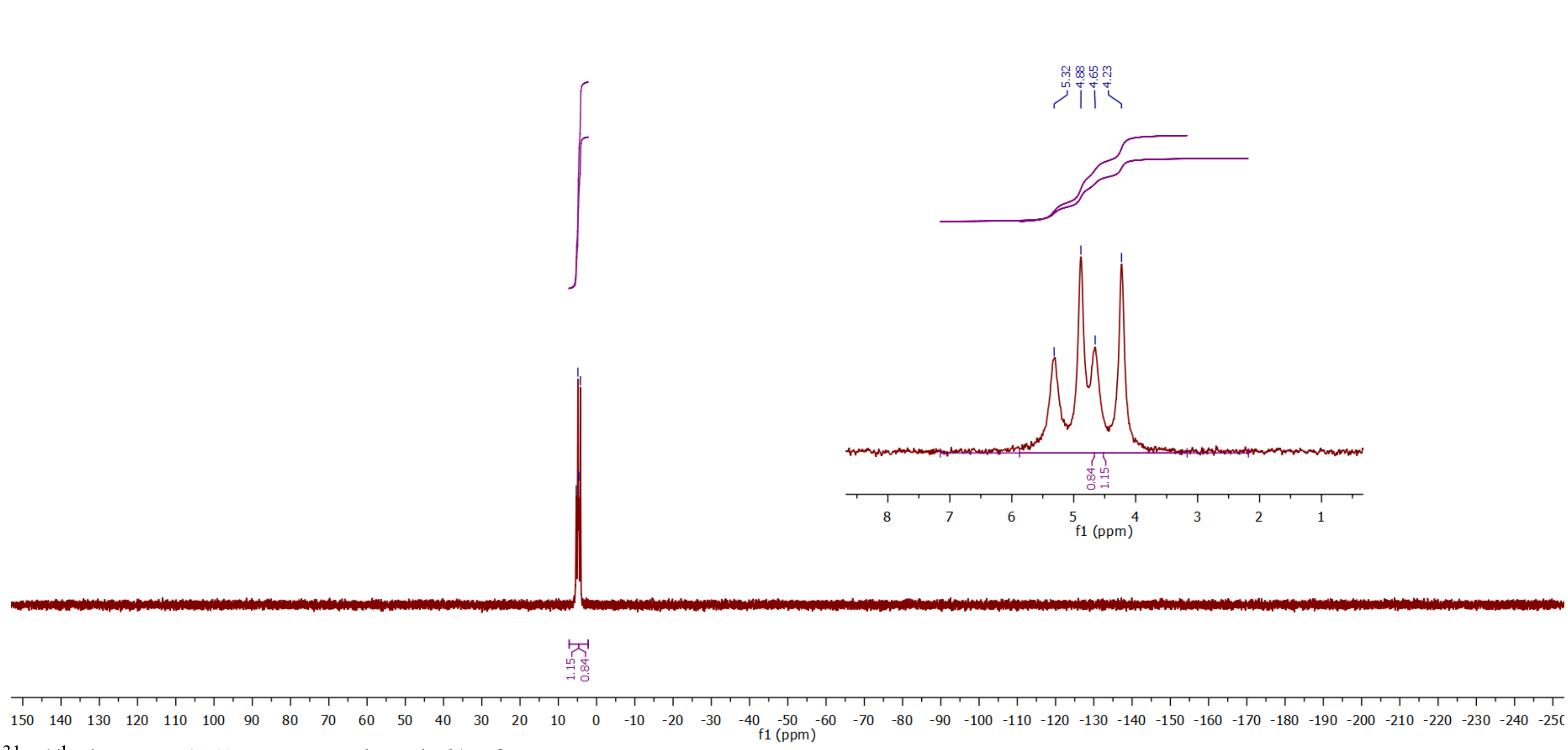

$^{31}P\{/^{1}H\}$ NMR (162 MHz, Methanol-d_4) of ***rac*-5c**.

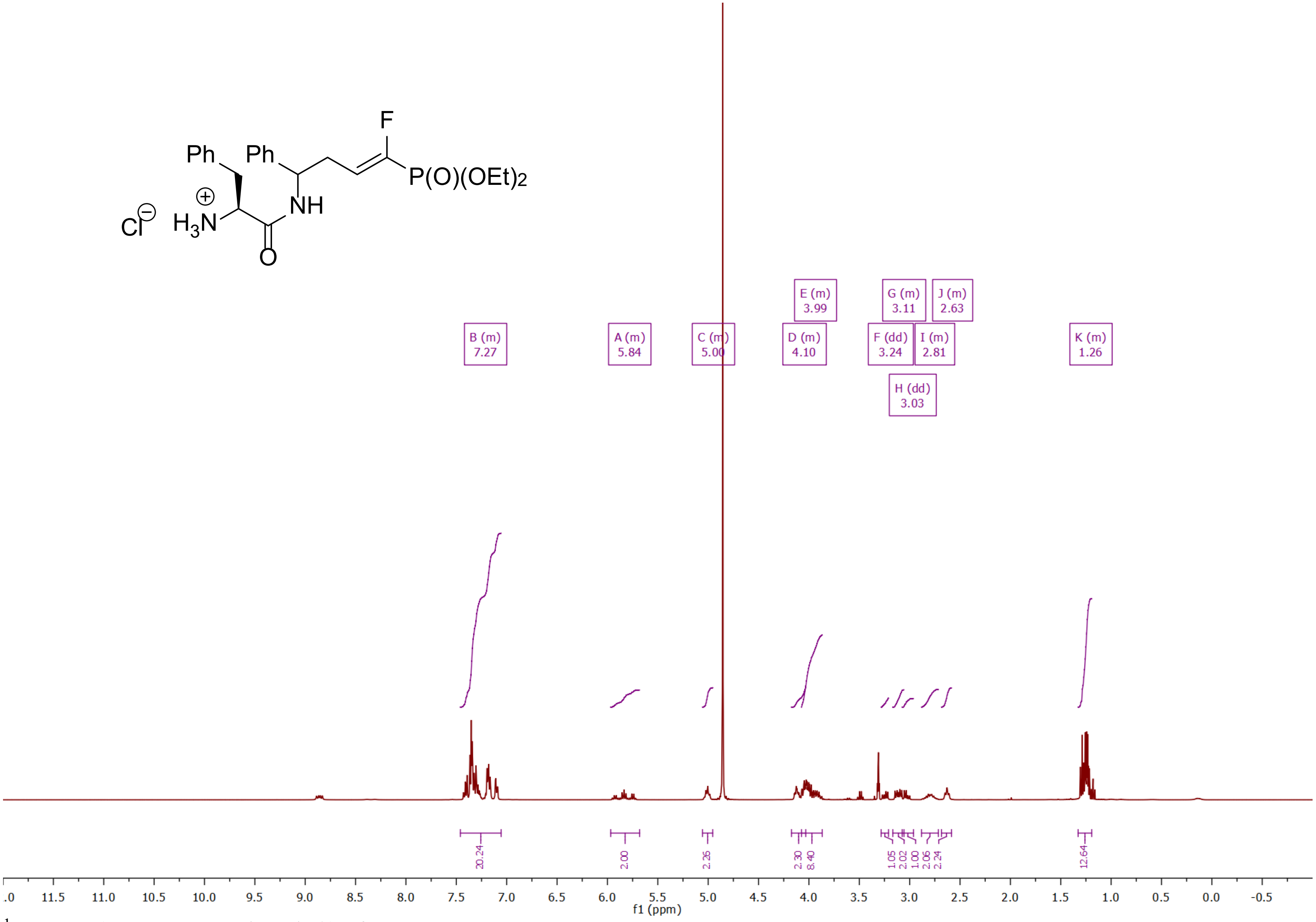

^{1}H NMR (400 MHz, Methanol-d_4) of ***rac*-5d**.

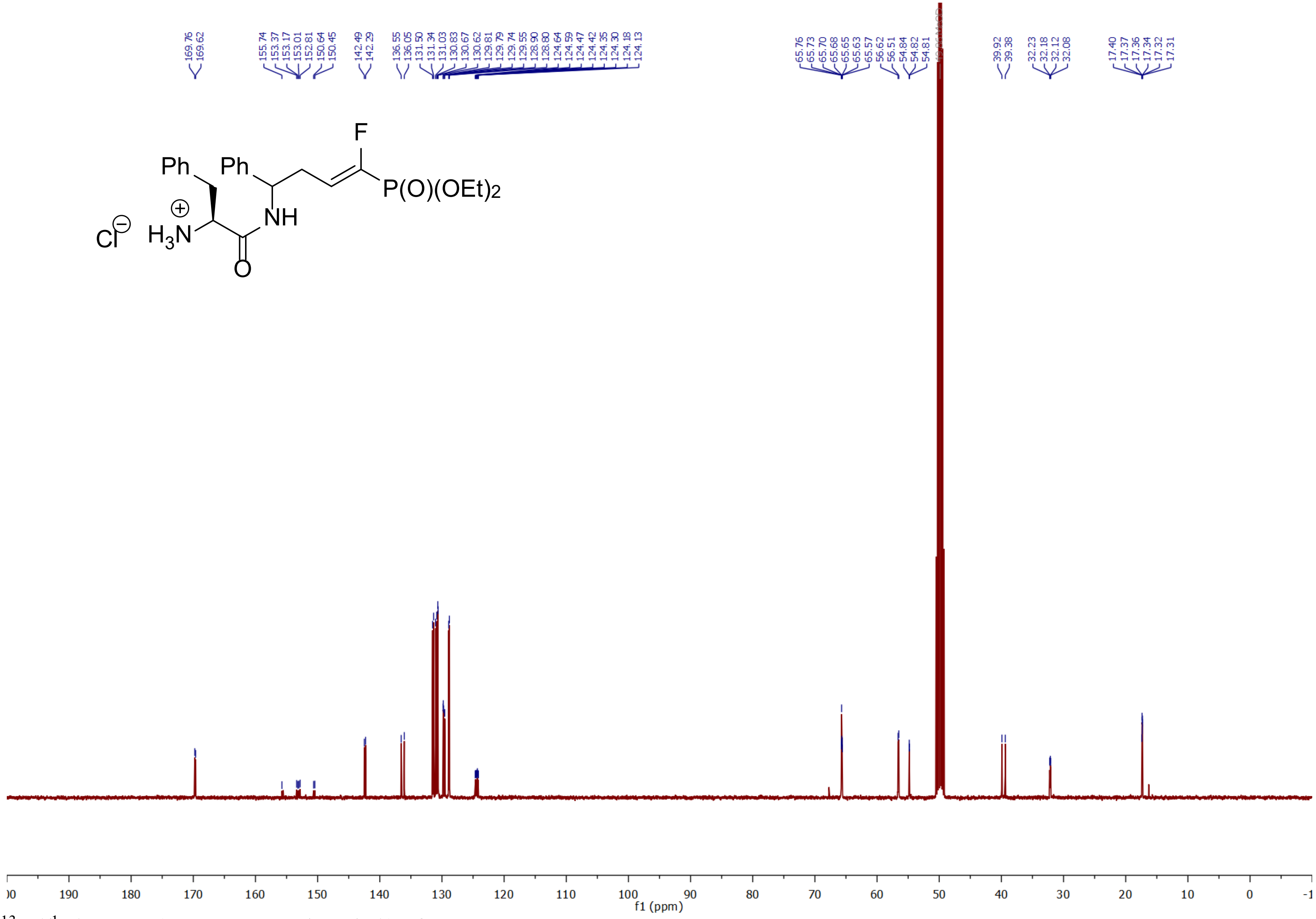

$^{13}C\{/^{1}H\}$ NMR (101 MHz, Methanol-d_4) of ***rac*-5d**.

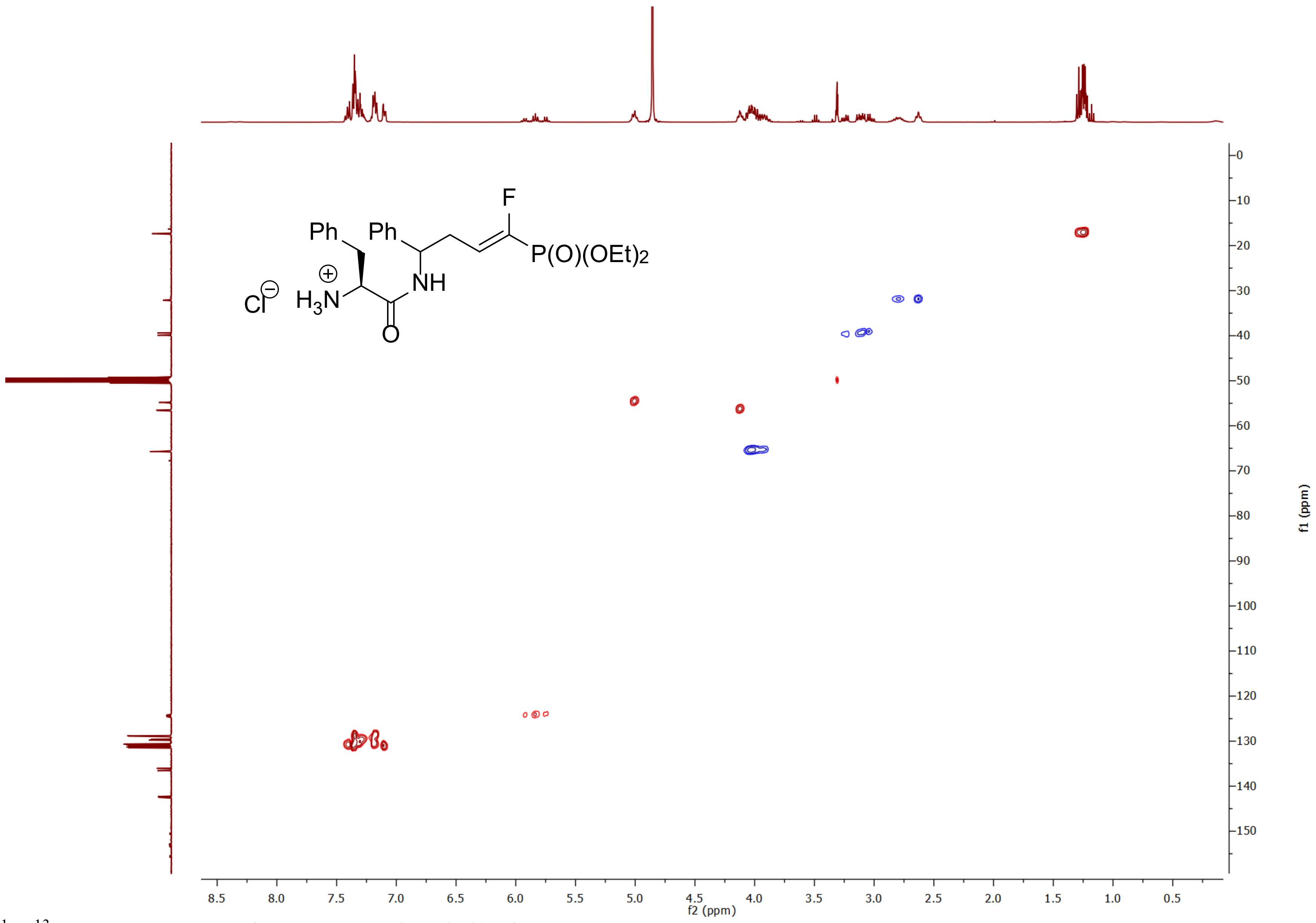

^{1}H-^{13}C HSQC (400 MHz /101 MHz, Methanol-*d*$_4$) of ***rac*-5d**.

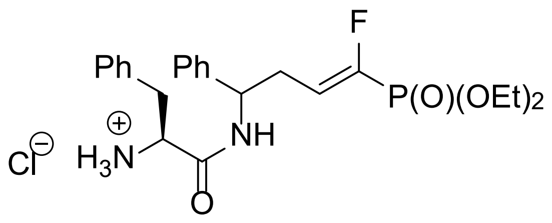

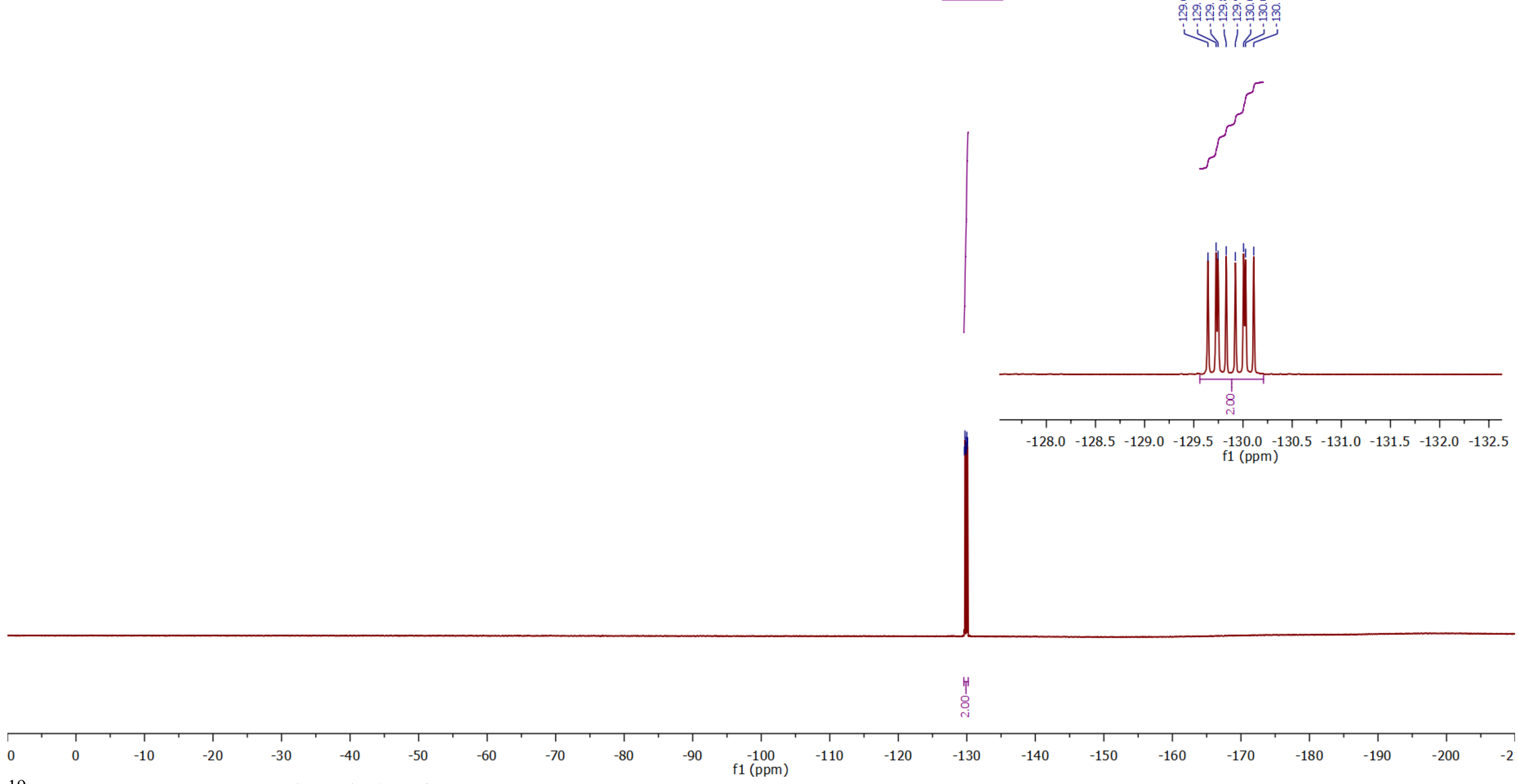

^{19}F NMR (377 MHz, Methanol-d_4) of ***rac*-5d**.

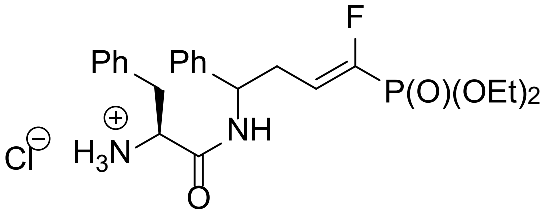

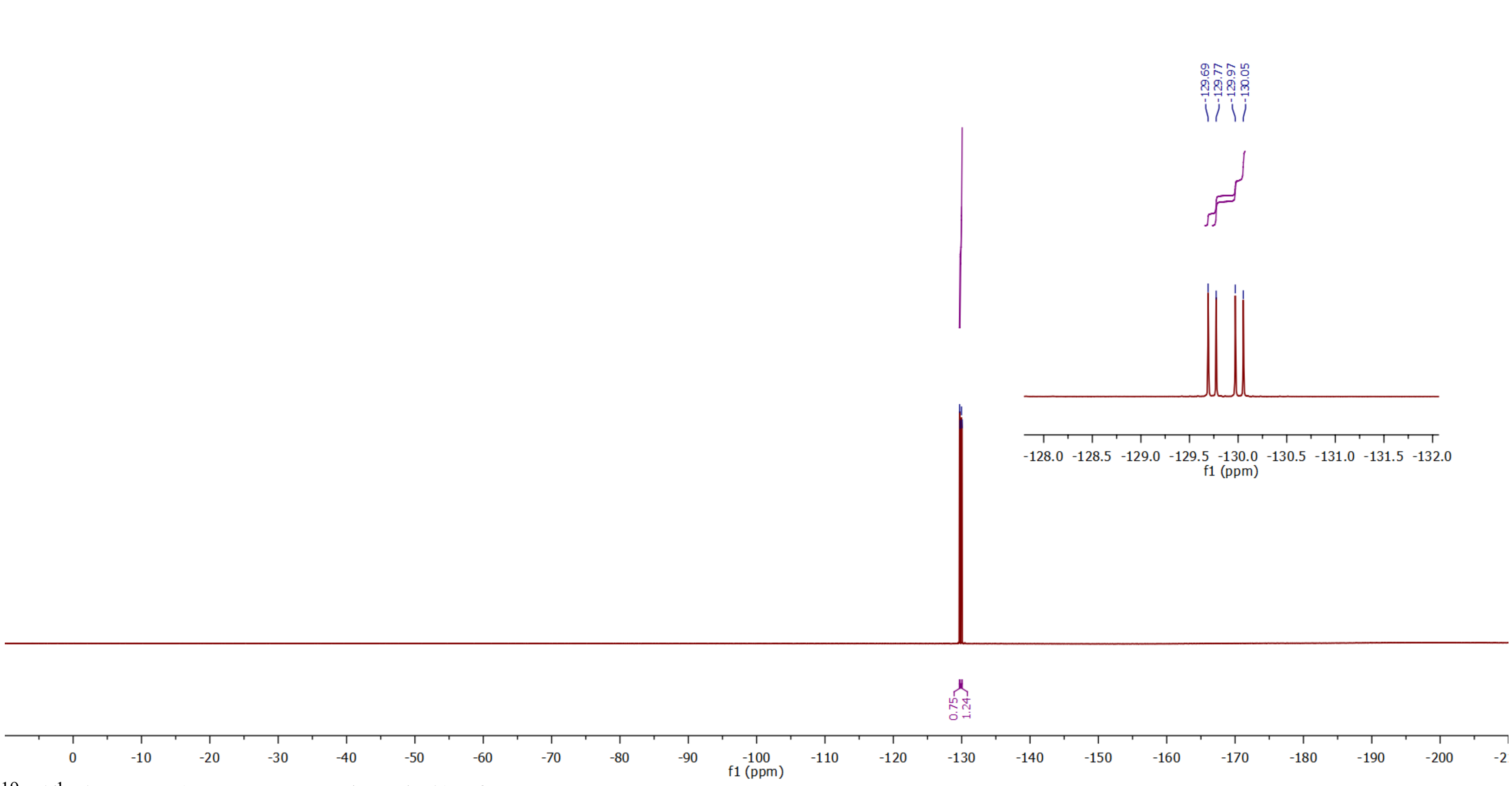

$^{19}F\{/^{1}H\}$ NMR (377 MHz, Methanol-d_4) of ***rac*-5d**.

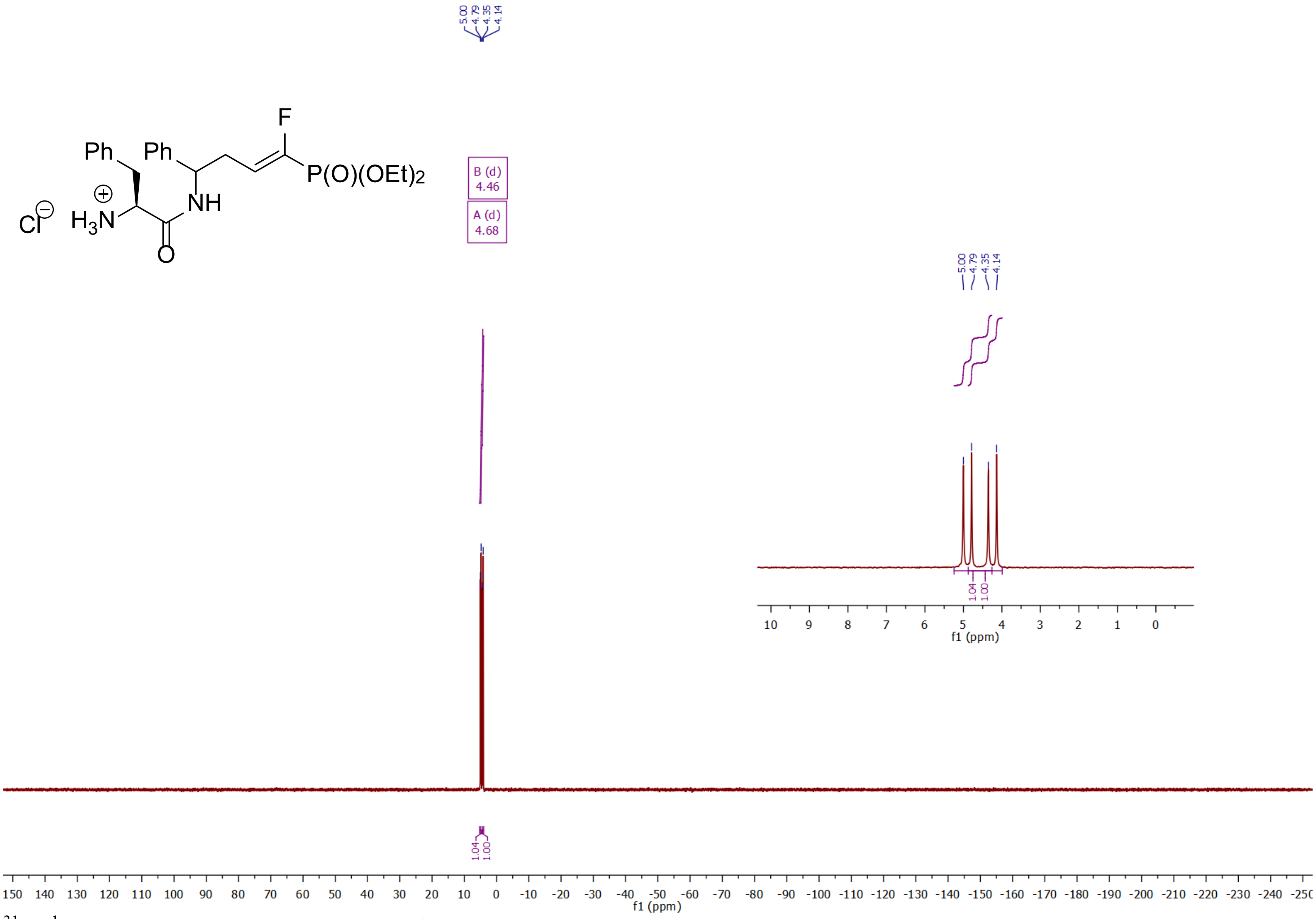

$^{31}P\{/^{1}H\}$ NMR (162 MHz, Methanol-d_4) of ***rac*-5d**.

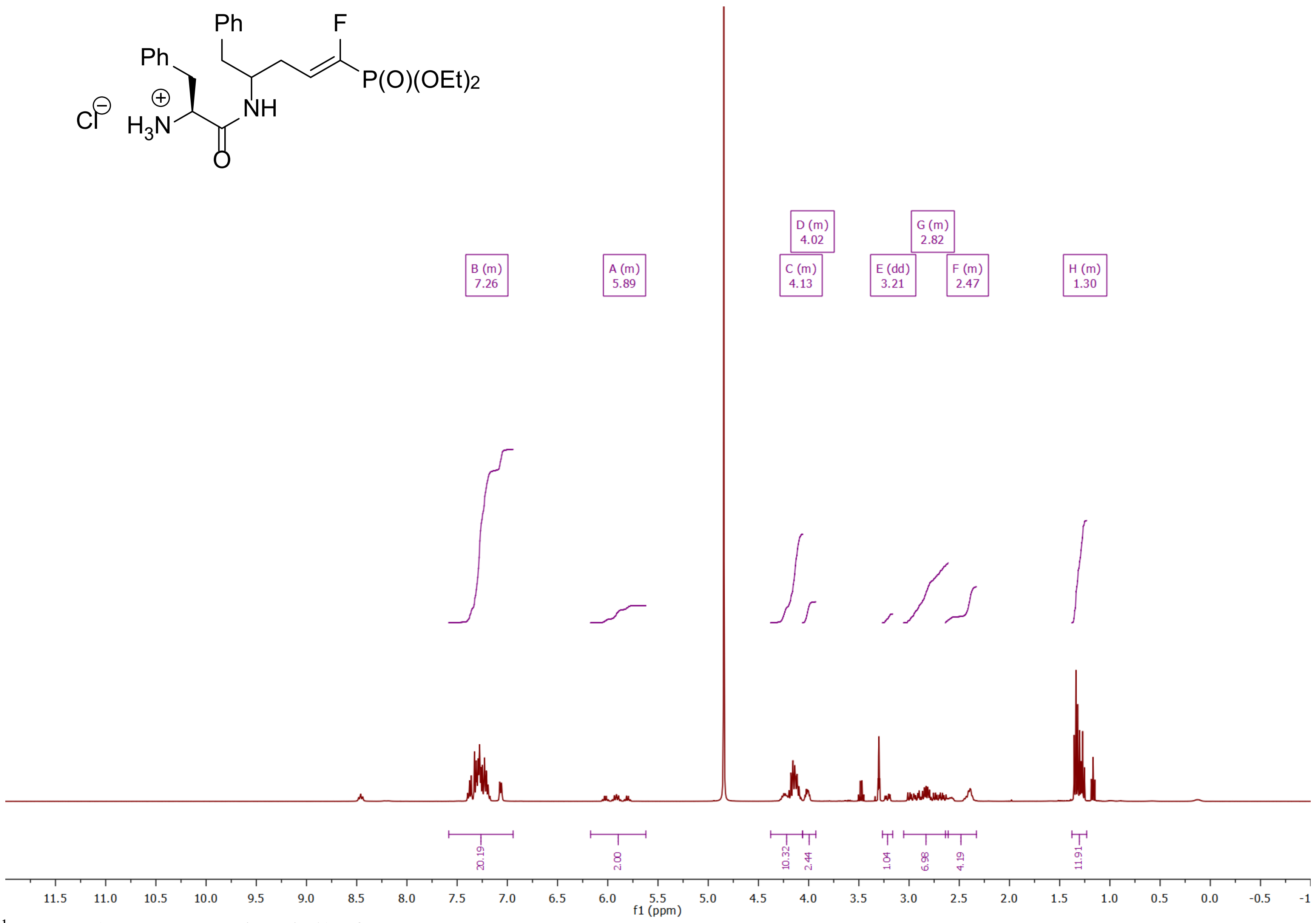

^{1}H NMR (400 MHz, Methanol-d_4) of ***rac*-5e**.

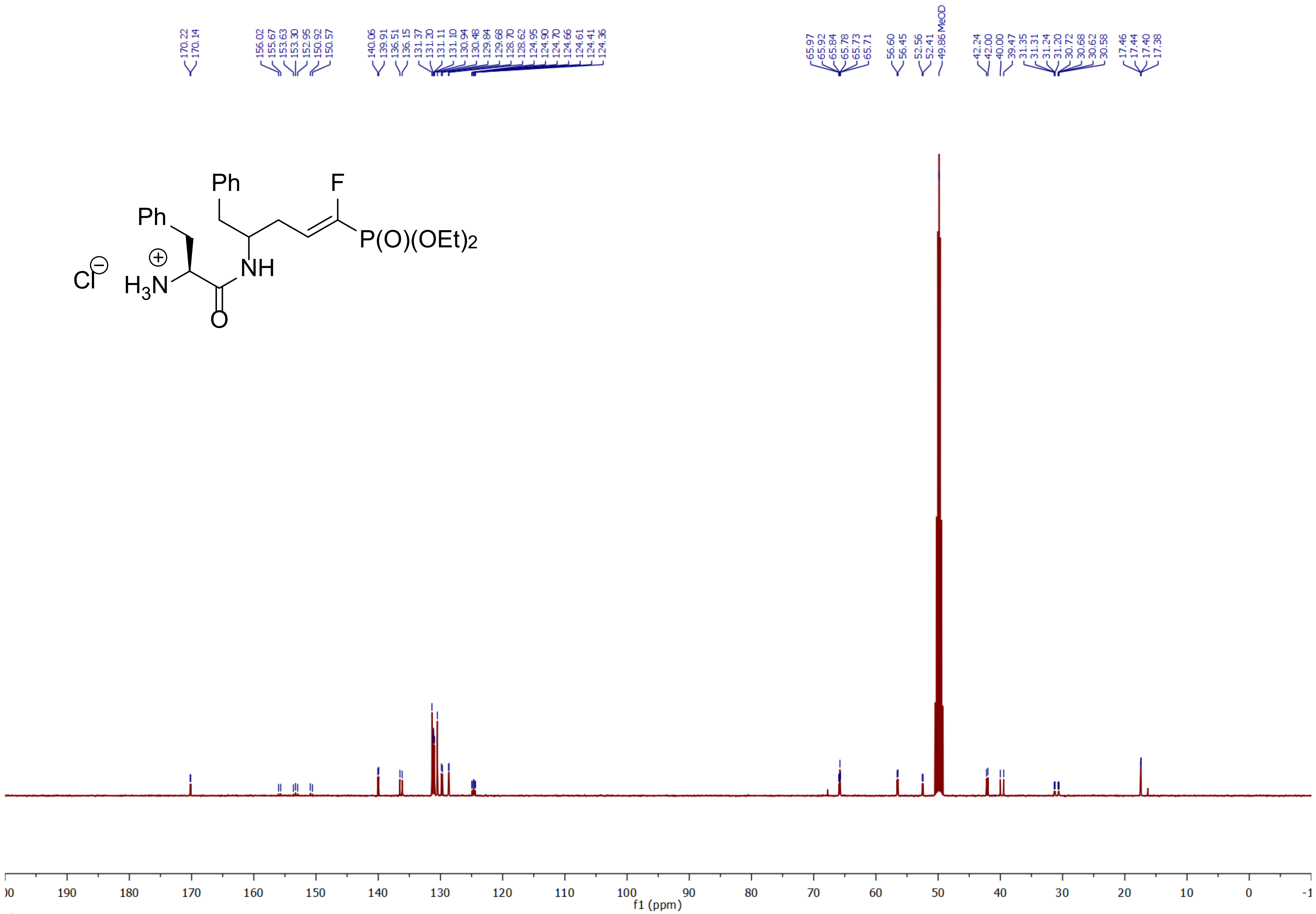

$^{13}C\{/^{1}H\}$ NMR (101 MHz, Methanol-d_4) of ***rac*-5e**.

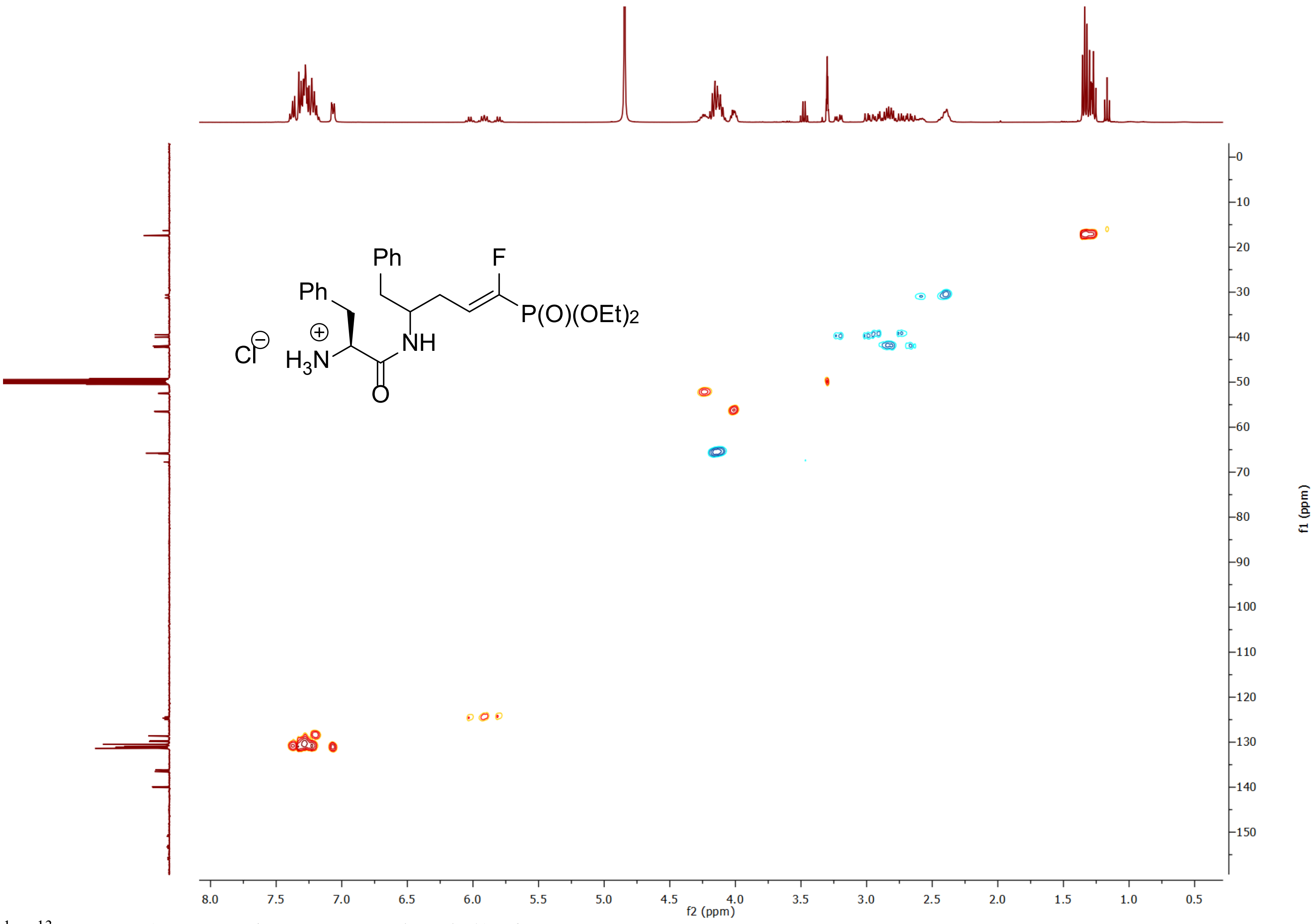

^{1}H-^{13}C HSQC (400 MHz /101 MHz, Methanol-d_4) of ***rac*-5e**.

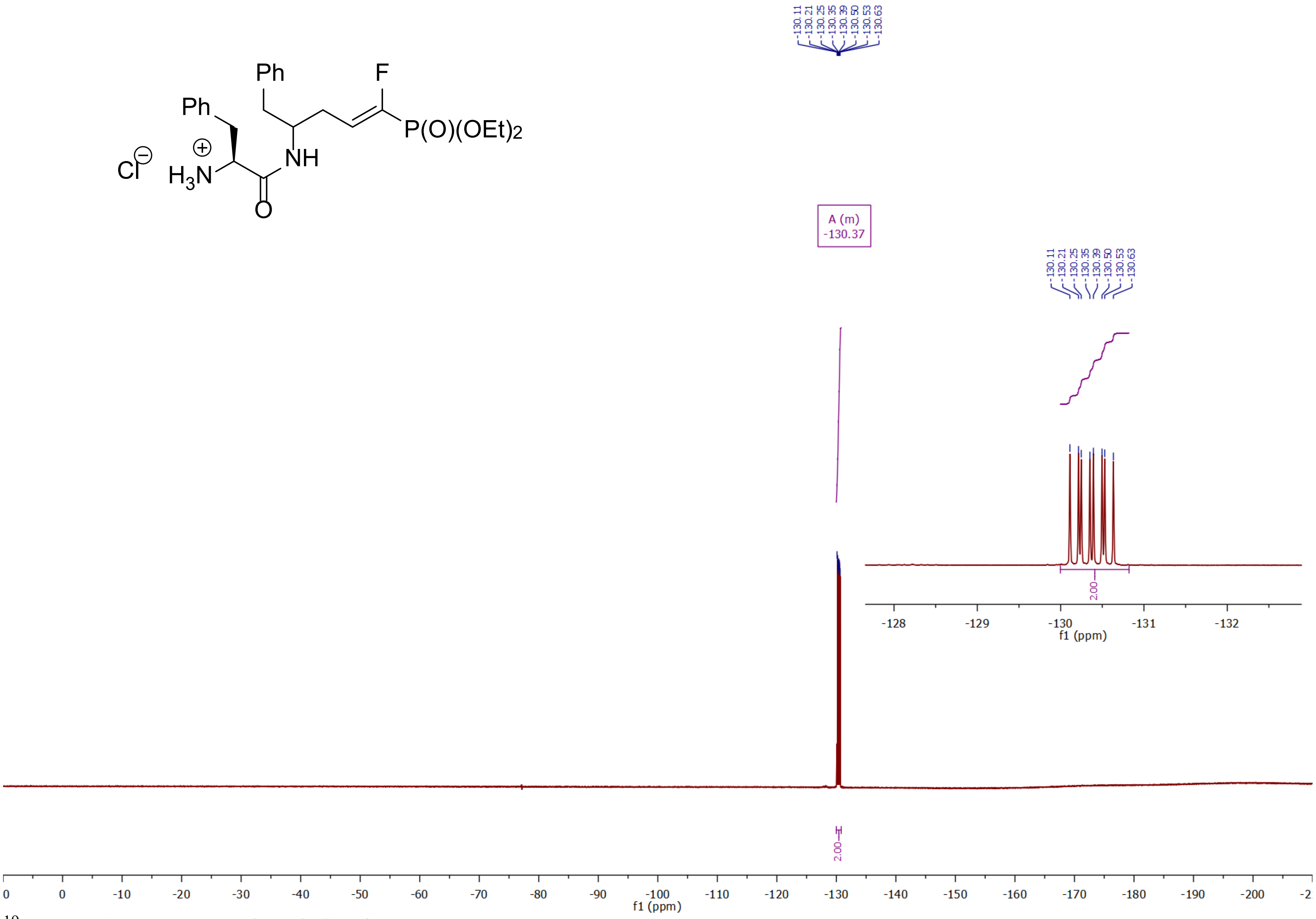

^{19}F NMR (377 MHz, Methanol-d_4) of ***rac*-5e**.

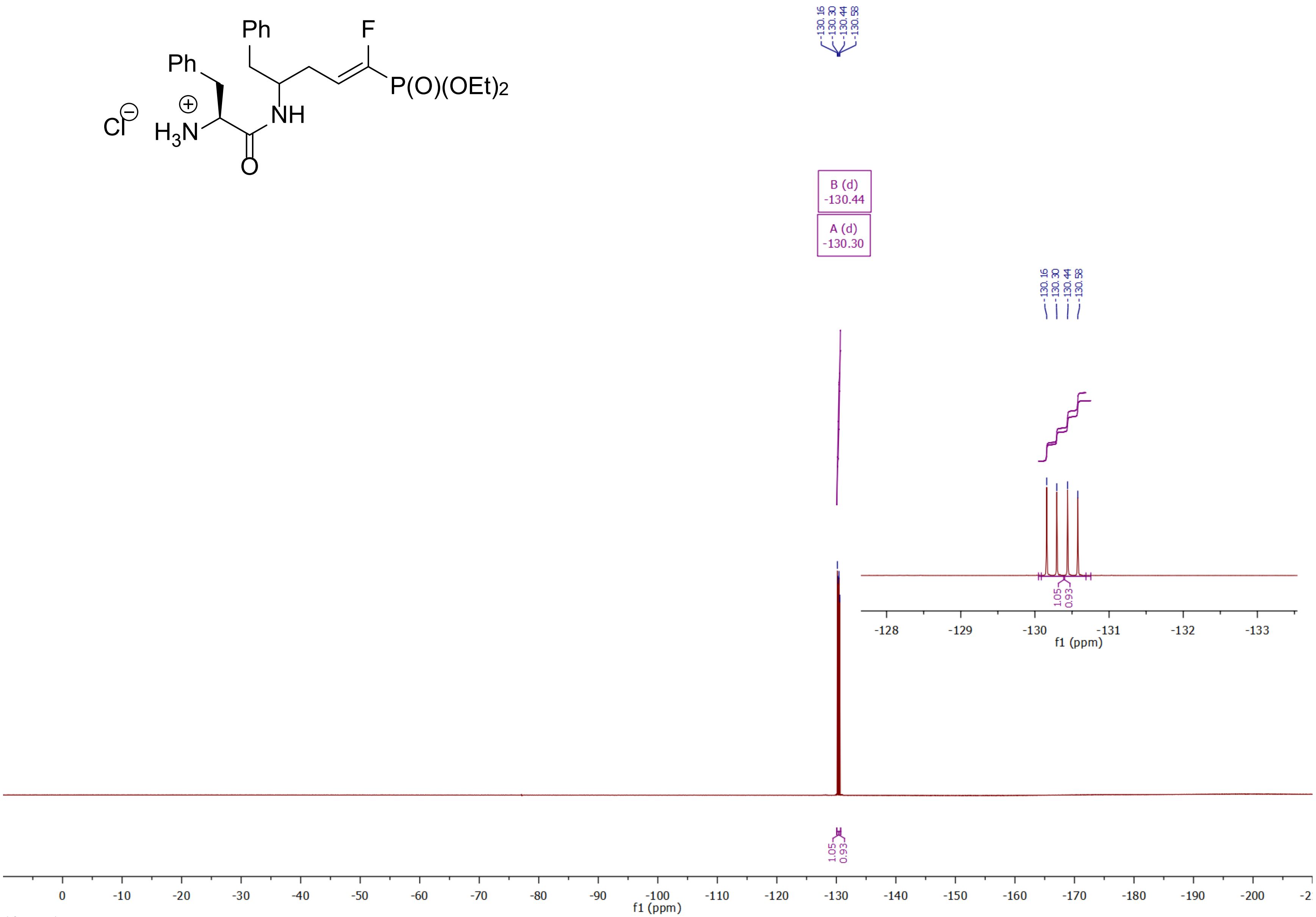

$^{19}F\{/^{1}H\}$ NMR (377 MHz, Methanol-d_4) of ***rac*-5e**.

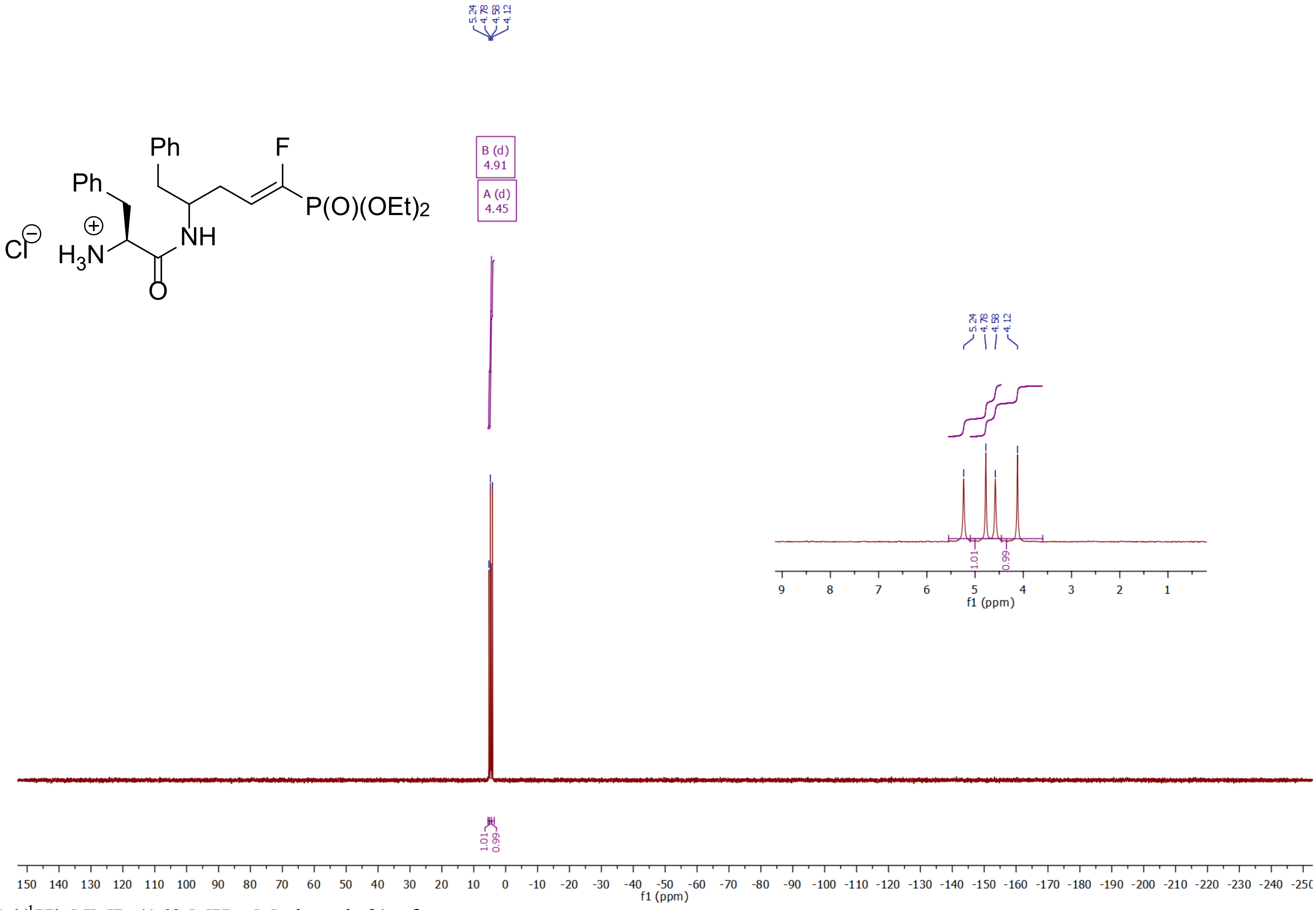

$^{31}P\{/^{1}H\}$ NMR (162 MHz, Methanol-d_4) of ***rac*-5e**.

2. Fluorine Interaction Analysis

Compounds (S,S)-5b and (S,S)-5c (Table S1, Table S2)

The fluorine atoms are positioned proximal to TRP 405 at distances of 3.10–3.11 Å to the indole nitrogen-hydrogen (N-H) atom, 3.66–3.71 Å from the indole nitrogen atom, and 3.23–3.28 Å from the indole benzene ring π-electron system. These distances suggest stabilizing electrostatic interactions between the electronegative fluorine atom and the indole aromatic system. The electron-withdrawing nature of fluorine enhances the electrostatic potential around the fluorine, promoting favorable van der Waals contacts and electrostatic attractions with the polarized indole N-H group and its π-system. The extended contact to the indole nitrogen (3.66–3.71 Å) indicates electrostatic stabilization without direct hydrogen bonding geometry at these distances.

Compounds (S,S)-5d and (S,S)-5e (Table S1, Table S2)

The broader substitution patterns (phenyl and benzyl groups, respectively) extend the interaction network significantly. The fluorine atoms contribute to binding stabilization through: (i) hydrogen bonding and electrostatic interactions with aromatic residues (TRP 405), (ii) electrostatic attractions with polar residues (HIS 381, GLN 228), and (iii) optimization of van der Waals contacts within the binding pocket.

Compound (S,S)-5d (Table S1, Table S2)

The fluorine atom engages in multiple stabilizing interactions with the binding pocket. Notably, the fluorine establishes a hydrogen bonding contact with the indole nitrogen atom of TRP 405 at approximately 2.84 Å (characteristic of N-H···F hydrogen bonding), alongside a close van der Waals contact with the indole N-H hydrogen at 1.94 Å. The fluorine also maintains favorable electrostatic interactions with the indole benzene ring at 3.53 Å, and engages with polar residues HIS 381 (3.58 Å) and GLN 228 (3.07–3.34 Å), establishing a robust and multivalent binding network. This combination of hydrogen bonding, van der Waals contacts, and electrostatic interactions creates particularly favorable binding complementarity.

Compound (S,S)-5e (Table S1, Table S2)

The benzyl substitution pattern similarly facilitates favorable interactions with the protein. The fluorine maintains a close contact with the TRP 405 indole N-H hydrogen at 2.10 Å and engages in weak hydrogen bonding with the indole nitrogen at 3.03 Å (characteristic of N-H···F hydrogen bonding). Additionally, the fluorine coordinates favorable electrostatic interactions with HIS 381 (3.57 Å) and GLN 228 (2.86–3.15 Å), extending the binding network. The N-H···F hydrogen bond with GLN 228 at (2.86 Å to hydrogen and 3.15 Å to hydrogen) is characteristic of moderate hydrogen bonding.

Table S1. *Potential interactions of fluorine atom in compounds* ***5a-e***.

Structure	Fluorine neighbors	Potential interactions
(*S*)-**5a** R = H	-	-
(*S,S*)- **5b** R = Me	TRP 405	3.10 Angstrom to hydrogen atom of indole's ring, 3.66 Angstrom to nitrogen of indole's ring, 3.28 Angstrom to indole's benzene ring,
(*S,R*)- **5b** R = Me	-	-
(*S,S*)- **5c** R = $CH_2CH(CH_3)_2$	TRP 405	3.11 Angstrom to hydrogen atom of indole's ring, 3.71 Angstrom to nitrogen of indole's ring, 3.23 Angstrom to indole's benzene ring
(*S,R*)- **5c** R = $CH_2CH(CH_3)_2$	-	-
(*S,S*)- **5d** R = Ph	TRP 405; HIS 381; GLN 228	1.94 Angstrom to hydrogen atom of indole's ring, 2.84 Angstrom to the nitrogen of indole's nitrogen atom TRP 405, 3.53 angstrom to indole's benzene ring TRP 405; 3.58 angstrom to hydrogen of HIS 381 residue; 3.07 angstrom to hydrogen of GLN 228 residue and 3.34 angstrom to nitrogen of amine group of GLN 228 residue
(*S,R*)- **5d** R = Ph	-	-
(*S,S*)- **5e** R = CH_2Ph	TRP 405; HIS 381; GLN 228	2.10 Angstrom to hydrogen atom of indole's ring of TRP 405, 3.03 Angstrom to nitrogen of indole's ring; 3.57 angstrom to hydrogen of HIS 381 residue; 2.86 angstrom to hydrogen of GLN 228 residue and 3.15 angstrom to nitrogen of amine group of GLN 228 residue
(*S,R*)- **5e** R = CH_2Ph	-	-

Table S2. *Potential types of interactions of fluorine atom in compounds* ***5a-e****.*

**Structure**	**Fluorine contact**	**Key Distance (Å)**	**Interaction type**
(*S*)-**5a** R = H	None	-	-
(*S,S*)- **5b** R = Me	TRP 405	F···H: 3.10;<br>F···N: 3.66;<br>F···π: 3.28	Electrostatic
(*S,R*)- **5b** R = Me	None	-	-
(*S,S*)- **5c** R = $CH_2CH(CH_3)_2$	TRP 405	F···H: 3.11;<br>F···N: 3.71;<br>F···π: 3.23	Electrostatic
(*S,R*)- **5c** R = $CH_2CH(CH_3)_2$	None	-	-
(*S,S*)- **5d** R = Ph	TRP 405<br><br>HIS 381<br><br>GLN 228	F···H: 1.94;<br>F···N: 2.84 (H-bond); F···π: 3.53;<br><br>F···H: 3.58;<br><br>F···H: 3.07;<br>F···N: 3.34	H-bonding + Electrostatic
(*S,R*)- **5d** R = Ph	None	-	-
(*S,S*)- **5e** R = CH_2Ph	TRP 405<br><br>HIS 381<br><br>GLN 228	F···H: 2.10;<br>F···N: 3.03 (H-bond);<br><br>F···H: 3.57;<br><br>F···H: 2.86;<br>F···N: 3.15 (H-bond)	H-bonding + Electrostatic
(*S,R*)- **5e** R = CH_2Ph	None	-	-
